# Cars2‐Mediated Cysteine Catabolism Drives Brown Fat Development and Thermogenesis Through Persulfidating EBF2

**DOI:** 10.1002/advs.202522690

**Published:** 2026-03-18

**Authors:** Xin Peng, Hongyong Song, Yin Long, Kaimin Wu, Yinkun Fu, Wenkai Zhou, Wenyan Li, Zhe Shi, Xu‐Yun Zhao

**Affiliations:** ^1^ Ruijin Hospital Lu Wan Branch Shanghai Jiao Tong University School of Medicine Shanghai China; ^2^ Department of Biochemistry and Molecular Cell Biology, Shanghai Key Laboratory for Tumor Microenvironment and Inflammation Key Laboratory of Cell Differentiation and Apoptosis of National Ministry of Education Shanghai Jiao Tong University School of Medicine Shanghai China; ^3^ Department of Oral and Maxillofacial & Head and Neck Oncology Shanghai Ninth People’s Hospital, College of Stomatology Shanghai China; ^4^ Department of Gastrointestinal Surgery Affiliated Hospital of Hebei Engineering University Handan Hebei China

**Keywords:** brown fat, cysteine metabolism, EBF2, PPARγ, protein persulfidation, thermogenesis

## Abstract

Targeting transcriptional regulatory complexes represents a promising strategy for activating thermogenic fat and treating obesity. In this study, we focused on identifying critical, specific metabolic pathways enriched during brown adipocyte differentiation. Through analysis of metabolomics and RNA sequencing data from precursor and mature brown and white adipocytes, we revealed that the cysteine catabolism pathway constitutes a unique metabolic process that is significantly upregulated during brown adipocyte differentiation. We further demonstrated that the cysteine persulfidation synthase Cars2, which is directly induced by EBF2, mediates cysteine catabolism and triggers brown fat protein persulfidation both in vitro and in vivo. Loss of Cars2 in thermogenic fat blocks brown fat formation, resulting in mice exhibiting reduced thermogenesis and energy expenditure. More importantly, Cars2 generates cysteine persulfide and its derivative H_2_S cell‐autonomously induces brown adipogenesis and enhances uncoupled respiration through persulfidation of EBF2. Mechanistically, persulfidated EBF2 facilitates its interaction with PPARγ or BRG1, enhancing recruitment of the EBF2‐PPARγ complex to browning gene promoters, thereby driving brown fat development and boosting thermogenic activity. Furthermore, treatment with the Cars2 coenzyme PLP or an H_2_S donor enhances brown adipocyte function and alleviates obesity progression in mice fed a high‐fat diet, indicating a novel metabolite‐based strategy for obesity treatment.

## Graphical abstract

We demonstrate that Cars2, a cysteine catabolic enzyme in mouse iBAT, is critical for cold tolerance and brown adipocyte differentiation. Through its CPERS activity, Cars2 produces CysSSH/H_2_S to induce EBF2 persulfidation, promoting its interaction with PPARγ and BRG1 to enhance thermogenic gene expression. EBF2 in turn upregulates Cars2, forming a positive feedback loop that drives the thermogenic browning phenotype.

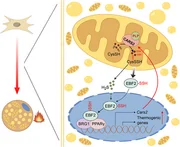

## 1 Introduction

Obesity has become a serious problem to public health worldwide, accompanied by changes in glucose and lipid metabolism and resulting in metabolic diseases [1, 2, 3, 4, 5]. The accumulation of fat in peripheral tissues develops insulin resistance and contributes to obesity progression. In addition to white adipocytes, which store energy, brown and beige adipocytes consume energy through dissipating energy in the form of heat via uncoupling protein‐1 (Ucp1) [6]. Therefore, strategies aimed at increasing Ucp1 expression to enhance energy expenditure in brown and beige adipocytes, or at promoting their differentiation, hold promise for the treatment of obesity and associated metabolic diseases.

The commitment and differentiation of brown adipocytes are regulated by a cascade of key transcription factors. Among them, early B‐cell factor 2 (Ebf2) is an essential mediator of both thermogenic adipocyte commitment and differentiation process [7]. It controls the orientation of a multipotent progenitor cell population toward the brown or beige adipocyte lineage and specifically marks and regulates the browning profile during differentiation [8, 9]. Recent studies have demonstrated that EBF2 recruits DPF3‐anchored BAF complexes to chromatin, thereby activating brown fat identity genes [7]. It also forms a ribonucleoprotein complex with brown fat lncRNA 1 (Blnc1) to exert a feedforward stimulation on the thermogenic gene program [10]. More importantly, EBF2 recruits peroxisome proliferator‐activated receptor gamma (Pparγ), a master regulator of adipocyte differentiation, to brown fat‐specific gene targets, and enhances the transcriptional activity of the ERRα/PGC1α complex to drive the expression of Ucp1 and other thermogenic genes [11, 12].

Recent studies indicate that metabolite‐based sensing signals represent a novel mechanism regulating adipose tissue homeostasis. Several metabolic sensing pathways are involved in the determination of white and brown fat identity [13, 14] as well as intercellular communication within adipose tissue [15, 16], highlighting the indispensable role of metabolite‐mediated pathways in regulating adipocyte thermogenic activity. However, the specific mechanisms by which metabolites mediate brown adipocyte differentiation remain largely unexplored.

Cysteine is a key nonessential amino acid for protein synthesis, which can be metabolized in cells to generate a variety of derivatives, such as glutathione (GSH), taurine, and hydrogen sulfide (H_2_S), that exert critical physiological functions [17]. Dietary cysteine promotes energy expenditure and suppresses food intake, thereby driving body fat loss through FMRFamide signaling in both fruit flies and mice [18]. H_2_S, a gaseous signaling molecule produced via cysteine catabolism, exerts cytoprotective effects at low concentrations. It regulates vascular tone and blood pressure, and confers beneficial effects in reducing liver injury and protecting against cardiovascular diseases [19, 20]. Notably, cysteine residues in proteins can undergo persulfidation mediated by H_2_S [21, 22]. Recent studies have indicated that protein persulfidation is a common post‐translational modification, often accompanied by alterations in protein physiological functions [22]. H_2_S can be produced by gut microbiota as well as endogenous enzymes, including cystathionine γ‐lyase (CSE), cystathionine β‐synthase (CBS), and 3‐mercaptopyruvate sulfur transferase (3‐MST) [23]. Among these, CSE is predominantly expressed in peripheral tissues, whereas CBS and 3‐MST are mainly expressed in the central nervous system [22, 24].

Researchers have reported that recombinant cysteinyl‐tRNA ligase from *E. coli* (EcCARS) can catalyze the cleavage of a sulfur atom from one cysteine and transfer it to another cysteine to form cysteine persulfide (CysSSH) [25]. In mammals, cysteinyl‐tRNA ligase 2 (Cars2), which is located specifically in mitochondria, can catalyze the synthesis of cysteine into CysSSH. This process is independent of its cysteinyl‐tRNA ligase activity but relies on its cysteine persulfide synthase (CPERS) activity. In the cytoplasm, CysSSH can participate in protein synthesis, ultimately leading to the formation of protein persulfidation, which suggests a critical role for CARS2 in mediating protein persulfidation. Additionally, the thioredoxin/thioredoxin reductase system in the cytoplasm desulfurizes CysSSH and generates H_2_S, which may further promote protein persulfidation [25]. Although brown adipocytes contain abundant mitochondria, the specific role of CARS2 in thermogenesis in brown adipose tissue remains unclear and requires further investigation.

In this study, we demonstrated that the expression of Cars2, an enzyme mediating cysteine catabolism in mouse interscapular brown adipose tissue (iBAT), is critical for maintaining core body temperature following cold exposure. Additionally, Cars2 plays a pivotal role in brown adipocyte (BAC) differentiation by inducing thermogenic gene expression and enhancing uncoupled respiration. Through its cysteine persulfide synthase (CPERS) activity, Cars2 regulates the production of CysSSH and its derivative H_2_S. These molecules in turn induce persulfidation of EBF2, a key transcriptional factor in BAT development, thereby facilitating the interaction of EBF2 with PPARγ or BRG1. Notably, Ebf2 also promotes Cars2 expression, forming a positive feedback loop that drives the browning phenotype. Our findings reveal that Cars2‐mediated cysteine persulfide production constitutes a unique and critical metabolic sensing pathway in mammalian bioenergetic homeostasis.

## 2 Results

### 2.1 Cysteine Catabolism Is Highly Enhanced During Brown Adipocyte Differentiation and Contributes to the Browning Phenotype

**FIGURE 1:**
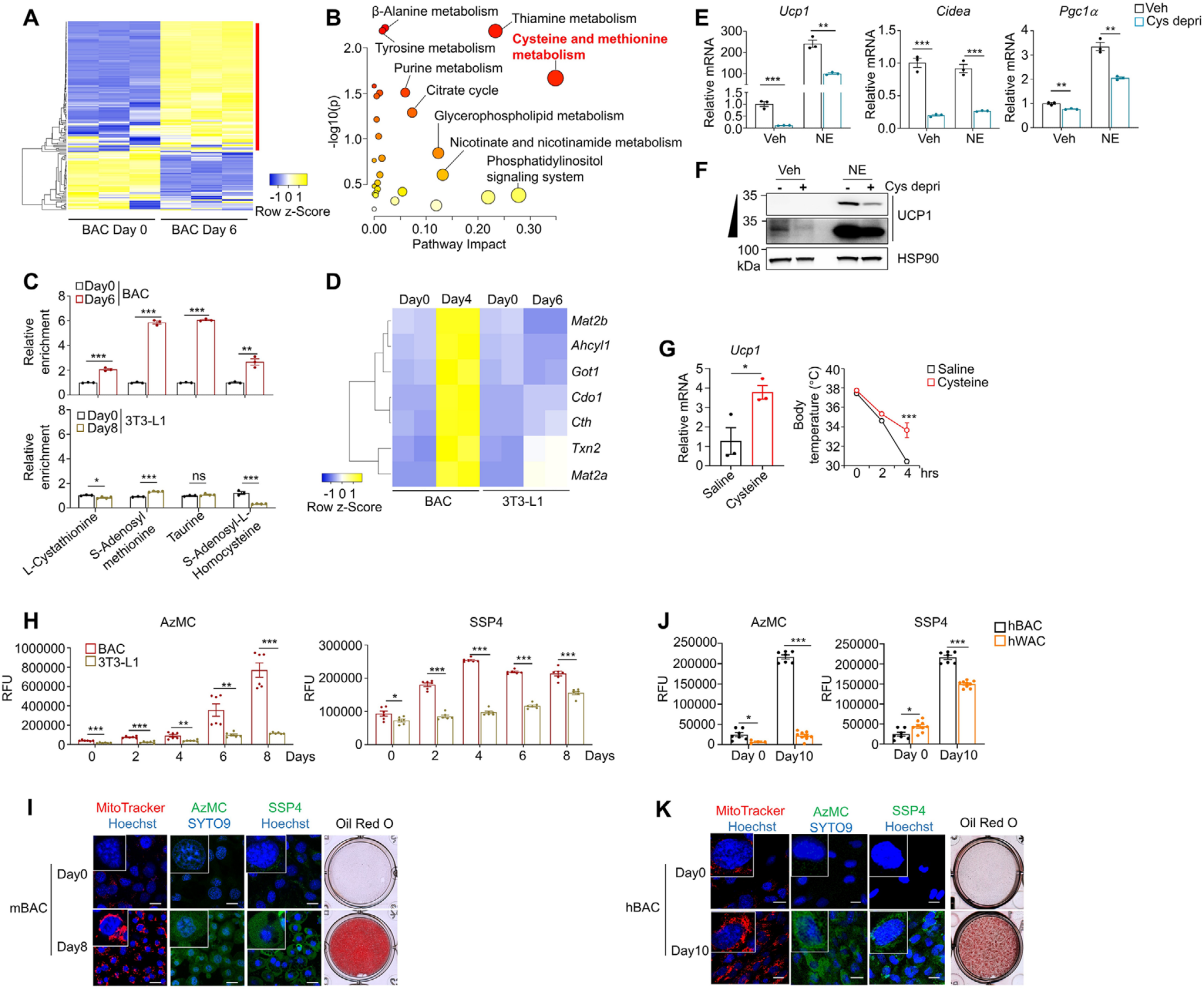
Cysteine metabolism was increased during brown adipocyte differentiation and contributed to the browning phenotype. (A) Heatmap of metabolites of mouse brown adipocytes (BAC, n = 3) before (Day 0) and after (Day 6) differentiation. (B) Pathway enrichment analysis of upregulated metabolites of mouse BAC before and after differentiation. (C) Changes in intermediate metabolites of cysteine metabolism in mouse BAC (n = 3) and white adipocytes (3T3‐L1) before (n = 3) and after differentiation (n = 4). (D) Expression of genes associated with cysteine metabolism in mouse BAC and 3T3‐L1 cells before and after differentiation. (E‐F) The culture medium of BAC (n = 3) was deprived of cystine for 24 h after differentiation. The expression of browning genes was determined after treated with vehicle (Veh) or norepinephrine (NE, 1 µM, 6 h) via qPCR (E) and western blotting (F). (G) Wild‐type mice were fasted for 16 h and intraperitoneally injected with saline (n = 3) or cysteine (1 mg/kg, n = 3). The mice were then immediately subjected to acute cold exposure (4°C) in fasting condition. Rectal temperature was monitored, and *Ucp1* expression in interscapular brown adipose tissue (iBAT) was determined by qPCR. (H) Hydrogen sulfide and sulfane sulfur levels at the indicated time points during the differentiation of mouse BAC (n = 6) and 3T3‐L1 (n = 6) were determined by corresponding probes (AzMC, for hydrogen sulfide, and SSP4, for sulfane sulfur), and the relative fluorescence intensity was measured with a microplate reader. (I) The mitochondrial content, hydrogen sulfide, sulfane sulfur levels and lipid droplets of the mouse BAC before (Day 0) and after (Day 6) differentiation were stained with MitoTracker, AzMC, SSP4 and Oil red O, respectively; the nucleus was stained with Hoechst 33342 (for MitoTracker and SSP4) and SYTO9 (for AzMC); and fluorescence images were taken via confocal microscopy. Scale bar = 20 µm. (J) Hydrogen sulfide and sulfane sulfur levels of human brown‐like adipocytes (hBAC) and human white adipocytes (hWAC) before (Day 0) and after (Day 10) differentiation were determined by corresponding probes (AzMC for hydrogen sulfide and SSP4 for sulfane sulfur, n = 4∼9), and the relative fluorescence intensity was measured via a microplate reader. (K) The mitochondrial content, hydrogen sulfide, sulfane sulfur and lipid droplets of hBAC before (Day 0) and after (Day 10) differentiation were stained with MitoTracker, AzMC, SSP4 and Oil red O, respectively; the nucleus was stained with Hoechst 33342 (for MitoTracker and SSP4) and SYTO9 (for AzMC); and fluorescence images were taken via confocal microscopy. Scale bar = 20 µm. Data are shown as the mean ± SEM. *p < 0.05, **p < 0.01, and ***p < 0.001, by unpaired Student's *t*‐test (C, E, left panel of G, H, and J); by two‐way ANOVA with multiple comparisons (right panel of G); ns, nonsignificant.

Brown adipocytes undergo dramatic metabolic reprogramming during differentiation. Such remodeling of metabolic homeostasis may affect the activity of transcription factors that mediate brown adipogenesis. To identify the unique metabolic pathways regulated during brown adipocyte differentiation, we conducted metabolomics analyses combined with RNA sequencing to compare the profiles before and after differentiation in both brown and white adipocytes. Brown adipocytes (BACs) and 3T3‐L1 white adipocytes were differentiated to a comparable extent (Figure S1A). In differentiated BAC, we observed a significant alteration of metabolites (Figure 1A). Clustering analysis revealed that cysteine and methionine metabolic processes represent the most prominently upregulated metabolic pathways during BAC differentiation (Figure 1B). Metabolites involved in cysteine synthesis, including S‐adenosylmethionine and S‐adenosyl‐L‐homocysteine, which serve as precursors for homocysteine and L‐cystathionine, as well as the cysteine derivative taurine, are highly elevated in mature brown adipocytes but not in white adipocytes (Figure 1C). In addition, the expression of genes involved in cysteine synthesis, including *Mat2a*, *Mat2b*, *Ahcyl1*, and *Got1*, as well as those mediating cysteine degradation, such as *Cdo1* and *Txn2*, was significantly and specifically upregulated in brown adipocytes following differentiation (Figure 1D). To investigate whether cysteine or its derivatives are essential for brown adipocyte function, we depleted cystine, an oxidized form of cysteine, from the culture medium of mature brown adipocytes. Cystine deprivation led to a drastic reduction in intracellular cysteine levels. However, it did not affect cellular lipid storage and viability (Figure S1B–D). In contrast, the expression of browning‐associated genes *Ucp1*, *Cidea*, and *Pgc1α* was downregulated, and norepinephrine (NE)‐induced upregulation of *Ucp1* and *Pgc1α* expression in brown adipocytes was significantly suppressed (Figure 1E,F and S1E). To investigate whether cysteine is sufficient to induce iBAT thermogenesis in vivo, we first fasted mice to reduce circulating cysteine levels (Figure S1F). These fasted mice were then directly administered cysteine or a vehicle control, followed by cold exposure. Cysteine administration preserved core body temperature in fasted mice under acute cold exposure and upregulated *Ucp1* expression in iBAT (Figure 1G). These results further confirm the critical role of cysteine metabolism in regulating iBAT thermogenesis. Cysteine catabolism involves three major pathways: condensation with glutamate and glycine to form glutathione (GSH); oxidation followed by decarboxylation to generate taurine; and breakdown into hydrogen sulfide (H_2_S), a key gasotransmitter that modulates diverse cellular functions. However, the role of H_2_S in regulating brown adipocyte differentiation remains largely unexplored (Figure S1G). To measure intracellular H_2_S concentrations, we utilized the H_2_S‐specific probe AzMC, which selectively binds to intracellular H_2_S. Additionally, the SSP4 probe was employed to detect sulfane sulfur species (RSSH), including CysSSH derived from cysteine. Cystine deprivation in mature BACs significantly reduced the fluorescence signals from both the AzMC and SSP4 probes, thereby confirming the specificity of these probes for detecting their respective targets (Figure S1H). More intriguingly, both H_2_S and RSSH are significantly more abundant during brown adipocyte differentiation compared to 3T3‐L1 adipocyte differentiation (Figure 1H,I). Notably, a marked induction in the production of H_2_S and RSSH was observed in mature human brown‐like adipocytes (hBACs) relative to their precursor cells. In contrast, only a modest induction of these molecules was detected in human white adipocytes (hWACs) when compared with their precursor counterparts (Figure 1J,K). Following their production, H_2_S diffused throughout the entire cell, whereas RSSH was predominantly localized in the cytosol, with low levels detected in the nucleus and mitochondria and no signal observed in lipid droplets (Figure S1I). Metabolomic analysis showed that taurine content was elevated in differentiated BACs (Figure 1C), while total glutathione (GSH) levels remained unaltered (Figure S1J). Although studies have shown that taurine supplementation counteracts obesity and potentially induces WAT browning [26], our results indicate that taurine treatment had a very limited effect on promoting BAC differentiation and Ucp1 expression (Figure S1K,L). These findings indicate that cysteine catabolism represents a unique metabolic process specifically induced during brown adipocyte differentiation that plays a critical role in regulating the thermogenic activity of brown adipocytes both in vitro and in vivo.

### 2.2 Cars2 is Transcriptionally Induced by EBF2 During Brown Adipocyte Differentiation and Mediates Brown Fat Protein Persulfidation

**FIGURE 2:**
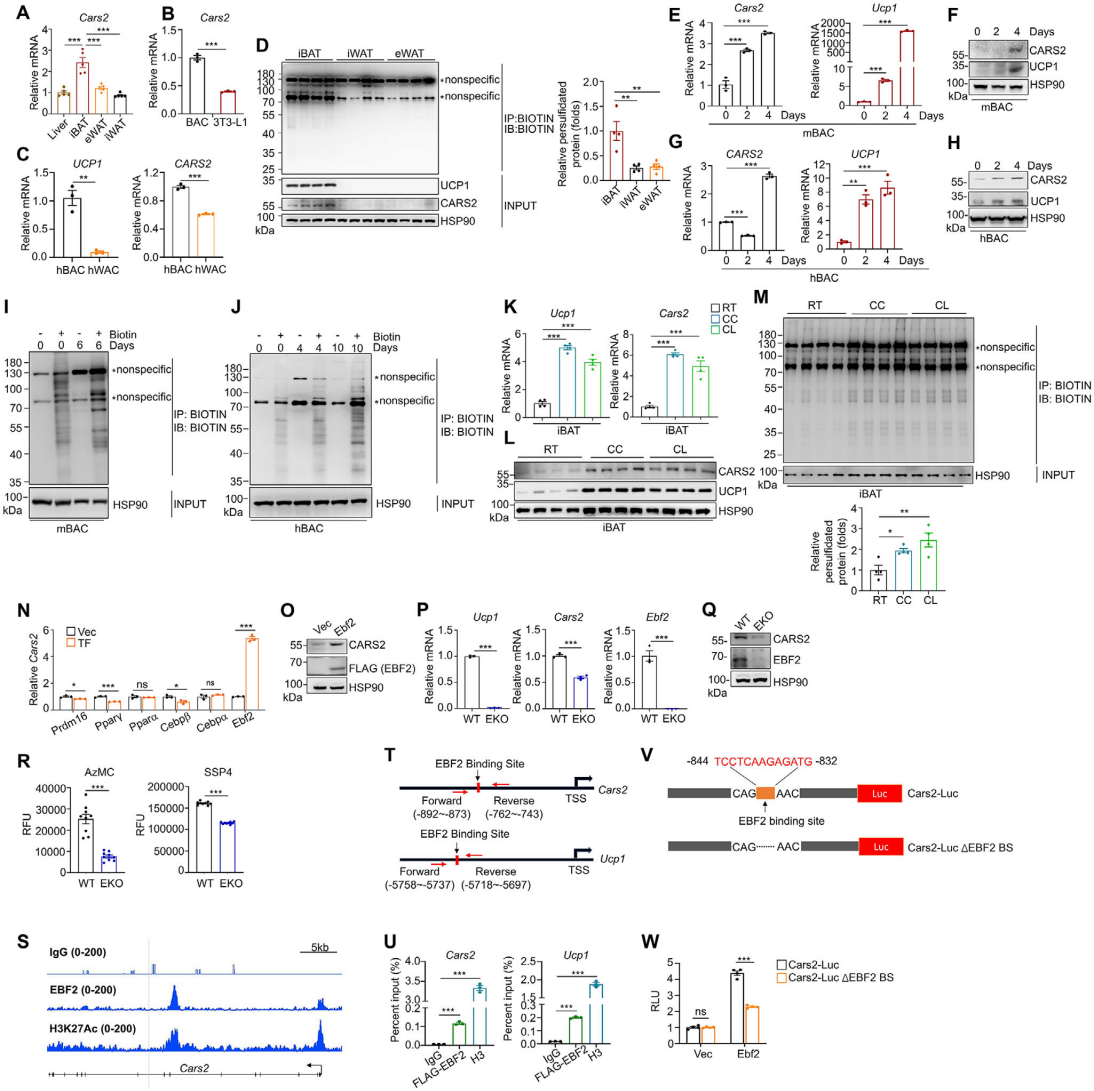
EBF2 induces Cars2 expression to increase persulfidated protein levels in differentiated and activated brown adipocytes. (A) QPCR analysis of *Cars2* expression in the liver, interscapular brown adipose tissue (iBAT), epididymal white adipose tissue (eWAT) and inguinal white adipose tissue (iWAT) of wild‐type mice (n = 5) kept at room temperature. (B) *Cars2* expression in differentiated mouse brown adipocytes (BAC, n = 3) and white adipocytes (3T3‐L1, n = 3) was determined by qPCR. (C) *UCP1* and *CARS2* expression in differentiated human brown‐like adipocytes (hBAC, n = 3) and human white adipocytes (hWAC, n = 3) was determined by qPCR. (D) The content of persulfidated protein in iBAT, iWAT and eWAT of wild‐type mice was determined by western blot after the biotin‐HPDP switch assay (left), and the relative amounts of persulfidated protein were determined by the density of the bands of persulfidated protein, normalized to that of HSP90. Then, the fold changes were calculated by dividing the relative persulfidated protein in the iWAT and eWAT by that in the iBAT (right). (E–H) Cars2 and Ucp1 mRNA and protein levels at the indicated time points during mouse BAC (E,F, n = 3) and human BAC (G‐H, n = 3) differentiation were determined by qPCR and western blotting. (I,J) The persulfidated protein content in mouse BAC (I) and human BAC (J) before and after differentiation was determined by western blot after the biotin‐HPDP switch assay. (K‐L) mRNA and protein were extracted from the iBAT of mice kept at room temperature (RT, n = 4), subjected to chronic cold (CC, n = 4), or treated with the β‐androgenic agonist CL316243 (CL, n = 4), and the expression levels of Cars2 and Ucp1 were determined by qPCR (K) and western blotting (L). (M) The persulfidated protein content in the iBAT of RT‐, CC‐ and CL‐treated mice was detected via western blotting after the biotin‐HPDP switch assay (top), and the relative amounts of persulfidated protein were determined by the density of the bands of persulfidated protein, normalized to that of HSP90. Then, the fold changes were calculated by dividing the relative persulfidated protein in the iBAT of cold‐ and CL‐treated mice by that of RT‐treated mice (bottom). (N) A set of transcription factors mediating BAC differentiation were overexpressed in BAC, and the mRNA level of *Cars2* in differentiated BAC (n = 3) was determined via qPCR. (O) Western blot analysis of CARS2 in Ebf2‐overexpressing BAC after differentiation. (P,Q) mRNA and protein levels of Cars2 in wild‐type (WT, n = 3) and *Ebf2* knockout (EKO, n = 3) mouse BAC after differentiation were measured via qPCR (P) and western blotting (Q). (R) Hydrogen sulfide and sulfane sulfur levels were analyzed via AzMC and SSP4 staining, respectively, in WT and *Ebf2*‐knockout mouse BAC (n = 9) after differentiation. (S) The binding activity of EBF2 to the *Cars2* promoter in differentiated mouse BAC was examined by Cut & Tag sequencing analysis. (T) Diagram showing the EBF2 binding site on the *Cars2* and *Ucp1* promoters and the locations of the ChIP‐qPCR primers. (U) The binding activity of overexpressed FLAG‐EBF2 to the *Cars2* and *Ucp1* promoters in differentiated mouse BAC (n = 3) was examined by ChIP‐qPCR analysis. (V) Diagram showing the *Cars2* promoter‐containing luciferase plasmid with the predicted EBF2 binding site (Cars2‐Luc) and its deletion luciferase plasmid (Cars2‐Luc ΔEBF2 BS). (W) Cars2‐Luc or Cars2‐Luc ΔEBF2 BS plasmids were co‐transfected with vector or Ebf2‐expressing plasmids into 293T cells (n = 4), and the relative luciferase activity was measured via a microplate reader. Data are shown as the mean ± SEM. *p < 0.05, **p < 0.01, and ***p < 0.001, by unpaired Student's *t*‐test (B, C, N, P, R and W); by one‐way ANOVA with Dunnett's test (A, D, E, G, K, M and U); ns, nonsignificant.

H_2_S synthesis from cysteine is catalyzed by CBS and CSE [23]. However, both *Cbs* and *Cse* are barely detectable in adipose tissue (Figure S1M), suggesting that H_2_S production in mature brown adipocytes is not primarily mediated by these enzymes. To further validate this, we treated BACs with DL‐propargylglycine (PAG), a specific CSE inhibitor, during differentiation. PAG treatment did not reduce H_2_S and RSSH levels. Instead, it slightly induced both the levels of these metabolites and UCP1 expression (Figure S1N,O). Recently, Takaaki Akaike and colleagues reported that cysteine can be efficiently catalyzed by cysteinyl‐tRNA synthetases (CARSs) to synthesize CysSSH. CARS family members, including Cars and Cars2, catalyze the cleavage of a sulfur atom from one cysteine molecule and its transfer to another cysteine, thereby forming CysSSH [25]. This CysSSH can be directly incorporated into proteins during translation, leading to the formation of protein persulfidation. As previously reported, the thioredoxin/thioredoxin reductase system (Trx/TrxR) is required for the production of H_2_S from persulfide residues [25], which suggests that H_2_S in BACs is generated from CysSSH (Figure S1G). Interestingly, *Cars2* was found to be enriched in iBAT, a tissue rich in mitochondria, whereas its levels were lower in WAT and other metabolic tissues, including the liver, muscle, and heart (Figure 2A and S2A). The expression of *Cars2* was also higher in mature mouse and human BACs compared to 3T3‐L1 adipocytes and human WACs, which is consistent with the higher levels of H_2_S and RSSH observed in mouse and human BACs relative to 3T3‐L1 adipocytes and human WACs (Figure 2B,C).

Previous studies have demonstrated that Cars2 acts as a principal cysteine persulfide synthase, mediating the formation of protein persulfidation [25]. Persulfidated proteins can be conjugated with biotin‐HPDP in a biotin switch assay [27]. Using this approach, we examined the content of persulfidated proteins in brown and white adipose tissues. Consistent with the expression pattern of Cars2, persulfidated proteins were more abundant in iBAT compared to epididymal white adipose tissue (eWAT) or inguinal white adipose tissue (iWAT) (Figure 2D). During the differentiation of mouse brown adipocytes, both the mRNA and protein levels of Cars2 increased gradually, and this trend showed a strong association with the expression profile of Ucp1 (Figure 2E,F and S2B). In addition, CARS2 expression during human BAC differentiation was similar to that in mice (Figure 2G,H and S2C). Next, we investigated the content of persulfidated proteins in BACs during differentiation. Our findings revealed that protein persulfidation levels in mature brown adipocytes were significantly higher compared to those in precursor cells, both in mouse and human models (Figure 2I,J and S2D). Additionally, both mRNA and protein expression levels of Cars2 and Ucp1 in iBAT were significantly upregulated under chronic cold exposure and following injection of CL316243 (CL), a β3‐adrenergic receptor agonist (Figure 2K,L and S2E). The induction of *Cars2* expression in eWAT and iWAT was relatively modest upon cold and CL treatment (Figure S2F). Consistently, the content of persulfidated protein was significantly increased in iBAT activated by chronic cold or CL treatment (Figure 2M). To explore the upstream regulator of Cars2 in BAC, we performed a screen using classic transcription factors that participate in brown adipogenesis [8, 28]. The results revealed that among five crucial transcription factors associated with BAC differentiation, only the overexpression of Ebf2 significantly upregulated Cars2 expression at both the mRNA and protein levels (Figure 2N,O and S2G). Furthermore, knocking out *Ebf2* in the BAC decreased Ucp1 and Cars2 expression at both the mRNA and protein levels (Figure 2P,Q and S2H). Depletion of *Ebf2* also decreased H_2_S and RSSH production (Figure 2R). Next, we performed a Cut & Tag sequencing assay to investigate whether EBF2 transcriptionally regulates *Cars2* expression. Notably, our results demonstrated that EBF2 directly binds to the promoter region of the *Cars2* gene (Figure 2S). Chromatin immunoprecipitation (ChIP) assay further confirmed the EBF2 binding site at −844 to −832 bp from the transcriptional start site (Figure 2T,U). This finding was further corroborated by a luciferase assay employing the EBF2‐binding‐site mutants (Figure 2V,W). Overall, these results reveal that EBF2 is a transcriptional regulator of Cars2, which induces Cars2 expression during brown adipocyte differentiation.

### 2.3 Deficiency of Cars2 in Thermogenic Adipocytes Inhibits Protein Persulfidation and Impairs Thermogenesis and Lipid Homeostasis

**FIGURE 3:**
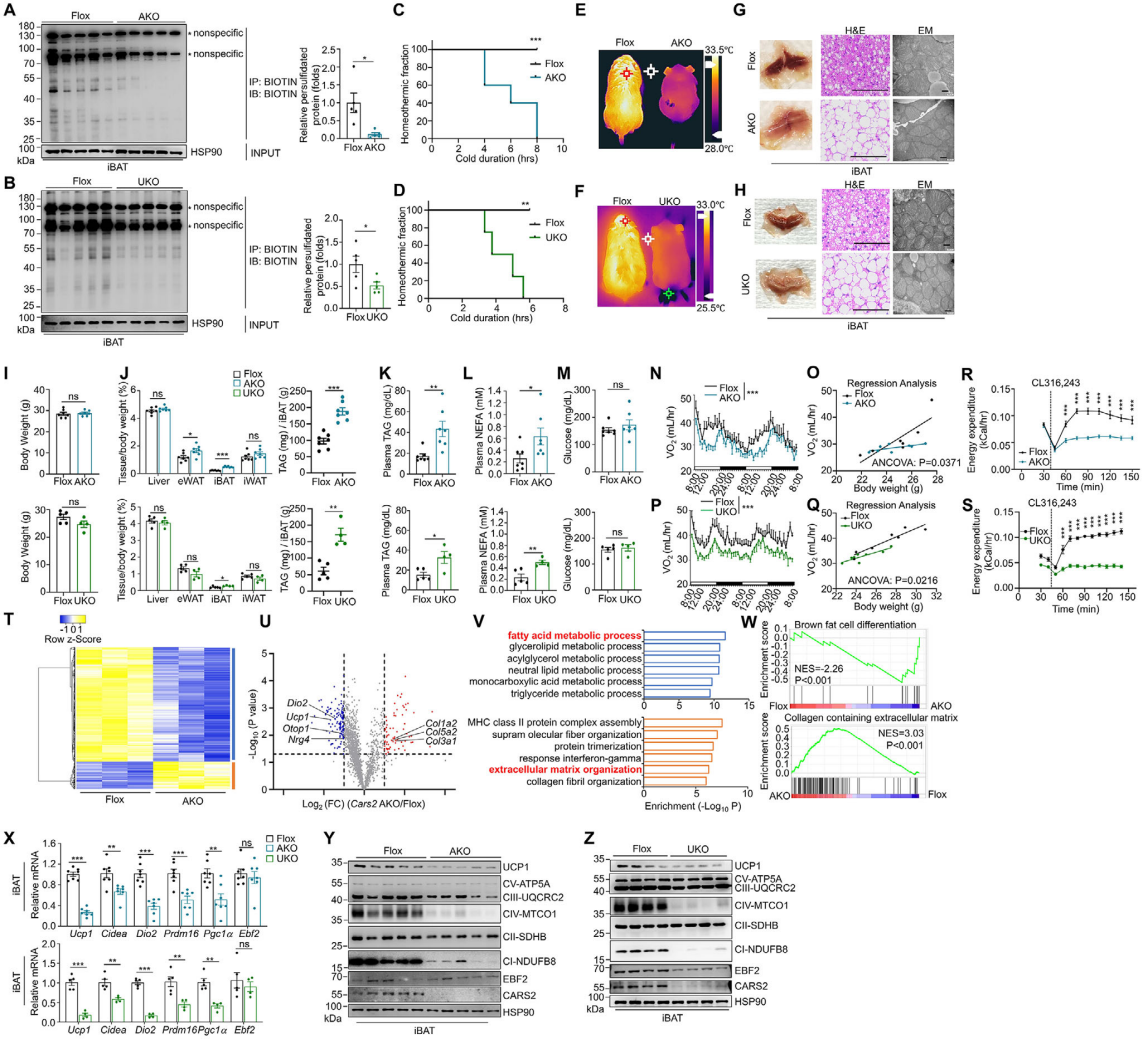
Cars2 ablation impedes the thermogenic activity of BAT. (A,B) The persulfidated protein content in iBAT from *Cars2* Adiponectin‐Cre knockout (A, AKO, n = 5) and Ucp1‐Cre knockout (B, UKO, n = 5) mice and controls (Flox, n = 5) after cold exposure was analyzed via western blotting after the biotin‐HPDP switch assay (left), and the relative amounts of persulfidated protein were determined by the density of the bands of persulfidated protein, normalized to that of HSP90. Then, the fold changes were calculated by dividing the relative persulfidated protein in the iBAT of AKO (A, right) and UKO (B, right) mice by that of control mice. (C,D) The homeothermic fraction (rectal temperature>30°C) of *Cars2* AKO (n = 7) and UKO (n = 4) mice and controls (AKO, n = 7, UKO, n = 5) in a 4°C acute cold exposure experiment. (E‐F) Infrared images of *Cars2* AKO, UKO and control mice after cold exposure for 4 h were taken with an infrared thermal camera. (G‐H) Morphology, H&E staining and electron microscopy images of iBAT from *Cars2* AKO and UKO mice and controls used in C‐D after cold exposure. Scale bar = 100 µm for H&E staining and 500 nm for electron microscopy images. (I–M) Body weights (I), percentage of tissue weights (left, J) and triglyceride (TAG) content in iBAT (right, J), plasma TAG (K), nonesterified fatty acid (NEFA, L) and blood glucose (M) levels of *Cars2* AKO and UKO mice and controls used in C‐D after cold exposure. (N,O) Oxygen consumption curves (N) and regression plots (O) of *Cars2* AKO mice (n = 8) and controls (n = 7) housed at ambient room temperature and measured in metabolic cages. (P‐Q) Oxygen consumption curves (P) and regression plots (Q) of *Cars2* UKO mice (n = 8) and controls (n = 7) housed at ambient room temperature and measured in metabolic cages. (R) Energy expenditure of *Cars2* AKO (n  =  7) and Flox mice (n  =  7) after CL316,243 (1 mg/kg) treatment. (S) Energy expenditure of *Cars2* UKO (n  =  4) and Flox mice (n  =  4) after CL316,243 (1 mg/kg) treatment. (T‐W) RNA sequencing data of iBAT from *Cars2* AKO mice (n = 3) and controls (n = 3) were analyzed by heatmap (T), volcano plot (U), Gene Ontology (GO) analysis (V) and Gene Set Enrichment Analysis (GSEA) (W). (X) mRNA levels of browning genes in the iBAT of *Cars2* AKO and UKO mice and the controls used in C‐D after cold tolerance were determined by qPCR. (Y–Z) Protein from the iBAT of *Cars2* AKO (Y) and UKO (Z) mice and controls used in C‐D after cold exposure was extracted and analyzed via western blotting with the indicated antibodies. Data are shown as the mean ± SEM. *p < 0.05, **p < 0.01, and ***p < 0.001 by unpaired Student's *t*‐test (A, B, I‐M and X); by Log‐rank (Mantel‐Cox) test (C and D); by two‐sided ANCOVA analysis (O and Q); by two‐way ANOVA (N, P, R and S), followed by Fisher's least‐significant difference post hoc test (R and S); ns, nonsignificant.

To investigate the role of Cars2 expression and its associated protein persulfidation in regulating iBAT function, we generated conditional knockout mouse models: one with *Cars2* deleted in all adipose tissues by crossing *Cars2*‐floxed mice with Adiponectin‐Cre transgenic mice (referred to as AKO), and another with *Cars2* specifically deleted in thermogenic adipose tissues by crossing with Ucp1‐Cre transgenic mice (referred to as UKO). In AKO mice, *Cars2* expression is specifically ablated in all adipose tissues, while in UKO mice, its expression is selectively eliminated in thermogenic adipose tissues (Figure S2I). The persulfidated protein content measured by a biotin switch assay in the iBAT was attenuated in AKO and UKO mice after cold exposure (Figure 3A,B), demonstrating that Cars2 possesses cysteine persulfide synthase (CPERS) activity to mediate persulfidation of endogenous proteins. Next, we explored the role of Cars2 in thermogenesis during cold exposure. At room temperature, core body temperatures were comparable between wild‐type (Flox) mice and AKO/UKO mice. However, after 4 h of exposure to 4°C, the core body temperatures of AKO and UKO mice were significantly lower than those of wild‐type controls (Figure S2J). The homeothermic fraction of AKO mice decreased substantially after 8 h of cold exposure, while that of UKO mice declined after 6 h (Figure 3C,D). Infrared imaging further revealed that *Cars2*‐deficient mice exhibited lower iBAT temperatures under cold exposure (Figure 3E,F). Histological analysis of iBAT showed that *Cars2* deficiency led to extensive lipid accumulation, with iBAT appearing whitened (Figure 3G,H). The morphology of other tissues showed no differences (Figure S2K,L). Electron microscopy and mitochondrial DNA quantification revealed that mitochondrial morphology and quantity remained almost unaffected (Figure 3G,H and S2M–P). Body weights of AKO and UKO mice were comparable to those of their wild‐type littermates (Figure 3I), but iBAT mass and triglyceride content was significantly higher in AKO and UKO mice, consistent with increased lipid accumulation in iBAT (Figure 3J). Additionally, plasma triglyceride and non‐esterified fatty acid levels were significantly elevated in AKO and UKO mice compared to wild‐type controls, indicating increased circulating lipids (Figure 3K,L). Compared with wild‐type control mice, AKO and UKO mice exhibited no significant differences in blood glucose levels, suggesting intact glucose homeostasis in both knockout models (Figure 3M). Then, we subjected the mice to metabolic cages and found that the oxygen consumption and energy expenditure were reduced in AKO and UKO mice compared to wild‐type controls (Figure 3N–Q and S3A,B). More importantly, *Cars2*‐AKO and ‐UKO mice exhibit significant suppression of energy expenditure induction upon CL stimulation (Figure 3R,S). No significant differences were observed in food intake, locomotor activity, or energy excretion in feces among the groups (Figure S3C–H). Also, no significant differences in lean and fat mass were detected between wild‐type and AKO mice (Figure S3I). mRNA sequencing of iBAT from AKO mice and controls revealed that *Cars2* deficiency downregulated the expression of numerous genes (Figure 3T,U). Specifically, the expression of thermogenic genes, such as *Ucp1* and *Dio2*, and brown fat enriched genes, such as *Otop1* and *Nrg4* was attenuated, while the expression of collagen genes, such as *Col1a2*, *Col5a2* and *Col3a1* was enhanced. Enrichment analysis revealed that the downregulated genes were clustered to fatty acid metabolic pathways, which are closely linked to thermogenic function, while upregulated genes were associated with extracellular matrix organization (Figure 3V,W). QPCR validation confirmed that *Cars2*‐deficient iBAT exhibited decreased mRNA expression of browning‐related genes, including *Ucp1*, *Cidea*, *Dio2*, *Prdm16*, and *Pgc1α*, and increased expression of whitening‐related genes [11], such as *Srebp1c*, *Leptin*, *Igf1* and *Enpep* (Figure 3X and S4A,B). Notably, collagen gene expression and fibrosis were enhanced in iBAT from AKO and UKO mice (Figure S4C–F). In eWAT and iWAT from AKO and UKO mice, thermogenic gene expression showed no significant differences between the groups (Figure S4G,H). Western blot analysis further showed reduced protein levels of the browning marker UCP1, as well as MTCO1 and NDUFB8, subunits of the respiratory chain complex (Figure 3Y,Z and S4I,J). To evaluate mitochondrial function in *Cars2*‐deficient mice, we measured ATP content in various fat depots. Mitochondrial ATP production was increased in AKO mice, while no difference was observed in UKO mice (Figures S4K,L), suggesting that despite reduced MTCO1 and NDUFB8 expression upon adipose *Cars2* depletion, mitochondrial homeostasis and oxidative respiration remained intact.

To evaluate the contributions of dysregulated thermogenic genes in *Cars2*‐deficient mice, we subjected *Cars2* AKO mice to thermoneutral conditions, under which iBAT thermogenic activity and thermogenic gene expression are largely attenuated. We observed that *Cars2* depletion did not result in a significant change in body weight (Figure S5A). Notably, even under these thermoneutral conditions, *Cars2* AKO mice still displayed histological whitening of iBAT, characterized by enlarged lipid droplets (Figure S5B), as well as sustained reductions in thermogenic gene expression (Figure S5C). Metabolic cage analyses demonstrated that *Cars2* deficiency did not lead to an additional reduction in energy expenditure under thermoneutral conditions. Consistently, food intake and locomotor activity were unaltered in these mice. (Figure S5D–H). This observation suggests that although thermoneutrality is unable to fully erase the disparities in thermogenic gene expression, it is adequate to abrogate the differences in energy expenditure in these animals. Additionally, plasma metabolite profiling showed that triglyceride and NEFA levels were not elevated in *Cars2* AKO mice (Figure S5I,J), suggesting that the associated lipid metabolic dysregulation arises as a consequence of disrupted energy homeostasis in these mice.

Collectively, these results indicate that Cars2 expression in iBAT is specifically required for mitochondrial thermogenic activity, which contributes to energy expenditure, likely through its ability to promote persulfidation of specific proteins.

### 2.4 Cars2 Cell‐Autonomously Induces Protein Persulfidation and Brown Adipogenesis

To explore the cell‐autonomous effect of Cars2 on mediating brown adipocyte differentiation, we established a stable BAC cell line with Cars2 overexpression. Our results showed that Cars2 overexpression in BACs led to increased H_2_S and RSSH levels following differentiation (Figures S6A,B), accompanied by an increase in the level of persulfidated proteins (Figure S6C,D). In the process of BAC differentiation, overexpression of Cars2 upregulated the expression of *Ucp1*, *Cidea* and *Pgc1α* both during differentiation and following NE stimulation (Figures S6E–J). Although overexpression of Cars2 had no significant effect on mitochondrial mass (Figure S6K), it reduced lipid droplet size (Figure S6L,M). Overexpressing Cars2 also increased uncoupled respiration, as measured by the oxygen consumption rate in mature BAC (Figure S6N). Consistently, the knockdown of Cars2 in pre‐BAC decreased the H_2_S and RSSH contents after differentiation (Figures S7A,B) and inhibited the formation of persulfidated proteins (Figure S7C,D). During the differentiation process of BAC, the suppression of Cars2 resulted in a reduction in the expression levels of *Ucp1* and *Cidea*. Similar reductions in the expression of these genes were also observed following NE treatment (Figures s7E–J). Additionally, Cars2 suppression led to a slight decrease in lipid droplet formation but did not significantly affect mitochondrial mass (Figure S7K). Moreover, the knockdown of Cars2 suppressed the oxygen consumption rate in differentiated BAC (Figure S7L). To investigate the role of Cars2 expression in mature BAC, we stably expressed an inducible Cre recombinase in immortalized primary brown adipocytes from *Cars2*‐floxed mice. Cre‐driven *Cars2* depletion is achieved upon doxycycline (Dox) treatment before and after differentiation. The depletion of *Cars2* in mature BAC also led to a decrease in the amounts of H_2_S and RSSH production and persulfidated proteins. This phenotypic change is similar to that observed following *Cars2* deletion in thermogenic fat (Figures S8A–D). More importantly, loss of *Cars2* in mature BACs exerts a profound inhibitory effect on the expression of browning genes, including *Ucp1*, *Cidea*, and *Pgc1α*, as well as on uncoupled respiration, without altering lipid deposition in these cells (Figures S8E–I). These findings indicate that Cars2 is also required for the expression of thermogenic genes in mature brown adipocytes. Overall, these results suggest that Cars2 cell‐autonomously promotes BAC differentiation and increases browning gene expression and uncoupled respiration in mature adipocytes.

### 2.5 The CPERS Activity of CARS2 Is Required to Induce the Browning Phenotype and Uncoupled Respiration of Brown Adipocytes

**FIGURE 4:**
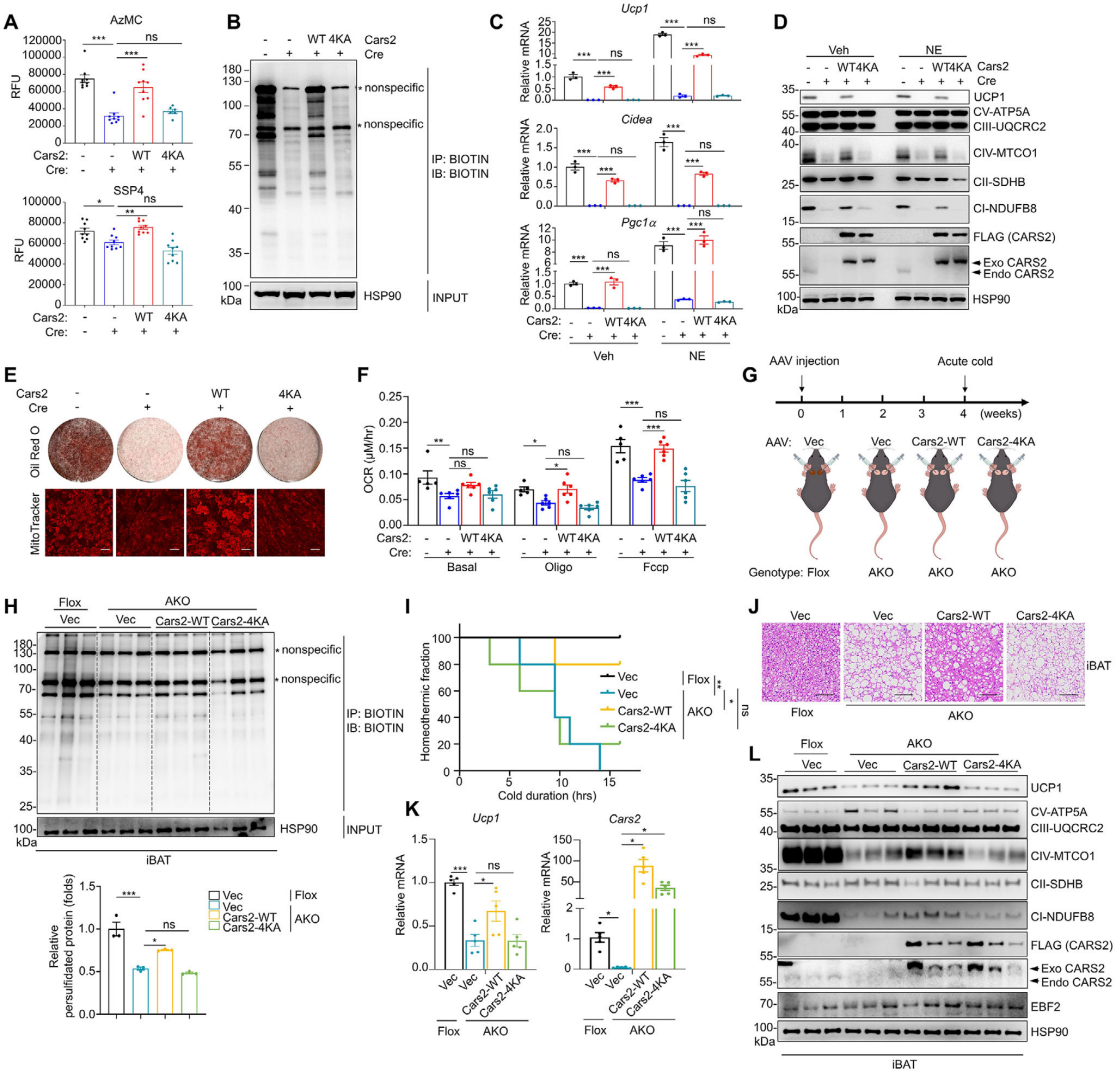
Cars2 regulates thermogenic gene expression and uncoupled respiration in BAC via its CPERS activity. (A) Hydrogen sulfide and sulfane sulfur levels were measured by AzMC and SSP4 probes in differentiated primary BACs isolated from the iBAT of newborn *Cars2* ^flox/flox^ mice expressing Vec or Cre recombinase (n = 6∼9) in combination with wild‐type or lysine‐to‐alanine (109 112 303 306) mutant (4KA) Cars2 in precursor cells as indicated. (B) The content of the persulfidated protein of differentiated BAC described in A was determined by western blot after the biotin‐HPDP switch assay. (C‐D) The mRNA and protein expression of browning genes in the differentiated BAC treated with vehicle (Veh) or norepinephrine (NE, 1 µM, 6 h) described in A was analyzed by qPCR (C, n = 3) and western blotting (D). (E) Lipid droplets and mitochondrial contents of the differentiated BAC described in A were stained with Oil red O and MitoTracker dye. Scale bar = 50 µm. (F) Oxygen consumption rates of differentiated BAC described in A were measured after vehicle (Veh), FCCP (10 µM) and oligomycin (Oligo, 10 µg/mL) treatment (n = 5∼6). (G) Diagram showing the injection strategy for AAV8 viruses expressing Cars2‐WT and Cars2‐4KA mutants or vector controls into the iBAT of control or *Cars2* AKO mice (n = 5 for each group) and the time points for acute cold exposure. (H) The content of persulfidated protein in the iBAT of mice injected with AAV as indicated in G was determined by western blotting after the biotin‐HPDP switch assay (top), and the relative amounts of persulfidated protein were determined by the density of the bands of persulfidated protein, normalized to that of HSP90. Then, the fold changes were calculated by dividing the relative persulfidated protein in the iBAT of AKO mice injected with AAV expressing Vec, Cars2‐WT and 4KA by that of control (Flox) mice (bottom). (I) Homeothermic fraction of mice (rectal temperature>30°C) injected with AAV as indicated in G in a 4°C acute cold exposure experiment. (J) H&E staining of iBAT from mice injected with AAV as indicated in G after cold exposure. Scale bar = 100 µm. (K,L) mRNA and protein expression of browning genes in the iBAT of mice injected with AAV as indicated in G was analyzed by qPCR (K, n = 5) and western blotting (L). Data are shown as the mean ± SEM. *p < 0.05, **p < 0.01, and ***p < 0.001 by one‐way ANOVA with dunnett's test (A, C, F, H and K); by Log‐rank (Mantel‐Cox) test (I); ns, nonsignificant.

CARS2 functions as the principal cysteine persulfide synthase (CPERS) in *E. coli*. Its CPERS activity is associated with two conserved motifs, ^73^KII^76^K and ^266^KMS^269^K, that are critical for binding to pyridoxal phosphate (PLP), a coenzyme of CARS2. The lysine residue (K) within these motifs are essential for CPERS activity. Single amino acid substitutions, K73A, K76A, K266A, and K269A, in CARS2 result in a near‐complete loss of its major CPERS activity. Notably, these two motifs are conserved in mammals. In mice, the corresponding motifs are ^109^KII^112^K and ^303^KMS^306^K. As such, we constructed a Cars2 mutant vector in which all four lysine residues were mutated to alanine (4KA), thereby ablating its CPERS activity. Importantly, this mutant restored the expression of MTCO1, a mitochondrial‐encoded cysteine‐containing protein, in *CARS2*‐deficient 293T cells. This finding indicates that the 4KA‐mutated Cars2 retains intact cysteinyl‐tRNA ligase activity (Figure S9A,B). Subsequently, we immortalized primary brown adipocytes derived from *Cars2*‐floxed mice and expressed Cre recombinase in the precursor cells to knockout *Cars2*. We then introduced wild‐type Cars2 or the 4KA mutant into this cell line to investigate the role of CPERS activity of Cars2 in mediating brown adipocyte differentiation. We found that knocking out *Cars2* in BAC decreased H_2_S, RSSH and persulfidated protein content, whereas reintroduction of wild‐type CARS2, but not the 4KA mutant, rescued these phenotypes (Figure 4A,B and S9C). Knockout of *Cars2* also decreases the expression of the browning‐associated genes *Ucp1*, *Cidea* and *Pgc1α* and subunits of respiratory chain complexes. Consistently, overexpression of wild‐type CARS2, but not the 4KA mutant, reversed the downregulation of these genes (Figure 4C,D and s9 D). We further observed that *Cars2* deficiency attenuated lipid droplet formation, reduced mitochondrial number, and decreased oxygen consumption rates in BACs. Again, overexpression of wild‐type CARS2, but not the 4KA mutant, restored these phenotypes (Figure 4E,F). To further confirm that the CPERS activity of CARS2 mediates iBAT thermogenic function in vivo, we directly injected adeno‐associated viruses (AAV) expressing wild‐type and 4KA‐mutant Cars2 into the iBAT of *Cars2* AKO mice (Figure 4G). Viral delivery via direct in situ injection into iBAT resulted in restricted transgene expression to iBAT, with no overexpression in eWAT or iWAT and only mild expression in the liver (Figure S9E). Wild‐type Cars2, but not the 4KA‐mutant, reversed the compromised protein persulfidation in the iBAT of *Cars2* AKO mice (Figure 4H). Notably, the expression of wild‐type CARS2 restored the cold intolerance phenotype and impaired iBAT activation in *Cars2* AKO mice, whereas the 4KA‐mutant did not (Figure 4I,J). Additionally, the expression of thermogenic genes and those related to the mitochondrial electron transport chain were rescued in the iBAT of *Cars2* AKO mice upon overexpression of wild‐type CARS2. In contrast, the expression of these genes in the group expressing 4KA‐mutant CARS2 remained unaltered compared with that in the *Cars2* AKO mice (Figure 4K,L and S9F). Interestingly, the protein expression of mitochondrial‐encoded genes, MTCO1 were not rescued by the expression of 4KA‐mutant CARS2 in the BAC, either in vitro or in vivo. The discrepancy compared to what was observed in 293T cells may be attributed to the inhibition of differentiation occurring in brown adipocytes with *Cars2* depletion that express the 4KA‐mutant CARS2. This is evidenced by the Oil Red O and MitoTracker staining (Figure 4E). PLP is the coenzyme of CARS2 that binds to two conserved motifs, KIIK and KMSK, to promote the CPERS activity of CARS2. PLP treatment upregulated the expression of *Ucp1* and *Cidea*. Importantly, *Cars2* deficiency diminished the effect of PLP on inducing the browning phenotype (Figure S9G–L). All these results suggest that the CPERS activity of CARS2 is essential for iBAT thermogenesis and BAC differentiation.

### 2.6 Cars2 Drives Brown Adipocyte Differentiation Through Persulfidating EBF2

**FIGURE 5:**
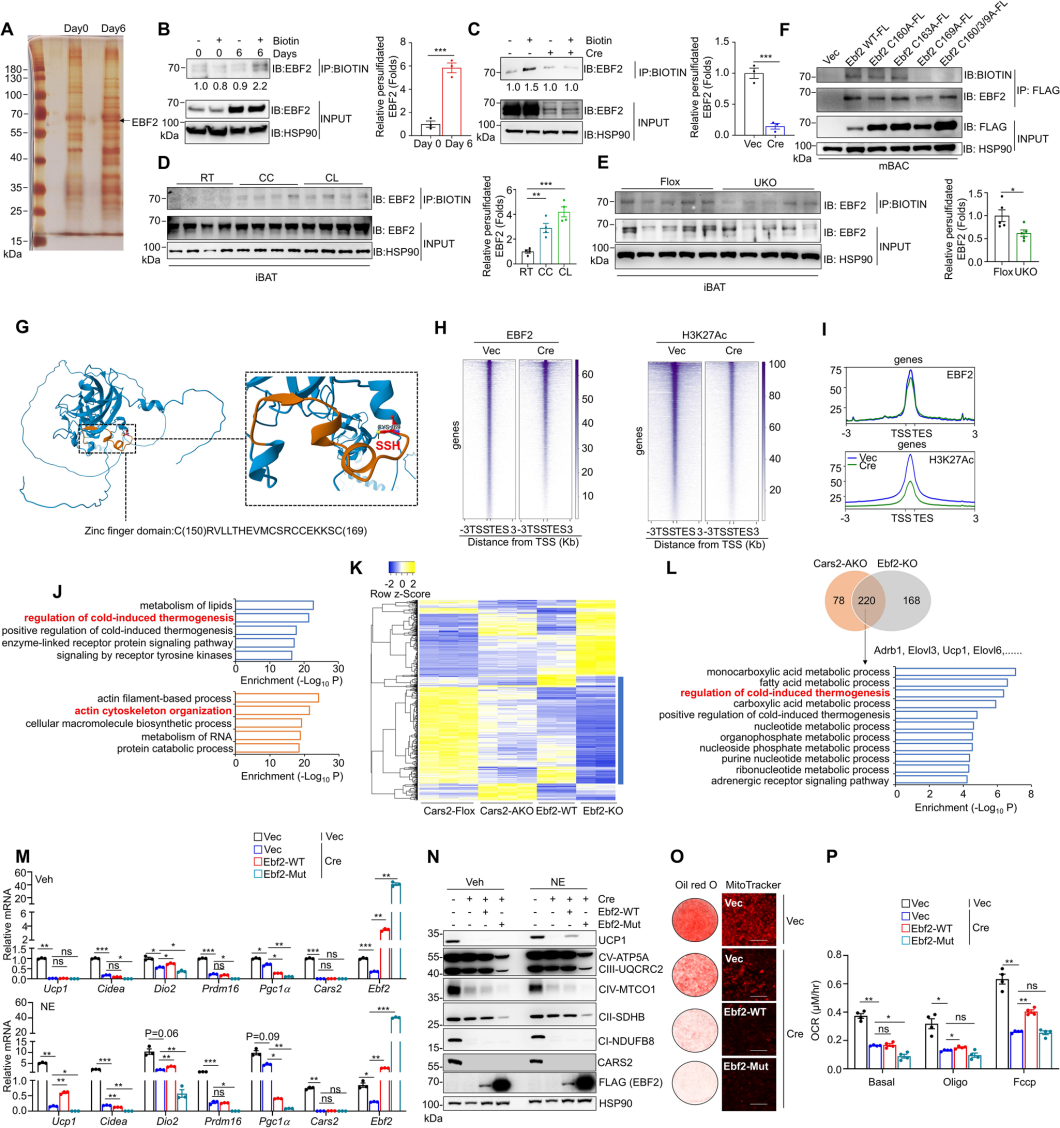
The persulfidation of EBF2 potentiates its activity and enhances brown adipocyte differentiation and uncoupled respiration. (A) Persulfidated proteins from precursor (Day 0) and mature (Day 6) mouse BAC were pulled down with streptavidin‐agarose after the biotin‐HPDP switch assay and subjected to SDS‐PAGE separation and silver staining. The indicated differential band was excised for mass spectrum identification. (B‐C) Persulfidated EBF2 in precursor (Day 0) and mature (Day 6) BAC (B) or primary BAC isolated from the iBAT of newborn *Cars2* ^flox/flox^ mice expressing Vec or Cre recombinase in precursor cells after differentiation (C) was determined via western blotting after the biotin‐HPDP switch assay. The relative density of the western blot bands was determined using image J. Relative persulfidated EBF2 in BAC at Day0 and Day 6 and primary BAC expressing Vec and Cre was calculated by the density of EBF2 (IP, Biotin+)/EBF2 (INPUT normalized with HSP90) minus EBF2 (IP, Biotin‐)/EBF2 (INPUT normalized with HSP90). Then, the fold changes were calculated by dividing the relative persulfidated EBF2 in BAC at Day 6 by that at Day 0 (B, right), or by dividing the relative persulfidated EBF2 in BAC expressing Cre by that expressing Vec (C, right). (D,E) Persulfidated EBF2 in the iBAT of mice kept at room temperature (RT, n = 4), subjected to cold acclimation (CC, n = 4) and treated with CL316243 (CL, n = 4) (D) or in the iBAT of *Cars2* control (Flox, n = 5), and Ucp1‐Cre knockout mice (UKO, n = 5) (E) was determined by western blotting after the biotin‐HPDP assay. The relative density of the western blot bands was determined using image J. Relative persulfidated EBF2 in the iBAT of mice was calculated by the density of EBF2 (IP)/EBF2 (INPUT normalized with HSP90). The fold changes were calculated by dividing the relative persulfidated EBF2 in the iBAT of chronic cold or CL‐treated mice by untreated mice (D), or by dividing the relative persulfidated EBF2 in the iBAT of *Cars2* UKO mice by Flox mice (E). (F) Wild‐type and mutant EBF2 with a Flag tag (FL) were overexpressed in BAC, and persulfidated EBF2 was purified via Flag agarose after the biotin‐HPDP switch assay and determined via western blotting after differentiation. (G) The protein structure of EBF2 was predicted via the AlphaFold program. (H,I) Heatmap (H) and profile (I) indicating the distribution of the global binding activity of EBF2 and acetylated histone (H3K27ac) in primary BAC isolated from the iBAT of newborn *Cars2* ^flox/flox^ mice expressing Vec or Cre in precursor cells after differentiation, as examined by Cut & Tag sequencing analysis. (J) Pathway enrichment analysis of genes with decreased (upper panel) and increased (lower panel) binding of EBF2 in Cre‐expressing BAC compared with Vec‐expressing BAC. (K) The gene expression signatures of iBAT from *Cars2* Adiponectin‐Cre knockout (AKO), *Ebf2*‐knockout (KO) and control mice are shown in the heatmap. (L) A Venn diagram is presented to illustrate the genes that are commonly downregulated in both *Cars2* AKO mice compared to control mice, and in *Ebf2* total KO mice (GSE97116) relative to control mice. Subsequently, these identified common downregulated genes were subjected to Gene Ontology (GO) analysis (bottom). (M,N) Wild‐type Ebf2 (Ebf2‐WT) or cysteine 169 to alanine mutant (Ebf2‐Mut) was overexpressed in primary BAC isolated from the iBAT of newborn *Cars2* ^flox/flox^ mice expressing Cre (n = 3) in precursor cells, and the expression of browning genes of matured BAC treated with vehicle (Veh) or norepinephrine (NE, 1 µM, 6 h) was analyzed by qPCR (M) and western blotting (N). (O) Lipid droplets and mitochondria of the BAC described in M‐N were stained with Oil red O and MitoTracker dye after differentiation. Scale bar = 50 µm. (P) Oxygen consumption rates of differentiated BAC described in M‐N were determined after FCCP (10 µM) and oligomycin (Oligo, 10 µg/mL) treatment (n = 4). Data are shown as the mean ± SEM. *p < 0.05, **p < 0.01, and ***p < 0.001 by unpaired Student's *t*‐test (B, C and E); by one‐way ANOVA with dunnett's test (D, M and P); ns, nonsignificant.

The above results lead us to hypothesize that a key transcription factor during BAC differentiation is persulfidated and activated by Cars2, thereby promoting a browning phenotype during differentiation. To identify increased persulfidated proteins during BAC differentiation. We labeled all persulfidated proteins in BAC with biotin‐HPDP and analyzed them using SDS‐PAGE followed by silver staining. The persulfidated protein profiling between day 0 and day 6 of differentiation exhibits an obvious difference (Figure 5A). The differential bands in the SDS‐PAGE gel before and after differentiation were subjected to mass spectrum identification. Among all the identified proteins, we noted that EBF2, a key transcription factor involved in BAC commitment and differentiation, undergoes persulfidation (Figure 5A). We subsequently precipitated persulfidated proteins via the biotin‐HPDP methods and verified that EBF2 persulfidation was increased after differentiation by western blotting (Figure 5B). Notably, *Cars2* knockout largely decreased EBF2 persulfidation (Figure 5C). Additionally, persulfidated EBF2 was increased in the iBAT of cold‐acclimated and CL‐treated mice (Figure 5D). In the iBAT of *Cars2* UKO mice, persulfidated EBF2 was significantly decreased compared with that in control mice (Figure 5E). To further determine the persulfidated epitope of EBF2, we initially mapped the persulfidated cysteine residue via mass spectrometry. We labeled all the cysteine and CysSSH residues in the EBF2 protein with N‐iodoacetyl l‐tyrosine methyl ester (TME‐IAM), an alkylating agent for stabilizing persulfides [29]. Then, the purified TME‐IAM‐labeled EBF2 protein was treated with dithiothreitol (DTT) to reduce the disulfide bonds in the CysSSH residue and release the labeled TME‐IAM. In *CARS2*‐depleted cells, the formation of CysSSH residues is reduced. Consequently, the TME‐IAM labels on these residues are retained after DTT treatment. Mass spectrometry analysis revealed an increased abundance of peptides containing TME‐IAM‐labeled cysteine residues at positions 150, 160, 163/164, 169, 254, and 556 of EBF2 in *CARS2*‐knockout 293T cells. This finding suggests that these residues are potentially persulfidated (Figure S10A). To further confirm the persulfidated cysteine residue in the EBF2 protein. We constructed a series of cysteine‐to‐alanine mutants of EBF2. Using a biotin switch assay, we measured persulfidation of WT and mutant EBF2 after transfecting them into 293T cells. Among all the EBF2 mutants, the C160A, C163A and C169A mutants presented significantly decreased persulfidated EBF2 expression (Figure S10B,C). Considering the results of both mass spectrometry and mutagenesis analyses, we speculate that the cysteine residues C160, C163 and C169 are potential persulfidated epitopes of EBF2. To further confirm the persulfidated cysteine residue of the EBF2 protein in BAC, we overexpressed wild‐type EBF2 and the C160A, C163A, and C169A mutants and detected persulfidation of EBF2 in the differentiated BAC. In line with the results in 293T cells, EBF2 persulfidation was significantly reduced in the C169A mutant in BACs, whereas it remained unchanged in the C160A and C163A mutants (Figure 5F and S10D). In addition, the AlphaFold program predicted that the C169 residue is in a zinc finger domain and is critical for the DNA‐binding activity of EBF2 (Figure 5G). These results provide compelling evidence that C169 of EBF2 is the major site that undergoes persulfidation by Cars2 in BAC.

Then, we evaluated the effect of persulfidation of EBF2 on its binding activity with Cut & Tag assay. In *Cars2*‐knockout BAC, the global binding activity of EBF2 decreased, accompanied by the attenuation of promoter regions with H3K27Ac modification (Figure 5H,I). Then, we clustered the genes with differential occupancy of EBF2, and the results showed that the genes with decreased EBF2 occupancy are involved in the thermogenic activity in *Cars2*‐deficient BAC. In contrast, EBF2 increased binding with the promoters of actin rearrangement genes (Figure 5J). To further elucidate the role of Cars2 in mediating EBF2 function, we analyzed the RNA sequencing results of iBAT from wild‐type and *Cars2* AKO mice, alongside datasets from wild‐type and *Ebf2* total knockout mice [7]. We found that the differential gene expression signature of *Cars2* AKO compared to wild‐type mice was very similar to that of *Ebf2* knockout mice compared to the wild‐type controls (Figure 5K). Among the 298 downregulated genes in the iBAT of *Cars2* AKO mice and the 388 downregulated genes in the iBAT of *Ebf2* knockout mice, 220 genes are downregulated in both groups. Pathway enrichment analysis revealed that the downregulated genes such as *Adrb1*, *Elovl3*, *Ucp1* and *Elovl6* in both groups were enriched in “regulation of cold‐induced thermogenesis” (Figure 5L and Table S3), indicating that Cars2 may regulate brown adipocyte thermogenic activity through EBF2. To validate this finding, we overexpressed wild‐type Ebf2 or the C169A mutant, which is incapable of being persulfidated, in *Cars2*‐deficient BACs. The deficiency of *Cars2* reduced the persulfidation of overexpressed wild type EBF2 (Figure S11A,B). However, overexpression of wild‐type EBF2 still partially rescued the expression of *Ucp1* and *Dio2*, particularly under NE‐treated conditions, whereas this rescuing effect was absent in cells expressing the C169A mutant (Figure 5M,N). Consistent results were also observed in OCR experiments. However, neither wild‐type nor C169A mutant EBF2 expression reversed the adipogenic defects in *Cars2*‐knockout BACs, including inhibited adipogenesis, as indicated by Oil Red O staining, reduced mitochondrial mass, revealed by MitoTracker staining, and impaired expression of mitochondrial‐encoded proteins (Figure 5N–P and S11C). Overall, these results suggest that the lack of EBF2 persulfidation decreases its transcriptional activity to induce browning gene expression and uncoupled respiration of BAC.

### 2.7 Persulfidation of EBF2 is Crucial for its Interaction With PPARγ or BRG1 and the Mediation of PPARγ Downstream Thermogenic Gene Expression

**FIGURE 6:**
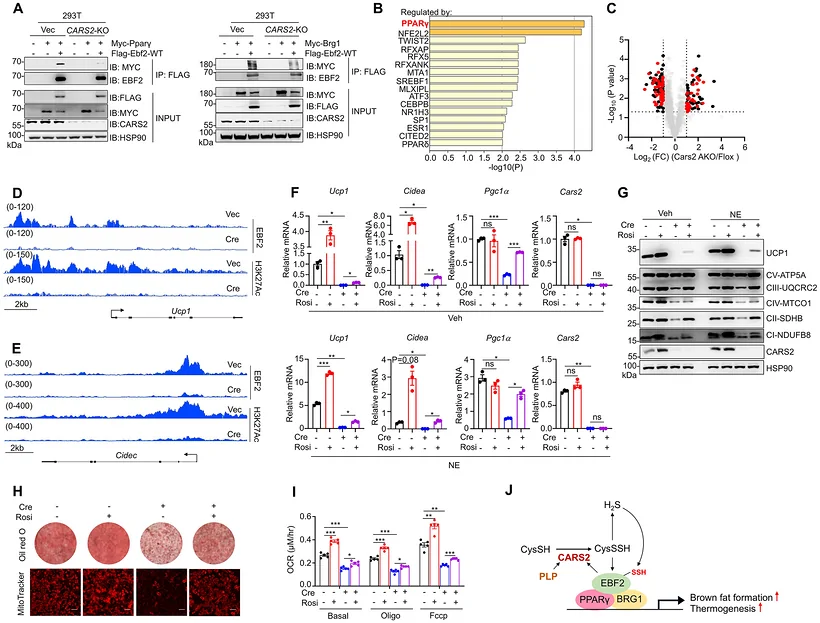
EBF2 persulfidation facilitates its interaction with PPARγ or BRG1 to induce thermogenic gene expression. (A) The interaction of PPARγ or BRG1 with EBF2 in the presence or absence of *CARS2* in 293T cells was determined via an immunoprecipitation assay. (B) Genes whose expression was downregulated in *Cars2* knockout iBAT compared with control iBAT were analyzed via transcription factor enrichment analysis. (C) RNA sequencing data of iBAT from *Cars2* Adiponectin‐Cre knockout (AKO) mice and controls were analyzed via a volcano plot, and the differentially expressed PPARγ‐predicted target genes (http://ppargene.org/) are highlighted in red. (D,E) The binding activity of EBF2 to the PPARγ target genes *Ucp1* (D) and *Cidec* (E) in primary BAC isolated from the iBAT of newborn *Cars2* ^flox/flox^ mice expressing vector (Vec) or Cre recombinase in precursor cells was examined by Cut & Tag sequencing analysis. (F‐G) Primary *Cars2*‐floxed BAC expressing vector (n = 3) or Cre recombinase (Cre, n = 3) in precursor cells were treated with rosiglitazone (Rosi, 1 µM) during differentiation (day0–day6). The mRNA and protein levels of browning genes of BAC treated with vehicle (Veh) or norepinephrine (NE, 1 µM, 6 h) after differentiation were analyzed by qPCR (F) and western blotting (G). (H) Lipid droplets and the mitochondrial contents of the differentiated BACs described in F‐G were stained with Oil red O and MitoTracker dye. Scale bar = 50 µm. (I) Oxygen consumption rates of differentiated BAC described in F‐G were measured after FCCP (10 µM) and oligomycin (Oligo, 10 µg/mL) treatment (n = 5). (J) Schematic illustration of the mechanism by which Cars2 mediates cysteine catabolism to regulate EBF2 persulfidation, thus inducing iBAT formation and thermogenesis. Data are shown as the mean ± SEM. *p < 0.05, **p < 0.01, and ***p < 0.001 by one‐way ANOVA with dunnett's test (F and I); ns, nonsignificant.

Previous studies revealed that EBF2 functionally interacts with PPARγ, a master regulator of brown adipogenesis, and recruits the BRG1/BAF complex to selectively mediate the browning gene program [7, 11]. To determine whether persulfidated EBF2 interacts with PPARγ or BRG1, we performed an immunoprecipitation assay in wild‐type and *CARS2*‐knockout 293T cells transfected with PPARγ or BRG1 together with EBF2. The persulfidation of EBF2 in *CARS2* knockout 293T cells was reduced (Figure S11D,E). We revealed that EBF2 strongly interacts with PPARγ or BRG1, as previously reported, whereas *CARS2* knockout decreased the interaction of EBF2 with PPARγ or BRG1 (Figure 6A and S11F). Consistent with the results in 293T cells, diminished interactions were also observed in mature *Cars2*‐knockout BAC, which exhibited reduced EBF2 persulfidation (Figure S11A,G,H). In addition, the EBF2 C160/163/169A mutant, which completely disrupted EBF2 persulfidation in 293T cells, also presented weaker interactions with either PPARγ or BRG1 compared to the wild‐type EBF2 (Figures S11I–L). Notably, transcriptional factor clustering analysis of downregulated genes in the iBAT of *Cars2* AKO mice revealed that PPARγ is the most highly enriched transcriptional factor, controlling the expression of the majority of these genes (Figure 6B). In addition, almost 50% of the differentially expressed genes in *Cars2* knockout iBAT were predicted to be PPARγ target genes (Figure 6C). Consistently, in differentiated BACs with *Cars2* knockout, both the binding of EBF2 to PPARγ downstream target genes such as *Ucp1* and *Cidec* and the chromatin accessibility of these genes were significantly reduced (Figure 6D,E). To further confirm that the decrease in PPARγ activity is critical for suppressing brown adipocyte differentiation in *Cars2*‐deficient BAC, we treated these *Cars2*‐deficient cells with rosiglitazone, a PPARγ agonist. The results demonstrated that treatment with the PPARγ agonist partially restored the suppressed expression of browning markers and subunits of the mitochondrial oxidative phosphorylation complex, along with improvements in lipid droplet formation, mitochondrial mass, and uncoupled respiratory function in BACs. However, it barely rescue the expression of mitochondrial‐encoded protein MTCO1 (Figure 6F–I and S11M). These results suggest that Cars2 induces EBF2 persulfidation to facilitate its interaction with PPARγ or BRG1, thereby mediating browning gene expression in BAC.

### 2.8 Cars2 Mediates EBF2 Persulfidation via H_2_S and CysSSH Signaling

**FIGURE 7:**
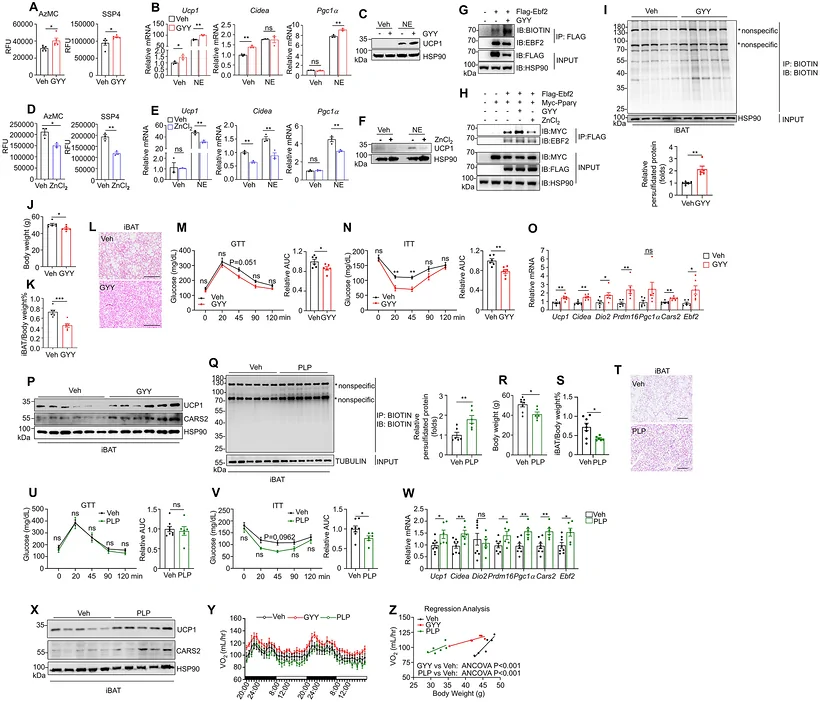
H_2_S donor and PLP supplementation promotes iBAT activity and alleviate diet‐induced obesity progression. (A–C) Mouse BAC was treated with vehicle (Veh) or GYY4137 (GYY, 100 µM) during differentiation (day0–day6). Hydrogen sulfide and sulfane sulfur levels in mature BAC (n = 5) were measured by AzMC and SSP4 probes (A). The mRNA (B, n = 3) and protein (C) levels of browning genes of BAC treated with vehicle (Veh) or norepinephrine (NE, 1 µM, 6 h) after differentiation were determined by qPCR and western blotting. (D–F) Mouse BAC was treated with vehicle (Veh) or ZnCl_2_ (100 µM) for 12 h after differentiation. Hydrogen sulfide and sulfane sulfur levels in mature BAC (n = 3) were measured by AzMC and SSP4 probes (D). The mRNA (E, n = 3) and protein (F) levels of browning genes of BAC treated with vehicle (Veh) or norepinephrine (NE, 1 µM, 6 h) after differentiation were determined by qPCR and western blotting. (G) Plasmids expressing wild‐type Ebf2 with a Flag tag was transfected into 293T cells, treated with vehicle or GYY4137 (100 µM, 48 h), then persulfidated EBF2 was purified via the biotin‐HPDP switch assay and determined via western blot analysis. (H) The interaction between overexpressed Myc‐tagged Pparγ and Flag‐tagged Ebf2 was examined in 293T cells following treatment with GYY4137 (100 µM, 48 h), ZnCl_2_ (100 µM, 48 h), or vehicle control, using an immunoprecipitation assay. (I) Wild‐type mice were intraperitoneally injected daily with GYY (120 mg/kg, n = 6) or saline (Veh, n = 6) and fed on high‐fat diet (HFD) for 4 months. The persulfidated protein content in the iBAT was determined by western blotting after the biotin‐HPDP switch assay (top), and the relative amounts of persulfidated protein were determined by the density of the bands of persulfidated protein, normalized to that of HSP90. Then, the fold changes were calculated by dividing the relative persulfidated protein in the iBAT of GYY‐treated mice by that of Veh‐treated mice (bottom). (J,K) The body weights (J) and percentages of iBAT weights (K) of the mice in I were measured after treatment. (L) Representative H&E staining of iBAT from Veh‐ and GYY‐treated mice in I after HFD feeding. Scale bar = 200 µm. (M,N) The glucose tolerance test (GTT, M) and insulin tolerance test (ITT, N) were performed in Veh‐ and GYY‐treated mice in I after HFD feeding for 12 and 13 weeks, respectively. The area under the curve (AUC) was calculated. The fold changes were calculated by dividing the AUC of GYY‐treated mice by that of Veh‐treated mice. (O,P) The browning genes in the iBAT of Veh‐ and GYY‐treated mice in I after HFD feeding were analyzed by qPCR (O) and western blotting (P). (Q) Wild‐type mice were intraperitoneally injected daily with PLP (75 mg/kg, n = 6) or saline (Veh, n = 8) and fed on HFD for 4 months. The persulfidated protein content in the iBAT was determined by western blotting after the biotin‐HPDP switch assay (left), and the relative amounts of persulfidated protein were determined by the density of the bands of persulfidated protein, normalized to that of HSP90. Then, the fold changes were calculated by dividing the relative persulfidated protein in the iBAT of PLP‐treated mice by that of Veh‐treated mice (right). (R,S) The body weights (R) and percentage of iBAT weights (S) of mice in Q were measured after treatment. (T) Representative H&E images of iBAT from Veh‐ and PLP‐treated mice in Q after HFD feeding. Scale bar = 200 µm. (U,V) GTT (U) and ITT (V) assays were performed in Veh‐ and PLP‐treated mice in Q after HFD feeding for 9 and 10 weeks, respectively. The AUC was calculated. The fold changes were calculated by dividing the AUC of PLP‐treated mice by that of Veh‐treated mice. (W,X) The browning genes in the iBAT of Veh‐ and PLP‐treated mice in Q after HFD feeding were analyzed by qPCR (W) and western blotting (X). (Y‐Z) Oxygen consumption curves (Y) and regression plots (Z) of HFD‐fed mice treated with Veh (n = 6), GYY (n = 6) or PLP (n = 6) measured in metabolic cages. Data are shown as the mean ± SEM. *p < 0.05, **p < 0.01, and ***p < 0.001 by unpaired Student's *t*‐test (A, B, D, E, I, J, K, O, Q, R, S, W, right panel of M,N and U,V); by two‐way ANOVA with multiple comparisons (left panel of M,N and U,V); by two‐sided ANCOVA analysis (Z); ns, nonsignificant.

Cars2 catalyzes cysteine catabolism by converting cysteine to CysSSH and its subsequent product H_2_S. These metabolites potentially function as signaling molecules that link mitochondrial Cars2 expression to persulfidation of the transcription factor EBF2. Our results demonstrated that CysSSH is primarily localized to the cytosol upon production (Figure S1I). According to the literature [25], cytosolic CysSSH may be incorporated into the EBF2 protein during translation, thus forming EBF2 persulfidation. Besides CysSSH, H_2_S has also been reported to mediate protein persulfidation, thereby regulating various physiological processes [30, 31, 32]. H_2_S functions as a gasotransmitter and is distributed throughout the cell (Figure S1I). Its presence in the nucleus may lead to the persulfidation of EBF2. We found that the H_2_S and CysSSH content was increased in both mouse and human BACs during differentiation (Figure 1H–K). Furthermore, we observed that *Cars2* deficiency in mouse BACs reduced cytosolic CysSSH levels and whole cell H_2_S levels, supporting the notion that CysSSH and H_2_S produced by Cars2‐mediated cysteine catabolism regulate EBF2 persulfidation (Figure S11N). To verify that H_2_S and CysSSH acts as signaling molecules regulating the browning phenotype of mature BACs by persulfidating EBF2, we treated BACs with GYY4137 (GYY), a slow‐releasing H_2_S donor, to increase intracellular H_2_S and RSSH levels, and with ZnCl_2_, an H_2_S scavenger, to decrease intracellular H_2_S and RSSH contents. Notably, GYY treatment induced the expression of BAC markers, including *Ucp1*, *Cidea*, and *Pgc1α* (Figure 7A–C and S11O). In contrast, ZnCl_2_ treatment attenuated the expression of browning‐related genes (Figure 7D–F and S11P). To directly evaluate the role of GYY in mediating EBF2 persulfidation, we treated 293T cells overexpressing EBF2 with GYY. We found that GYY treatment further enhanced the persulfidation of ectopically expressed EBF2 (Figure 7G and S11Q). More importantly, GYY treatment promotes the interaction between EBF2 and PPARγ, whereas ZnCl_2_ treatment reduces this interaction (Figure 7H and S11R). Collectively, these results suggest that H_2_S and CysSSH, metabolic products of Cars2‐driven cysteine catabolism, serve as signaling molecules that induce EBF2 persulfidation, thereby promoting its interaction with PPARγ to drive brown adipocyte differentiation.

### 2.9 Supplementation With H_2_S Donor and PLP Alleviate Diet‐Induced Obesity Progression

To further explore the function of H_2_S in vivo, we subjected the mice to high‐fat diet (HFD) feeding and performed daily treatment with GYY during this process. We found that chronic GYY treatment directly increased iBAT protein persulfidation (Figure 7I). Intriguingly, GYY treatment reduced body weight gain and suppressed lipid accumulation in the iBAT of obese mice (Figure 7J–L). Of note, Glucose tolerance test (GTT) and Insulin tolerance test (ITT) revealed that GYY treatment enhances insulin sensitivity (Figure 7M,N and S12A,B). QPCR analysis revealed that the expression of a set of thermogenic genes was induced in the iBAT of GYY‐treated mice (Figure 7O), and this induction was further verified by western blot analysis (Figure 7P and S12C). Notably, the expression of Cars2 was also induced in iBAT after GYY treatment (Figure 7O,P and S12C), indicating that H_2_S accumulation in iBAT may promote Cars2 expression to form a feedforward loop to induce the browning phenotype.

PLP, the active form of vitamin B6, is a coenzyme of CARS2. Supplementation with PLP may alleviate diet‐induced obesity progression by increasing CARS2 activity. To test this hypothesis, we administered PLP daily to a group of HFD‐fed mice. PLP treatment significantly induced iBAT persulfidation (Figure 7Q), which is probably through activating CARS2. More importantly, PLP‐treated mice exhibited a significant reduction in body weight gain and a decrease in iBAT weight (Figure 7R,S). Importantly, PLP treatment reduced lipid content in the iBAT of obese mice and induced thermogenic gene expression. Consequently, insulin resistance in these obese mice was alleviated (Figure 7T–X and Figure S12D–F). Interestingly, PLP treatment also increased Cars2 expression (Figure 7W,X and S12F), suggesting that elevated CARS2 activity may upregulate its own expression via feedforward signaling to amplify PLP‐induced brown fat activation.

To investigate the effects of GYY and PLP treatment on energy consumption during HFD feeding. The GYY‐ and PLP‐treated obese mice were subjected to a metabolic cage experiment. Compared to those in saline‐treated mice, the VO_2_ level and energy expenditure of GYY‐ and PLP‐treated mice were significantly higher (Figure 7Y,Z and S13A). The food intake and activity of the mice were not influenced (Figure S13B,C). The body composition analysis indicates the fat mass in the GYY‐ and PLP‐treated mice was reduced while the lean mass of these mice was unchanged, probably due to the activation of iBAT in these mice (Figure s13 D).

In conclusion, our study revealed that Cars2 expression is induced by EBF2 during brown adipocyte differentiation, thereby mediating cysteine catabolism to produce CysSSH and H_2_S. These metabolites act as signaling molecules to enhance EBF2 activity by persulfidating EBF2 and facilitating its interaction with PPARγ or BRG1, which in turn drives the browning phenotype and thermogenic activity. Activating CARS2 via treatment with its coenzyme PLP, or increasing H_2_S levels using GYY, induces brown fat formation and alleviate diet‐induced obesity progression. This implies that dietary intake of vitamin B6 may exert a protective effect against obesity development (Figure 6J).

## 3 Discussion

Thermogenic fat is a class of special adipose tissue that frequently consumes energy through uncoupled respiration. The activation of thermogenic fat has become an attractive strategy for weight loss. However, how to stimulate thermogenesis safely is still challenging. The major obstacle of using β3 adrenoreceptor agonists to activate thermogenic fat is their side effects on targeting other β adrenoreceptors expressed in cardiomyocytes [33]. Although brown and beige fat are derived from different progenitors, their differentiation mechanisms are similar. Both of these processes are mediated by the transcriptional factors EBF2, PRDM16 and PPARγ, which constitute a transcriptional complex. This regulatory machinery has been recognized as a promising therapeutic target to increase thermogenic fat formation. Recently, studies have implied that the reprogramming of metabolic homeostasis in the cell determines cell identity and cell fate by metabolic sensing mechanisms [34]. Along with this notion, we hypothesize that the altered metabolite content during brown/beige adipocyte differentiation is sufficient to drive thermogenic adipose tissue development and functions.

In this study, we focused on exploring intracellular metabolic homeostasis reprogramming in brown adipocyte differentiation and demonstrated that cysteine catabolism is an exclusive cell‐intrinsic metabolic pathway enriched in the browning process. Among all the derivatives from cysteine, H_2_S and CysSSH production is largely unknown for their functions in brown adipocyte differentiation. Although the synthesis of H_2_S is catalyzed mainly by CBS and CSE in tissues such as the liver, but in iBAT, H_2_S production is largely mediated by Cars2. Cars2 belongs to the aminoacyl‐tRNA synthetase (AARS) family as a traditional mitochondrial‐located cysteinyl‐tRNA ligase, which mediates the translation of mitochondrial‐encoded proteins. Recently, studies identified Cars2 as a cysteine persulfide synthase that mediates protein persulfidation. In the BAC, we verified that Cars2 is transcriptionally upregulated by EBF2 during brown adipogenesis and iBAT development upon cold and CL treatment. The expression of Cars2 is essential for mediating the formation of protein persulfidation, whereas H_2_S production likely occurs via the release from persulfidated cysteine. Using two knockout mouse models to ablate *Cars2* in thermogenic adipocytes, we demonstrated that *Cars2* deficiency resulted in iBAT dysfunction characterized by impaired thermogenesis, lipid accumulation, and fibrosis in iBAT. This was accompanied by a significant downregulation of thermogenic gene expression and a reduction in energy expenditure in mice. However, *Cars2* deficiency causes very little damage to mitochondrial morphology, number and size, although the expression of the respiration chain complex components MTCO1 and NDUFB8 is decreased. ATP production is subtly induced in the iBAT of *Cars2* AKO mice, which is consistent with the loss of uncoupled respiration in these mice. These results suggest that defects in uncoupled mitochondrial respiration are the primary contributors to the impaired browning phenotype in *Cars2* deficient mice. Interestingly, *Cars2* depletion induces hyperlipidemia, characterized by elevated plasma triglyceride and free fatty acid levels, whereas glucose homeostasis is minimally affected. These phenotypic changes are abrogated under thermoneutral conditions, with no differences in energy expenditure observed between *Cars2* AKO mice and controls, supporting that such lipid metabolic dysregulation is a consequence of disrupted energy homeostasis in *Cars2* AKO mice. Then, we illustrated that Cars2 expression cell‐autonomously regulates protein persulfidation and the browning phenotype during differentiation and in mature adipocytes. The dysfunctional browning activity in the *Cars2*‐deficient BAC is restored by WT but not the CPERS activity null mutant CARS2 (4KA) both in vitro and in vivo, suggesting that the CPERS enzyme activity of CARS2 mediates brown adipocyte differentiation. To support this hypothesis, PLP treatment improved the browning feature of BAC, which was dependent on Cars2 expression. Since Cars2 regulates protein persulfidation, we next focused on identifying a key persulfidated factor that governs brown adipocyte differentiation. We revealed that EBF2 is persulfidated by CARS2 generated cysteine derivative CysSSH and H_2_S. We identified C169 residue of EBF2 is persulfidated and is critical for the formation of a zinc finger‐like loop that determines EBF2 transcriptional activity. The global profiling of acetylated H3K27 around the promoter region displayed a drastic decrease after *Cars2* knockout, indicating that gene expression was globally downregulated. More importantly, the binding preference of EBF2 undergoes a switch from thermogenic genes to cytoskeletal genes in *Cars2*‐knockout BAC. This change may be due to the disruption of the EBF2 and PPARγ interaction and chromatin remodeling. The overexpression of WT EBF2 in *Cars2* knockout cells decreased its activity in inducing thermogenic genes. However, it still partially restored the browning phenotype. In contrast, the persulfidation‐site mutant EBF2 completely abolished its ability to mediate thermogenic function. Meanwhile, treatment with Rosi, which directly enhances PPARγ activity, exerts a more profound effect on the induction of thermogenic genes in *Cars2* deficient cells, yet it also only partially rescues uncoupled respiration in BACs. These results suggest that Cars2‐mediated EBF2 persulfidation may not solely contribute to Cars2‐dependent iBAT thermogenesis. Other mechanisms underlying the impairment of iBAT thermogenesis in *Cars2*‐deficient mice require further investigation.

To elucidate how Cars2‐mediated cysteine catabolism in mitochondria transmits signals to the nucleus to induce EBF2 persulfidation, we visualized two Cars2‐dependent cysteine metabolites, H_2_S and CysSSH, using their fluorescent probes. We found that CysSSH mainly localizes to the cytosol, likely incorporated into EBF2 by translation, whereas H_2_S localizes to the nucleus to directly induce EBF2 persulfidation. More importantly, this subcellular distribution of H_2_S and CysSSH is mediated by Cars2. Further evidence showed that H_2_S donor GYY‐induced EBF2 persulfidation enhances its interaction with PPARγ and upregulates thermogenic gene expression in BACs. Furthermore, in an in vivo setting, we demonstrated that direct treatment with GYY increases global protein persulfidation and improves the browning phenotype. Consequently, GYY treatment alleviates body weight gain and insulin resistance that develop during obesity progression. In addition, supplementation with CARS2 coenzyme PLP also increases protein persulfidation in iBAT, resulting in the activation of iBAT in obese mice and a reduction in body weight. Since both sulfur species and PLP are abundant in dietary food, our results suggest that the dietary intake of sulfur species and PLP‐rich food may serve as an important strategy for losing weight by targeting CARS2 to increase EBF2 persulfidation.

Collectively, these findings position Cars2‐mediated intracellular cysteine catabolism, which promotes EBF2 persulfidation and facilitates its binding to PPARγ and BRG1 to drive iBAT development, as a core cell‐intrinsic regulatory mechanism governing iBAT thermogenesis under physiological conditions. A recent study by Dixit and colleagues focused on dietary interventions for weight loss, and demonstrated that dietary cysteine deficiency drives thermogenic fat activation and reduces body weight [35]. Their study revealed that systemic nutrient sensing of cysteine availability modulates endogenous cysteine metabolic pathways, concomitant with transcriptional upregulation of thermogenic genes in adipose tissue. In line with our findings, both studies establish that cysteine metabolism governs the transcriptional regulation of thermogenic genes in adipose tissue. Importantly, our work is directly relevant to physiological conditions and further delineates the cell‐intrinsic molecular pathways through which cysteine metabolism governs the transcriptional program underlying brown adipocyte differentiation and thermogenic activation. Furthermore, we identify Cars2 as a novel molecular target for modulating iBAT activity during obesity progression, thereby establishing a new metabolite‐directed therapeutic strategy for obesity intervention.

## 4 Methods

### 4.1 Animal Studies

Our study examined male mice in chow‐ and HFD‐feeding experiments because male animals exhibited less variability in phenotype, and female mice were resistant to HFD‐induced obesity. All animal experiments were approved by the Institutional Animal Care and Use Committee (IACUC) of Shanghai Jiao Tong University School of Medicine and performed consistently with the IACUC's rules (Approval number: JUMC2023‐108‐A). The mice were maintained under a 12/12 h light/dark cycle and fed regular rodent chow. Mice aged between 8 and 28 weeks were used in the experiments. *Cars2* ^flox/flox^ mice were purchased from the Shanghai Model Organisms Center and further crossed with Adiponectin‐Cre (Jackson Laboratory, #028020) and Ucp1‐Cre mice (Jackson Laboratory, #024670) to generate adipose tissue‐specific *Cars2* knockout and thermogenic adipose tissue‐specific *Cars2* knockout mice, respectively. All the mouse experiments were independently replicated at least twice, and we did not exclude any data points or mice unless a technical issue or human error occurred. The sample size was determined based on two key considerations. First, previous relevant publications that reported sufficient statistical power with similar cohort sizes; second, our own preliminary experimental data, which provided supportive evidence for the chosen sample sizes. Additionally, we selected a cohort of mice strictly matched for gender, age, and date of birth to minimize inter‐individual variability, and all mice meeting these inclusion criteria were included in the final analysis. For brown adipose activation, CL316243 (Sigma, C5976) was intraperitoneally injected for 7 days at dose of 1 mg/kg. In the HFD‐induced obese mouse model, PLP (Sigma, P3657), GYY4137 (Dojindo, SB06) were intraperitoneally administered for 60 days at doses of 75 and 120 mg/kg, respectively. The tissues were dissected and immediately frozen in liquid nitrogen before RNA and protein extraction. For histology, the tissues were fixed with 4% formalin overnight at 4°C and subjected to paraffin embedding and H&E staining. Sirius red (Abcam, ab246832) staining was performed as previously described [36].

### 4.2 Acute Cold Exposure Experiment and Infrared Imaging

Mice were maintained at room temperature (∼25°C) before cold exposure. During cold exposure, the mice were maintained in a temperature‐controlled chamber at 4°C with free access to food and water. For the cysteine supplementation assay, mice were fasted for 16 h prior to cold exposure and remained fasted throughout the entire cold exposure period. Rectal temperature was measured with a rectal thermometer at the indicated time points during cold exposure. The infrared image was captured by an infrared thermal camera. We set a rectal temperature lower than 30°C as the cutoff to calculate the homeothermic fraction. Plasma triglyceride (Sigma, TR0100) and free fatty acid (WAKO, 294–63601) levels were determined via commercial ELISA kits.

### 4.3 Metabolic Phenotype Analyses

Indirect calorimetry was performed by using the Comprehensive Laboratory Animal Monitoring System (CLAMS, Columbus Instruments) at room temperature (∼25°C) or thermoneutral temperature (∼30°C). The mice were individually acclimated to metabolic chambers for 24 h and monitored for an additional 48 h on a 12 h light‒dark cycle and had free access to chow or high‐fat food and water. Oxygen consumption (VO_2_) and carbon dioxide production (VCO_2_) were recorded at 10 min intervals. The oxygen consumption and heat generation were adjusted to the body weight of each mouse. Locomotor activity and food intake were monitored during the indirect calorimetry experiments using built‐in sensors located in each cage. A regression‐based analysis of the relationship between absolute oxygen consumption (VO_2_) and energy expenditure values, with respect to body weight was analyzed at https://calrapp.org [37, 38]. For thermoneutral temperature metabolic cage experiments, mouse were pre‐housed at 30°C for 4 weeks, then placed them in metabolic cages at 30°C to collect data. To assess CL316,243‐induced energy expenditure, mice were anesthetized with pentobarbital (90 mg/kg, intraperitoneally) and acclimated to the experimental environment for 60 min to allow oxygen consumption to stabilize. The mice were then injected with CL316,243 (1 mg/kg), and data were collected over a 2‐h period in animals housed at 20°C, as previously described [39]. For the GTT, the mice were fasted for 16 h and received an intraperitoneal (i.p.) injection of D‐glucose (1 g/kg body weight) (Sigma, G5767). For ITT, the mice were injected intraperitoneally with human insulin (Novo Nordisk; 1 U/kg body weight) after 4 h of fasting. For both the GTT and ITT, tail vein blood glucose was measured at time points 0 min, 20 min, 45 min, 90 and 120 min after treated with glucose or insulin using a glucometer monitor (Sinocare). The area under the curve (AUC) of GTT and ITT was generated via GraphPad Prism.

### 4.4 Administration of Adeno‐Associated Virus

AAV administration was performed as described previously [40]. Wild‐type mouse Cars2 and the 4KA mutant coding sequence were cloned into the pAAV‐CAG‐MCS vector. The AAV expressing vector, Cars2‐WT (pCAG‐Cars2‐WT) and the 4KA mutant (pCAG‐Cars2‐4KA) were produced by cotransfected empty vector or CAG‐Cars2‐WT/4KA with the DF6 and AAV8 serotype plasmids into 293T cells. AAVs were purified via density gradient centrifugation. To overexpress Cars2 in the iBAT of *Cars2*‐AKO mice, AAV was injected into interscapular brown adipose tissue (iBAT) pads on both sides. Briefly, the mice were anesthetized with isoflurane, and the hair around the scapular area was shaved. A horizontal incision was made on the skin around the interscapular areas, followed by exposure of the fat pad via tweezers. A total of 50 µL of AAV liquid containing 5 × 10^11^ viral genomes (vg) was injected into each fat pad at multiple spots (5–8 spots per fat pad). The skin was subsequently closed with a surgical staple. 4 weeks after AAV injection, the mice were subjected to acute cold exposure.

### 4.5 Cell Culture and Differentiation

HEK293T (RRID:CVCL_0045) and 3T3‐L1 cells (RRID:CVCL_0063) were obtained from American Type Culture Collection (ATCC). Mouse brown preadipocytes (BAC) were separated from the iBAT of newborn wild‐type or *Cars2* ^flox/flox^ mice, and the cells were immortalized by expressing the large T antigen of the SV40 virus as previously reported [41]. BAC's RRID is not available since this is an immortalized cell line derived from primary cells. This cell line has been utilized in our previous study as a reliable tool for assessing brown adipocyte differentiation [10]. Briefly, the coding sequence of the large T antigen was constructed into the pMSCV‐plus‐hygromycin vector (a retrovirus transfer plasmid), and was co‐transfected with packaging plasmids (Gag‐pol and VSVG) into 293T cells to acquire the pseudoretrovirus. Primary mouse BAC were infected with the retrovirus to acquire the large T antigen, and hygromycin was used to select the positive cells. Human primary brown‐liked preadipocytes and white preadipocytes were isolated from fat tissue obtained from the area near the supraclavicular region in the neck [42] or from the subcutaneous region, which was obtained from Shanghai Ninth People's Hospital. The procedure was approved by the ethics committee of Shanghai Ninth People's Hospital. All cell lines used in this study were regularly tested and confirmed to be free of mycoplasma contamination. HEK293T cells, mouse brown preadipocytes, 3T3‐L1 cells and human brown‐like and white preadipocytes were maintained in DMEM supplemented with 10% fetal bovine serum (Sigma, F8318). Confluent brown preadipocytes, 3T3‐L1 preadipocytes or human preadipocytes were subjected to differentiation by adding induction medium containing 0.5 mM IBMX (ACROS, 228420050), 125 µM indomethacin (Sigma, I‐7378), 1 µM dexamethasone (Sigma, D2915), 20 nM insulin (Sigma, I5500) and 1 nM T3 (Sigma, T2877). Cells were switched to differentiation medium (DMEM, 10% FBS, 20 nM insulin and 1 nM T3) after 2 days. The differentiation medium was changed every 2 days until mature adipocytes formed. The cells were treated with vehicle or 1 µM norepinephrine (Sigma, A9512) for 6 h to activate the expression of thermogenic genes before being collected. For the rosiglitazone or PAG treatment, 1 µM rosiglitazone (MCE, HY‐17386) or 500 µM DL‐Propargylglycine (Sigma, P7888) was added to the culture medium throughout the differentiation process. The cell culture experiments were performed in triplicate and repeated at least three times.

### 4.6 Plasmids

To overexpress mouse Cars2, Ebf2 and their mutants in BAC, the coding sequences of *Cars2* (Cars2‐WT and 4KA （K109A, K112A and K303A and K306A）) and Ebf2 (Ebf2‐WT, C160A, C163A, C169A, C160/163/169A) with a Flag‐tag were cloned into pMSCV‐plus‐puro retroviral overexpression plasmids. For Cars2 knockdown in BAC, the hairpin sequence targeting Cars2 was cloned into pSuper‐puro plasmids. For overexpression in 293T cells, the coding sequences of mouse PPARγ and BRG1 with a Myc tag and the coding sequences of mouse Ebf2 and its mutants with a Flag tag were cloned into the pCDNA3 plasmid. For the luciferase assay, the mouse *Cars2* promoter sequence (−2000 bp to +115 bp) was cloned into the pGL3‐basic plasmid to construct a *Cars2* promoter‐containing luciferase (Cars2‐luc) vector. The sequence tcctcaagagatg (−844 to −832 bp) was predicted as a potential Ebf2 binding site. Therefore, we constructed a luciferase plasmid that contained a *Cars2* promoter with a deleted Ebf2 binding site (Cars2‐luc ΔEbf2 BS). For the screen of transcriptional factors that regulating Cars2 expression, the coding sequences of mouse Prdm16, PPARα, PPARγ, Cebpα and Cebpβ with a Flag tag were cloned into pMSCV‐plus‐puro retroviral overexpression plasmid. For *Ebf2*‐KO in BAC and *CARS2*‐KO in 293T cells, small guide RNA (sgRNA) sequences targeting mouse Ebf2 and human CARS2 were cloned into the LentiCRISPRv1 vector. The sequences of the sh‐RNAs and sgRNAs are listed in Table S2.

### 4.7 Stable and Inducible Knockout of Cars2 in BAC

BAC harboring *Cars2* ^flox/flox^ were separated from the iBAT of newborn *Cars2* ^flox/flox^ mice and immortalized with the large T antigen of SV40. For stable knockout of *Cars2*, Cre recombinase was cloned into pMSCV‐plus‐hygro plasmids (pMSCV‐Cre‐hygr), which were co‐transfected with packaging plasmids (Gag‐pol and VSVG) into 293T cells to acquire the pseudo retrovirus (pMSCV‐Cre‐hygr), and the empty vector was used as control. Pre‐BAC were infected with pMSCV‐plus‐hygr or pMSCV‐Cre‐hygr retrovirus and selected with hygromycin (InvivoGen, ant‐hg‐1) until the control cells were killed. For inducible knockout of *Cars2* in BAC, the coding sequence of Cre recombinase was cloned into the pInducer3.0 lentiviral plasmid (pInducer‐Cre). The pInducer‐Cre lentivirus was packaged by co‐transfection with packaging plasmids (Tet, Rev, Gag‐pol, Vsvg) into 293T cells, and the lentivirus was collected 48 h later. BAC‐Incre cells were acquired via infection with pInducer‐cre lentivirus encoding Cre recombinase and then were selected with G418 (Basal Media, S150J7) until the uninfected control cells were completely killed. Cre expression was induced by treating BAC‐Incre cells with 100 ng/mL doxycycline (Selleck, S4163), thus to inducible knockout *Cars2* before or after differentiation.

### 4.8 Cystine Deprivation Assay

For the cystine deprivation assay, control DMEM (Gibco, 11960069) and methionine‐ and cystine‐deficient medium (Gibco, 21013024) supplemented with 30 mg/L methionine (MedChemExpress, HY‐N0326) were used to treat the BAC for 24 h after differentiation (days 5–6).The cell viability was determined by CCK‐8 assay (Selleck, B34302), and the cysteine level was measured by using commercialized Cysteine Assay kit (Biovision, K558‐100).

### 4.9 MitoTracker, Intracellular H_2_S and RSSH Measurements

The mitochondrial contents of the cells were stained with MitoTracker probe (Invitrogen, MP07510). The level of intracellular H_2_S was determined by 7‐azido‐4‐methylcoumarin (AzMC), a fluorogenic probe used for the detection of hydrogen sulfide (Sigma, 802409). The intracellular RSSH was determined via SSP4 (Dojindo, SB10), a sulfane sulfur probe. Briefly, the cells were washed once with serum‐free DMEM and incubated with MitoTracker probes (250 nM) or AzMC (50 µM) in serum‐free DMEM or incubated with SSP4 (20 µM) and 0.003% cremophor EL (Aladdin Biochem, C107105) to load SSP4 into living cells in serum‐free DMEM for 30 min at 37°C. After being washed three times with serum‐free medium, the cells were scraped, resuspended and transferred to a 96‐well plate, and the fluorescence intensity was measured via a microplate reader (Molecular Devices, ID5). After AzMC, SSP4 and Mitotracker staining, the nucleus was staining by incubating with serum‐free medium supplemented with 0.2 µM SYTO9 (MKBio, MX4229) for AzMC or 2 µg/mL Hoechst 33342 (Beyotime, C1022) for MitoTracker and SSP4 at 37°C for 10 min. Then, the cells were washed with serum‐free DMEM three times, and fluorescence images were obtained via confocal microscopy (Leica, TCS Sp8).

### 4.10 Gene Expression Analyses

Total RNA was extracted from cells and tissues using the TRIzol method following the manufacturer's instructions. For RT‐qPCR, 1 µg of RNA was reverse transcribed via HiScript II Q RT SuperMix (Vazyme, R222‐01), followed by qPCR via SYBR Green (Bimake, B21203). Relative mRNA expression was normalized to the expression of AP0. The primers used for gene expression are listed in Table S1.

### 4.11 Coimmunoprecipitation (CoIP) and Western Blot Analysis

Cells or tissues were lysed with RIPA buffer (150 mM NaCl, 1% NP40, 50 mM Tris‐HCl (pH 8.0), 25 mM NaF, 2 mM Na_3_VO_4_, 1 mM PMSF, and protease inhibitor cocktail) [43]. The lysate was sonicated and then centrifuged to remove insoluble cellular debris. IP and Western blotting were performed as described previously [44]. Briefly, 2 µg of Flag antibody (Sigma, F1804) was used for 500 µg of total whole‐cell lysate for each IP. For Western blotting, whole‐cell lysates containing 30 µg of whole protein were mixed with 2× SDS‐PAGE reduced loading buffer and boiled at 95°C for 5 min per gel well. Protein samples were separated by SDS‐PAGE and transferred onto nitrocellulose membranes for Western blotting. Nonspecific binding to the antibodies was blocked with 5% nonfat milk at room temperature for 1 h. Primary antibodies against UCP1 (Alpha Diagnostic, UCP11‐A), CARS2 (Invitrogen, PA5‐59722), HSP90 (Proteintech, 13171‐1‐AP), TUBULIN (Proteintech, 11224‐1‐AP), OXPHOS (Abcam, ab110413), EBF2 (R&D, AF7006), BIOTIN (Santa Cruz, sc‐101339), MYC tag (Proteintech, 16286‐1‐AP), FLAG (MBL, M185‐3L) and MTCO1 (CST, 62101) were incubated at 4°C overnight while rotating. To remove the residual primary antibody, the membranes were washed five times in TBS with 0.05% Tween‐20 (TBST) while agitating for 5 min per wash. The secondary antibody conjugated with HRP was incubated at room temperature for 1 h and washed five times in TBST while agitating. An ECL kit (Sharebio) was used for detection.

### 4.12 Luciferase Reporter Assay

The cells were grown to 70% confluence in a 24‐well plate and then co‐transfected with reporter gene constructs (Cars2‐luc and Cars2‐luc ΔEbf2 BS) and vector control or EBF2. All transfections were performed with Lipofectamine 2000 (Invitrogen, 11668019) according to the manufacturer's instructions. Cell extracts were prepared and mixed with luciferase substrate (Promega, E1500) 48 h after transfection, and luciferase activity was measured via a microplate reader system (Molecular Devices, ID5). All the experiments were performed three times in triplicate.

### 4.13 Oxygen Consumption Rates of Cells

The cell oxygen consumption rate (OCR) was measured via an oxygen meter with a Mitocell (Strathkelvin Instruments, MT200) mixing chamber. The cells were scraped off with a cell scraper and suspended in 400 µL of cell culture medium to measure the basal OCR of the cells. Oligomycin A (Selleck, S1478) and FCCP (MCE, HY‐100410) were added to measure the uncoupled and maximum OCRs, respectively. The OCR was calculated via software (782 Oxygen System version 4.0) and normalized to relative total protein content.

### 4.14 Biotin Switch Assay

A modified biotin switch assay was carried out according to procedures reported previously [45]. First, differentiated mature brown cell pellets or mouse adipose tissue were homogenized in 1 mL HEN buffer (250 mM HEPES‐NaOH pH 7.7, 1 mM EDTA, 0.1 mM neocuproine (Sigma, N1501), 150 µM DFO (MCE, HY‐B0988), 0.1% sodium deoxycholate (Sangon Biotech, A600150), 1% NP‐40 (Sangon Biotech, A600385), 1 mM PMSF (Beyotime, ST505) and protease inhibitor cocktail (Bimake, B14002) and then sonicated. The lysates were centrifuged at 12 000 rpm for 10 min at 4°C to remove the lipid layer, and the protein concentration was determined by Bradford assay. Lysates containing 2 mg of total protein were added to 1 mL of HEN buffer supplemented with 2.5% SDS and 20 mM methyl thanethiosulfonate (MMTS) (Sigma, 208795), followed by frequent shaking at 50°C for 30 min at 110 rpm/min. Then, the proteins were precipitated with 2.5 mL of precooled acetone for 15 min at −20°C and centrifuged at 3100 rpm for 5 min to pellet the protein. The protein pellet was washed twice with 70% acetone to remove unbound MMTS. The pellets were resuspended in HENS buffer (HEN buffer containing 1% SDS) supplemented with 0.8 mM biotin‐HPDP (APExBio, A8008), vortexed well and incubated with rotation in the dark for 2 h at room temperature. Next, the proteins were precipitated with 2.5 mL of precooled acetone for 15 min at −20°C, centrifuged at 3100 rpm for 5 min and washed twice with 70% acetone again. The pellets were subsequently resuspended in 250 µL of HENS buffer. Then, the biotinylated proteins were subjected to western blot analysis directly or pulled down with streptavidin agarose. For the streptavidin agarose precipitation assay, 250 µL of HENS buffer was added to 750 µL of neutralization buffer (20 mM HEPES‐NaOH pH 7.7, 100 mM NaCl, 1 mM EDTA, 0.5% Triton X‐100), which was subsequently incubated with streptavidin agarose (Thermo Fisher, 20347) or Flag‐antibody‐conjugated agarose (Sigma, A2220) at 4°C with rotation for 3 h. Finally, the protein‐binding agarose was washed six times with neutralization buffer containing 600 mM NaCl. Then, the agarose was resuspended in 1× loading buffer without DTT and boiled at 95°C for 5 min. The supernatants were transferred into a new EP tube and subjected to western blot analysis with antibodies against biotin or EBF2.

### 4.15 Oil Red O Staining

After brown adipocyte differentiation, the cells were fixed with 2% formalin for approximately 30 min at room temperature. Then, the cells were washed with double‐distilled water three times and subsequently stained with Oil Red O (Sigma, O0625) solution for 2 h at room temperature. Next, the cells were washed with double‐distilled water three times and poured off to air dry. Images were acquired with a scanner.

### 4.16 Cut & Tag Assay

Cut & Tag assay was performed using a commercialized kit (Vazyme, TD903) according to the manufacturer's instructions. For each sample, pre‐washed 5 × 10^4^ differentiated wild type or *Cars2*‐KO mouse BAC in 100 µL wash buffer were incubated with10 µL pre‐activated ConA beads for 10 min at room temperature (RT) with gentle mixing. The supernatant was removed, and bead‐bound cells were incubated with primary antibody (8 µg/mL for EBF2 antibody (R&D, AF7006) and 1:25 dilution for H3K27ac antibody (CST,4353) in 50 µL antibody buffer at 4°C overnight. Then, bead bound cells were incubated with 50 µL of unconjugated secondary antibody (1:100 diluted in Dig wash buffer) for 1 h at RT with rotation. After 3 washes with 200 µL Dig wash buffer, bead‐bound cells was incubated with pA/G Tnp (0.04 µM in Dig 300 buffer) for 1 h at RT with rotation. After 3 washes with 200 µL Dig‐300 buffer, bead‐bound cells were incubated with 40 µL Dig‐300 Buffer and 10 µL TTBL for 1 h at 37°C to allow DNA fragmentation; 5 µL of Proteinase K, 100 µL of Buffer L/B and 20 µL of DNA Extract Beads were added to the samples, vortexed well and incubated for 10 min at 55°C with 2–3 gentle mixing steps to stop fragmentation and DNA extraction. Then, the beads were washed once with 200 µL WA buffer and twice with 200 µL WB buffer. The fragmented DNA was eluted in 22 µL DNase‐free water; 15 µL DNA was used for amplify the libraries according to the manufacture's instruction and the amplified DNA was cleaned up with 100 µL VAHTS DNA Clean Beads (Vazyme #N411), eluted in 22 µL DNase‐free water and subjected for sequencing.

### 4.17 Protein Modeling by AlphaFold

The structure of mouse EBF2 (AF‐O08792‐F1‐v4) was predicted by AlphaFold (https://alphafold.ebi.ac.uk/). The model confidence, measured by pLDDT (predicted Local Distance Difference Test), falls within the range of 70<pLDDT<90, indicating an above‐average (pLDDT = 72.16) confidence level. The PDB file is downloaded and visualized using WeMol (https://wemol.wecomput.com/ui/#/).

### 4.18 RNA‐seq Data Analysis

RNA sequencing was performed on polyA‐selected mRNA from the iBAT of control mice and *Cars2* AKO mice and brown adipocytes at the initial and final stages of differentiation. The Fastq files were aligned to the mouse reference genome (mm38) using the aligner STAR [46]. HTSeq was used to count reads mapped to each gene feature [47]. DESeq2 was used for differential expression analysis [48]. Gene enrichment analysis was performed using METASCAPE (https://metascape.org/gp/index.html).

### 4.19 Statistical Analysis

Statistical analysis was performed using GraphPad Prism 9. The measurements were taken from distinct samples. Normality of the data was assessed using the Shapiro–Wilk test and Q–Q plots. For normally distributed data, statistical differences were evaluated via two‐tailed unpaired Student's t tests for comparisons between two groups or analysis of variance (ANOVA) and appropriate post hoc analyses for comparisons of more than two groups. Analysis of covariance (ANCOVA) was employed to compare oxygen consumption and energy expenditure between mice, with body weight included as a covariate for statistical adjustment. All analyses were conducted using the online tool available at https://calrapp.org. Prior to analysis, ANCOVA assumptions were rigorously verified: the homogeneity of error variances was confirmed via Levene's test (*p* > 0.05); the homogeneity of regression slopes was assessed by testing the interaction between body weight and fixed factors (*p* > 0.05); and the normality of residuals was also validated (*p* > 0.05). Adjusted means and standard error of the mean (SEM) were reported only when all assumptions were satisfied. Notably, due to the absence of a linear correlation between body weight and energy expenditure in CL316243‐stimulated experiments, ANCOVA was not applied to these datasets. A p value of less than 0.05 (*p < 0.05, **p < 0.01, and ***p < 0.001) was considered to indicate statistical significance. The statistical methods and corresponding *p* values for the data shown in each panel are included in the figure legends.

## Author Contributions

Hongyong Song, Xin Peng and Xu‐Yun Zhao conceived the project and designed the research. Hongyong Song, Xin Peng performed the majority of the studies. Yin Long, Kaimin Wu, Yinkun Fu and Zhe Shi performed some animal and cell experiments. Wenkai Zhou collected the human clinical samples. Hongyong Song, Xin Peng and Xu‐Yun Zhao analyzed the data and wrote the manuscript.

## Funding

This work was funded by the National Key R&D Program of China (2020YFA0803603 and 2023YFA1800802 to X.Y.Z.), the National Natural Science Foundation of China (32571512 to X.Y.Z and 81902313 to H.S.), and the innovative research team of high‐level local universities in Shanghai (SHSMU‐ZDCX20212501 to X.Y.Z.).

## Ethics Statement

The experimental protocol was approved by the University Committee on the Use and Care of Animals at Shanghai Jiao Tong University (Reference Number: A2022‐064). The research was prospectively reviewed and approved by the Ethics Committee of Shanghai Ninth People's Hospital (Reference Number: 2018‐86‐T77), and informed consent was obtained from the participants. All the research was conducted in accordance with both the Declarations of Helsinki and Istanbul.

## Conflicts of Interest

The authors declare that they have no competing interests.

## Supporting information




**Supporting File 1**: advs74881‐sup‐0001‐SuppMat.docx.

**Supporting File 2**: advs74881‐sup‐0002‐Table S1.pdf.

**Supporting File 3**: advs74881‐sup‐0003‐DataFile.pdf.

## Data Availability

The raw RNA sequencing data are available in the Gene Expression Omnibus database: accession ID GSE242244. The raw metabolomics data are available via MetaboLights (https://www.ebi.ac.uk/metabolights/index) with identifiers MTBLS12234 and MTBLS12237. The authors confirm that the datasets used and analyzed during the current study are available from the corresponding author upon reasonable request.

## References

[1] X.‐F. Pan , L. Wang , and A. Pan , “Epidemiology and Determinants of Obesity in China,” Lancet Diabetes & Endocrinology 9, no. 6 (2021): 373–392, 10.1016/s2213-8587(21)00045-0. 34022156

[2] D. W. Haslam and W. P. T. James , “Obesity,” Lancet 366, no. 9492 (2005): 1197–1209, 10.1016/s0140-6736(05)67483-1. 16198769

[3] G. Seravalle and G. Grassi , “Obesity and Hypertension,” Pharmacological Research 122 (2017): 1–7, 10.1016/j.phrs.2017.05.013. 28532816

[4] U. Peters , A. E. Dixon , and E. Forno , “Obesity and Asthma,” Journal of Allergy and Clinical Immunology 141, no. 4 (2018): 1169–1179, 10.1016/j.jaci.2018.02.004. PMC5973542 29627041

[5] E. J. Gallagher and D. LeRoith , “Obesity and Diabetes: the Increased Risk of Cancer and Cancer‐Related Mortality,” Physiological Reviews 95, no. 3 (2015): 727–748, 10.1152/physrev.00030.2014. PMC4491542 26084689

[6] P. Cohen and S. Kajimura , “The Cellular and Functional Complexity of Thermogenic Fat,” Nature Reviews Molecular Cell Biology 22, no. 6 (2021): 393–409, 10.1038/s41580-021-00350-0. PMC8159882 33758402

[7] S. N. Shapira , H.‐W. Lim , and S. Rajakumari , “EBF2 Transcriptionally Regulates Brown Adipogenesis via the Histone Reader DPF3 and the BAF Chromatin Remodeling Complex,” Genes & development 31, no. 7 (2017): 660–673, 10.1101/gad.294405.116. PMC5411707 28428261

[8] W. Wang and P. Seale , “Control of Brown and Beige Fat Development,” Nature Reviews Molecular Cell Biology 17, no. 11 (2016): 691–702, 10.1038/nrm.2016.96. PMC5627770 27552974

[9] W. Wang , M. Kissig , and S. Rajakumari , “EBF2 is a Selective Marker of Brown and Beige Adipogenic Precursor Cells,” Proceedings of the National Academy of Sciences 111, no. 40 (2014): 14466–14471, 10.1073/pnas.1412685111. PMC4209986 25197048

[10] X.‐Y. Zhao , S. Li , G.‐X. Wang , Q. Yu , and J. D. Lin , “A Long Noncoding RNA Transcriptional Regulatory Circuit Drives Thermogenic Adipocyte Differentiation,” Molecular Cell 55, no. 3 (2014): 372–382, 10.1016/j.molcel.2014.06.004. PMC4127104 25002143

[11] S. Rajakumari , J. Wu , and J. Ishibashi , “EBF2 determines and Maintains Brown Adipocyte Identity,” Cell Metabolism 17, no. 4 (2013): 562–574, 10.1016/j.cmet.2013.01.015. PMC3622114 23499423

[12] A. R. Angueira , S. N. Shapira , and J. Ishibashi , “Early B Cell Factor Activity Controls Developmental and Adaptive Thermogenic Gene Programming in Adipocytes,” Cell Reports 30, no. 9 (2020): 2869–2878.e4, 10.1016/j.celrep.2020.02.023. PMC7079313 32130892

[13] Q.‐X. Ma , W.‐Y. Zhu , and X.‐C. Lu , “BCAA–BCKA Axis Regulates WAT Browning through Acetylation of PRDM16,” Nature Metabolism 4, no. 1 (2022): 106–122, 10.1038/s42255-021-00520-6. 35075301

[14] L. O. Leiria , C.‐H. Wang , and M. D. Lynes , “12‐Lipoxygenase Regulates Cold Adaptation and Glucose Metabolism by Producing the Omega‐3 Lipid 12‐HEPE from Brown Fat,” Cell Metabolism 30, no. 4 (2019): 768–783, 10.1016/j.cmet.2019.07.001. PMC6774888 31353262

[15] B. Niemann , S. Haufs‐Brusberg , and L. Puetz , “Apoptotic Brown Adipocytes Enhance Energy Expenditure via Extracellular Inosine,” Nature 609, no. 7926 (2022): 361–368, 10.1038/s41586-022-05041-0. PMC9452294 35790189

[16] W. Wang , J. Ishibashi , and S. Trefely , “A PRDM16‐Driven Metabolic Signal from Adipocytes Regulates Precursor Cell Fate,” Cell Metabolism 30, no. 1 (2019): 174–189, 10.1016/j.cmet.2019.05.005. PMC6836679 31155495

[17] G. J. McBean , “Cysteine, Glutathione, and Thiol Redox Balance in Astrocytes,” Antioxidants (Basel) 6, no. 3 (2017): 62, 10.3390/antiox6030062. PMC5618090 28771170

[18] T. Song , W. Qin , and Z. Lai , “Dietary Cysteine Drives Body Fat Loss via FMRFamide Signaling in Drosophila and Mouse,” Cell Research 33, no. 6 (2023): 434–447, 10.1038/s41422-023-00800-8. PMC10235132 37055592

[19] Q. Zheng , L. Pan , and Y. Ji , “H_2_S Protects against Diabetes‐accelerated Atherosclerosis by Preventing the Activation of NLRP3 Inflammasome,” The Journal of Biomedical Research 34, no. 2 (2020): 94–102, 10.7555/jbr.33.20190071. PMC7183300 32305963

[20] S. Zhao , T. Song , and Y. Gu , “Hydrogen Sulfide Alleviates Liver Injury through the S‐Sulfhydrated‐Kelch‐like ECH‐Associated Protein 1/Nuclear Erythroid 2–Related Factor 2/Low‐Density Lipoprotein Receptor–Related Protein 1 Pathway,” Hepatology 73, no. 1 (2021): 282–302, 10.1002/hep.31247. 32219872

[21] M. R. Filipovic , J. Zivanovic , B. Alvarez , and R. Banerjee , “Chemical Biology of H_2_S Signaling through Persulfidation,” Chemical Reviews 118, no. 3 (2018): 1253–1337, 10.1021/acs.chemrev.7b00205. PMC6029264 29112440

[22] B. D. Paul and S. H. Snyder , “H_2_S: A Novel Gasotransmitter That Signals by Sulfhydration,” Trends in Biochemical Sciences 40, no. 11 (2015): 687–700, 10.1016/j.tibs.2015.08.007. PMC4630104 26439534

[23] J. B. Vicente , F. Malagrinò , M. Arese , E. Forte , P. Sarti , and A. Giuffrè , “Bioenergetic Relevance of Hydrogen Sulfide and the Interplay between Gasotransmitters at human Cystathionine β‐synthase,” Biochimica et Biophysica Acta (BBA)—Bioenergetics 1857, no. 8 (2016): 1127–1138, 10.1016/j.bbabio.2016.03.030. 27039165

[24] B. D. Paul , J. I. Sbodio , and R. Xu , “Cystathionine γ‐Lyase Deficiency Mediates Neurodegeneration in Huntington's Disease,” Nature 509, no. 7498 (2014): 96–100, 10.1038/nature13136. PMC4349202 24670645

[25] T. Akaike , T. Ida , and F.‐Y. Wei , “Cysteinyl‐tRNA Synthetase Governs Cysteine Polysulfidation and Mitochondrial Bioenergetics,” Nature Communications 8, no. 1 (2017): 1177, 10.1038/s41467-017-01311-y. PMC5660078 29079736

[26] Y.‐Y. Guo , B.‐Y. Li , W.‐Q. Peng , L. Guo , and Q.‐Q. Tang , “Taurine‐Mediated Browning of White Adipose Tissue Is Involved in Its Anti‐obesity Effect in Mice,” Journal of Biological Chemistry 294, no. 41 (2019): 15014–15024, 10.1074/jbc.RA119.009936. PMC6791308 31427436

[27] A. K. Mustafa , M. M. Gadalla , N. Sen , et al., “H_2_S Signals through Protein S‐Sulfhydration,” Science Signaling 2, no. 96 (2009): 2000464, 10.1126/scisignal.2000464. PMC2998899 19903941

[28] T. Inagaki , J. Sakai , and S. Kajimura , “Transcriptional and Epigenetic Control of Brown and Beige Adipose Cell Fate and Function,” Nature Reviews Molecular Cell Biology 17, no. 8 (2016): 480–495, 10.1038/nrm.2016.62. PMC4956538 27251423

[29] S. Kasamatsu , T. Ida , and T. Koga , “High‐Precision Sulfur Metabolomics Innovated by a New Specific Probe for Trapping Reactive Sulfur Species,” Antioxidants & Redox Signaling 34, no. 18 (2021): 1407–1419, 10.1089/ars.2020.8073. 33198504

[30] L. Li and P. K. Moore , “Putative Biological Roles of Hydrogen Sulfide in Health and Disease: A Breath of Not so Fresh Air?,” Trends in Pharmacological Sciences 29, no. 2 (2008): 84–90, 10.1016/j.tips.2007.11.003. 18180046

[31] C. Szabo , “Hydrogen Sulphide and Its Therapeutic Potential,” Nature Reviews Drug Discovery 6, no. 11 (2007): 917–935, 10.1038/nrd2425. 17948022

[32] W. Yang , G. Yang , X. Jia , L. Wu , and R. Wang , “Activation of K ATP Channels by H_2_S in Rat Insulin‐Secreting Cells and the Underlying Mechanisms,” The Journal of Physiology 569, no. 2 (2005): 519–531, 10.1113/jphysiol.2005.097642. PMC1464240 16179362

[33] J. R. S. Arch , “Challenges in β_3_ ‐adrenoceptor Agonist Drug Development,” Therapeutic Advances in Endocrinology and Metabolism 2, no. 2 (2011): 59–64, 10.1177/2042018811398517. PMC3474627 23148171

[34] S. Ghosh‐Choudhary , J. Liu , and T. Finkel , “Metabolic Regulation of Cell Fate and Function,” Trends in Cell Biology 30, no. 3 (2020): 201–212, 10.1016/j.tcb.2019.12.005. PMC7043867 31983571

[35] A. H. Lee , L. Orliaguet , and Y.‐H. Youm , “Cysteine Depletion Triggers Adipose Tissue Thermogenesis and Weight Loss,” Nature Metabolism 7, no. 6 (2025): 1204–1222, 10.1038/s42255-025-01297-8. PMC12198010 40461845

[36] D. Ma , M. M. Molusky , and J. Song , “Autophagy Deficiency by Hepatic FIP200 Deletion Uncouples Steatosis from Liver Injury in NAFLD,” Molecular Endocrinology 27, no. 10 (2013): 1643–1654, 10.1210/me.2013-1153. PMC4061382 23960084

[37] S. Virtue , C. J. Lelliott , and A. Vidal‐Puig , “What Is the Most Appropriate Covariate in ANCOVA When Analysing Metabolic Rate?,” Nature Metabolism 3, no. 12 (2021): 1585, 10.1038/s42255-021-00505-5. 34903886

[38] T. D. Müller , M. Klingenspor , and M. H. Tschöp , “Revisiting Energy Expenditure: How to Correct Mouse Metabolic Rate for Body Mass,” Nature Metabolism 3, no. 9 (2021): 1134–1136, 10.1038/s42255-021-00451-2. 34489606

[39] X. Liu , A. He , and D. Lu , “Peroxisomal Metabolism of Branched Fatty Acids Regulates Energy Homeostasis,” Nature 646 (2025): 1223–1231, 10.1038/s41586-025-09517-7. PMC12571886 40963015

[40] V. Jimenez , S. Muñoz , and E. Casana , “In Vivo Adeno‐Associated Viral Vector–Mediated Genetic Engineering of White and Brown Adipose Tissue in Adult Mice,” Diabetes 62, no. 12 (2013): 4012–4022, 10.2337/db13-0311. PMC3837045 24043756

[41] J. Klein , M. Fasshauer , M. Ito , B. B. Lowell , M. Benito , and C. R. Kahn , “β_3_‐Adrenergic Stimulation Differentially Inhibits Insulin Signaling and Decreases Insulin‐induced Glucose Uptake in Brown Adipocytes,” Journal of Biological Chemistry 274, no. 49 (1999): 34795–34802, 10.1074/jbc.274.49.34795. 10574950

[42] A. M. Cypess , B. Cannon , and J. Nedergaard , “Emerging Debates and Resolutions in Brown Adipose Tissue Research,” Cell Metabolism 37, no. 1 (2025): 12–33, 10.1016/j.cmet.2024.11.002. PMC11710994 39644896

[43] Y. Lin , Y. Wu , and J. Li , “The SNAG Domain of Snail1 Functions as a Molecular Hook for Recruiting Lysine‐Specific Demethylase 1,” The EMBO Journal 29, no. 11 (2010): 1803–1816, 10.1038/emboj.2010.63. PMC2885925 20389281

[44] B. P. Zhou , J. Deng , and W. Xia , “Dual Regulation of Snail by GSK‐3β‐mediated Phosphorylation in Control of Epithelial–Mesenchymal Transition,” Nature Cell Biology 6, no. 10 (2004): 931–940, 10.1038/ncb1173. 15448698

[45] B. D. Paul and S. H. Snyder , “Protein Sulfhydration,” Methods in Enzymology 555 (2015): 79–90, 10.1016/bs.mie.2014.11.021. 25747476

[46] A. Dobin , C. A. Davis , and F. Schlesinger , “STAR: Ultrafast Universal RNA‐seq Aligner,” Bioinformatics 29, no. 1 (2013): 15–21, 10.1093/bioinformatics/bts635. PMC3530905 23104886

[47] S. Anders , P. T. Pyl , and W. Huber , “HTSeq—A Python Framework to Work With High‐Throughput Sequencing Data,” Bioinformatics 31, no. 2 (2015): 166–169, 10.1093/bioinformatics/btu638. PMC4287950 25260700

[48] M. I. Love , W. Huber , and S. Anders , “Moderated Estimation of Fold Change and Dispersion for RNA‐seq Data with DESeq2,” Genome Biology 15, no. 12 (2014): 550, 10.1186/s13059-014-0550-8. PMC4302049 25516281

