# Supplementary material for: Cars2‐Mediated Cysteine Catabolism Drives Brown Fat Development and Thermogenesis Through Persulfidating EBF2
Source: Adv Sci (Weinh). 2026 Mar 18;13(31):e22690. doi: 10.1002/advs.202522690 (PMC13588024; doi:10.1002/advs.202522690)

Fig.1F

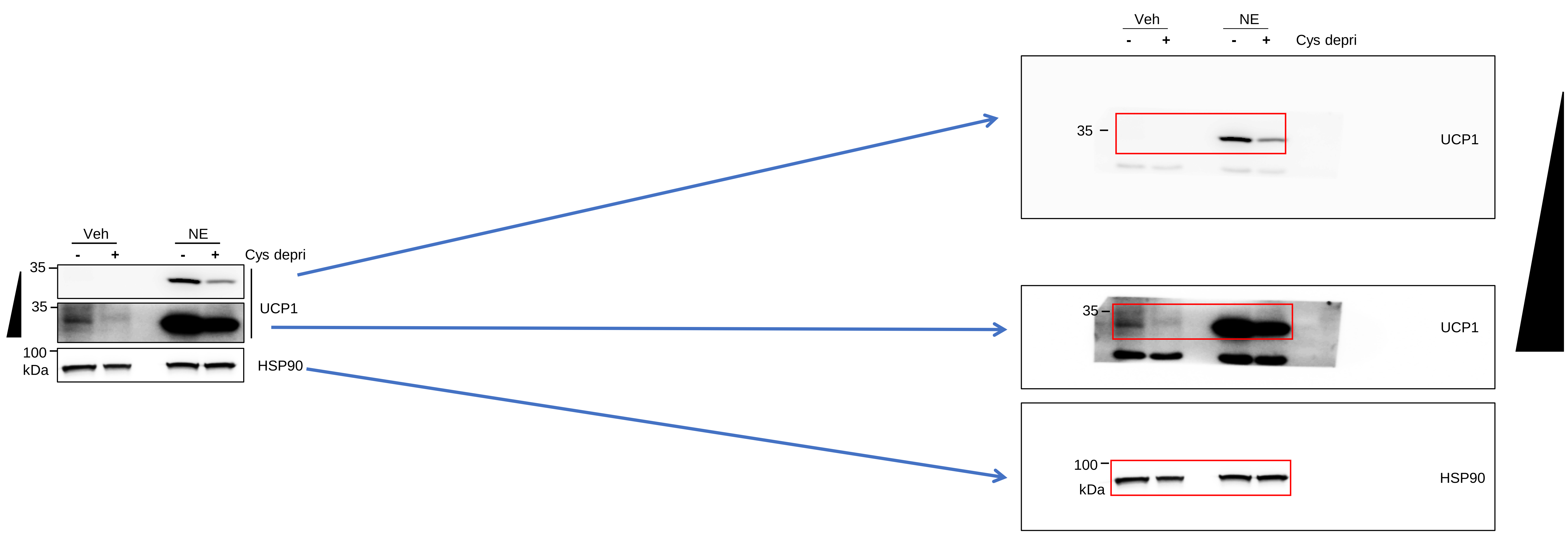

Figure 1

Fig.2D

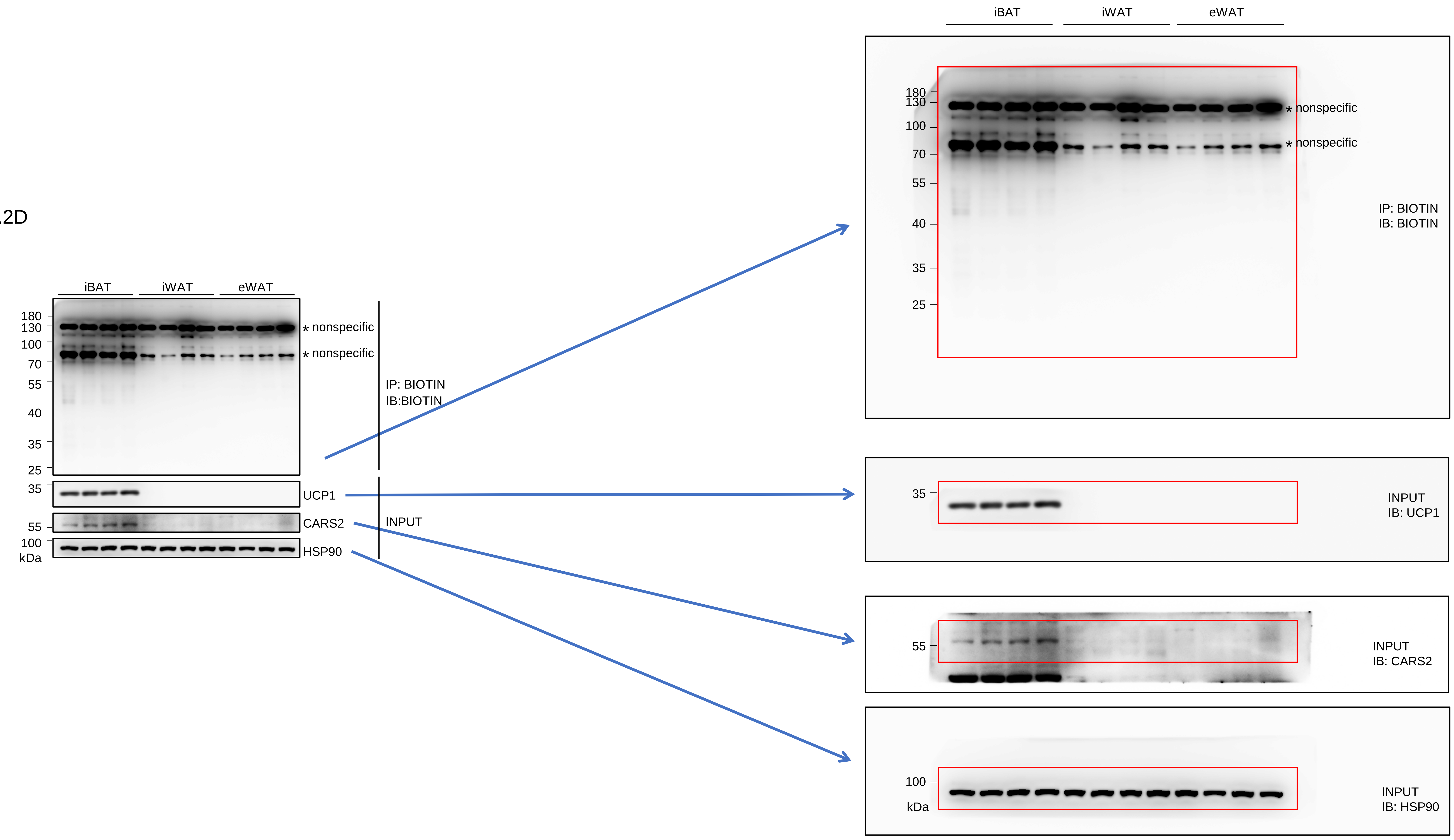

Fig.2F

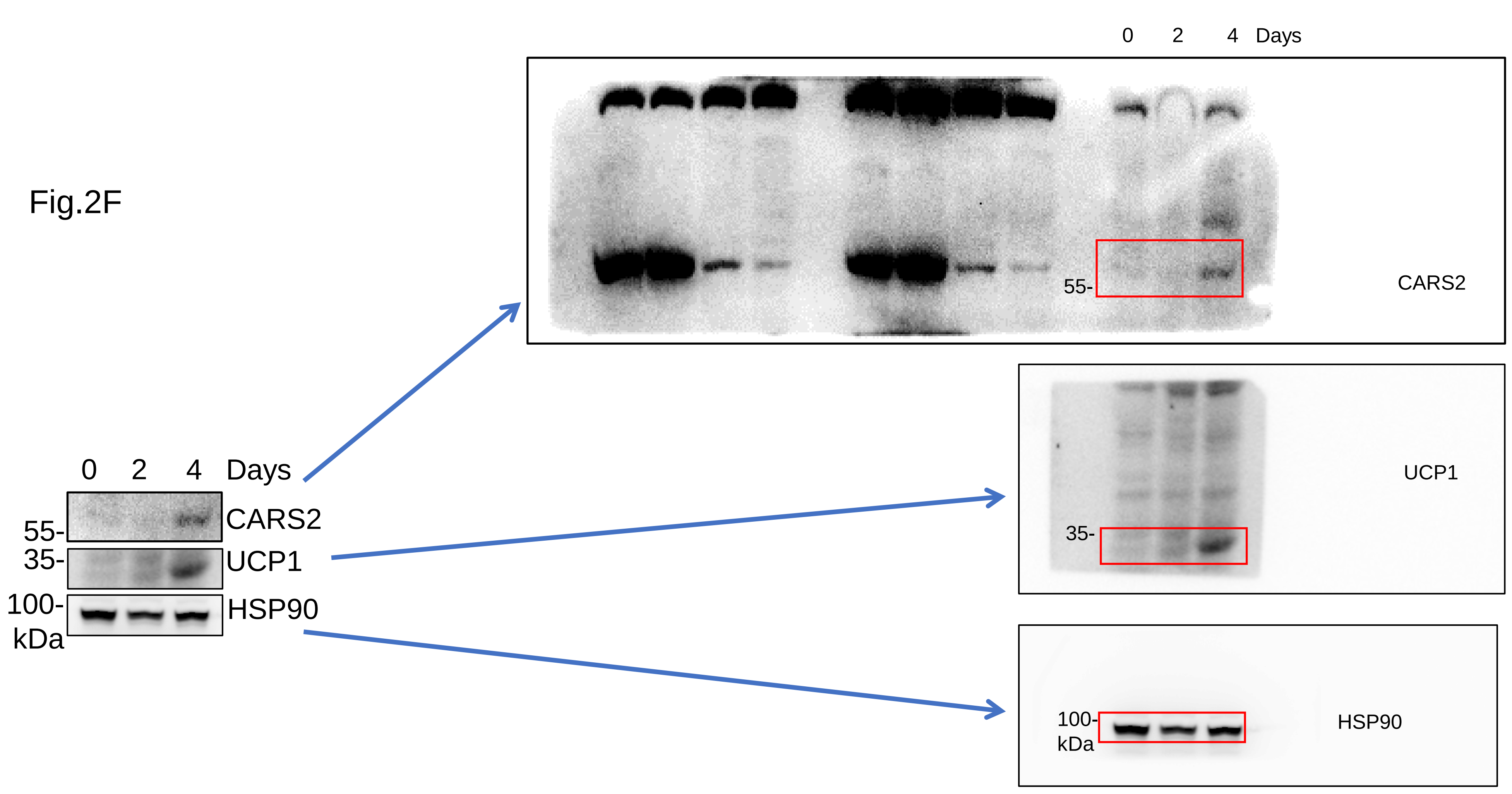

Fig.2H

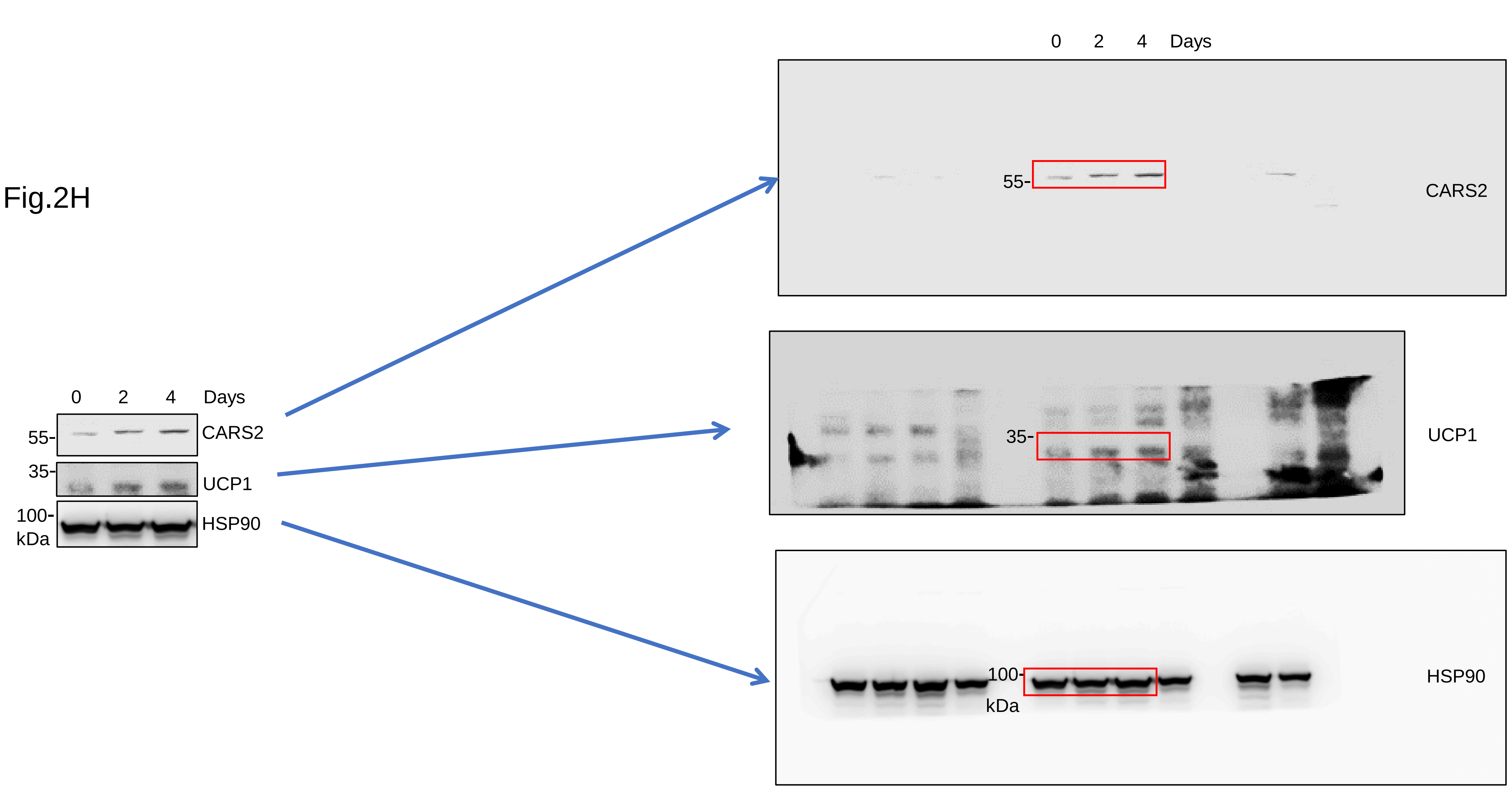

Figure 2

Fig 2I

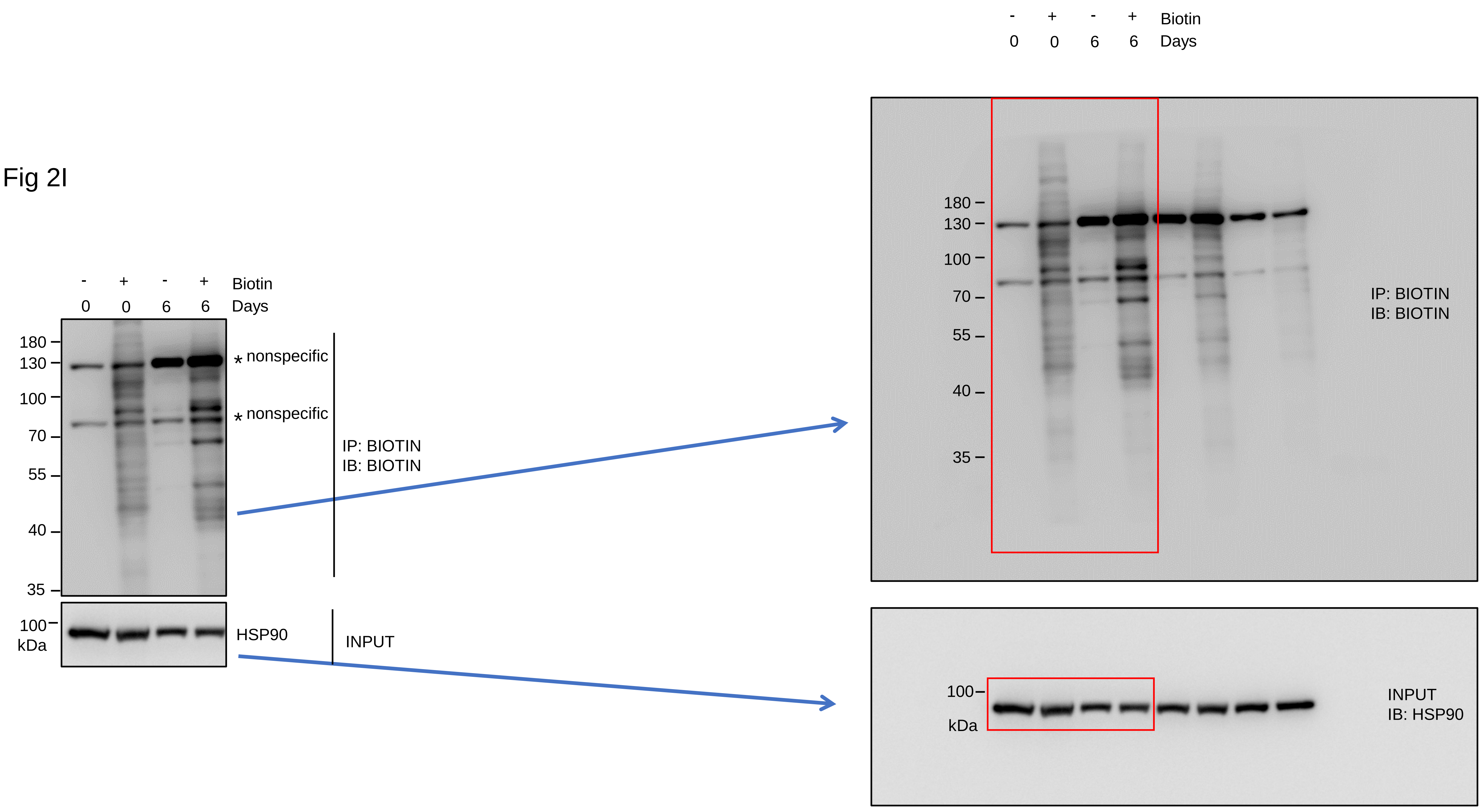

Fig 2J

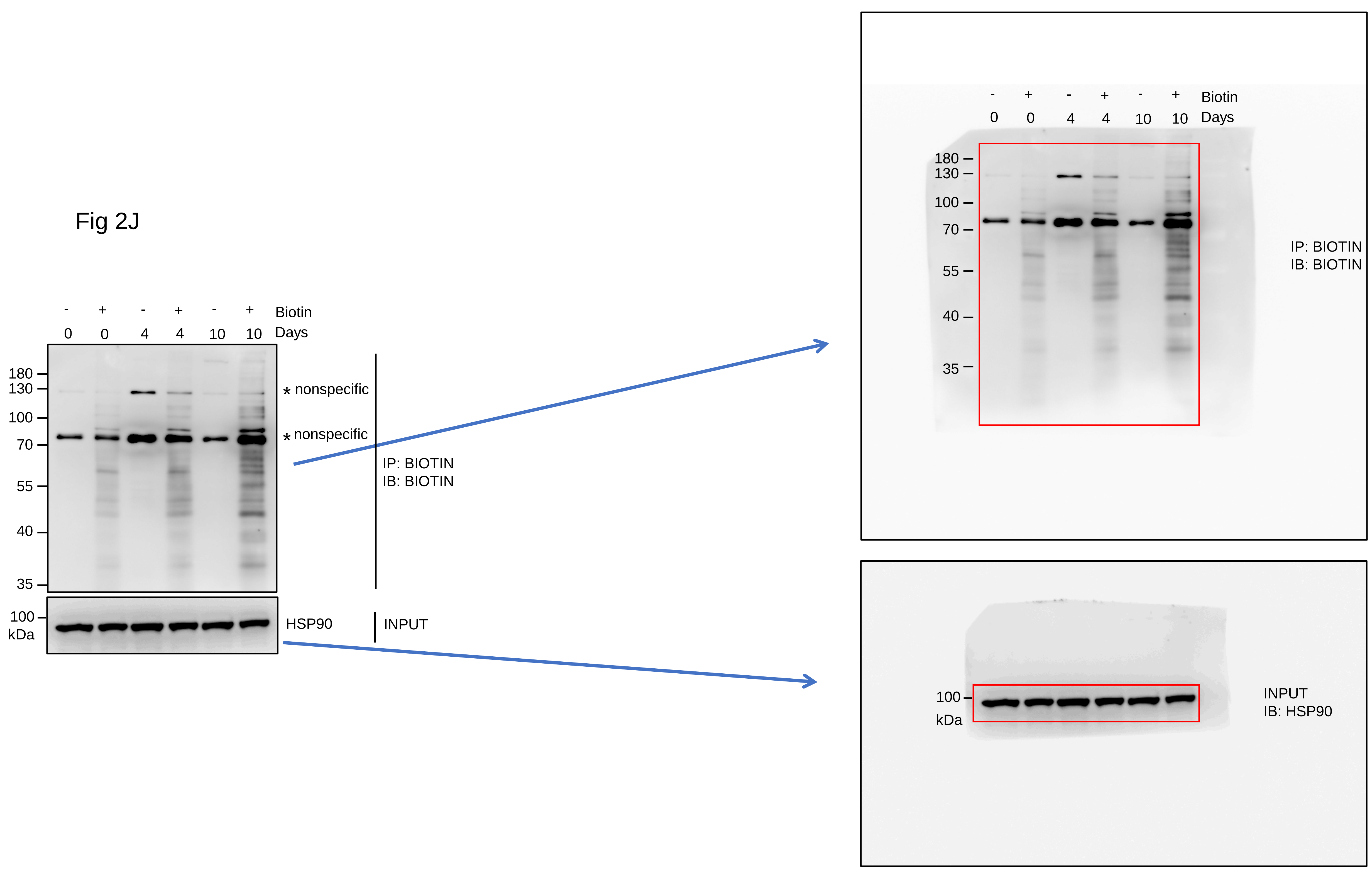

Figure 2

Fig 2L

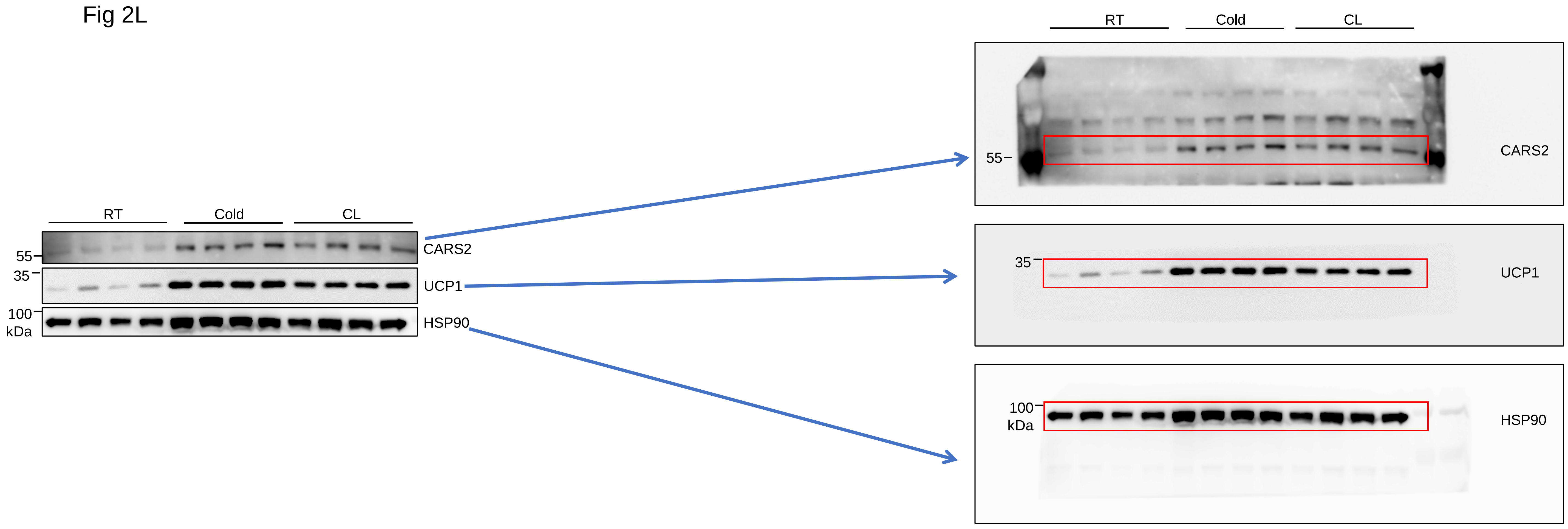

Figure 2

Fig 2M

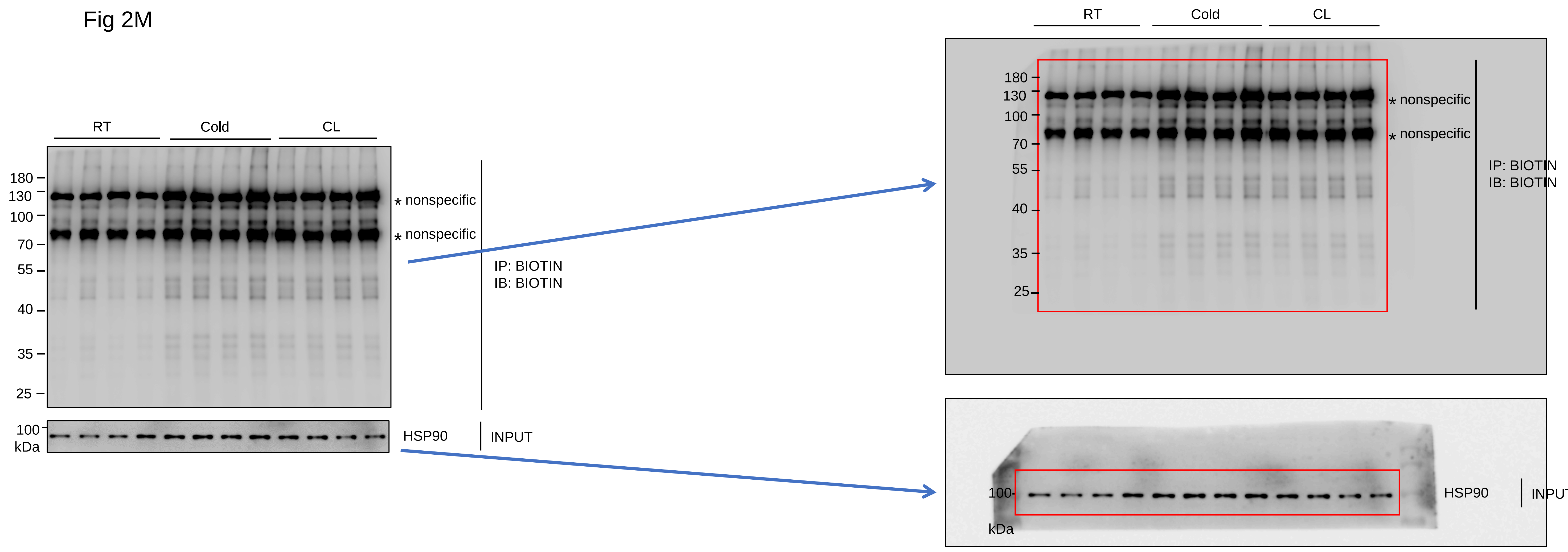

Fig 2O

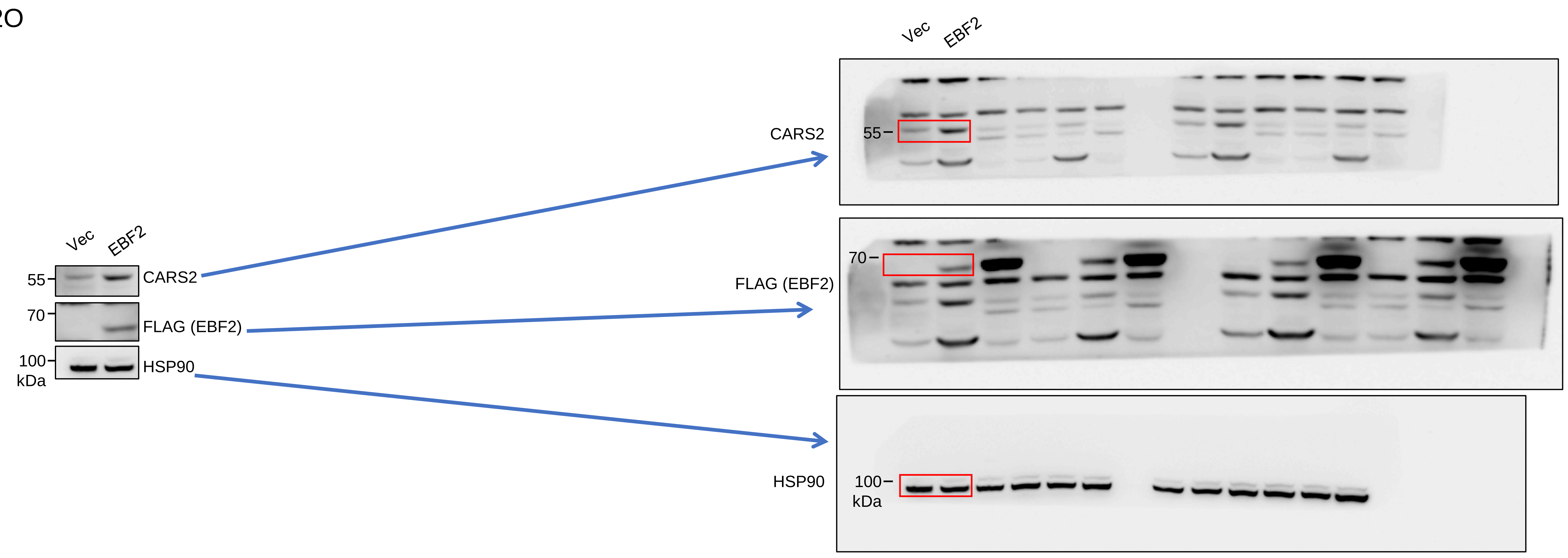

Fig 2Q

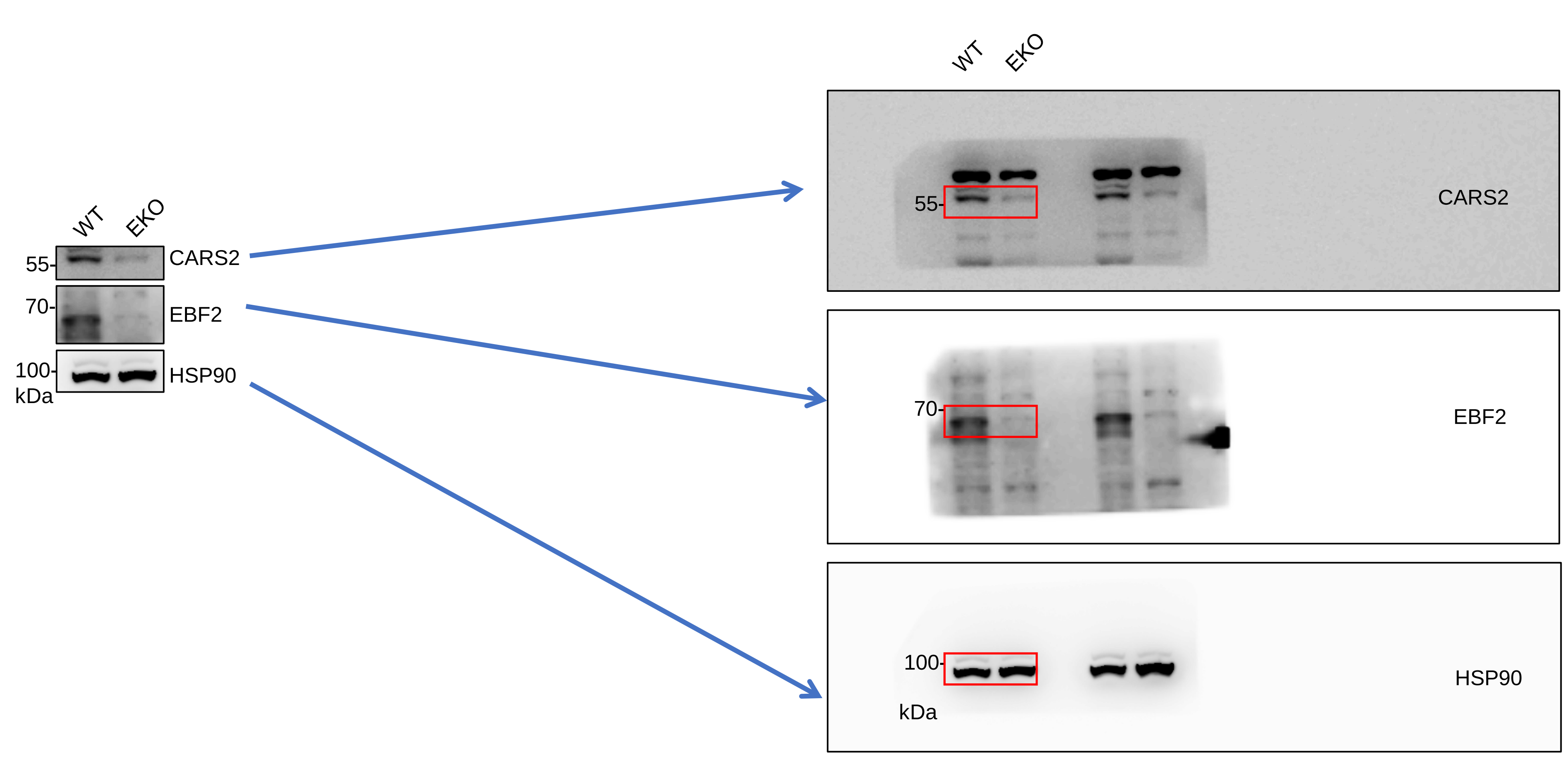

Figure 2

Fig 3A

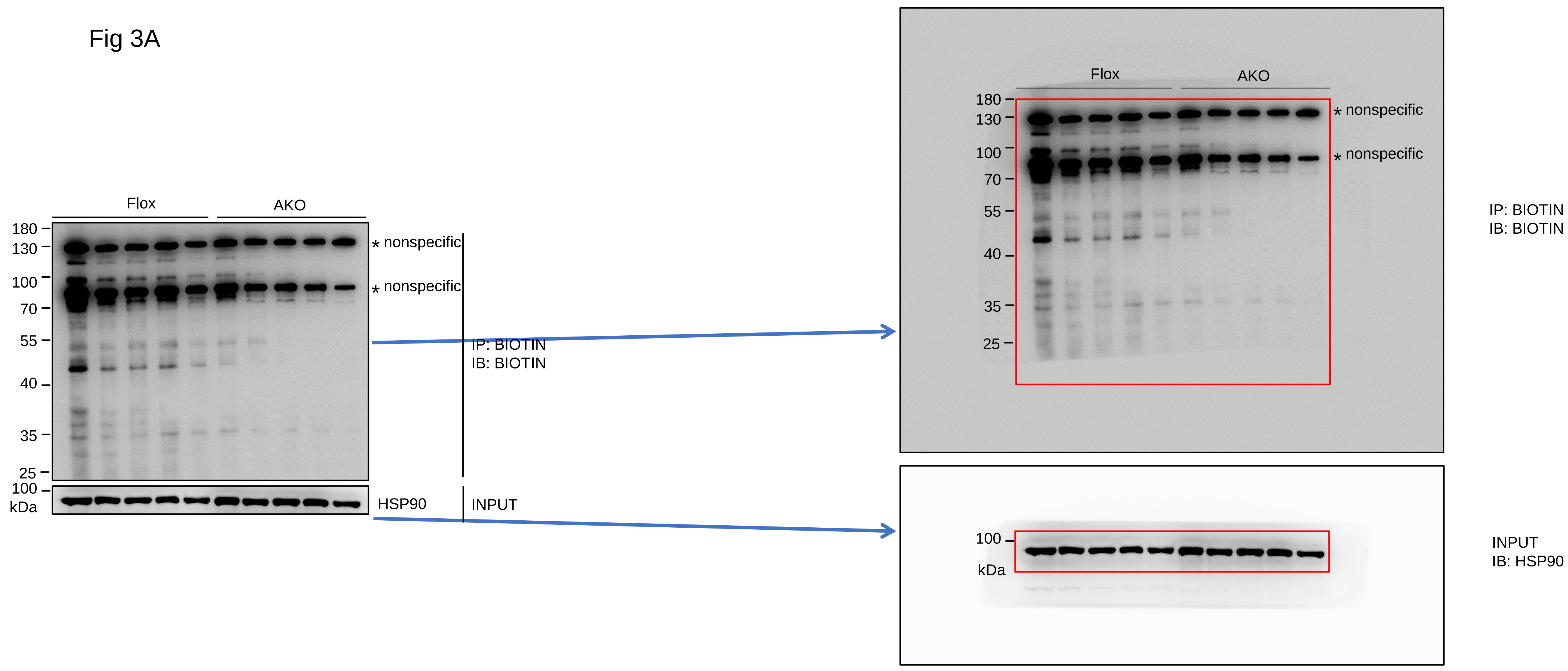

Fig 3B

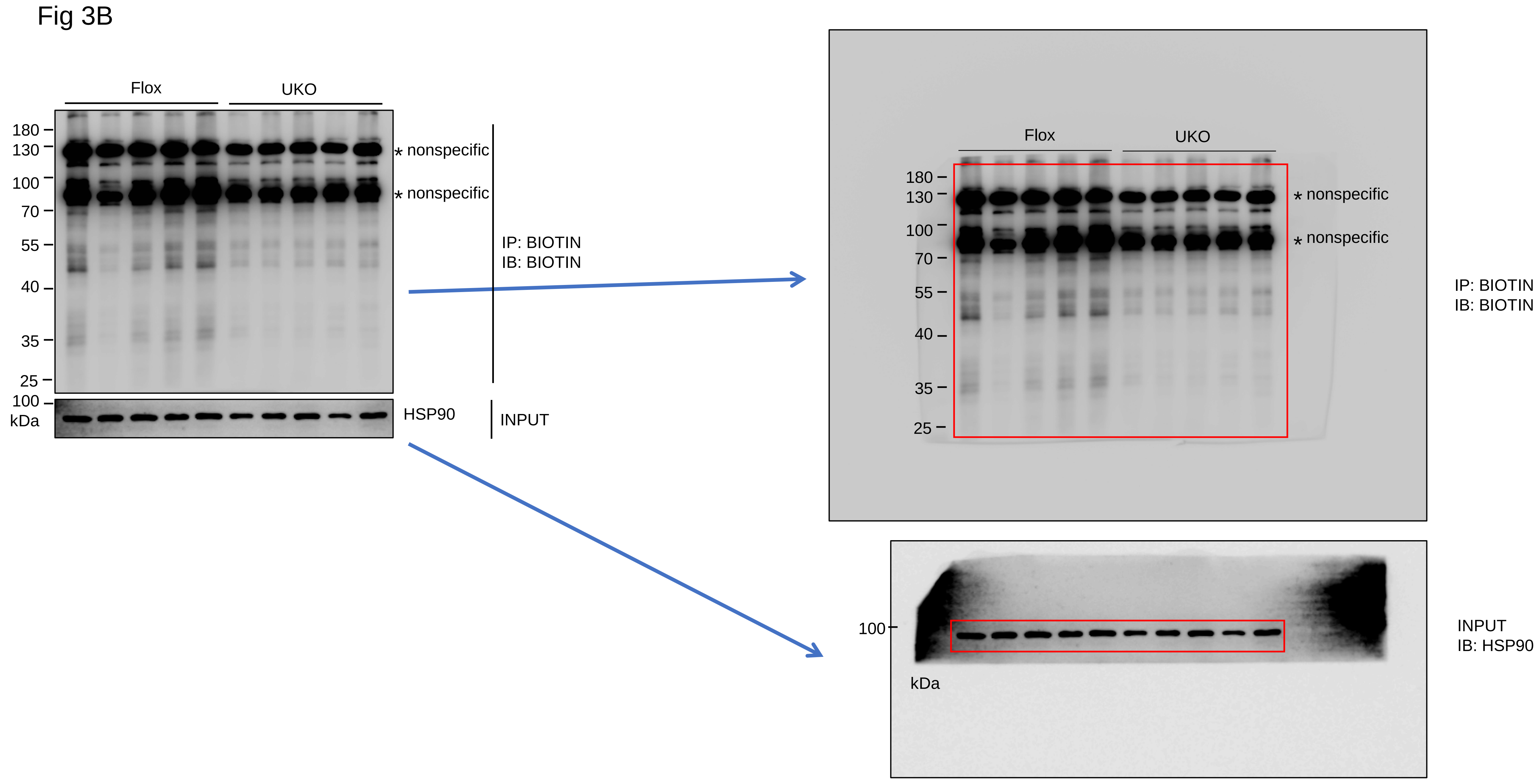

Figure 3

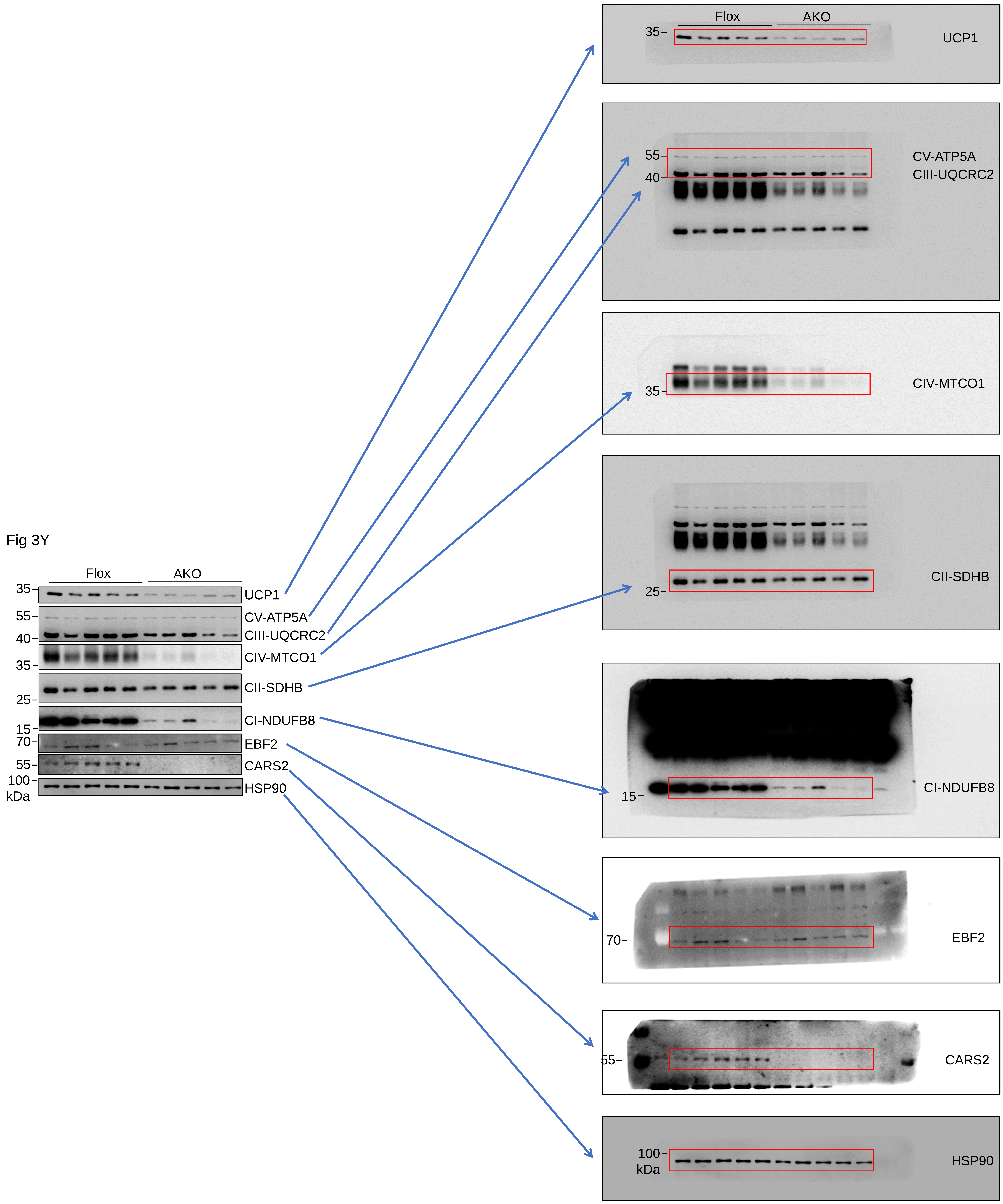
Fig 3Y
Flox
AKO
35-
55-
40-
35-
25-
15-
70-
55-
100-
kDa
UCP1
CV-ATP5A
CIII-UQCRC2
CIV-MTCO1
CII-SDHB
CI-NDUFB8
EBF2
CARS2
HSP90

Figure 3

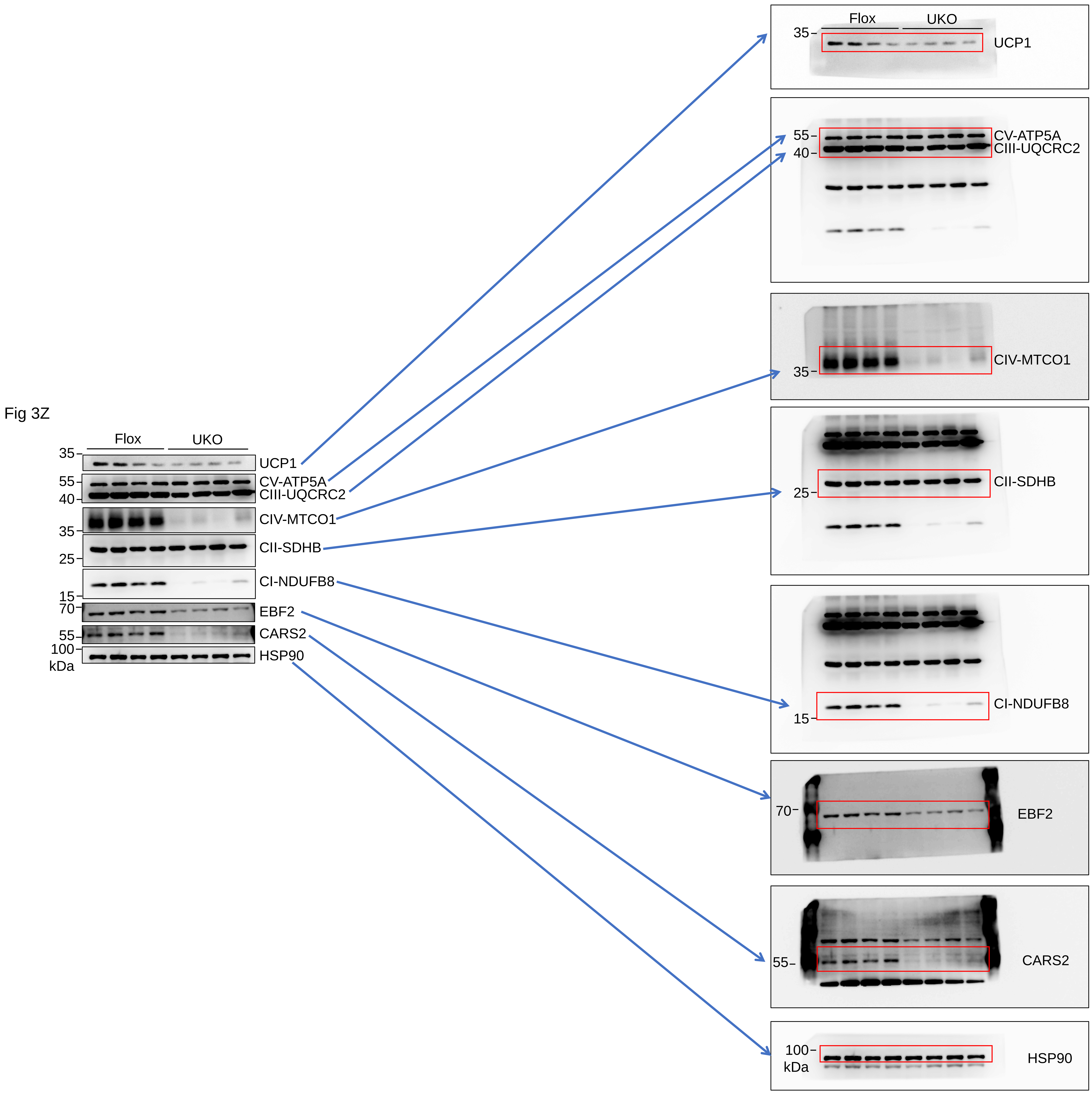
Fig 3Z
Flox
UKO
35
55
40
35
25
15
70
55
100
kDa
UCP1
CV-ATP5A
CIII-UQCRC2
CIV-MTCO1
CII-SDHB
CI-NDUFB8
EBF2
CARS2
HSP90

Figure 3

Fig.4B

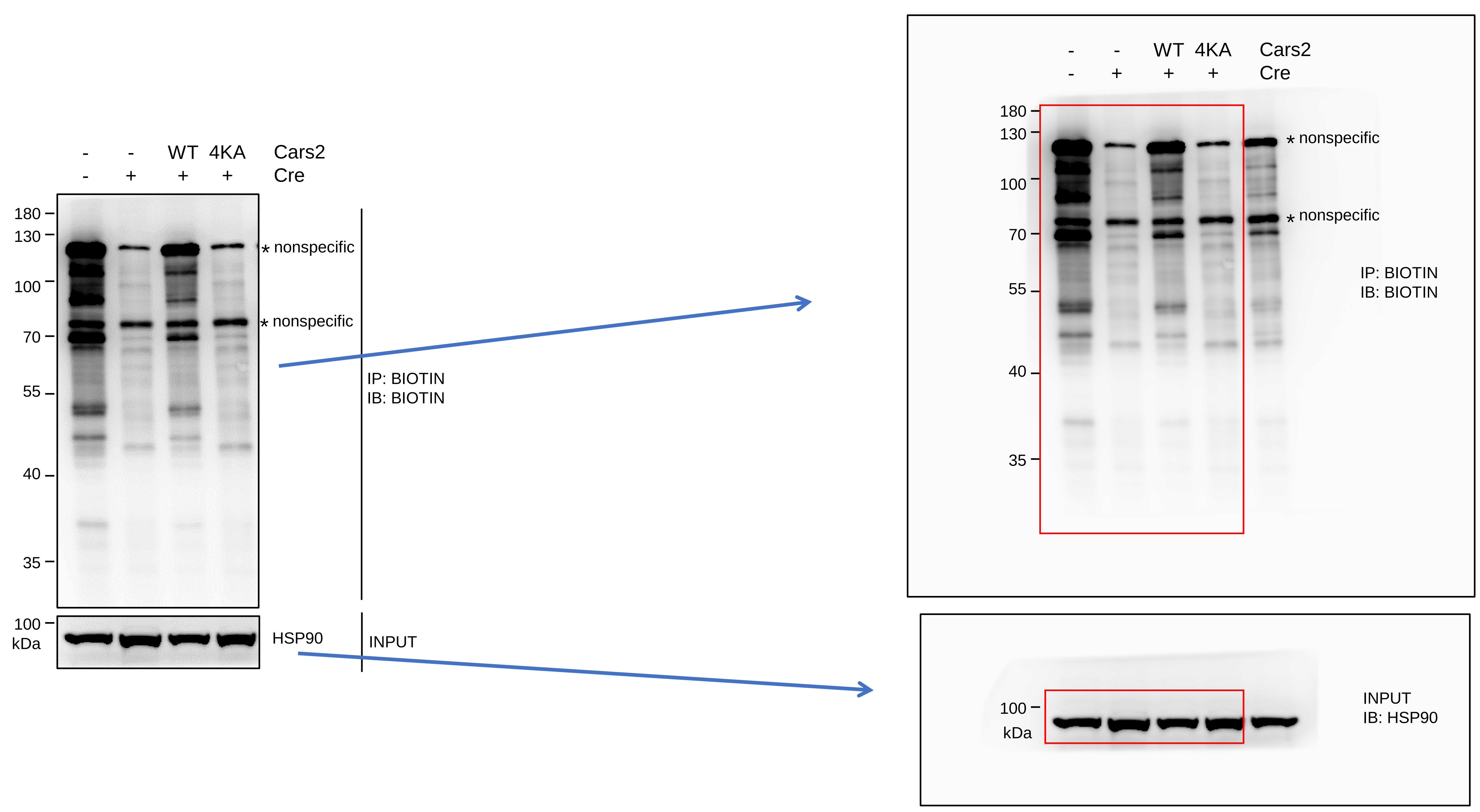

Figure 4

Fig.4D

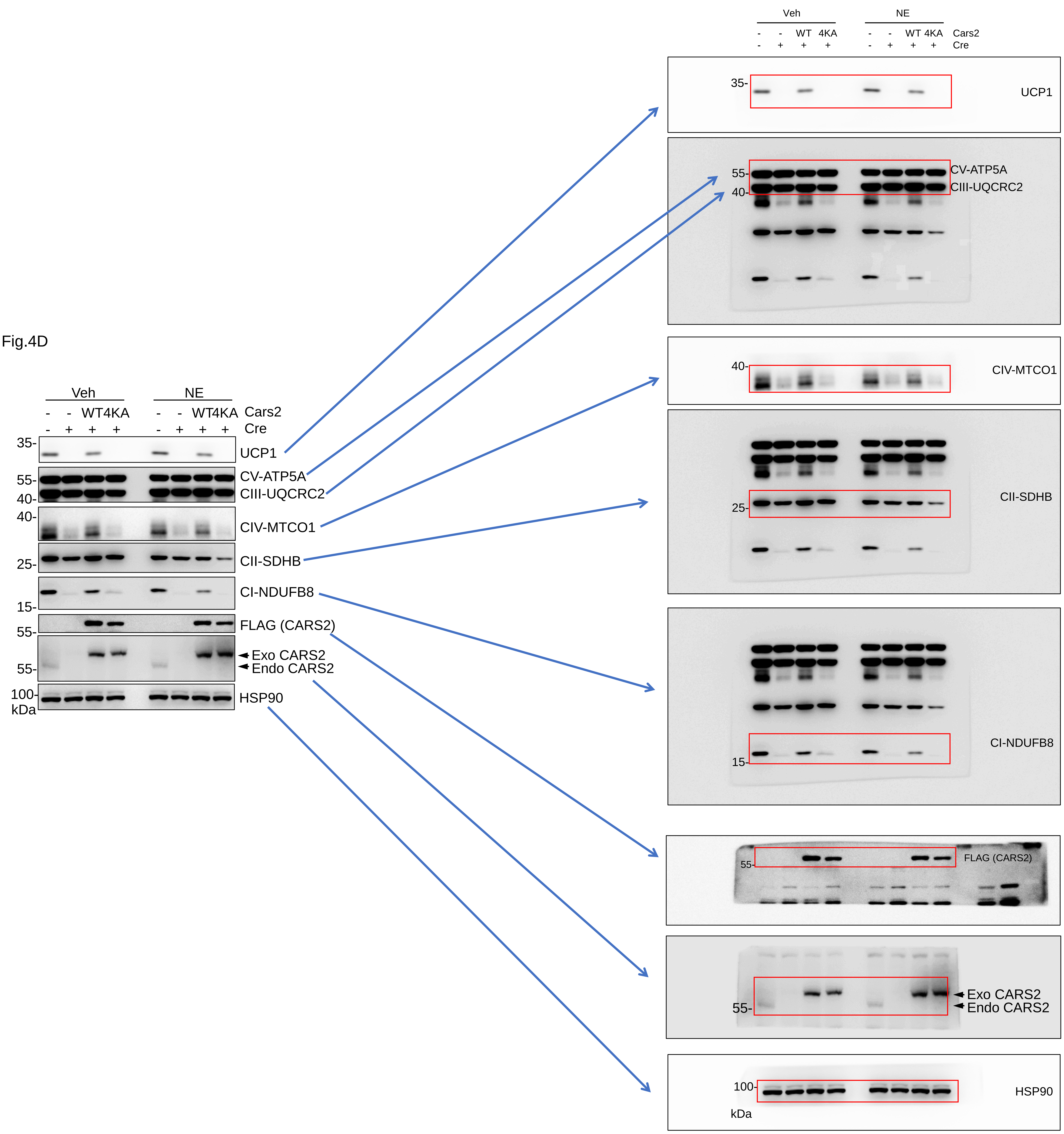

Figure 4

Fig.4H

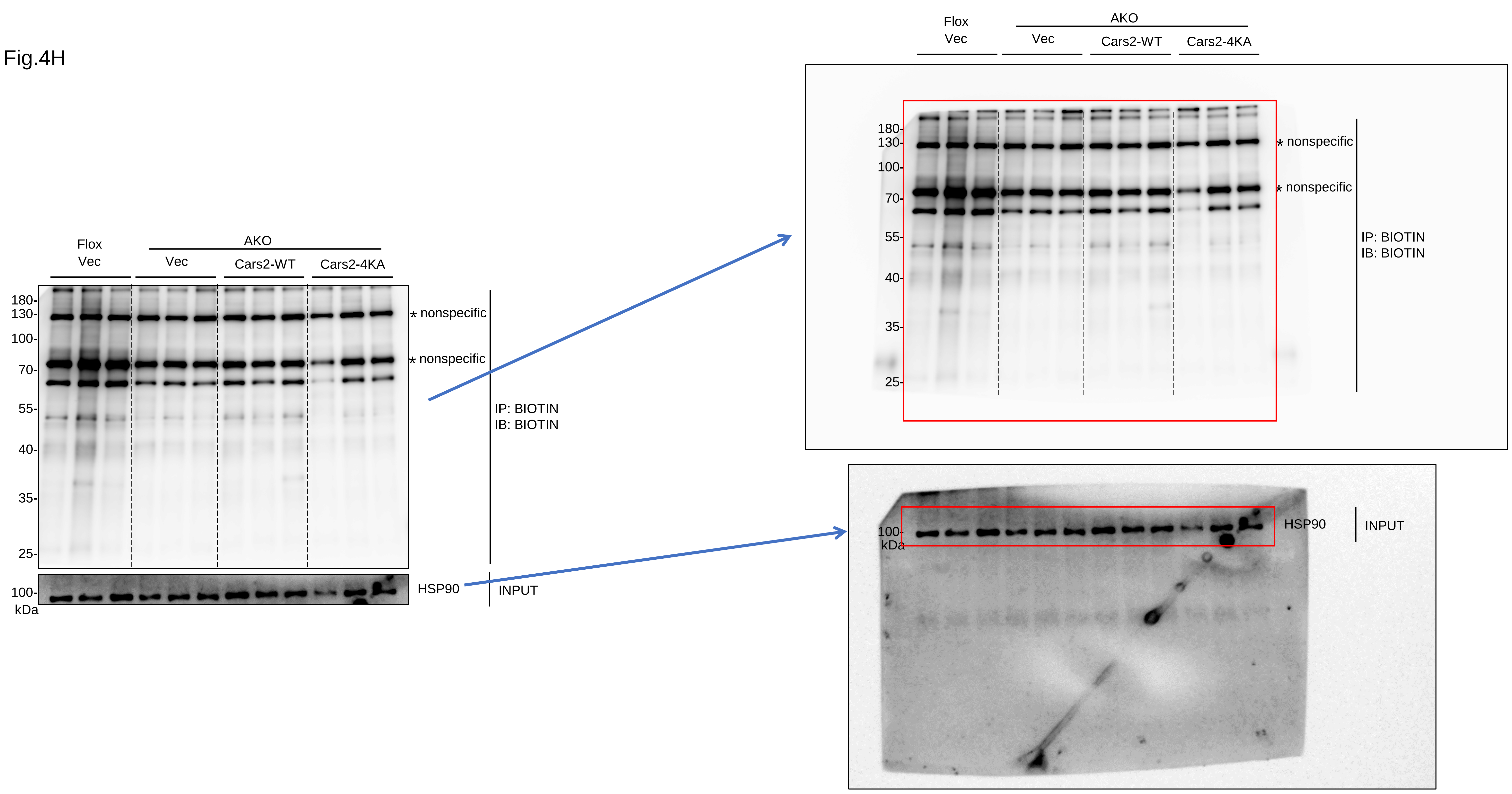

Figure 4

Fig.4L

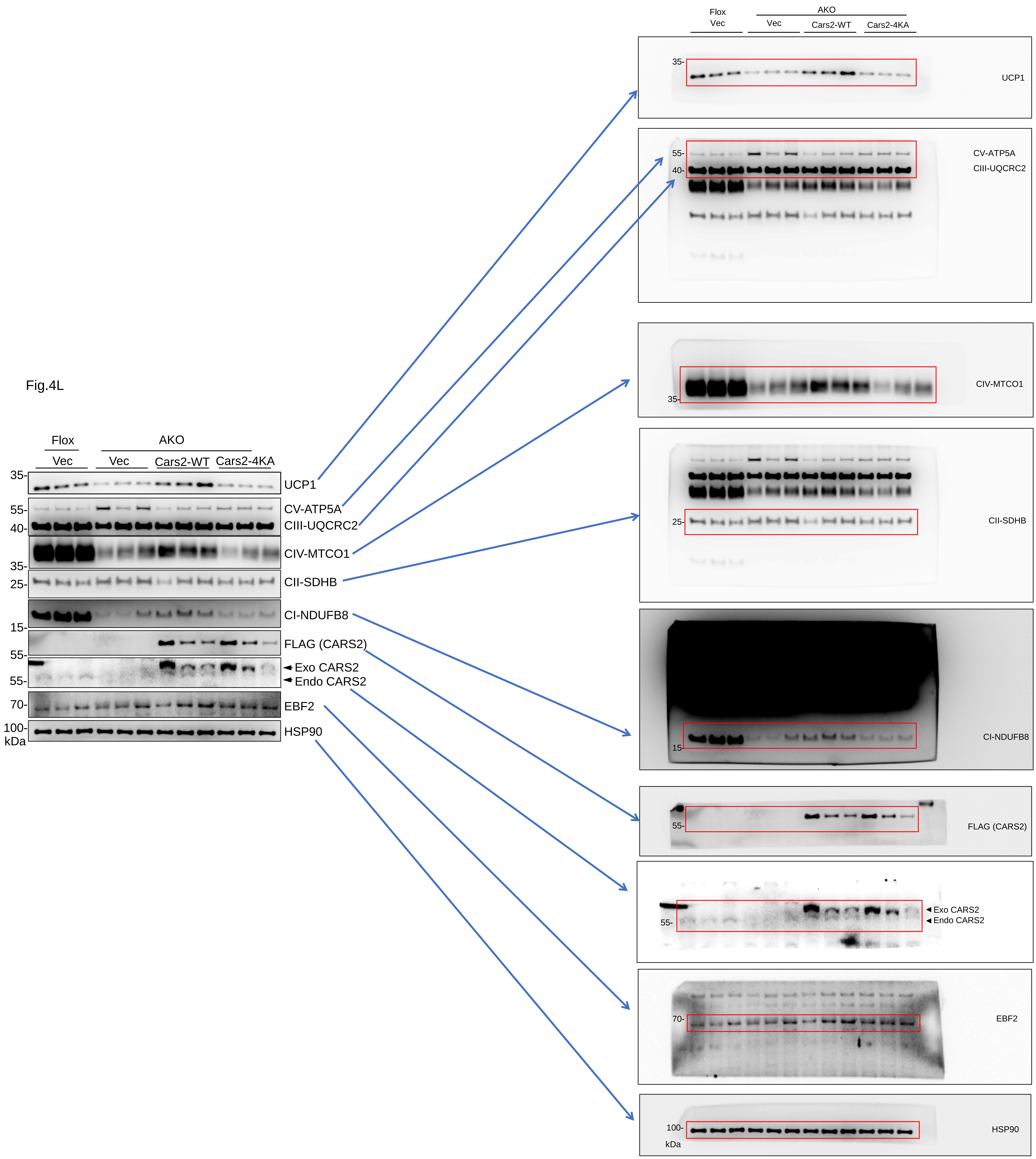

Figure 4

Fig.5B

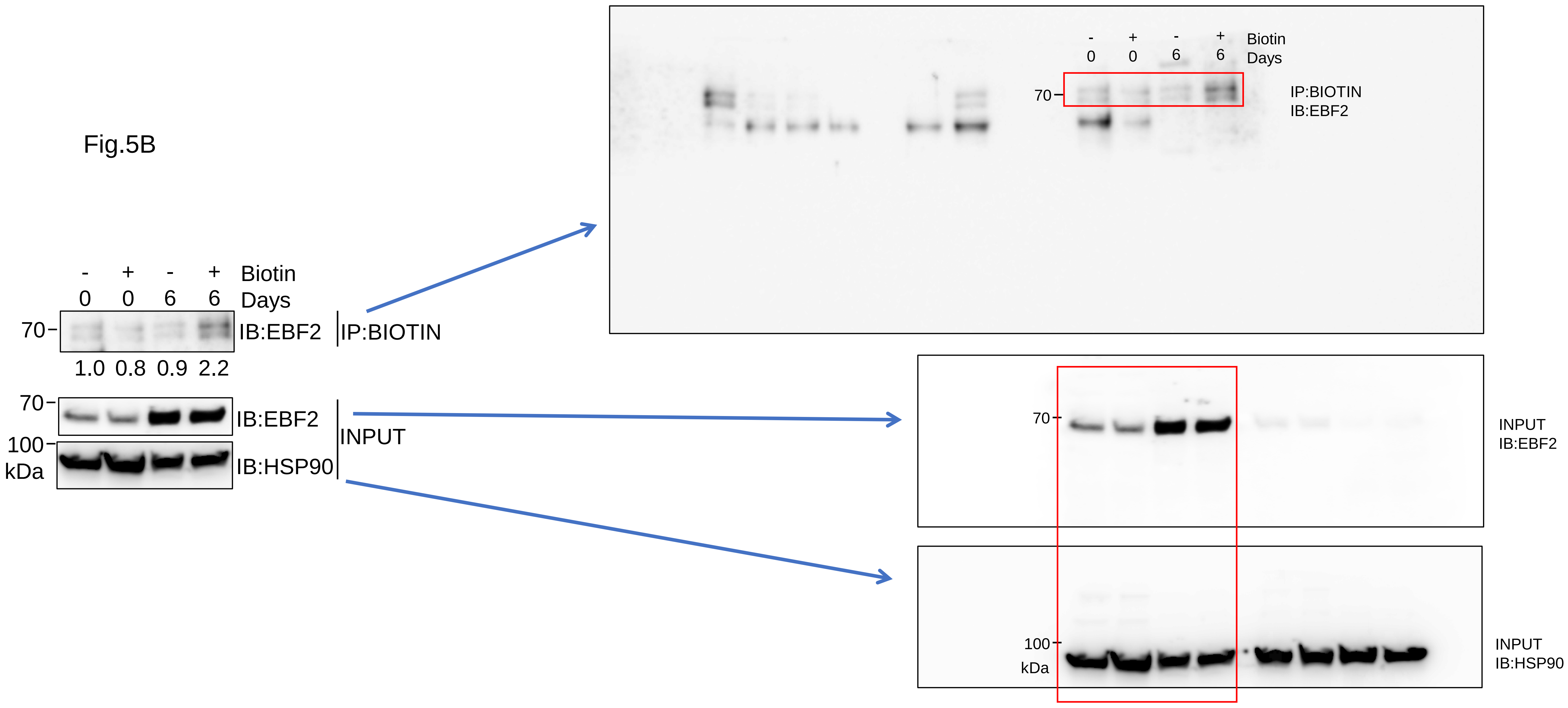

Fig.5C

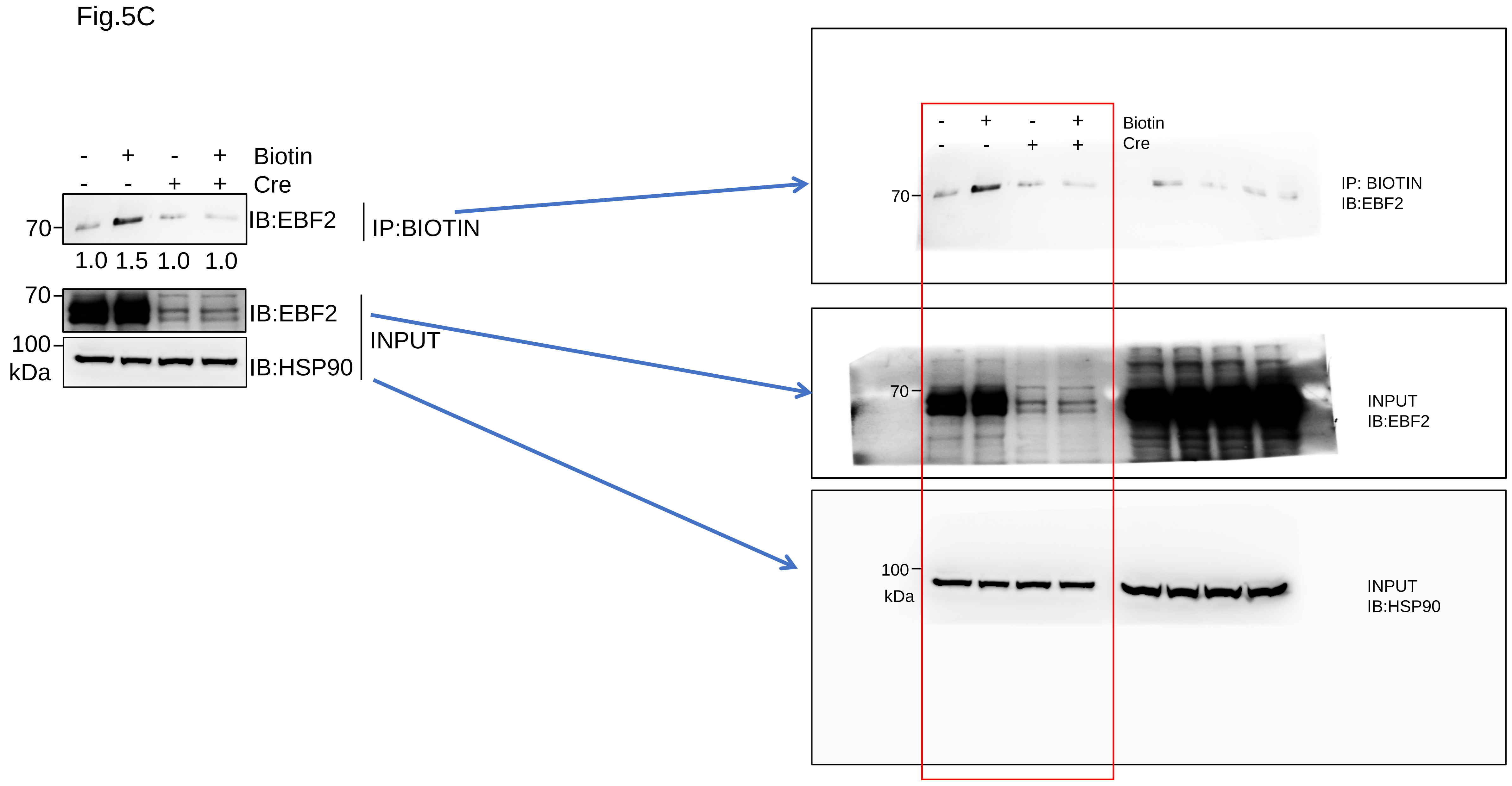

Figure 5

Fig.5D

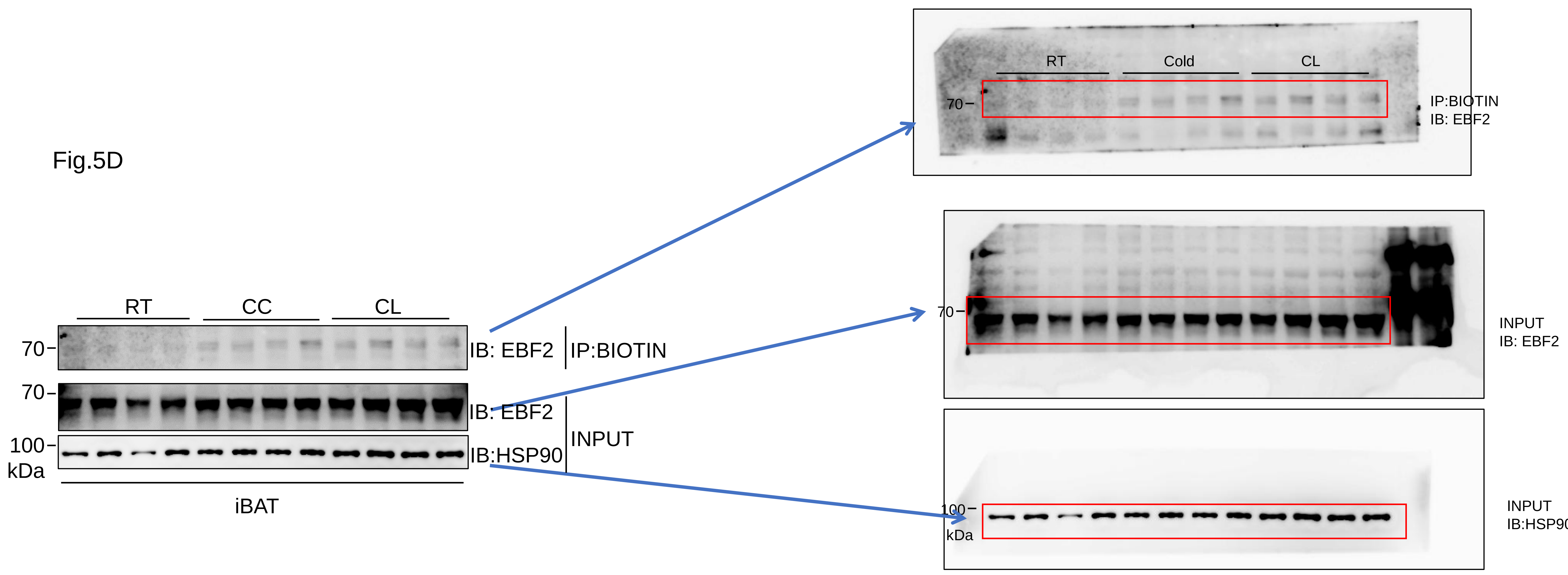

Fig.5E

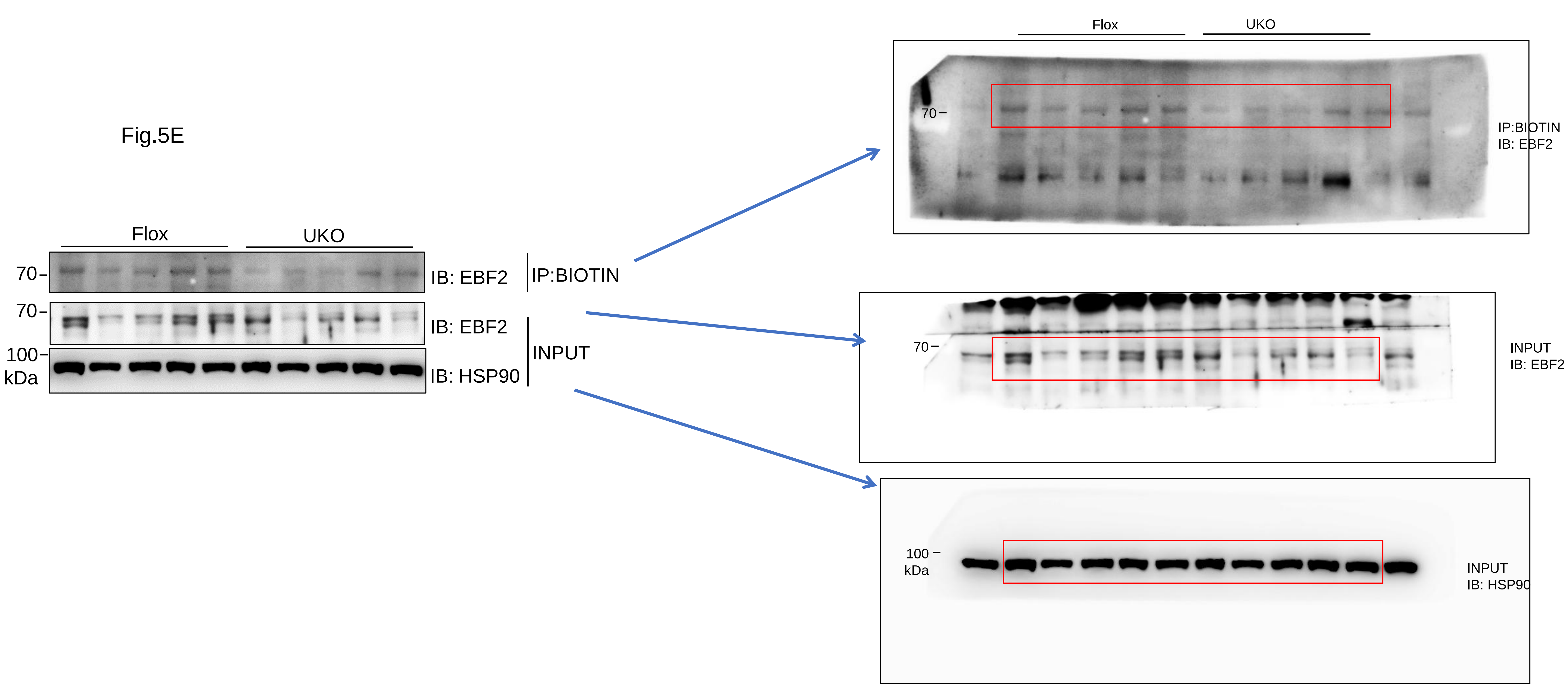

Figure 5

Fig.5F

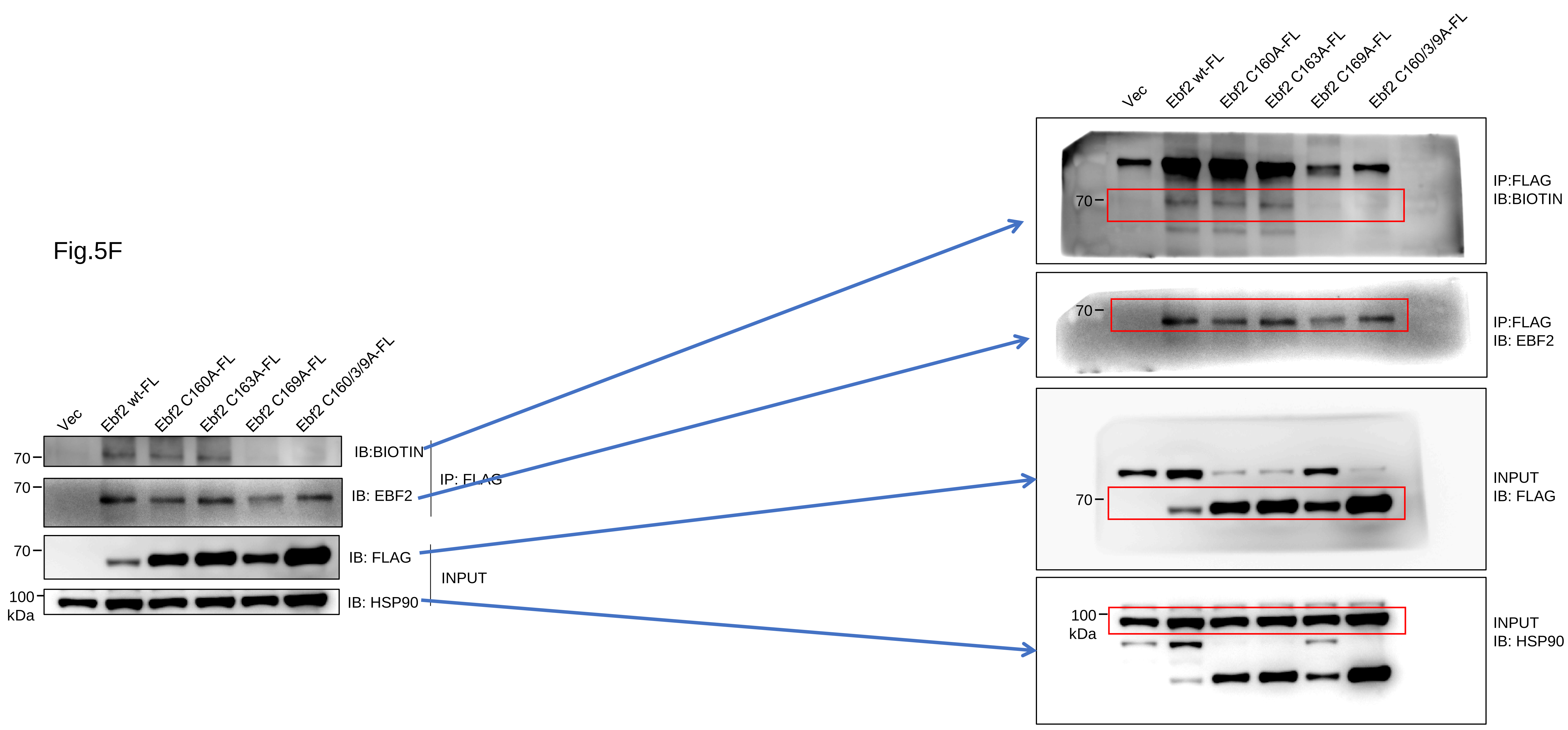

Figure 5

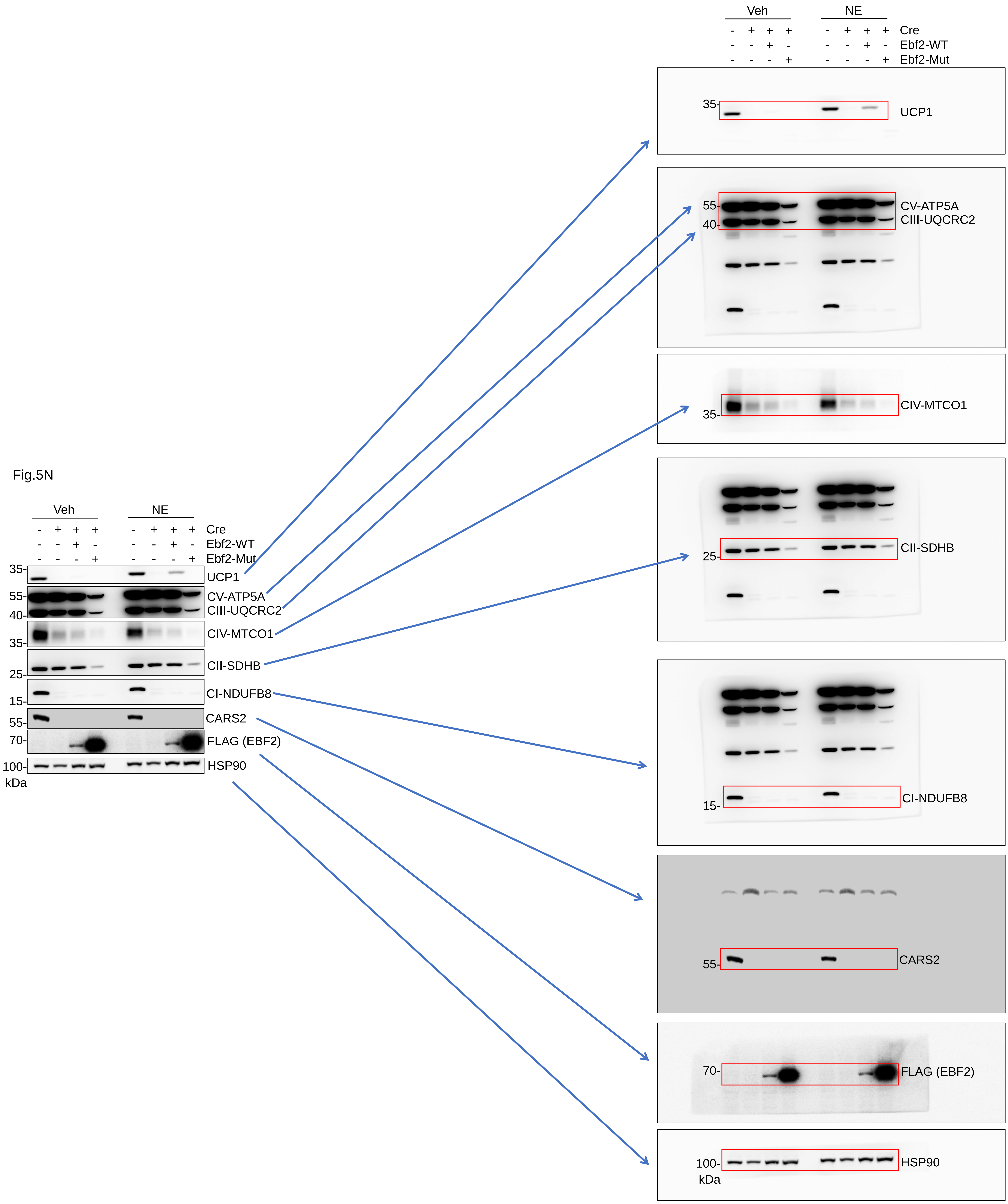
Fig.5N
Veh
NE
Cre
Ebf2-WT
Ebf2-Mut
UCP1
CV-ATP5A
CIII-UQCRC2
CIV-MTCO1
CII-SDHB
CI-NDUFB8
CARS2
FLAG (EBF2)
HSP90
kDa
35-
55-
40-
25-
15-
70-
100-

Figure 5

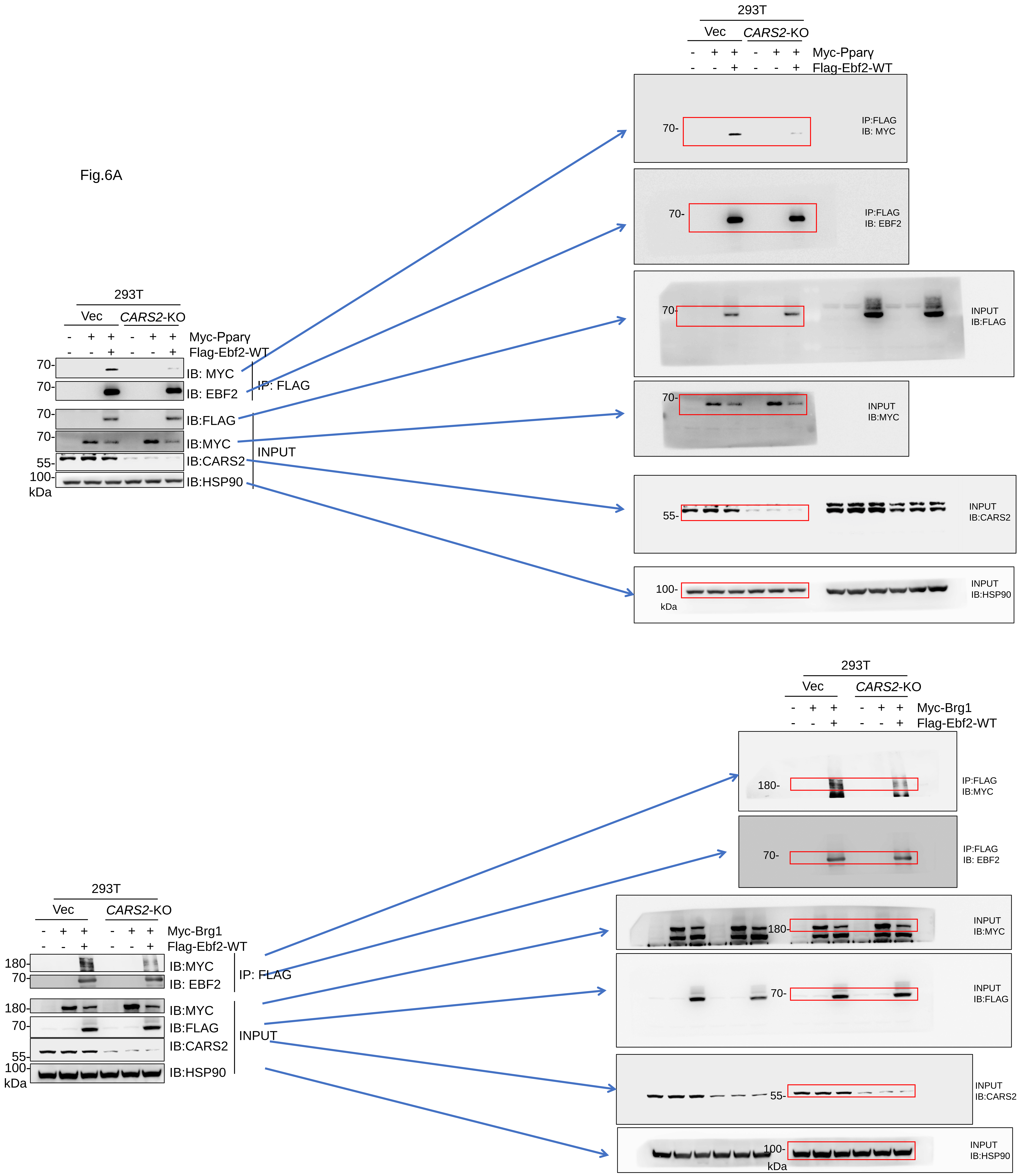
Fig.6A
293T
Vec
CARS2-KO
Myc-Pparγ
Flag-Ebf2-WT
IB: MYC
IB: EBF2
IP: FLAG
IB:FLAG
IB:MYC
IB:CARS2
IB:HSP90
INPUT
70-
55-
100-
kDa
IP:FLAG
IB: MYC
IP:FLAG
IB: EBF2
INPUT
IB:FLAG
INPUT
IB:MYC
INPUT
IB:CARS2
INPUT
IB:HSP90
Myc-Brg1
180-

Figure 6

Fig.6G

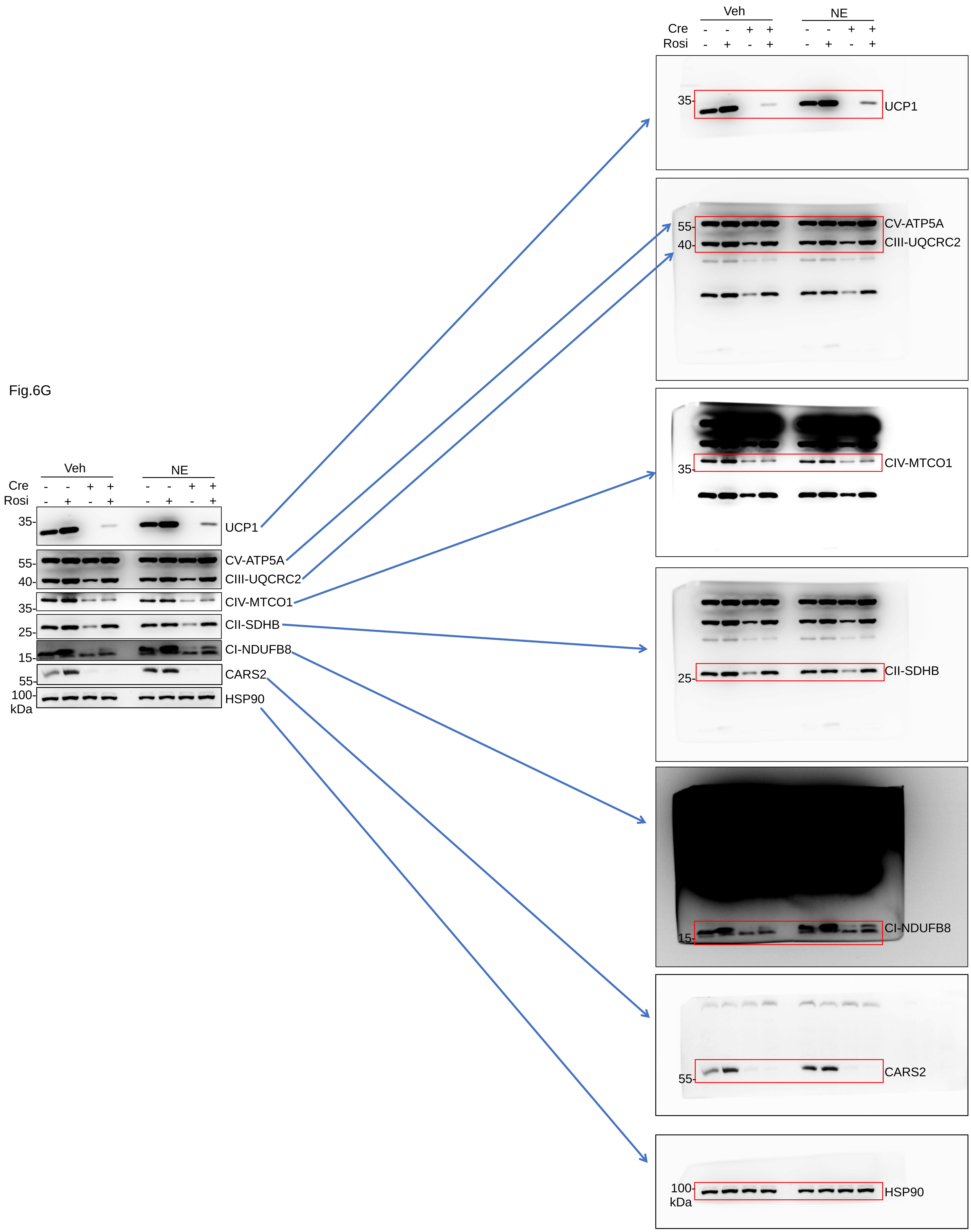

Figure 6

Fig.7C

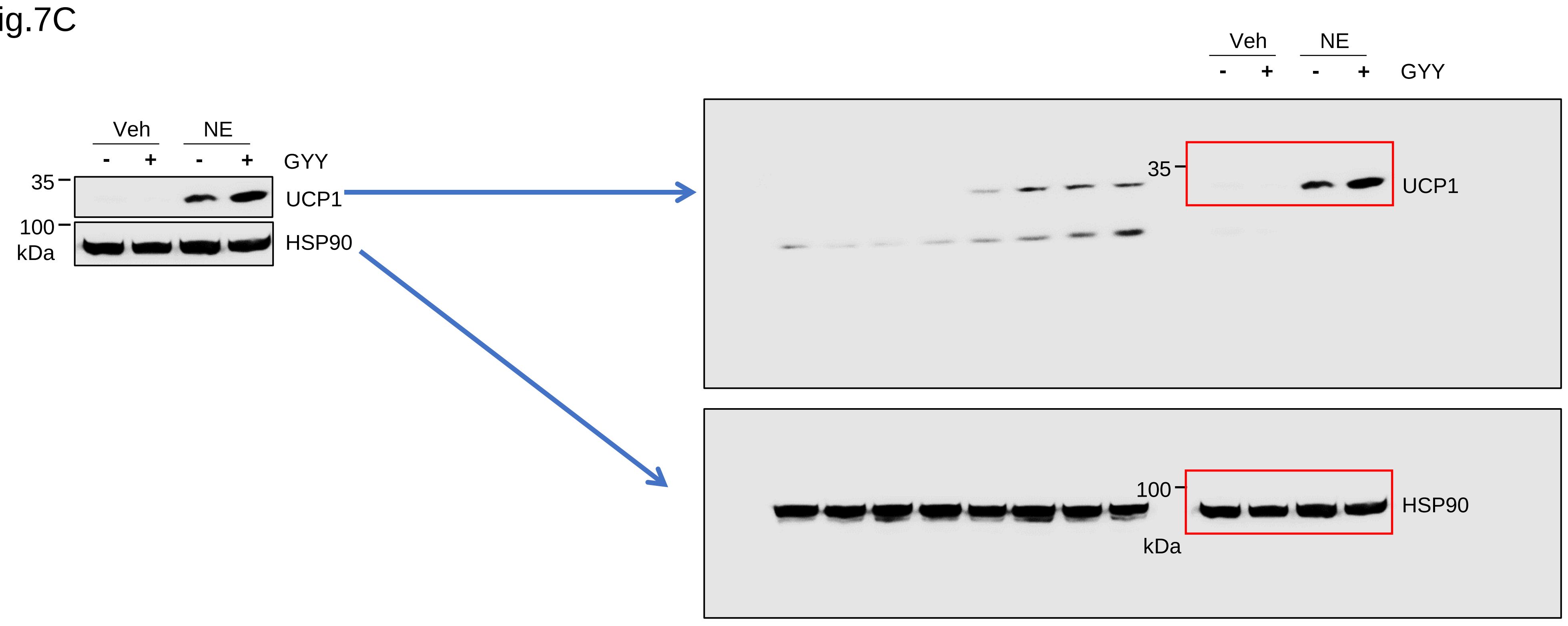

Fig.7F

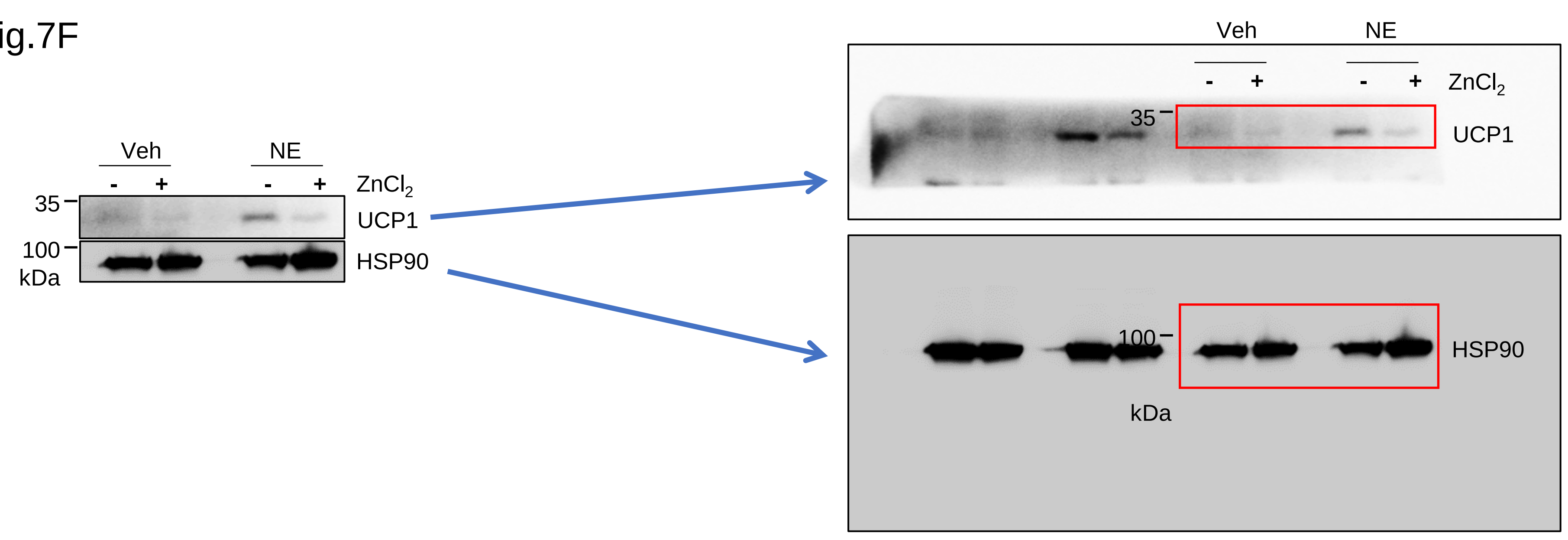

Fig.7G

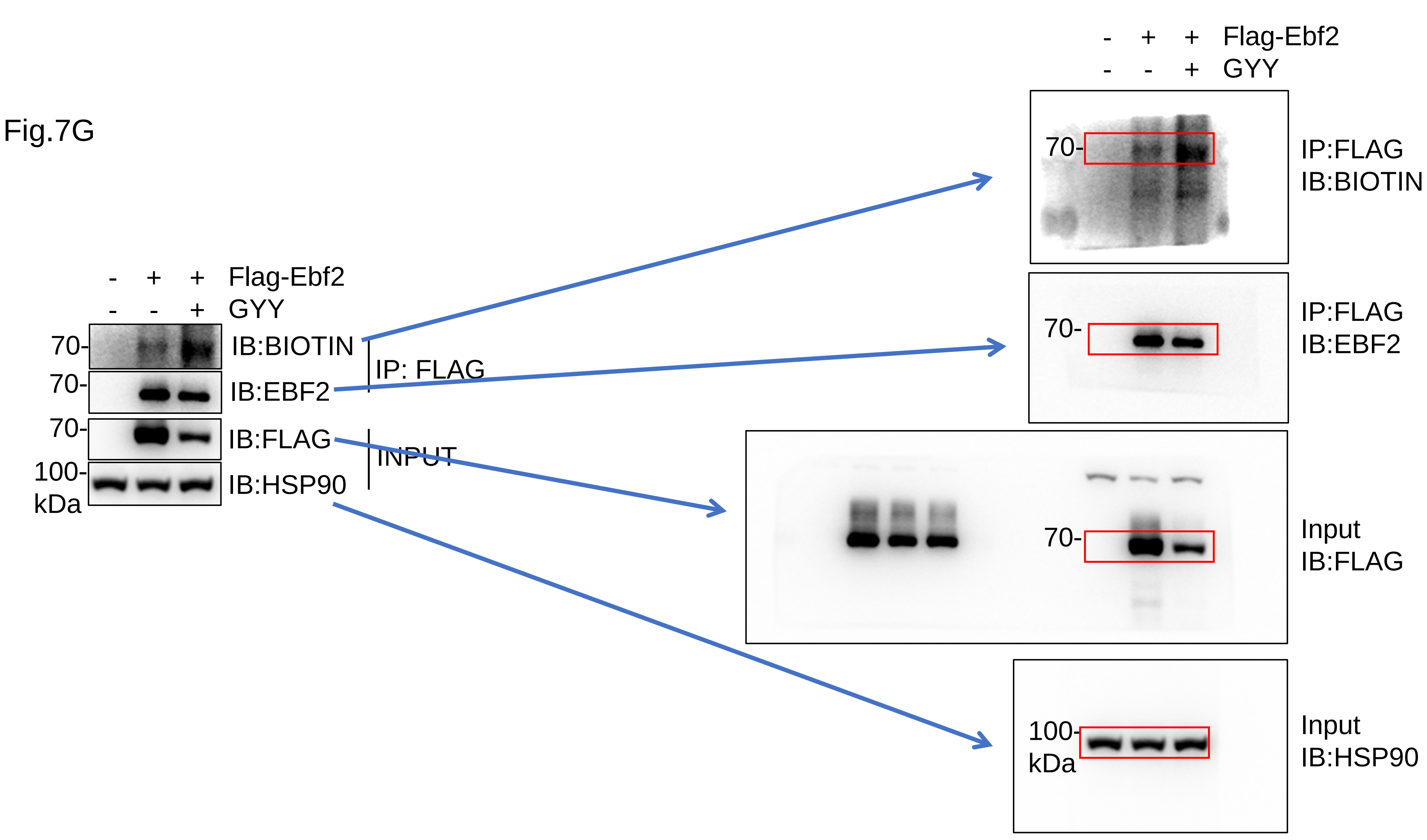

Figure 7

Fig.7H

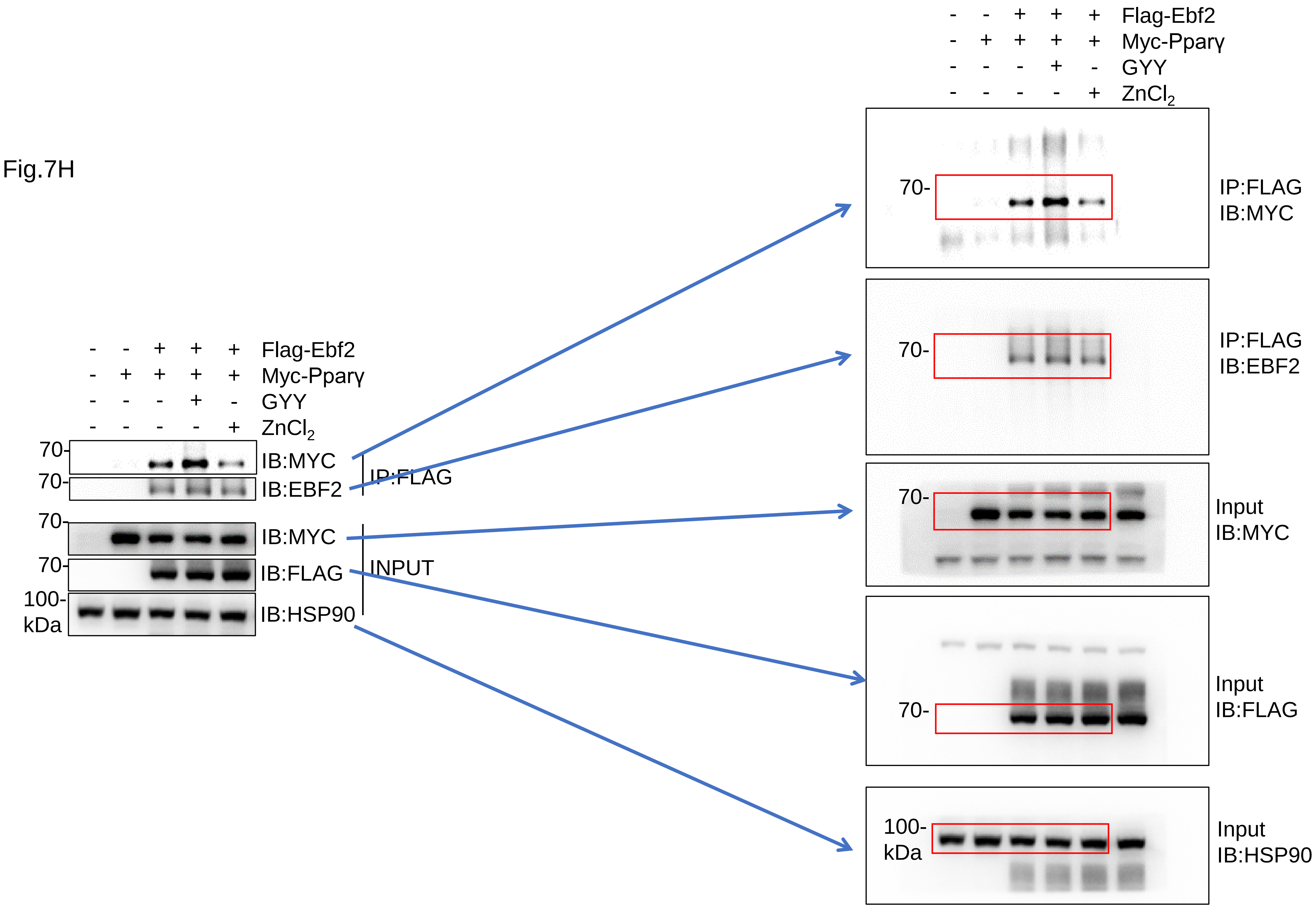

Fig.7I

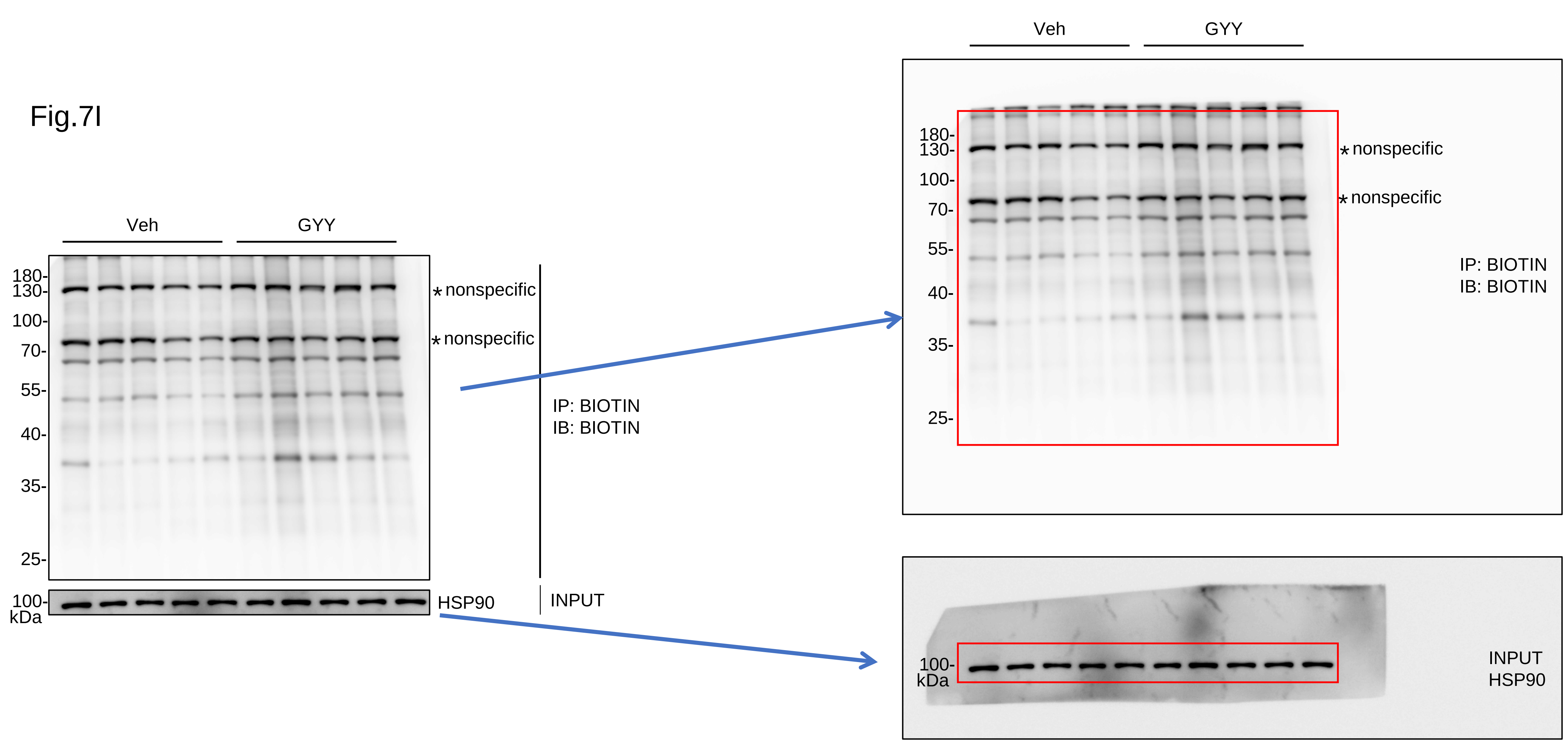

Figure 7

Fig.7P

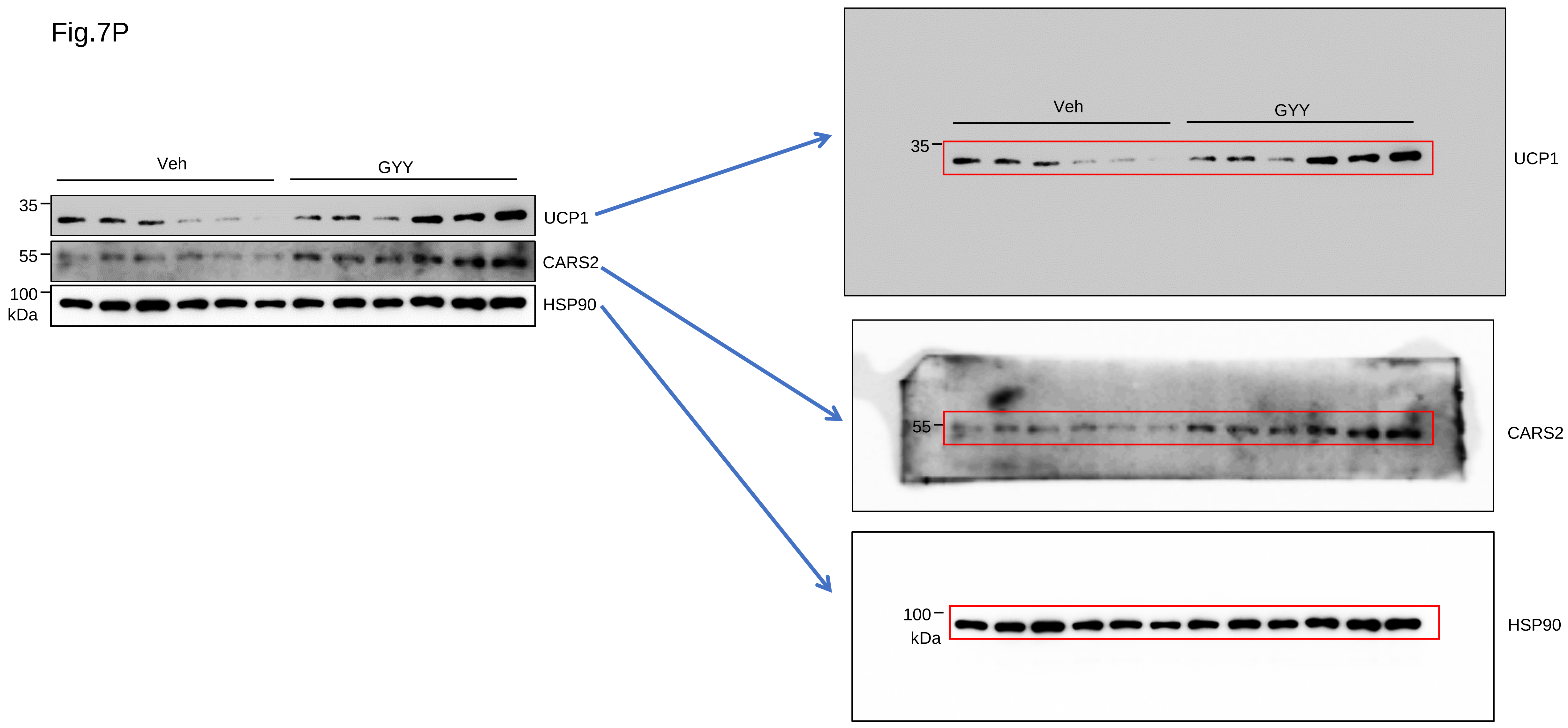

Fig.7Q

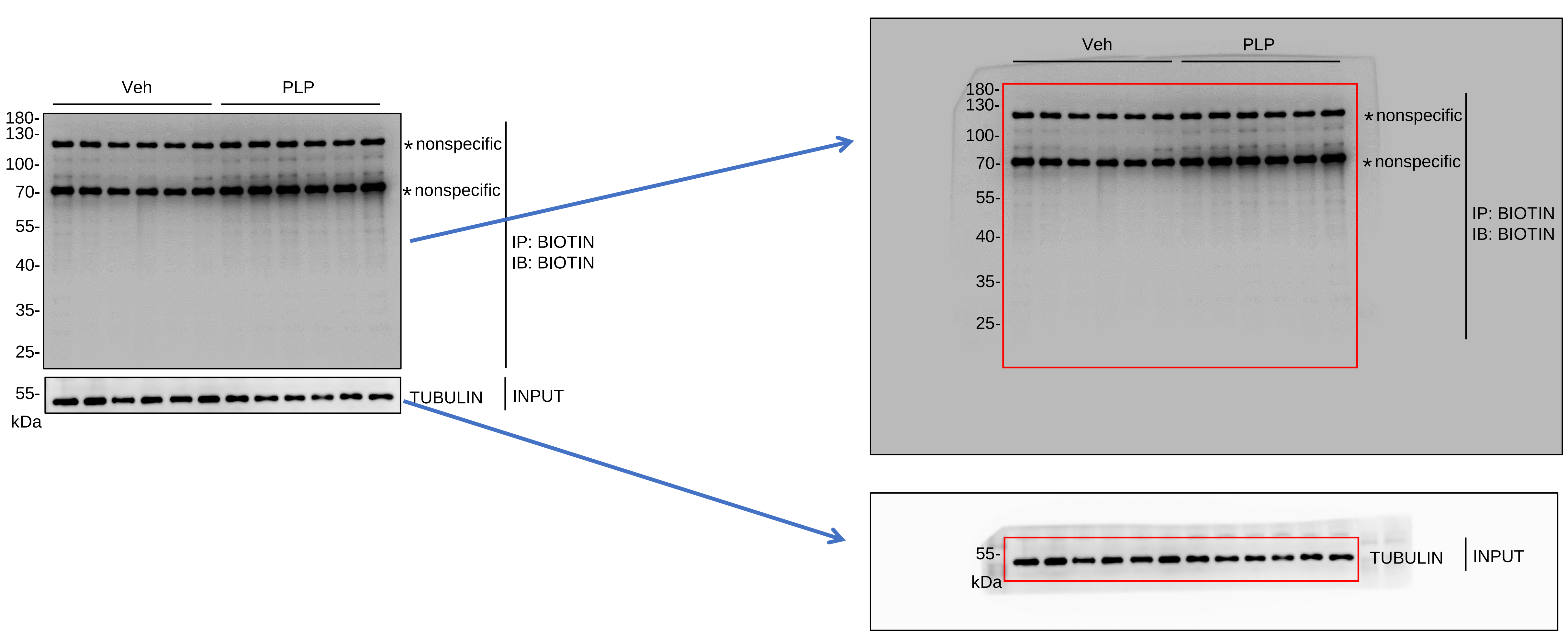

Fig.7X

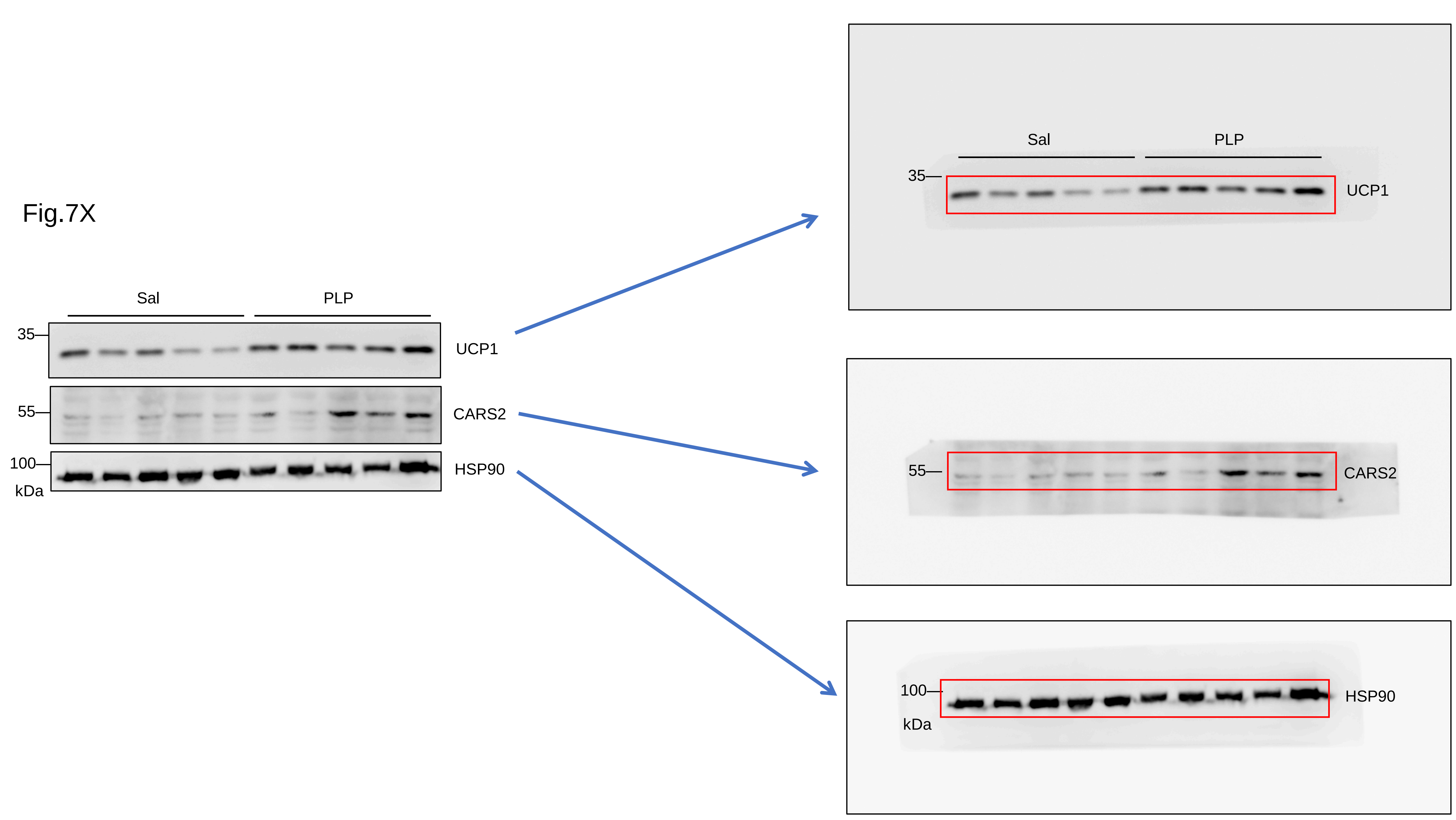

Figure 7

Fig.S1L

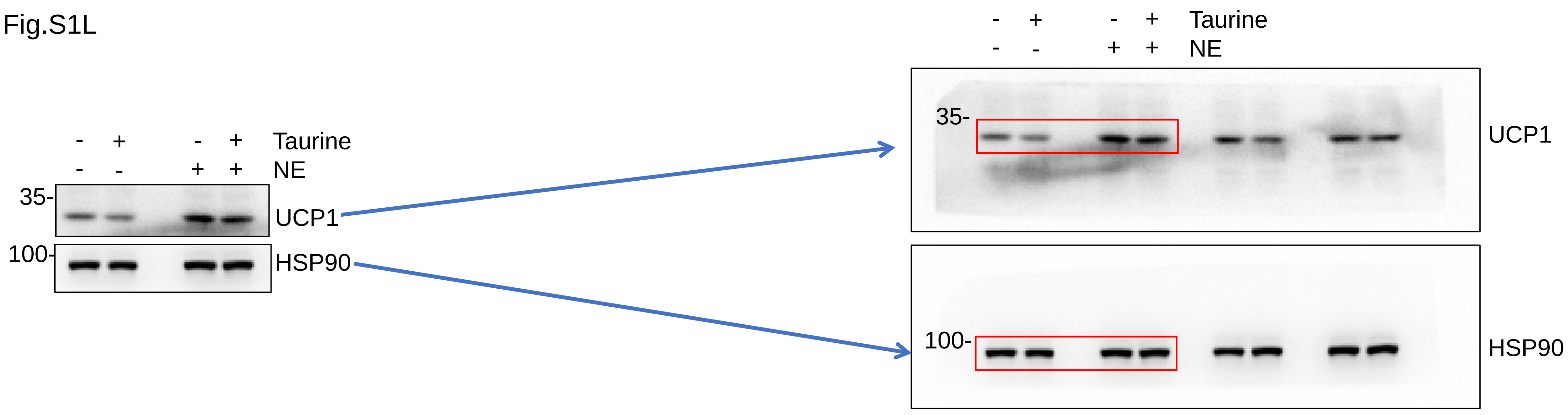

Fig.S1O

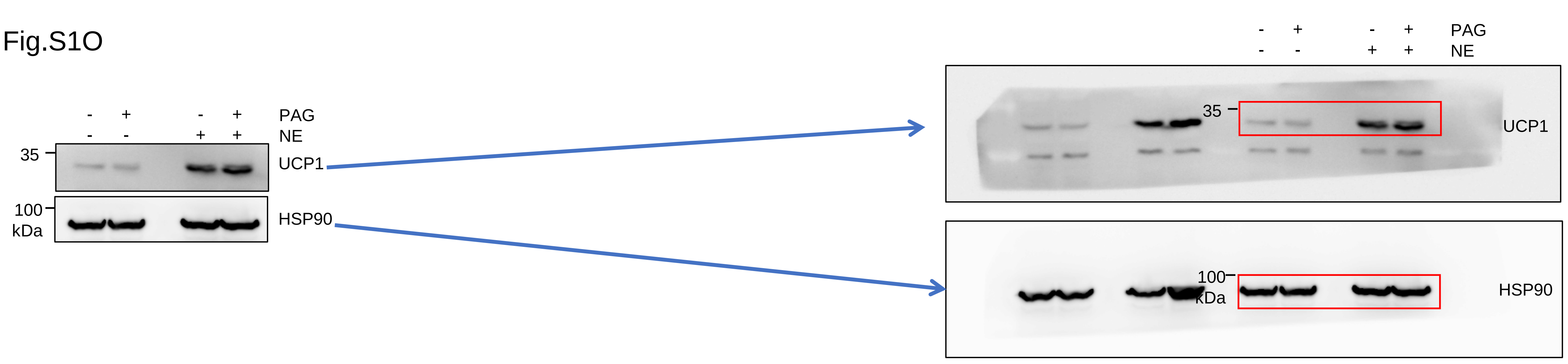

Figure S1

Fig.S6C

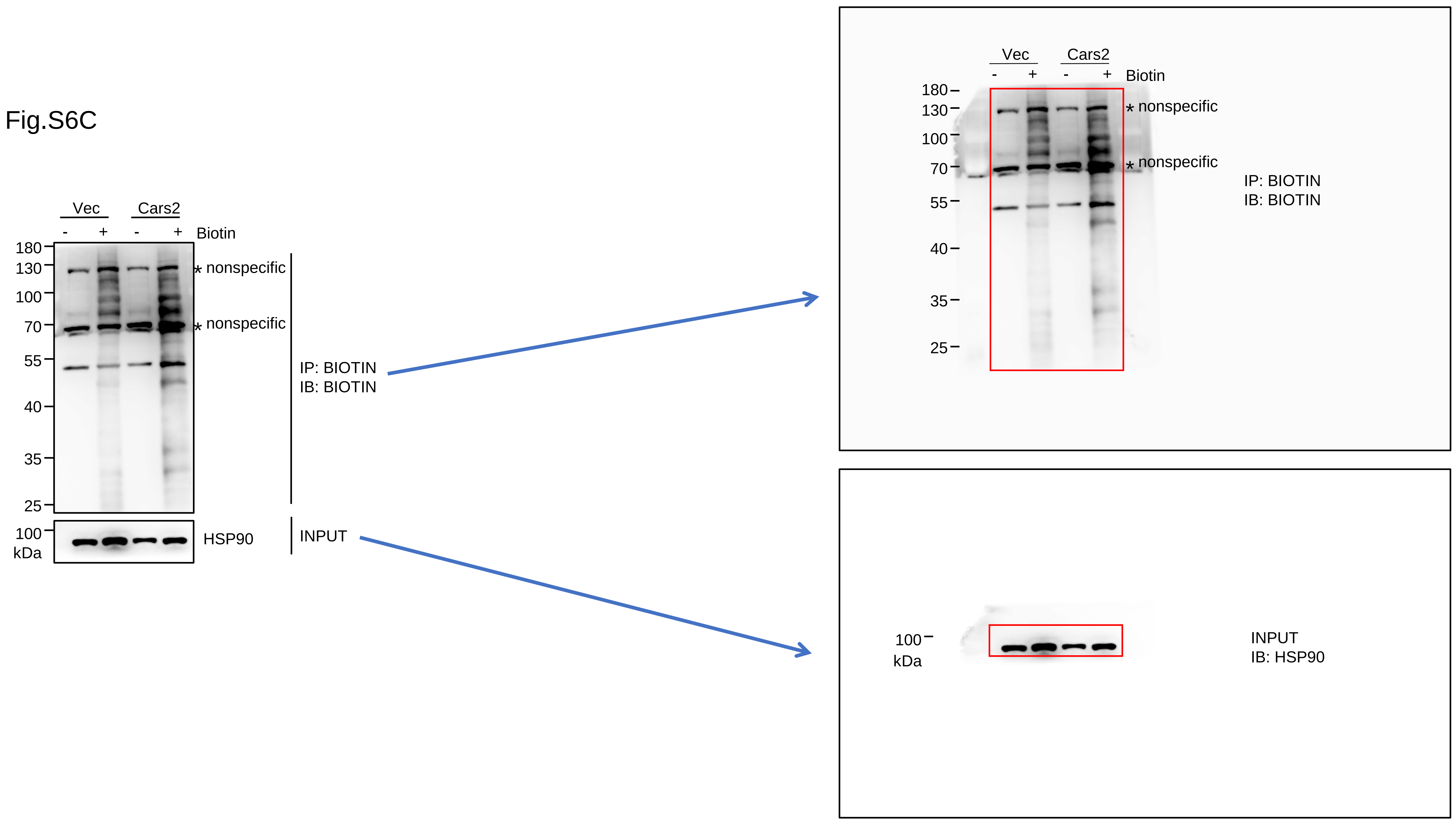

Fig.S6F

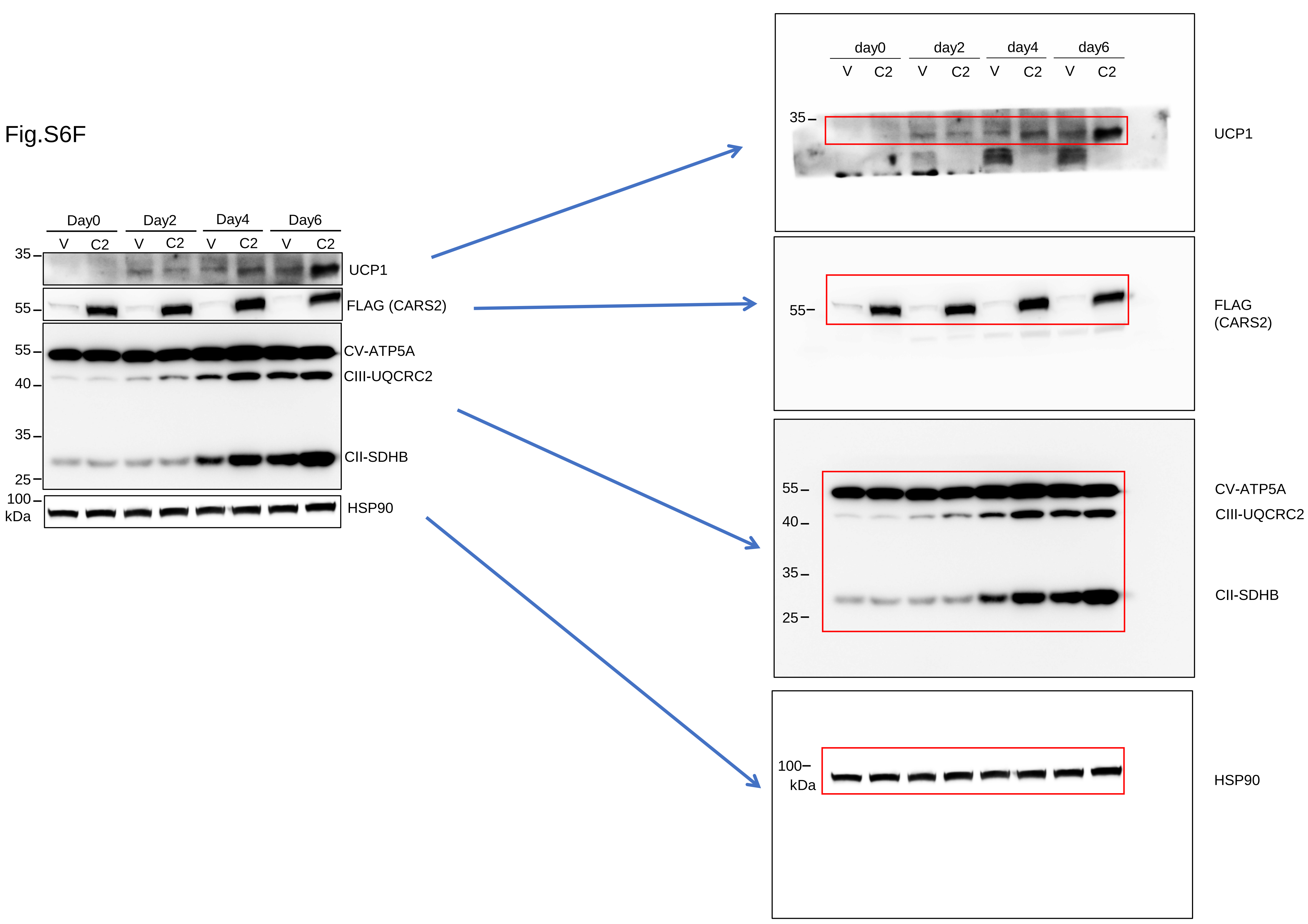

Figure S6

Fig.S6I

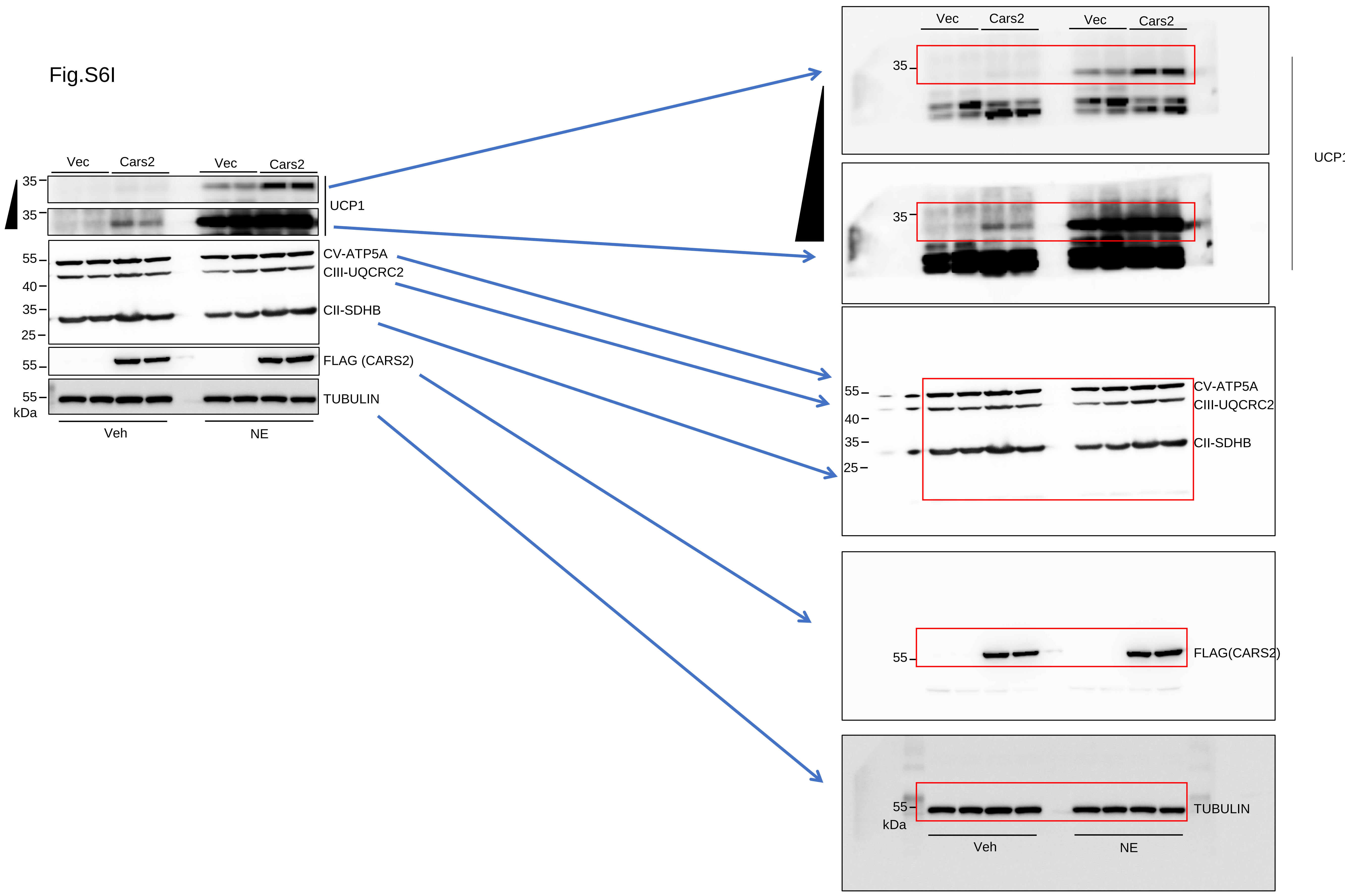

Figure S6

Fig.S7C

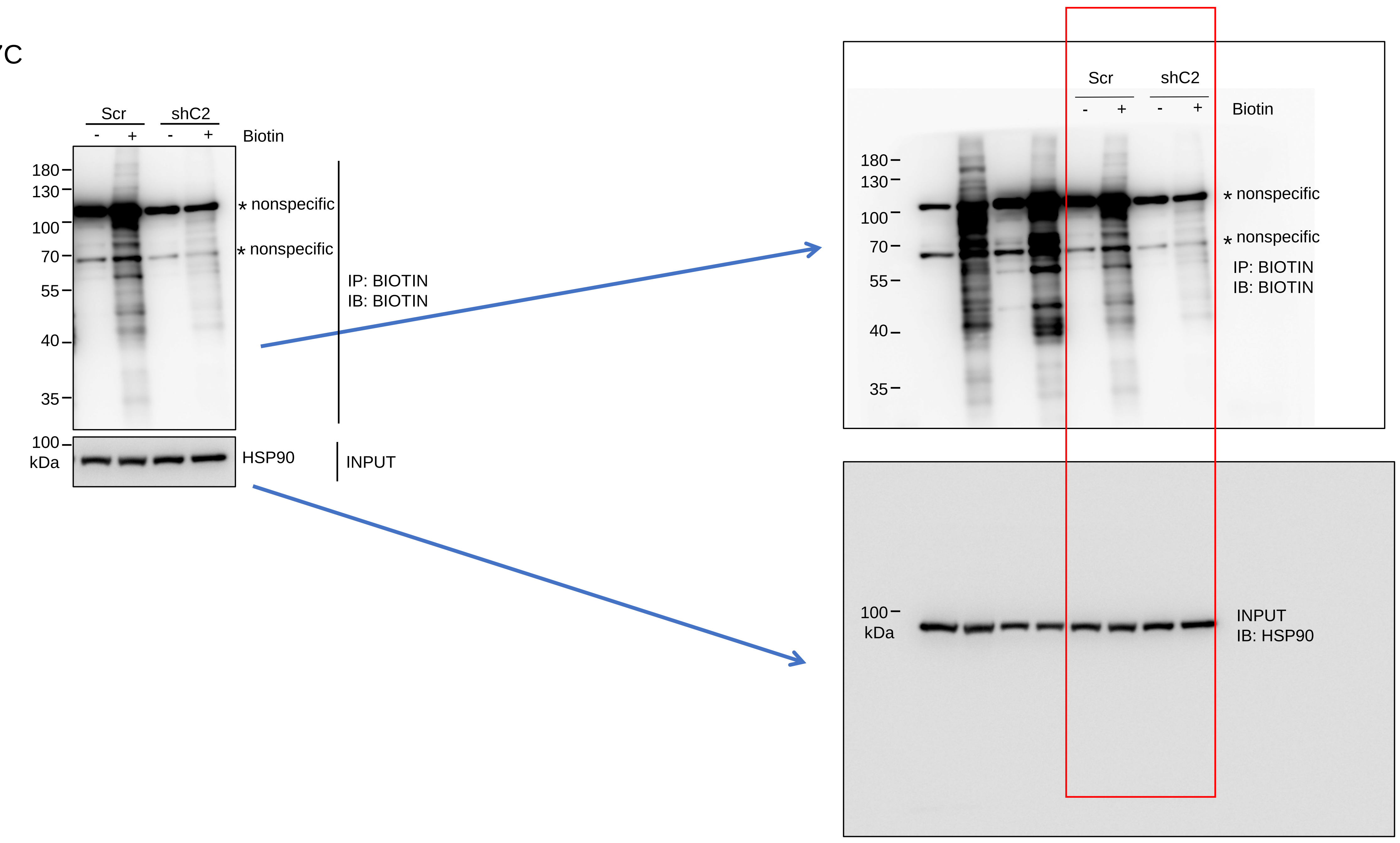

Fig.S7F

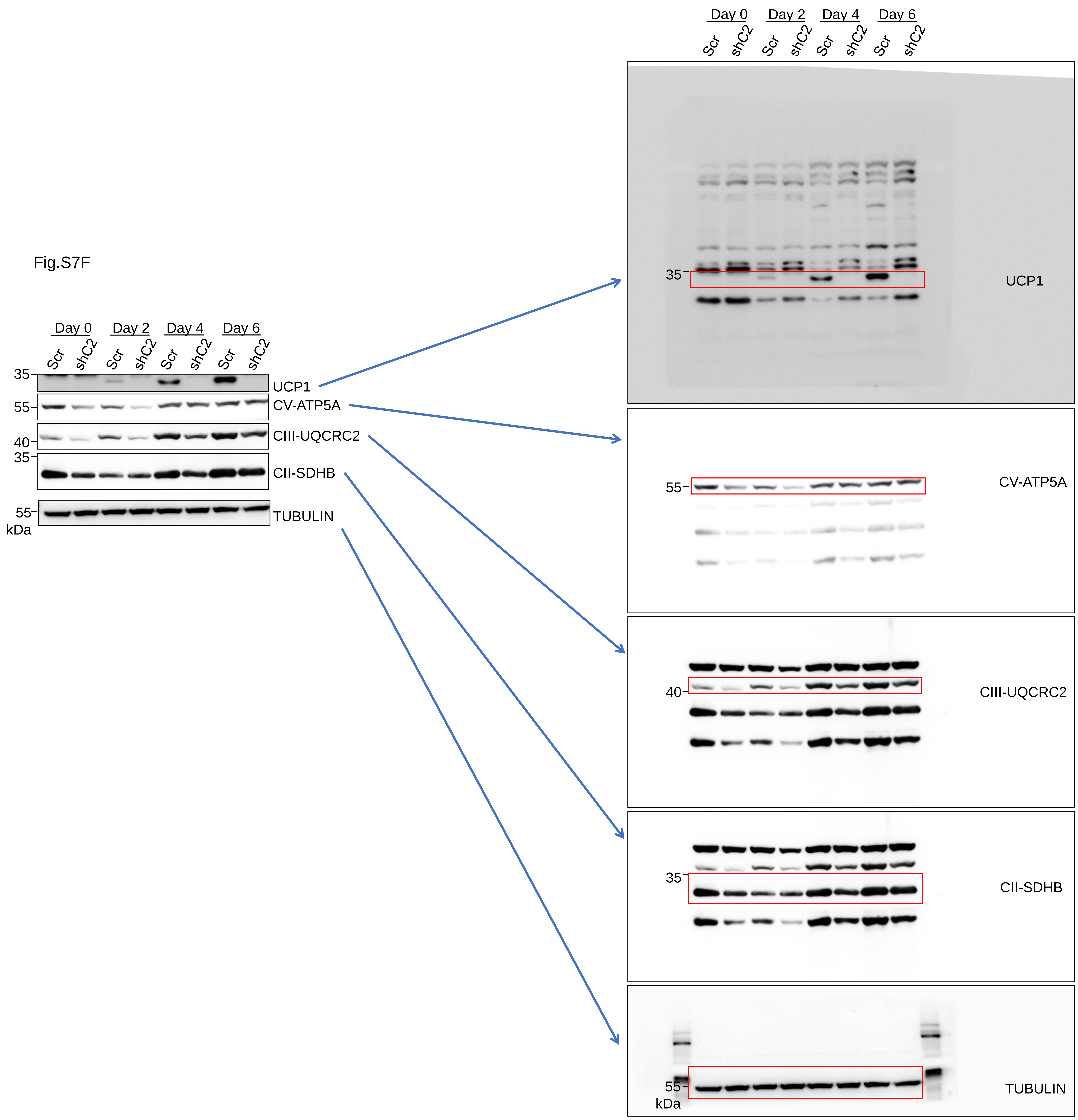

Figure S7

Fig.S7I

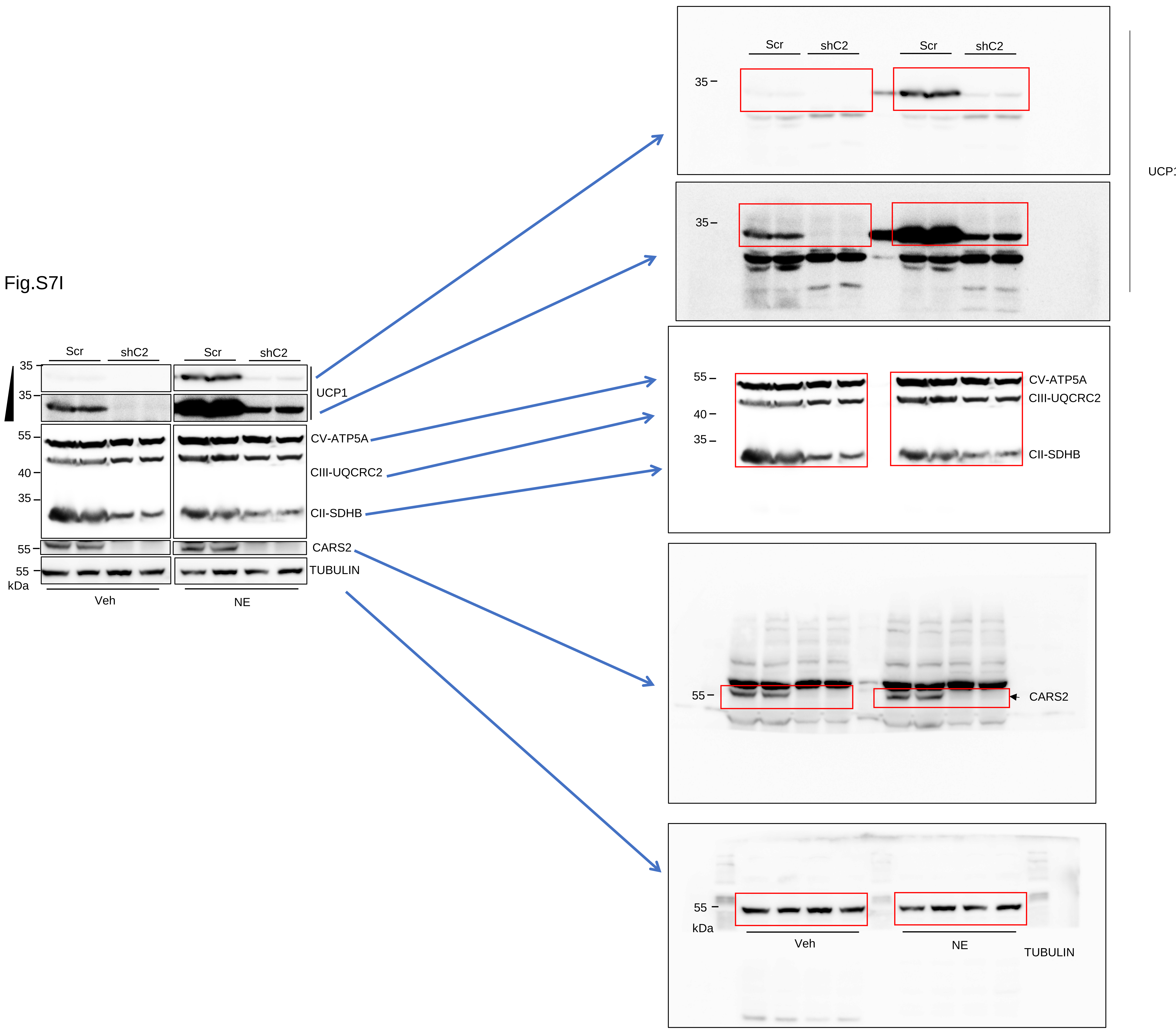

Figure S7

Fig.S8C

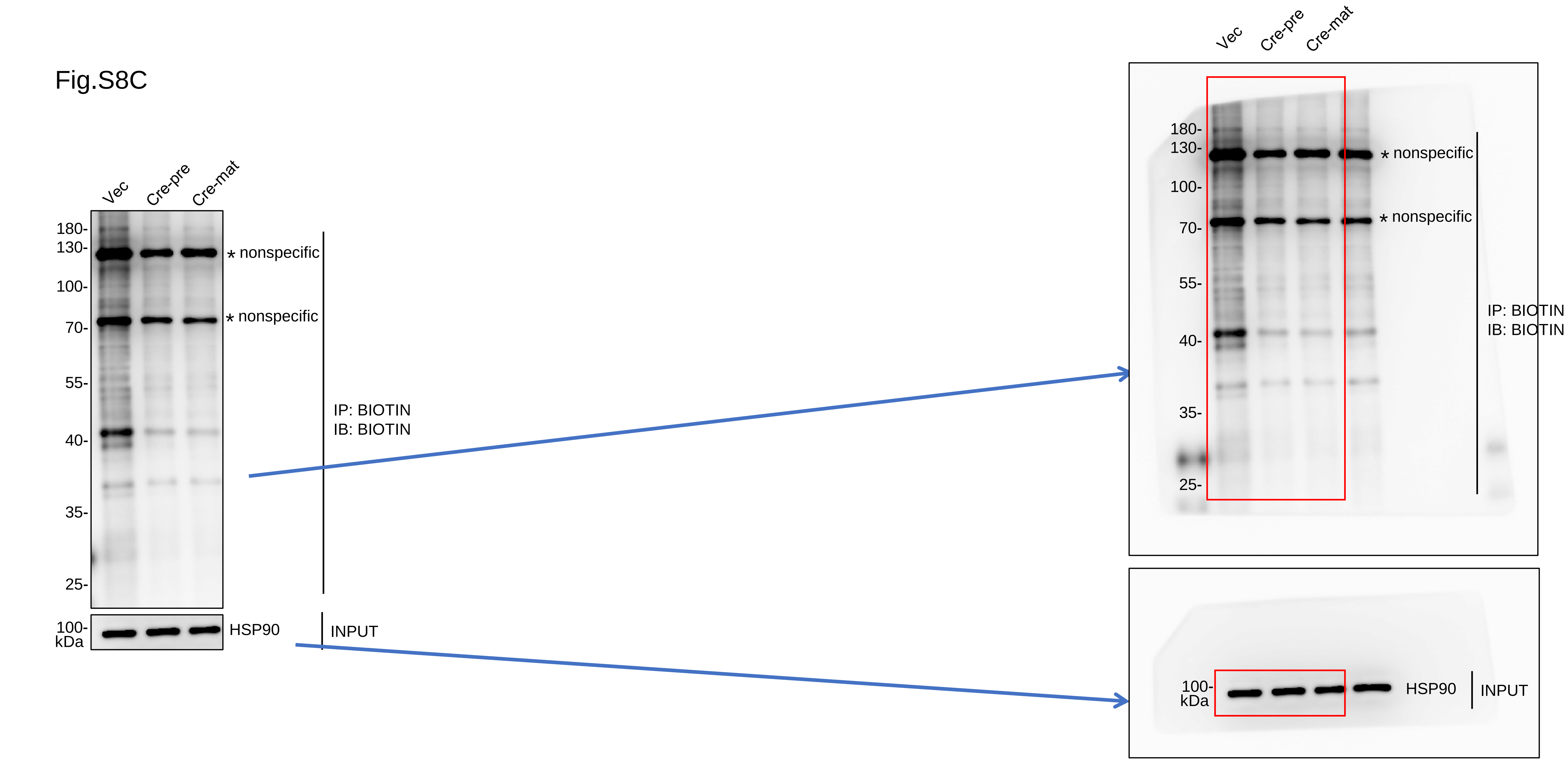

Fig.S8F

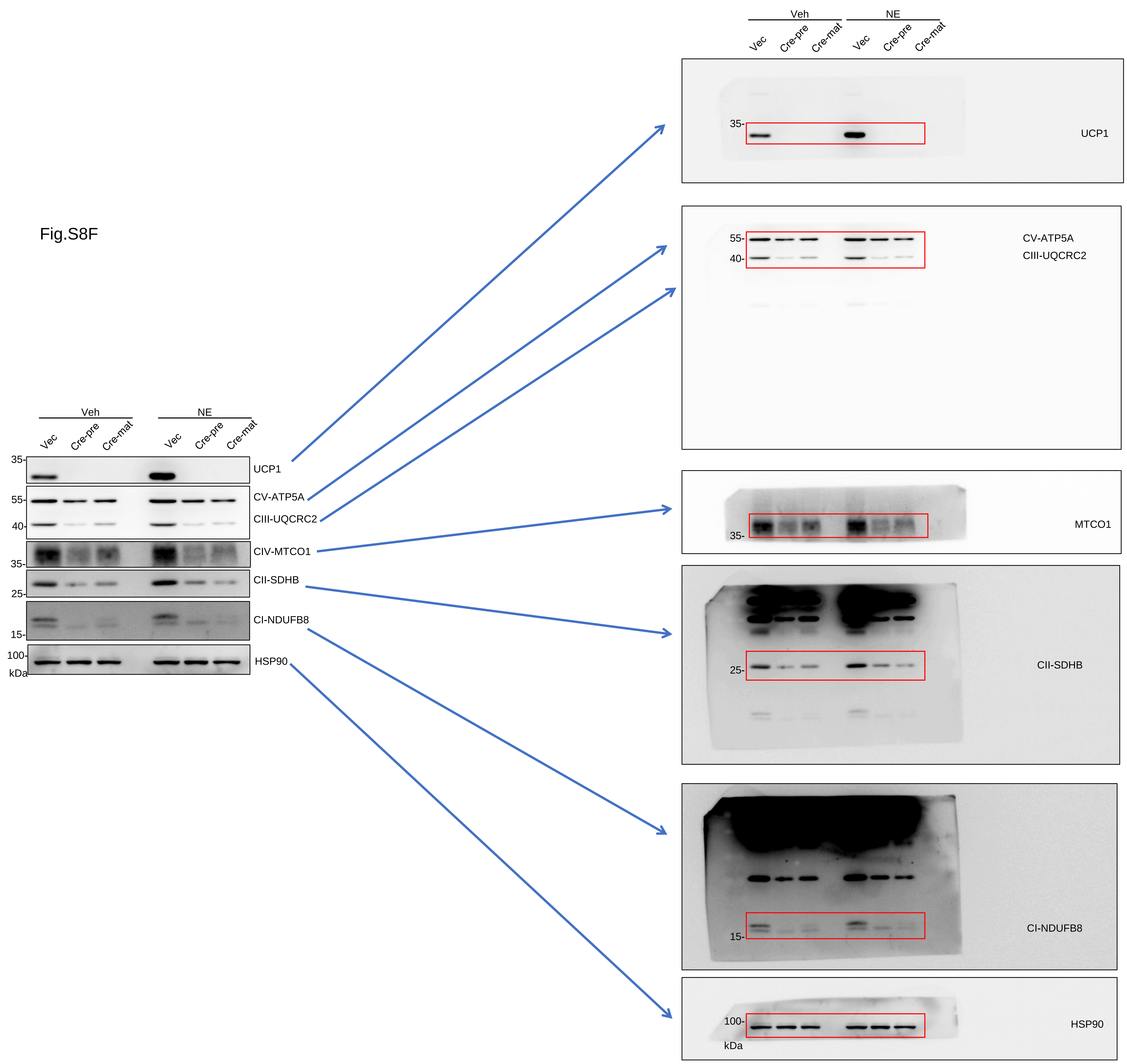

Figure S8

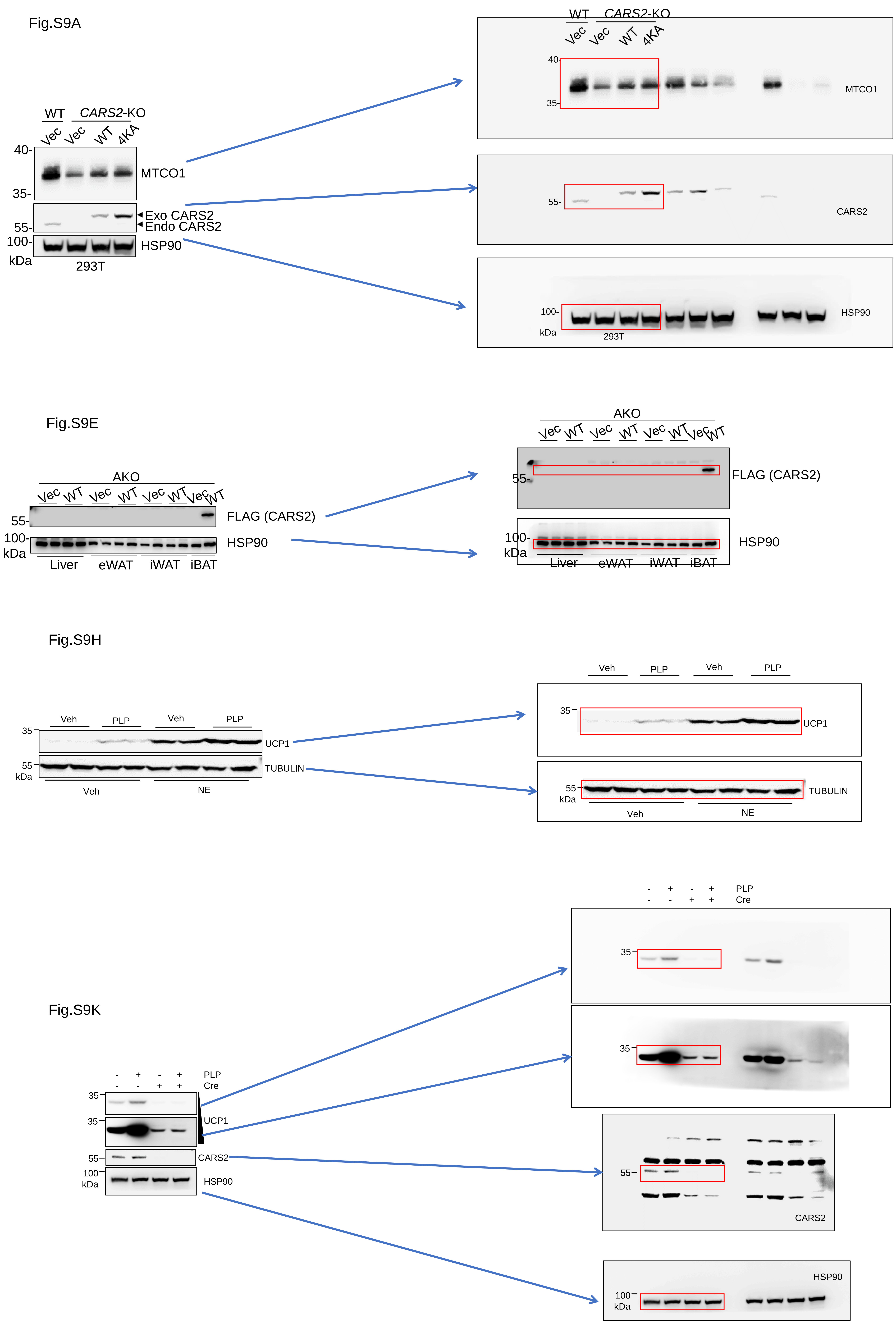
Fig.S9A
WT
CARS2-KO
Vec
Vec
WT
4KA
40-
35-
MTCO1
Exo CARS2
Endo CARS2
55-
100-
HSP90
kDa
293T
CARS2
Fig.S9E
AKO
Vec
WT
55-
FLAG (CARS2)
HSP90
Liver
eWAT
iWAT
iBAT
Fig.S9H
Veh
PLP
35
UCP1
55
TUBULIN
kDa
NE
Fig.S9K
PLP
Cre
CARS2

Figure S9

Fig.S10B

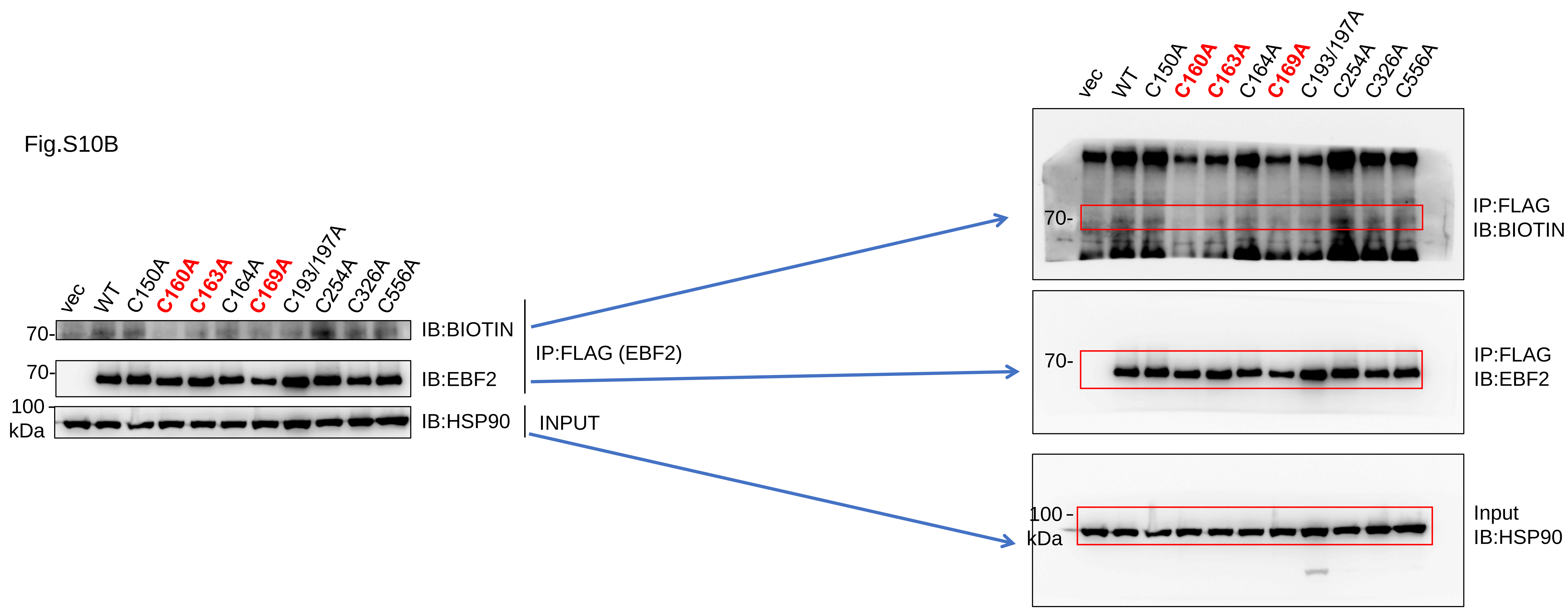

Figure S10

Fig.S11A

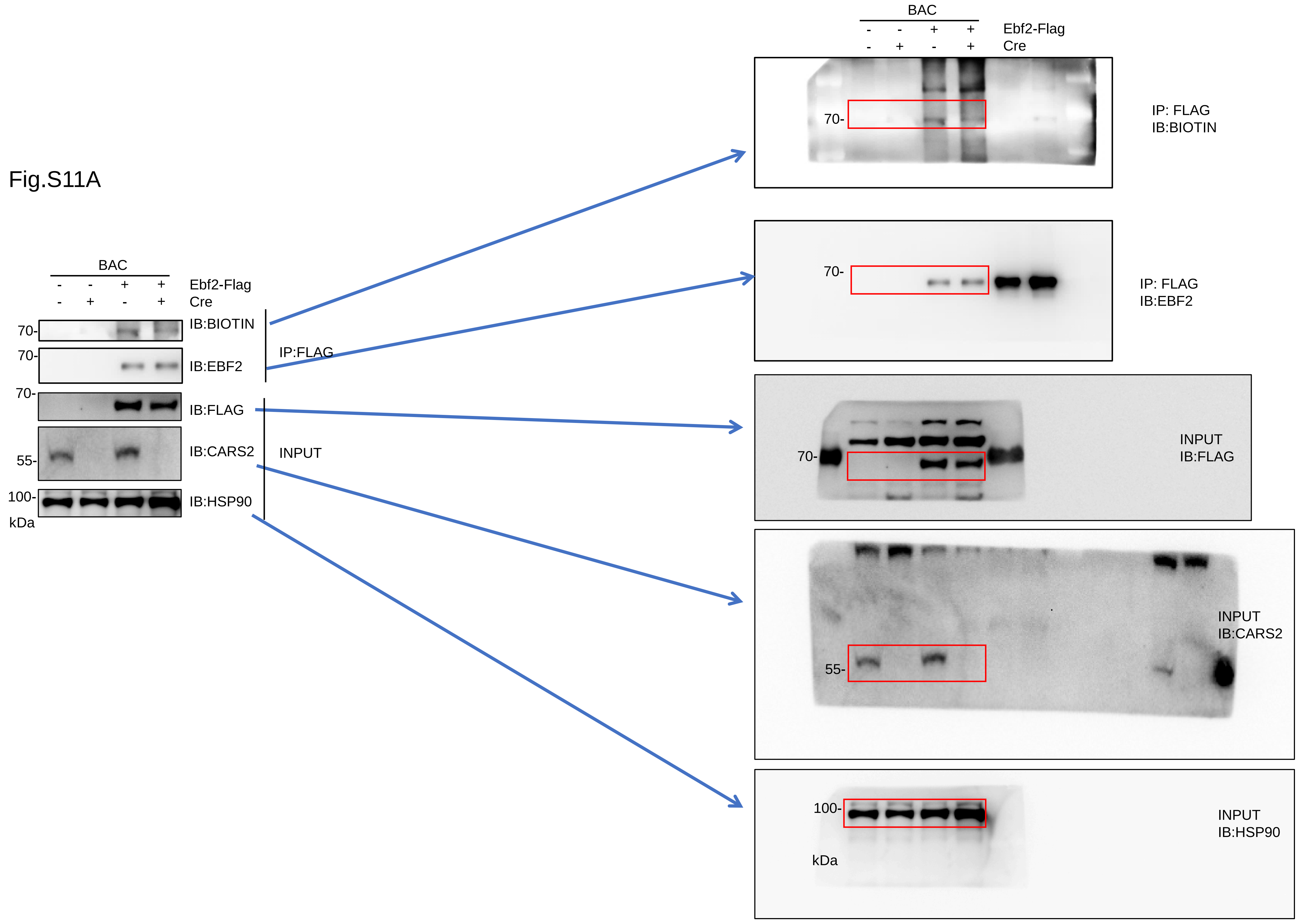

Fig.S11D

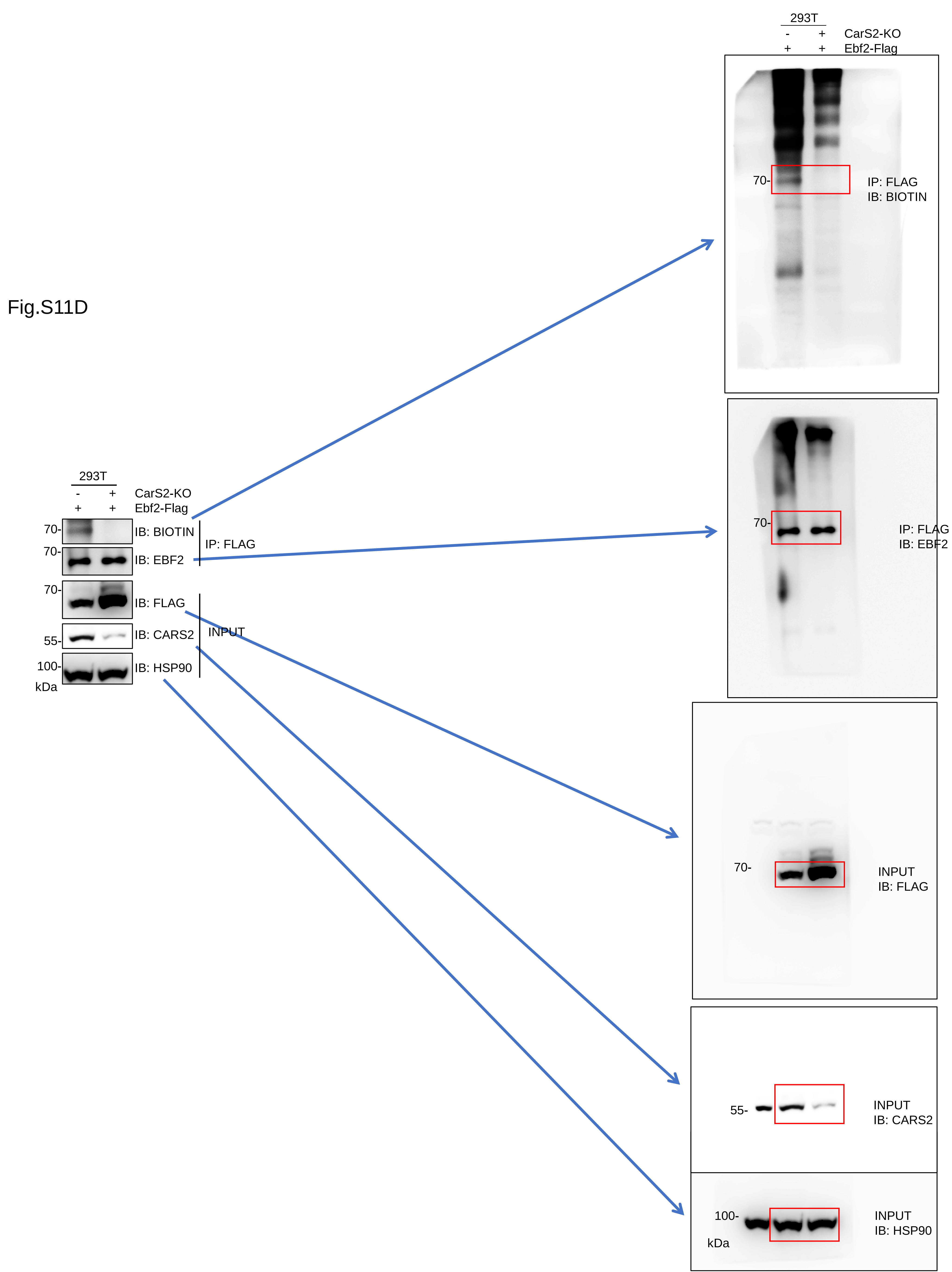

Figure S11

Fig.S11G

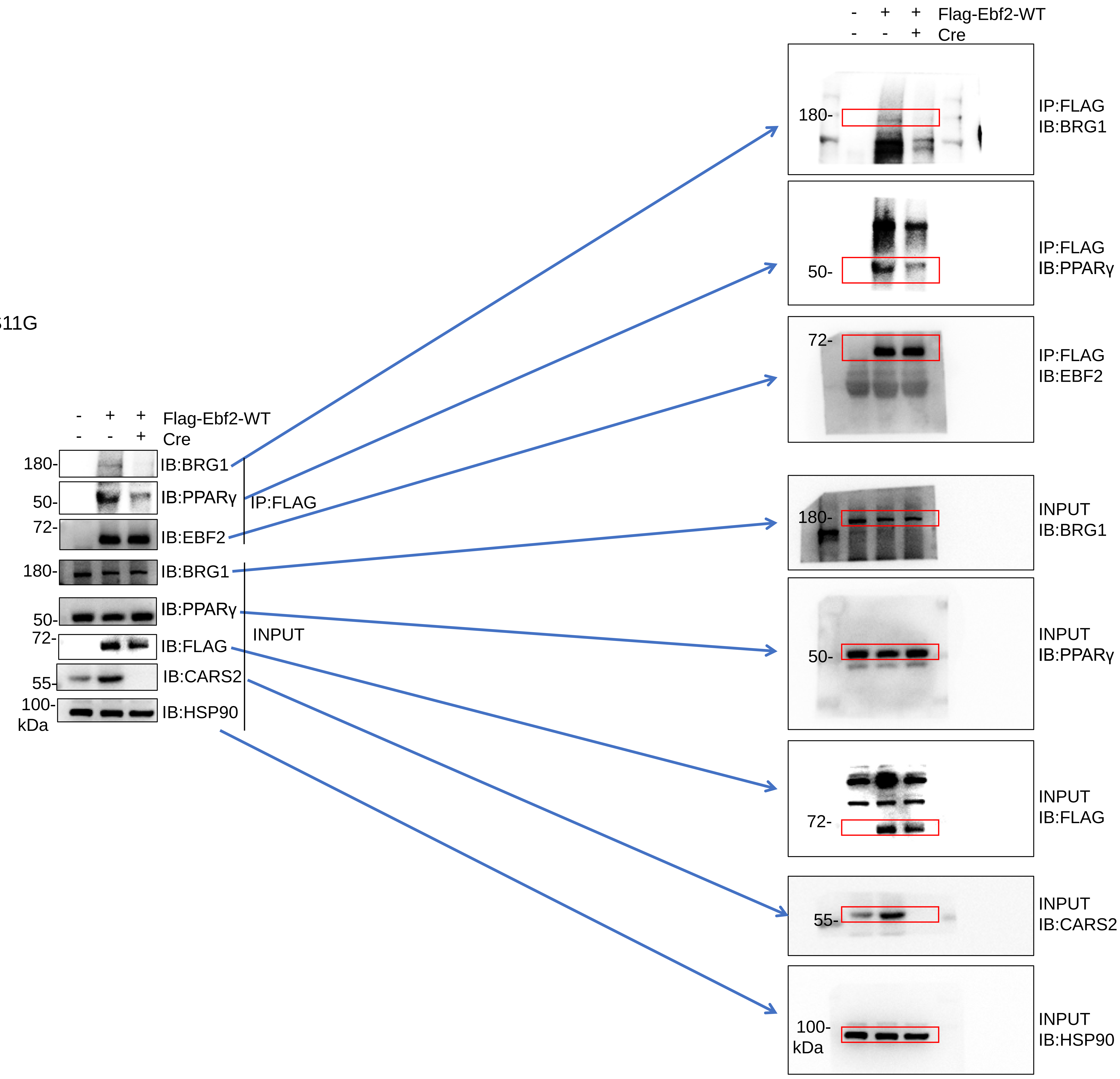

Figure S11

Fig.S11I

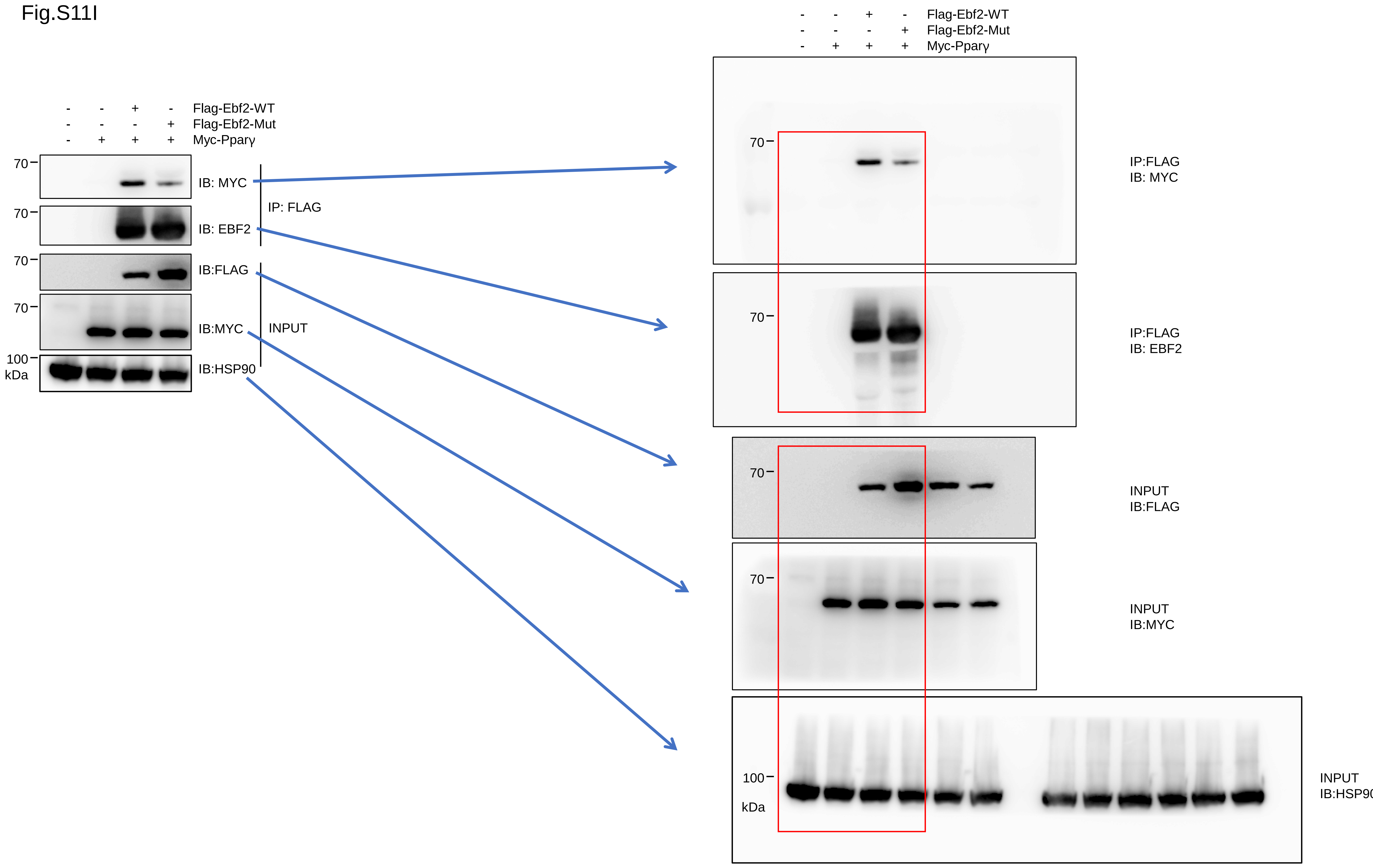

Fig.S11K

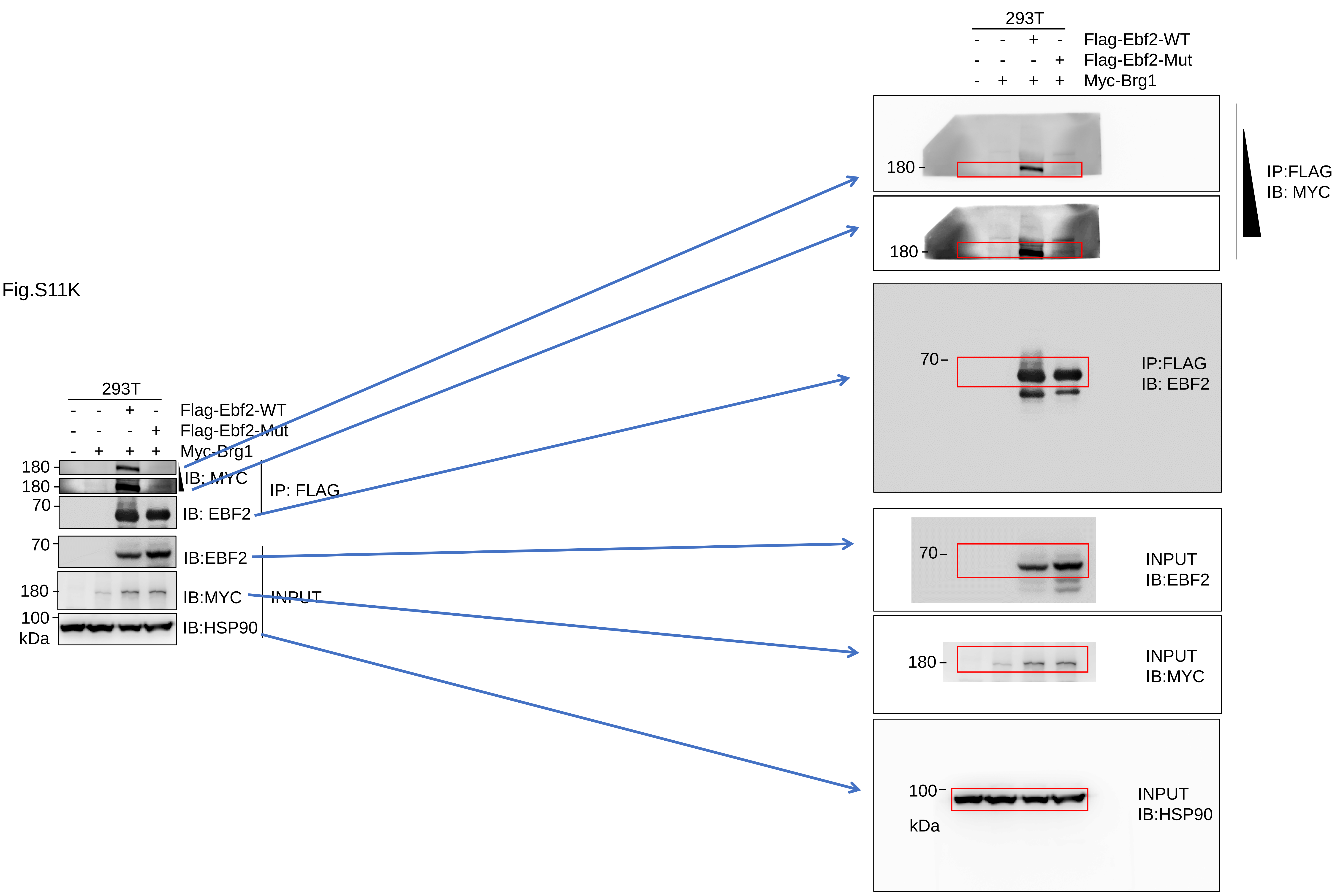

Figure S11

Figure 1 I

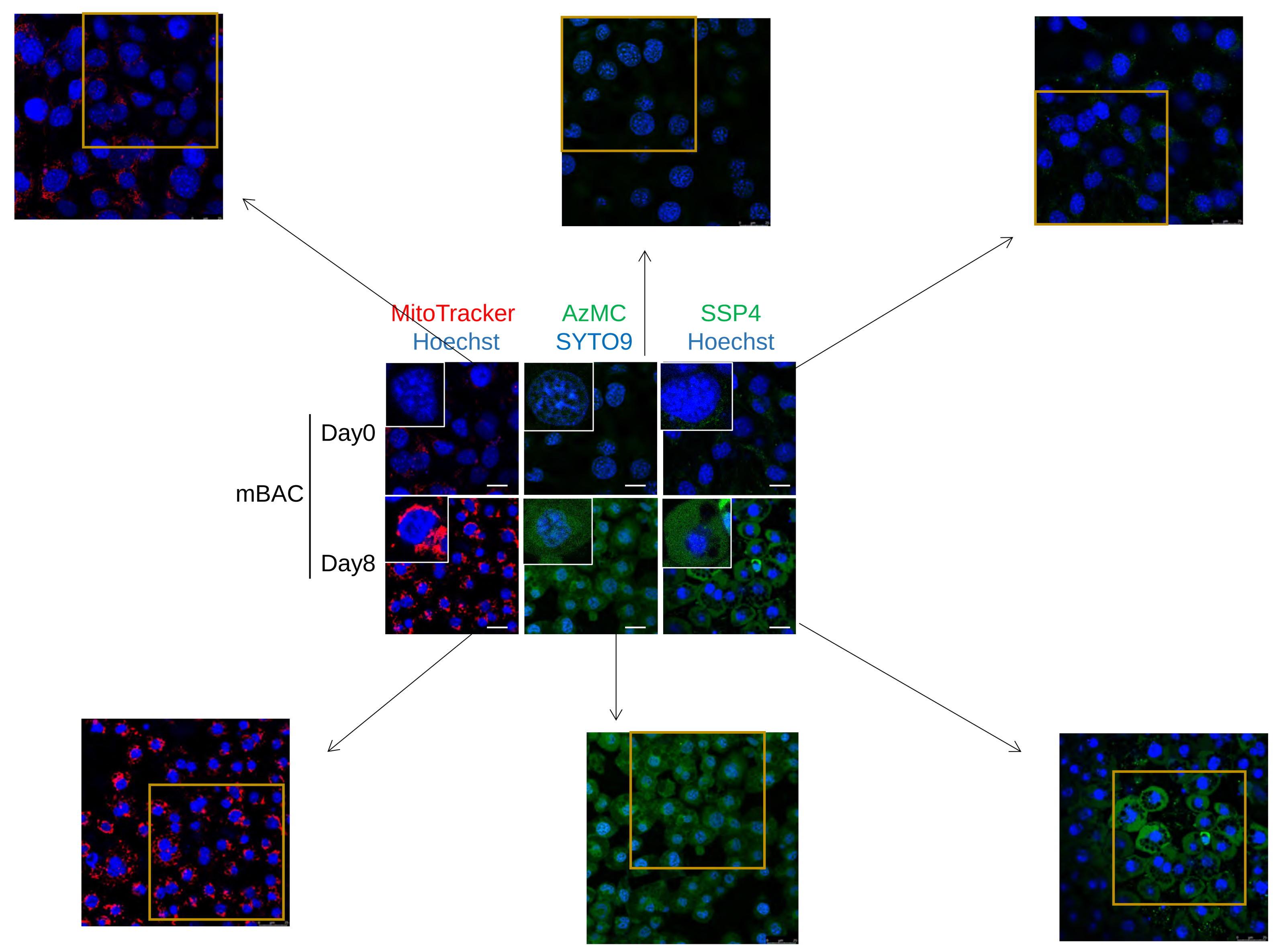
MitoTracker
Hoechst
AzMC
SYTO9
SSP4
Hoechst
mBAC
Day0
Day8

Figure 1K

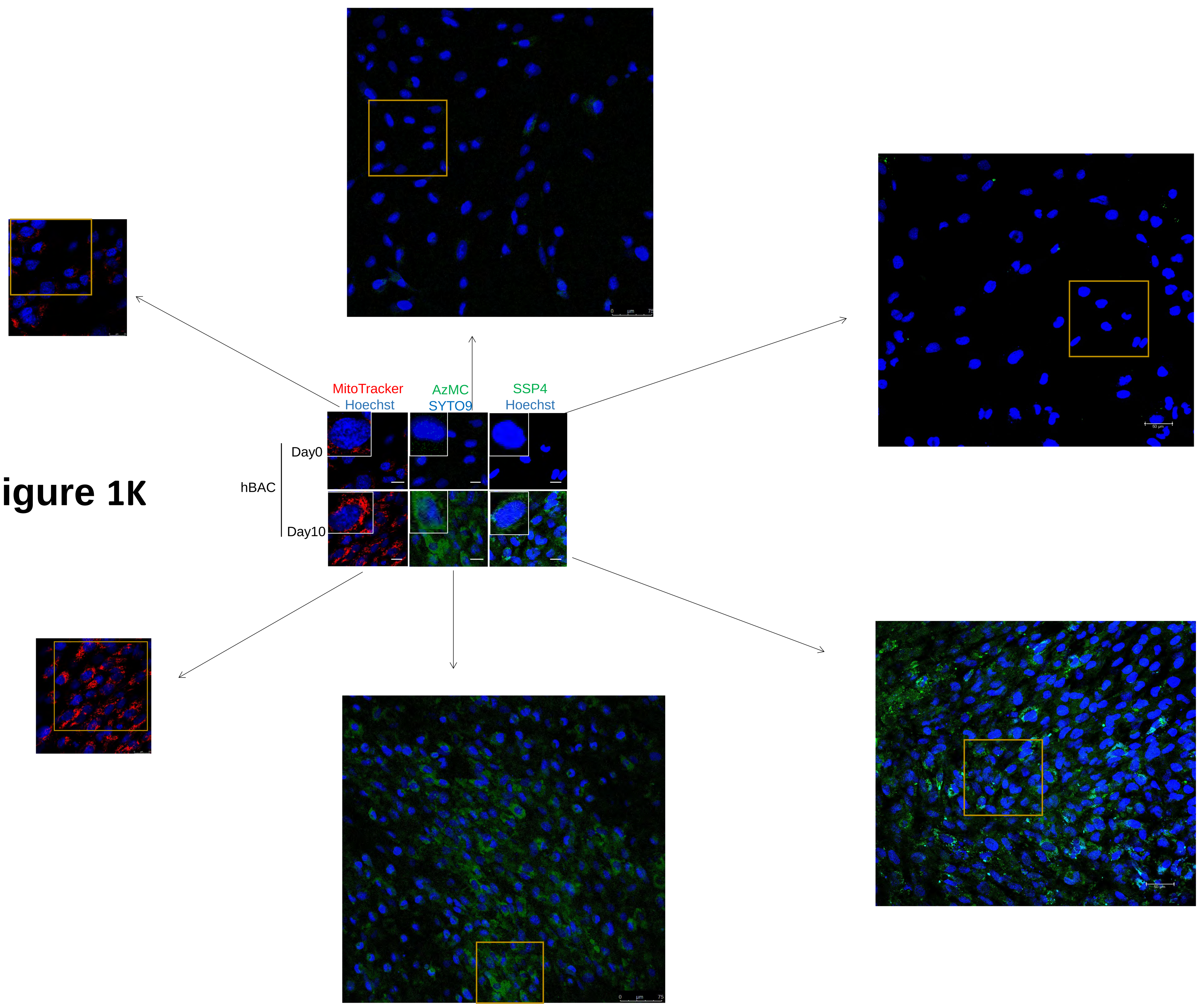
MitoTracker
Hoechst
AzMC
SYTO9
SSP4
Hoechst
hBAC
Day0
Day10

Figure 3G

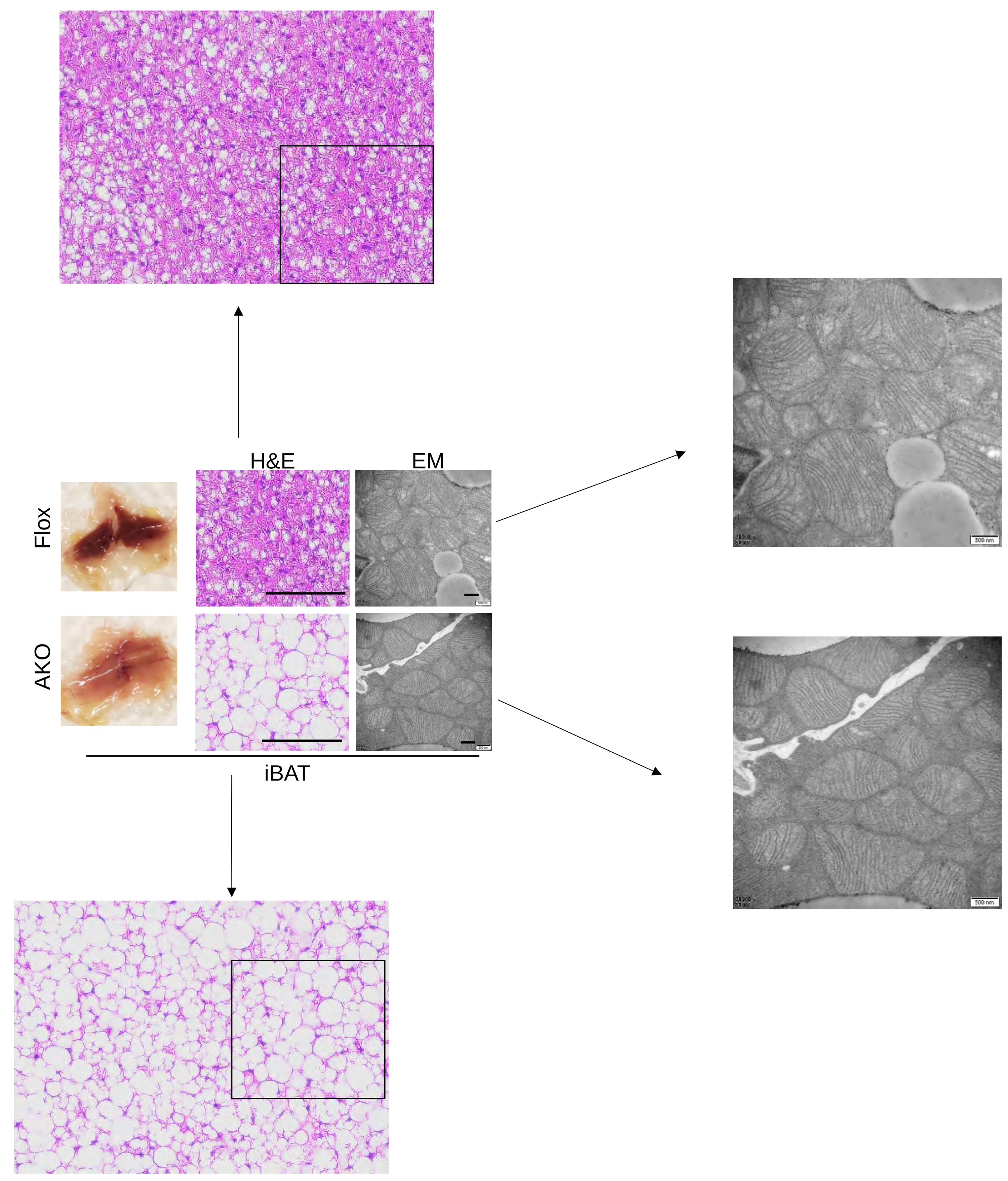

Figure 3H

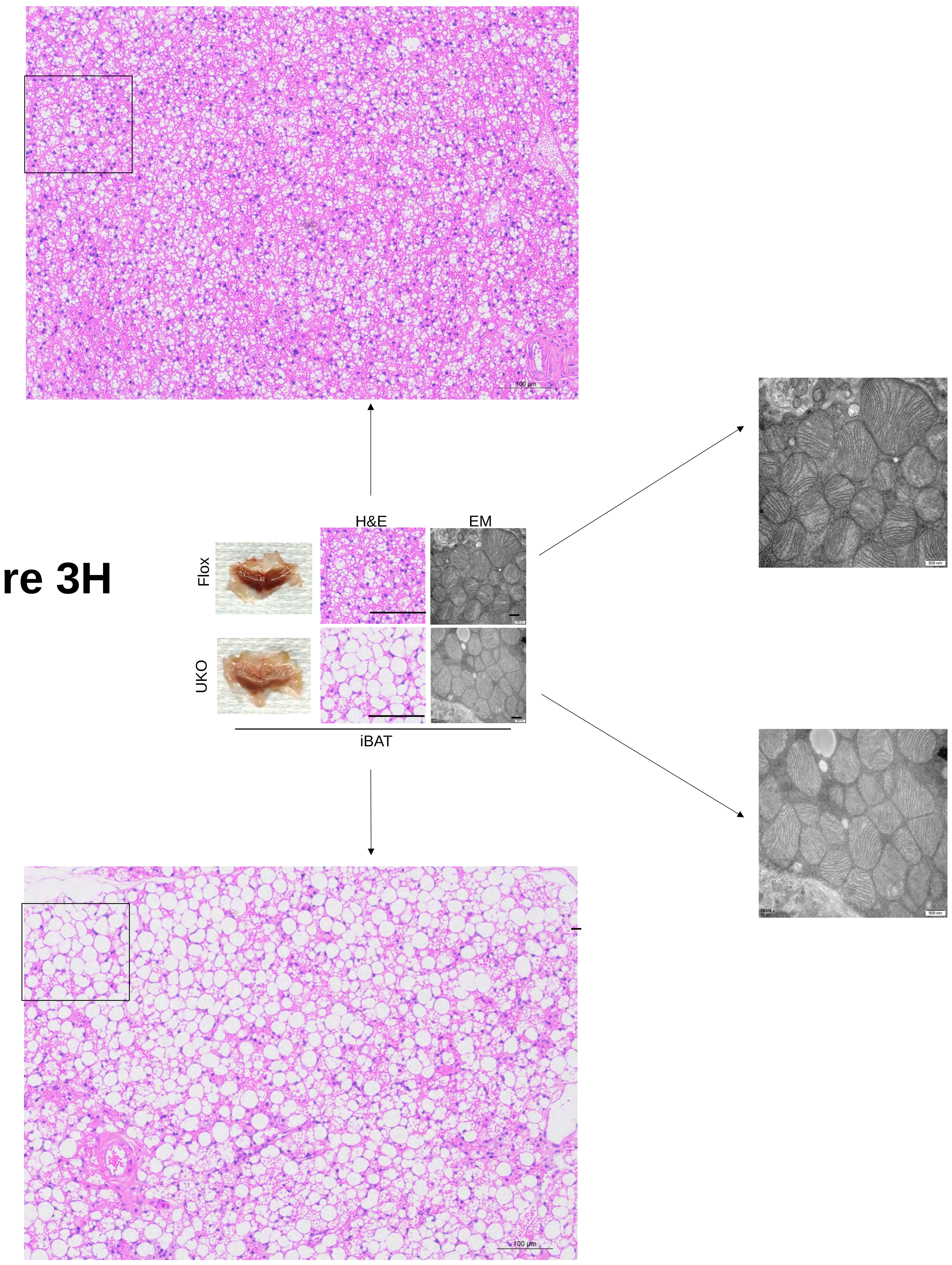

Figure 4E

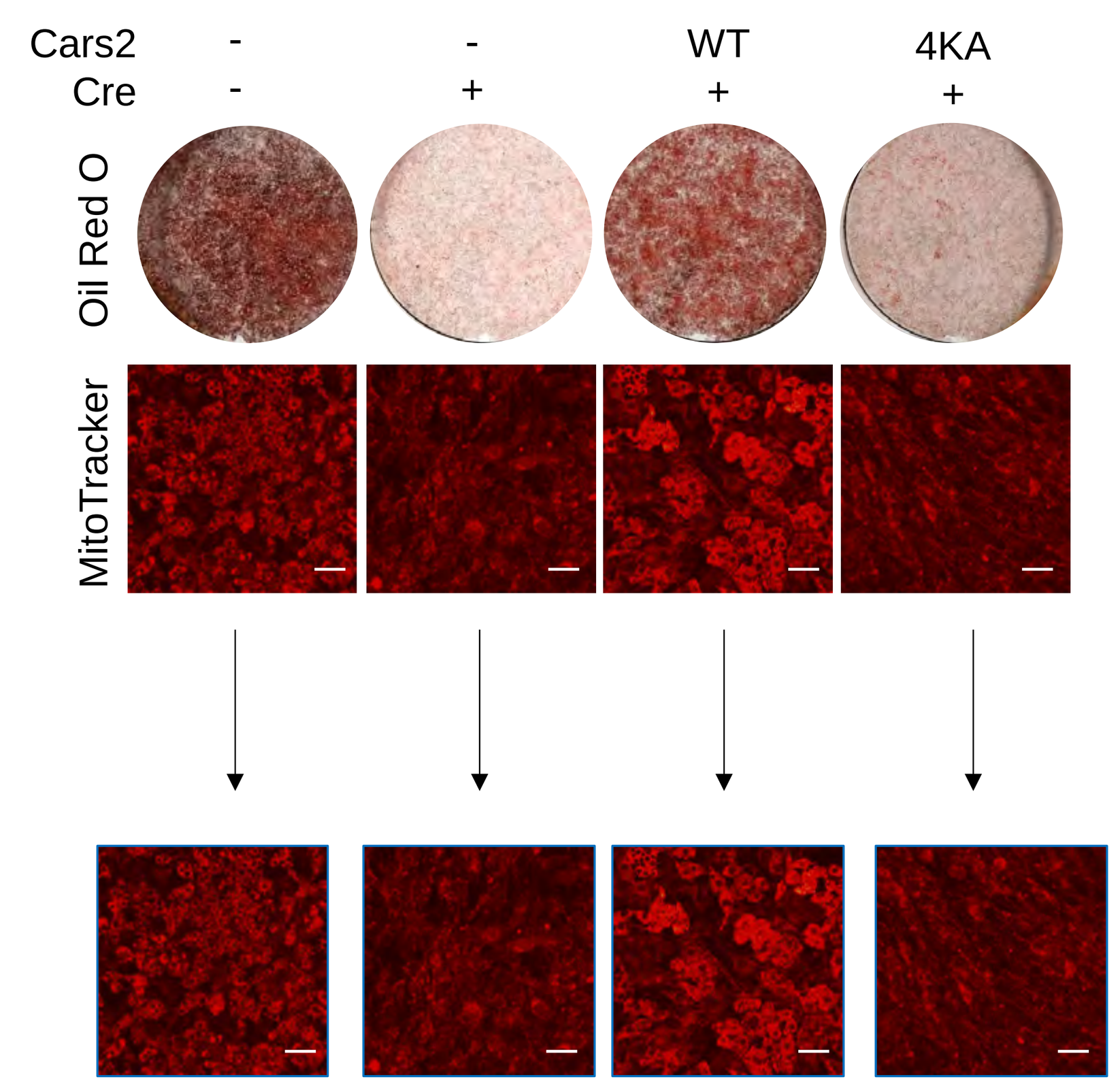

Figure 4J

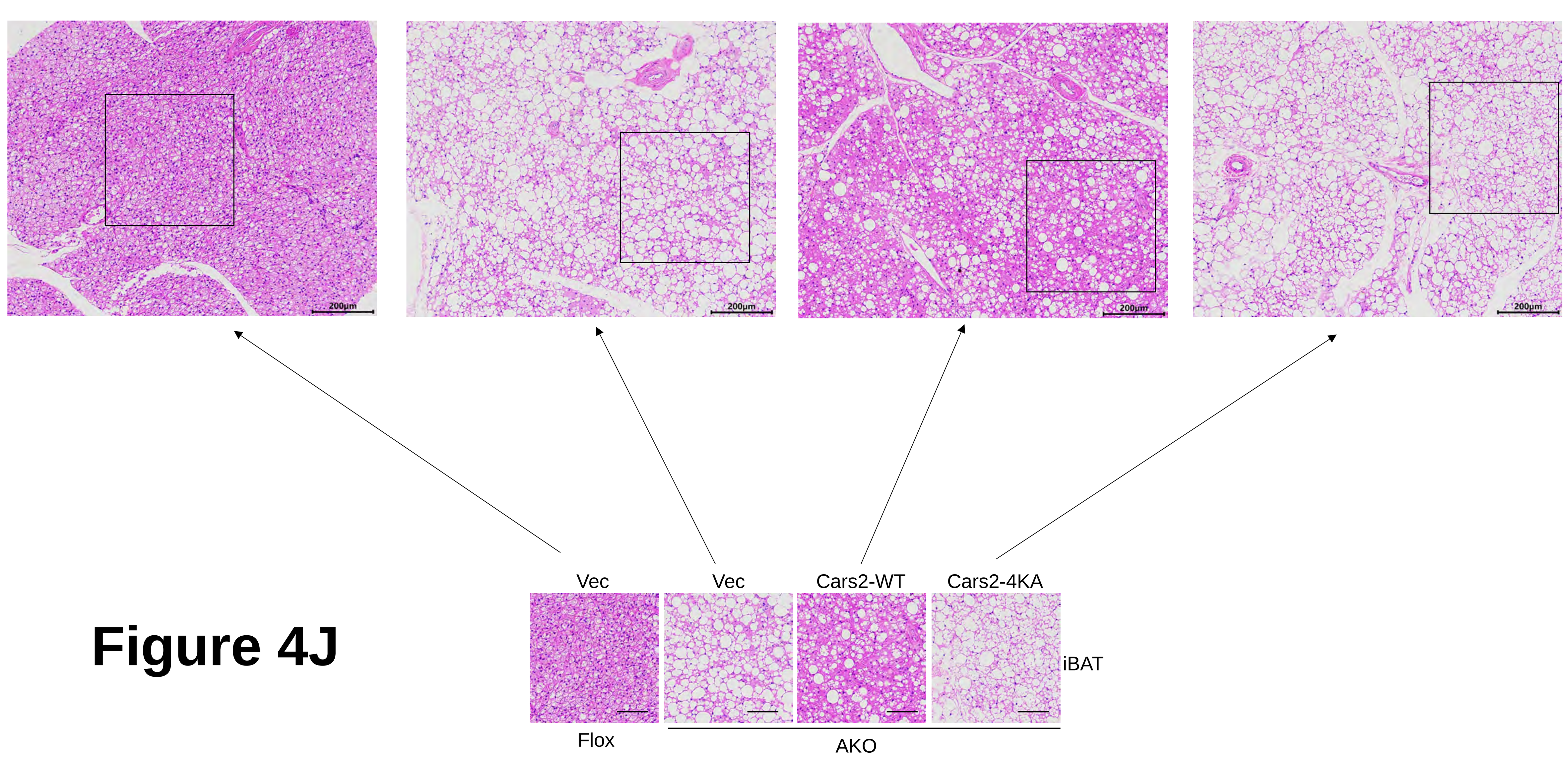

Figure 5O

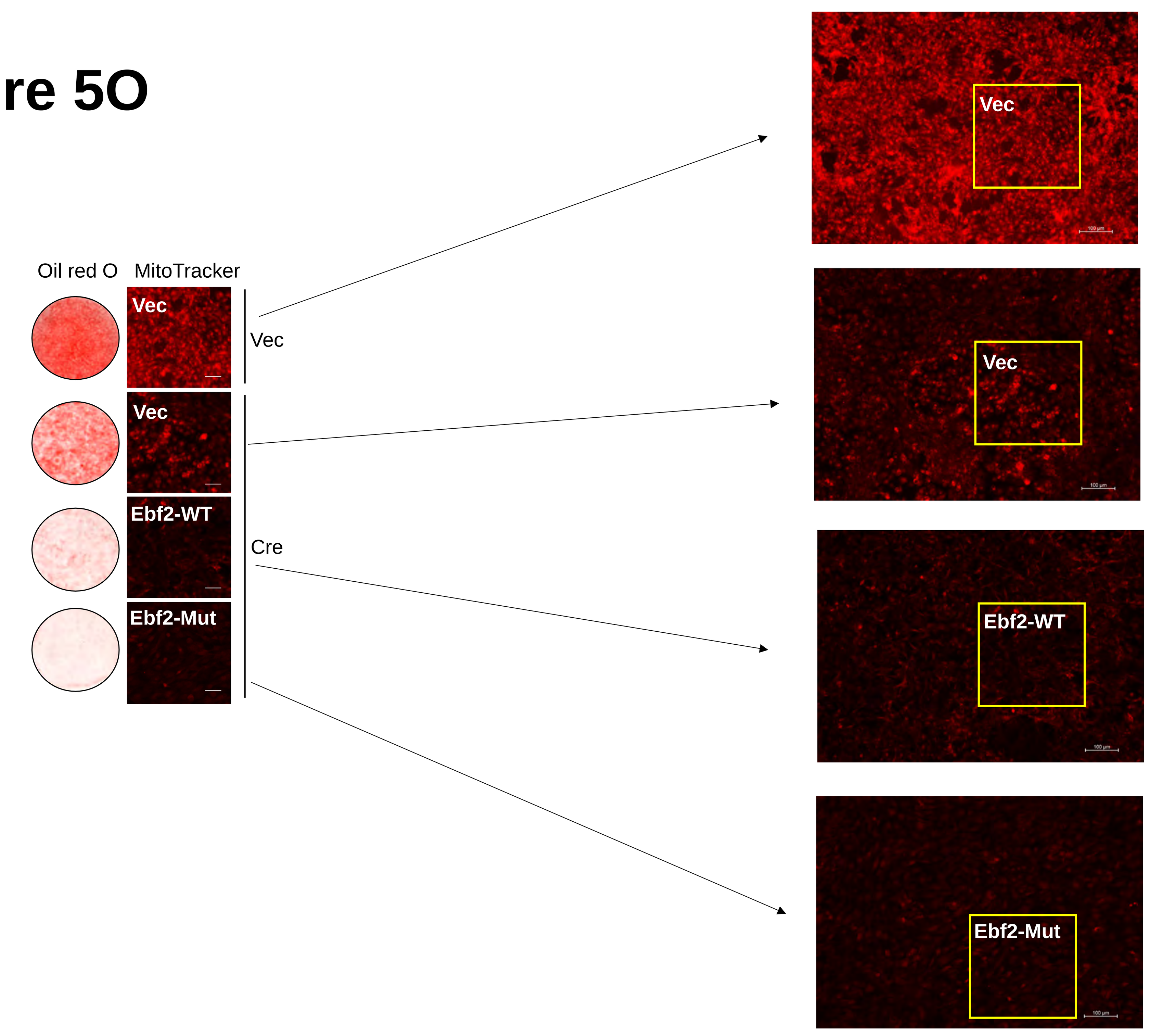
Oil red O
MitoTracker
Vec
Vec
Vec
Ebf2-WT
Ebf2-Mut
Cre
Vec
Vec
Ebf2-WT
Ebf2-Mut

Figure 6H

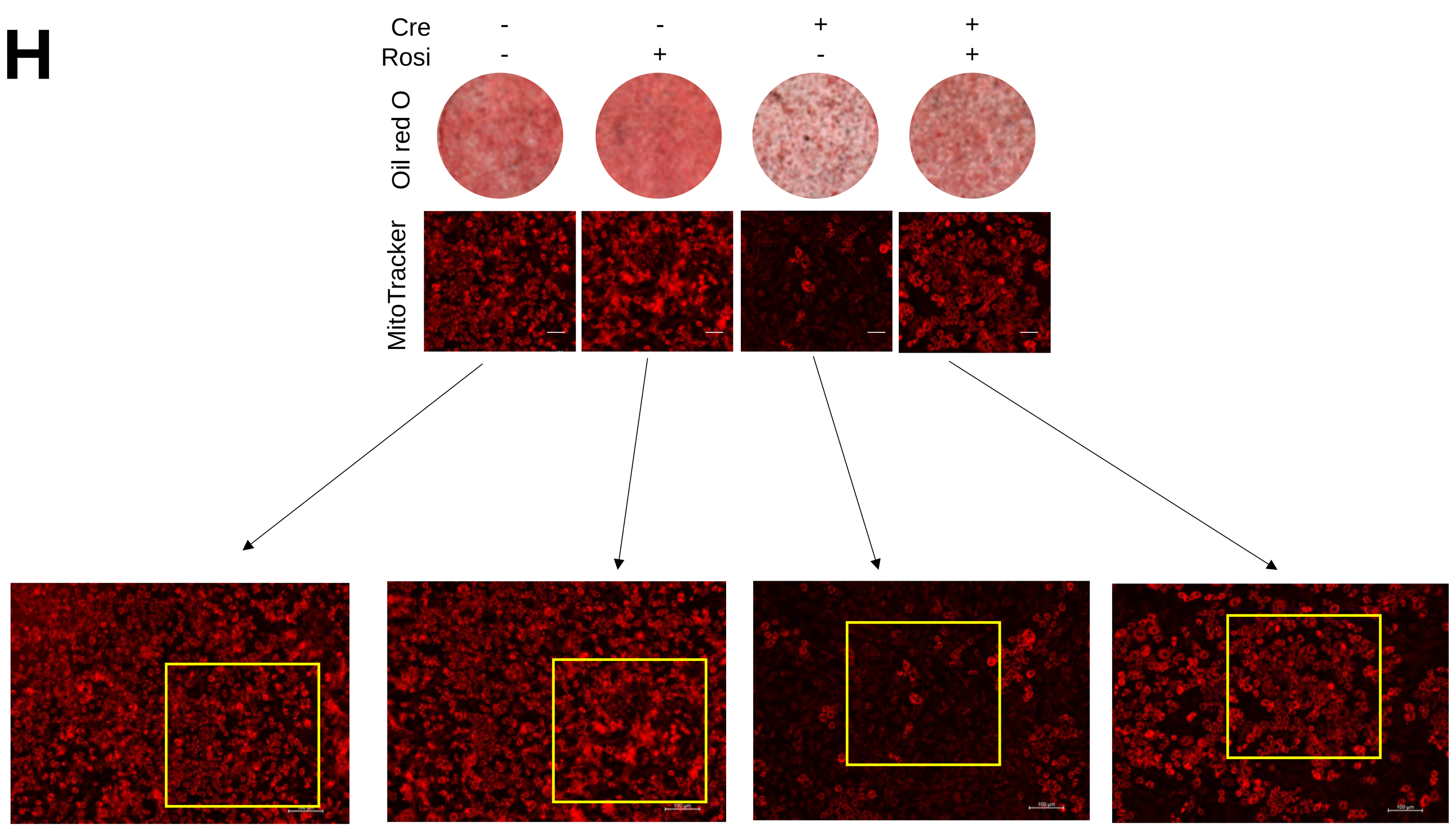

Figure 7L

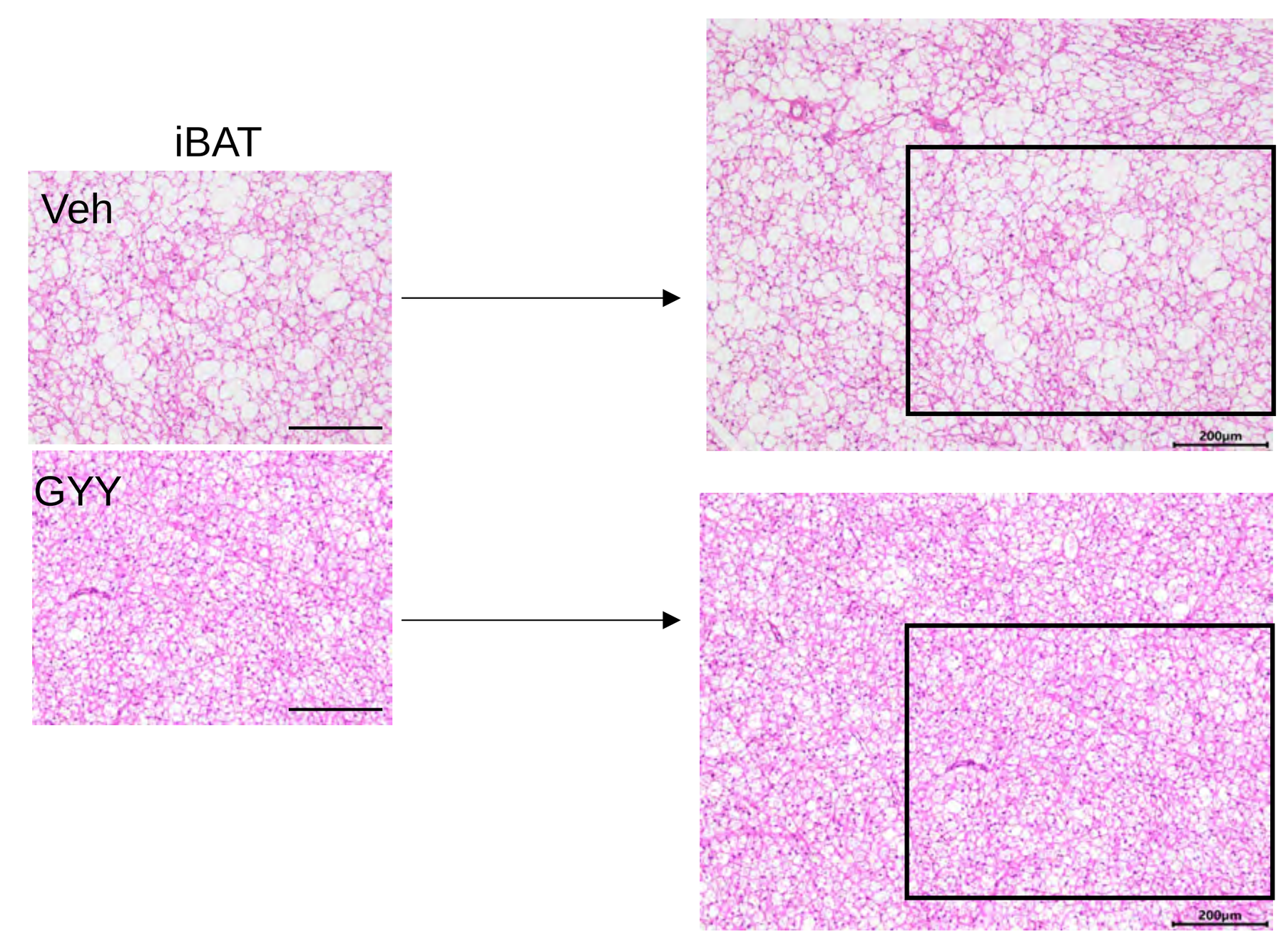

Figure 7T

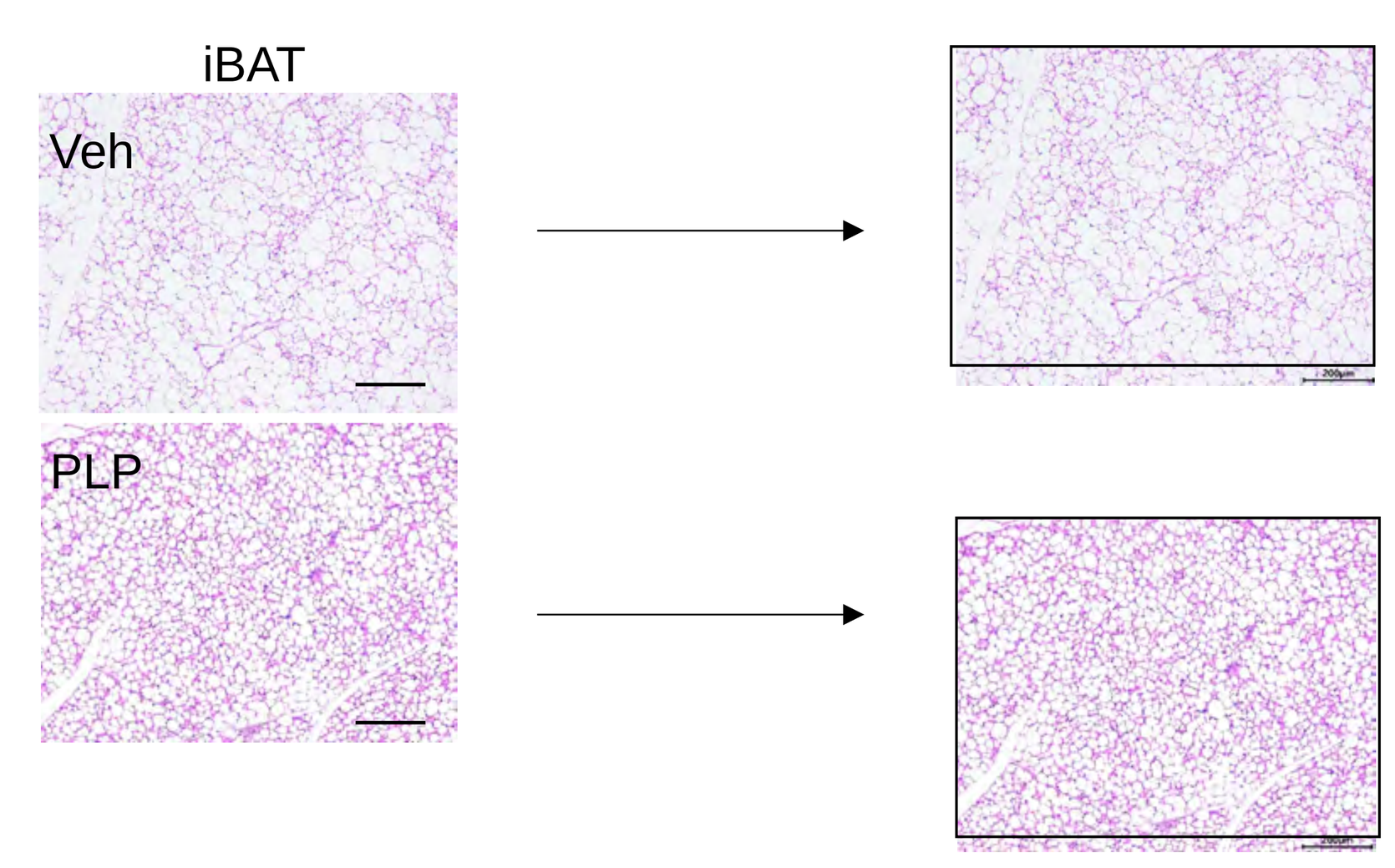

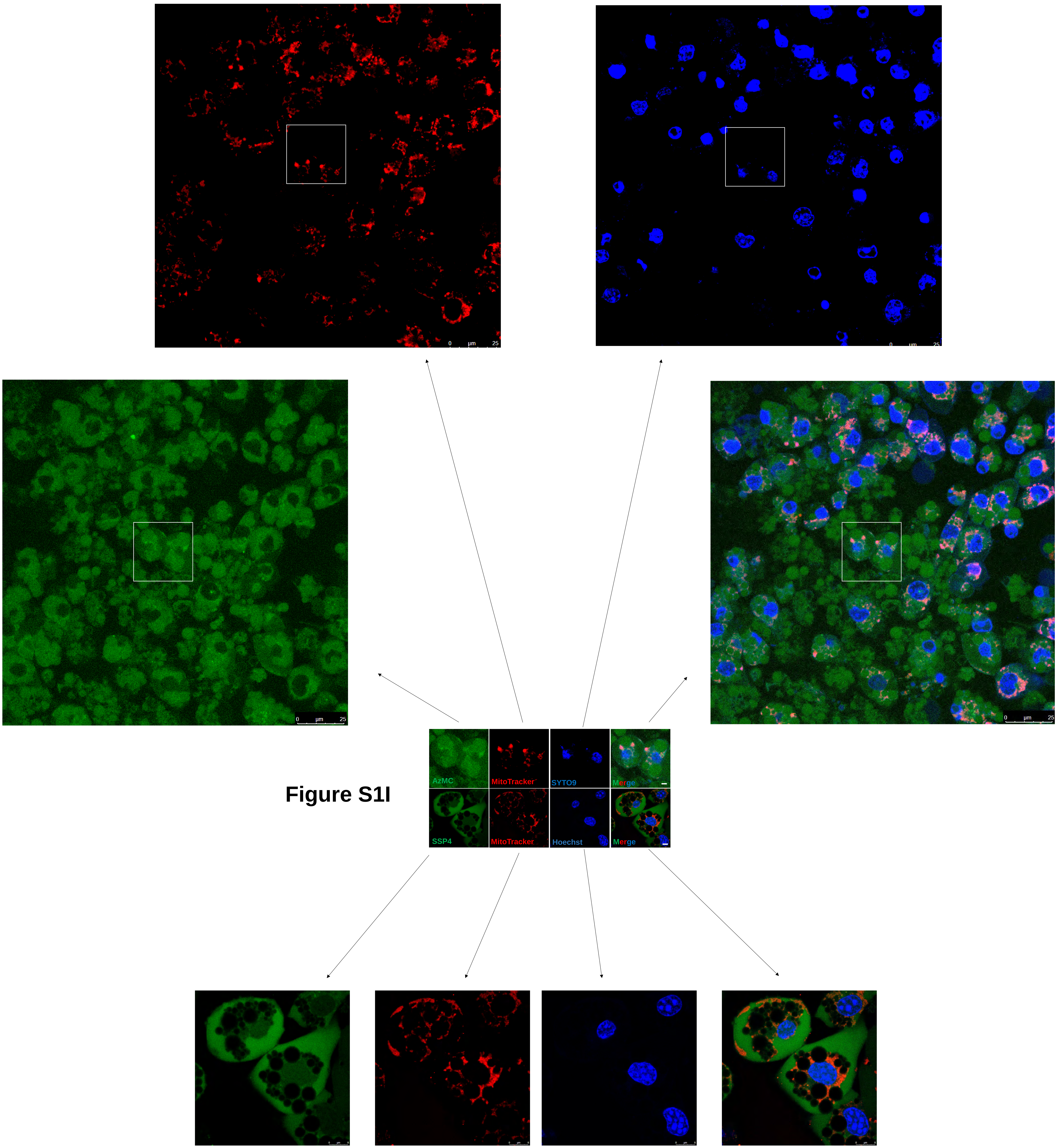

0 µm 25
0 µm 25
0 µm 25
0 µm 25
AzMC
MitoTracker
SYTO9
Merge
SSP4
MitoTracker
Hoechst
Merge

Figure S1I

Figure S2K

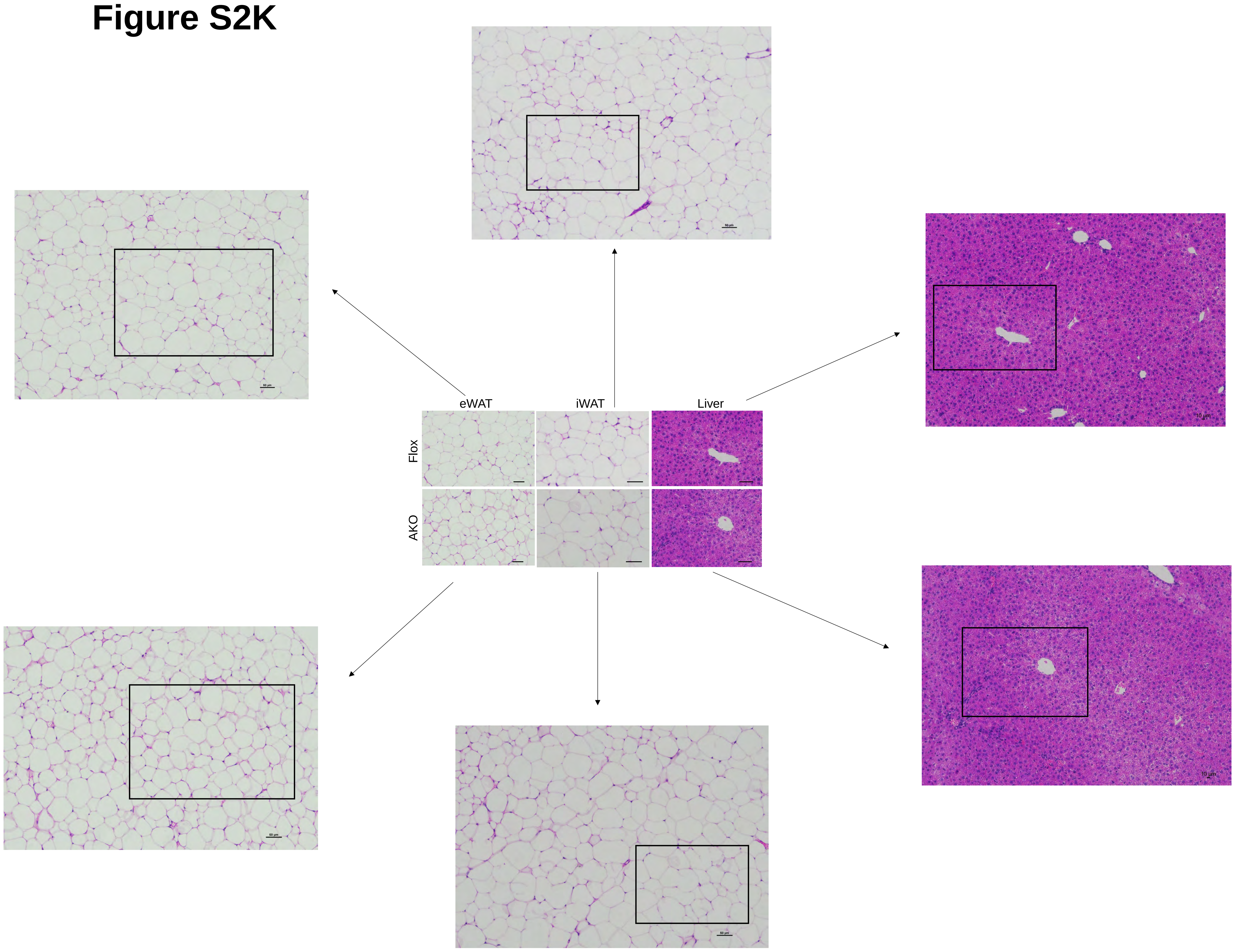

Figure S2L

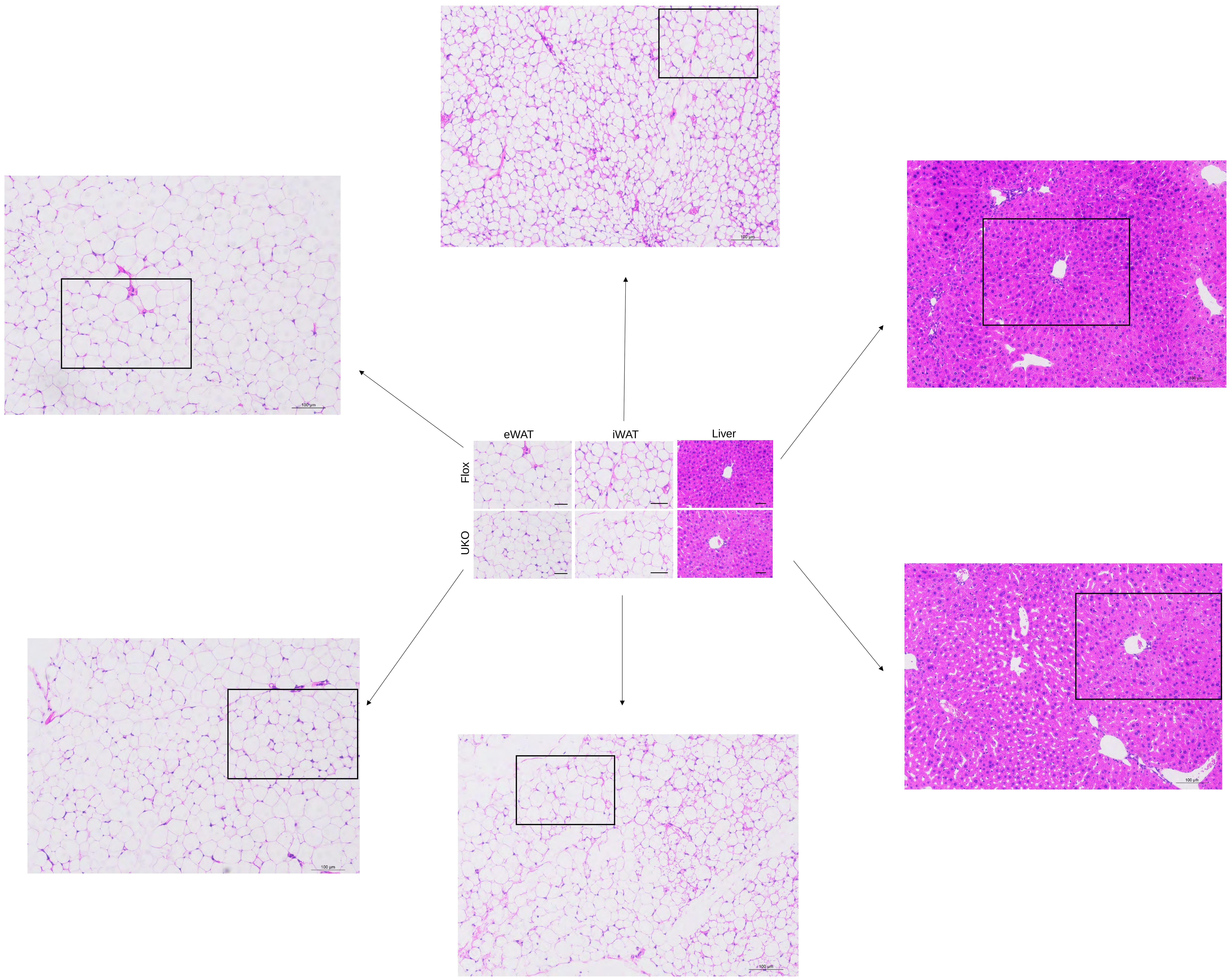

Figure S4E

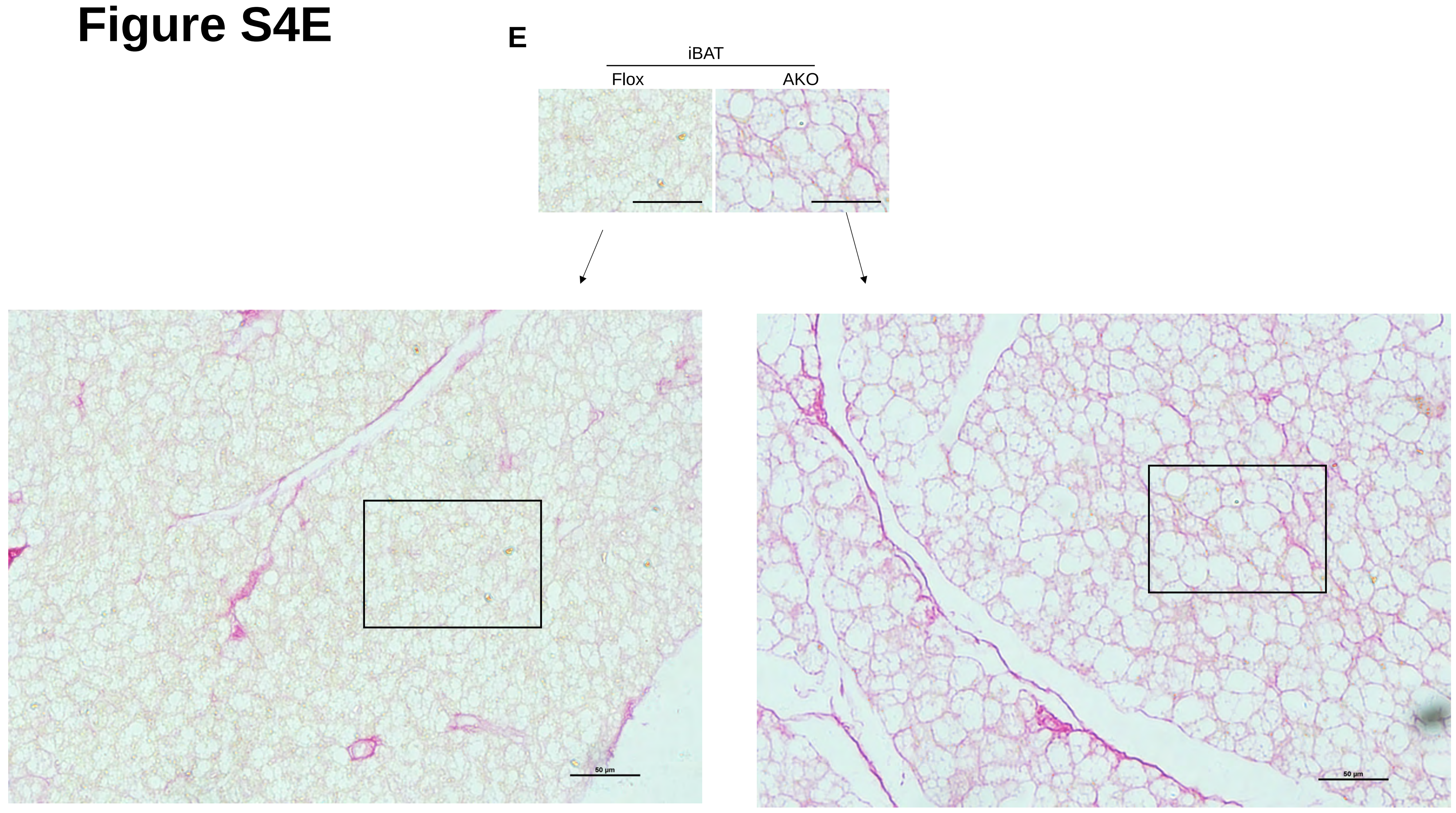

Figure S4F

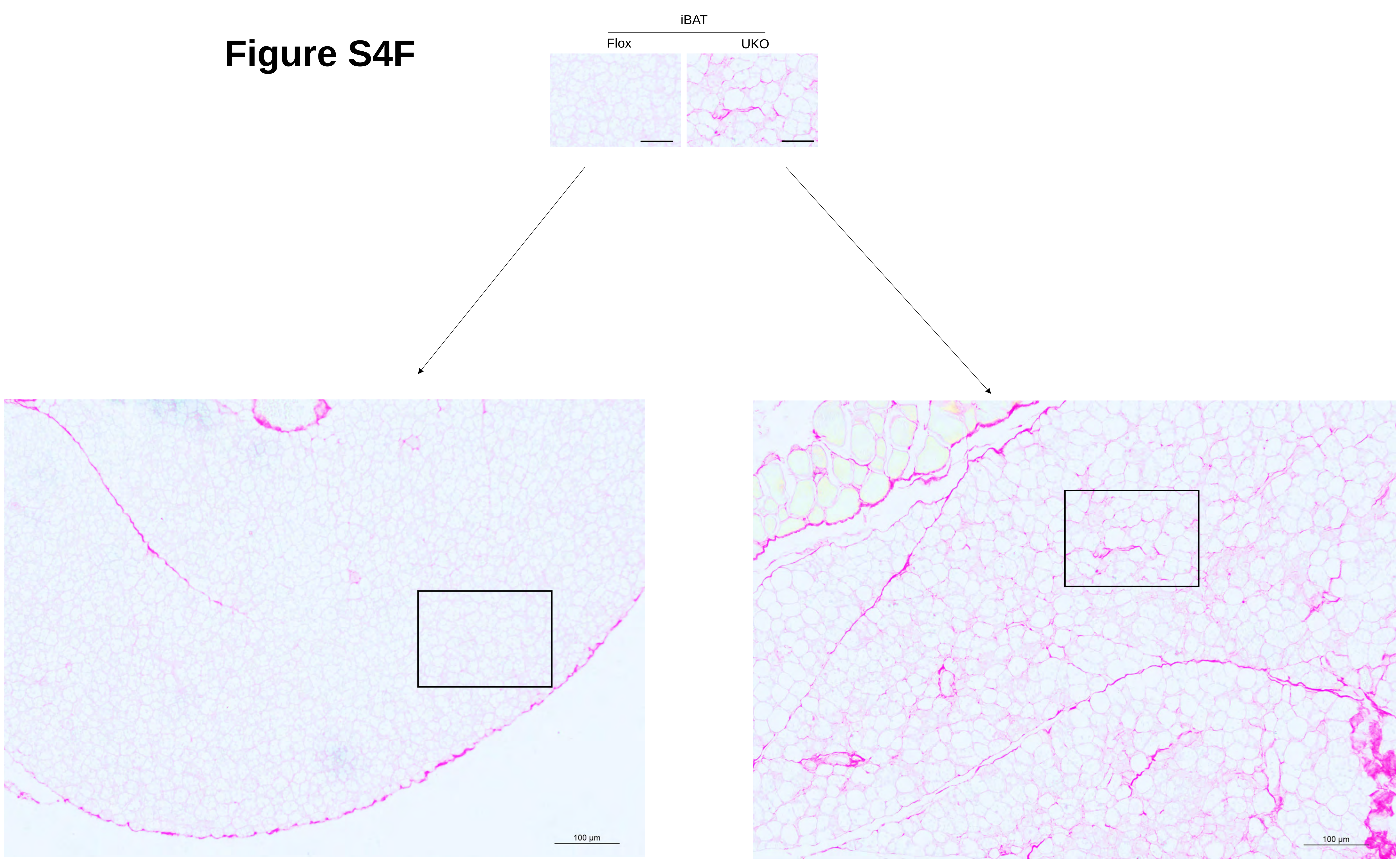

Figure S5B

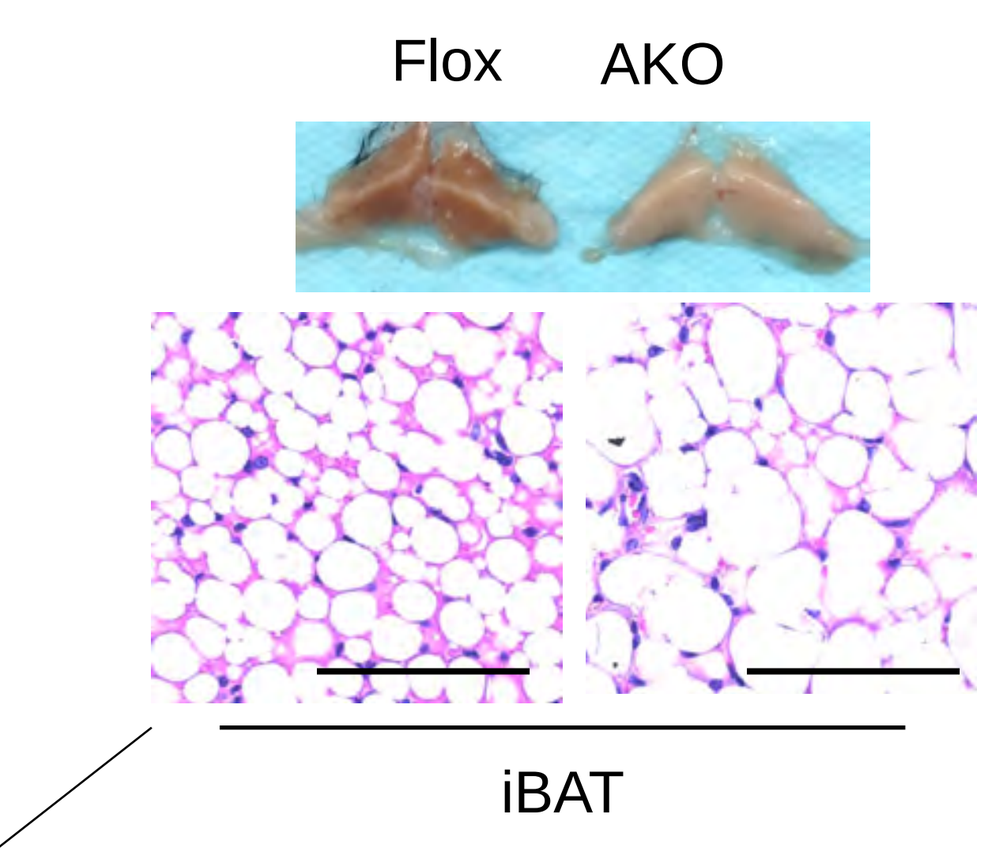

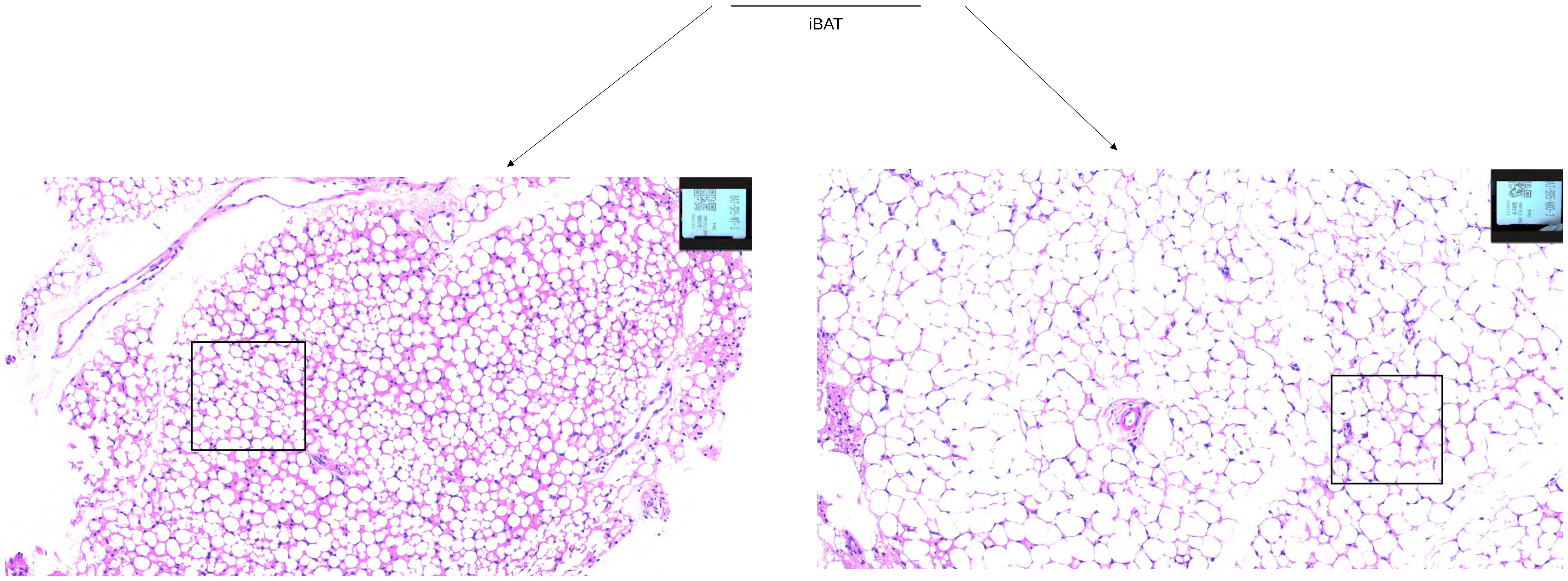

Figure S6K

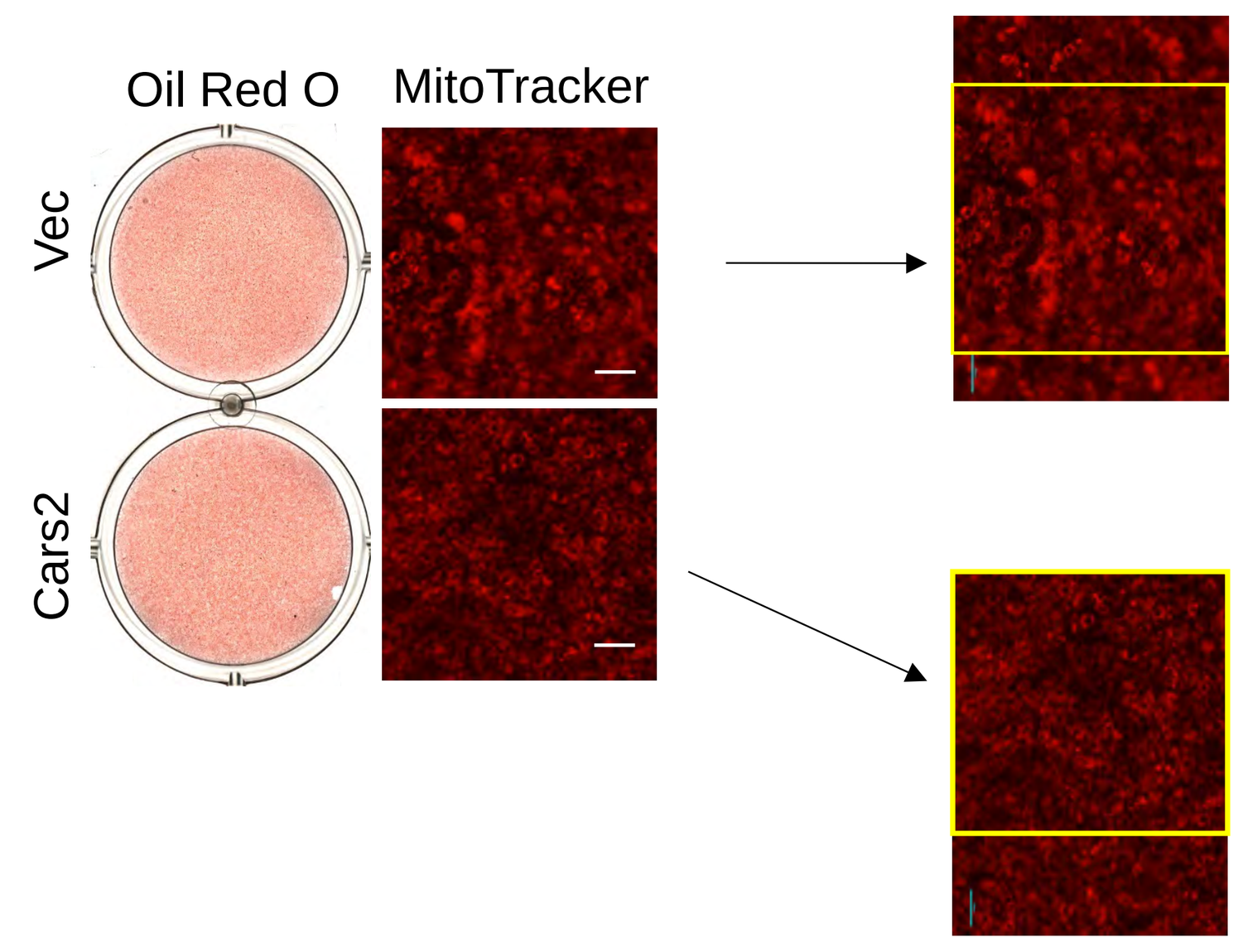

Figure S6L

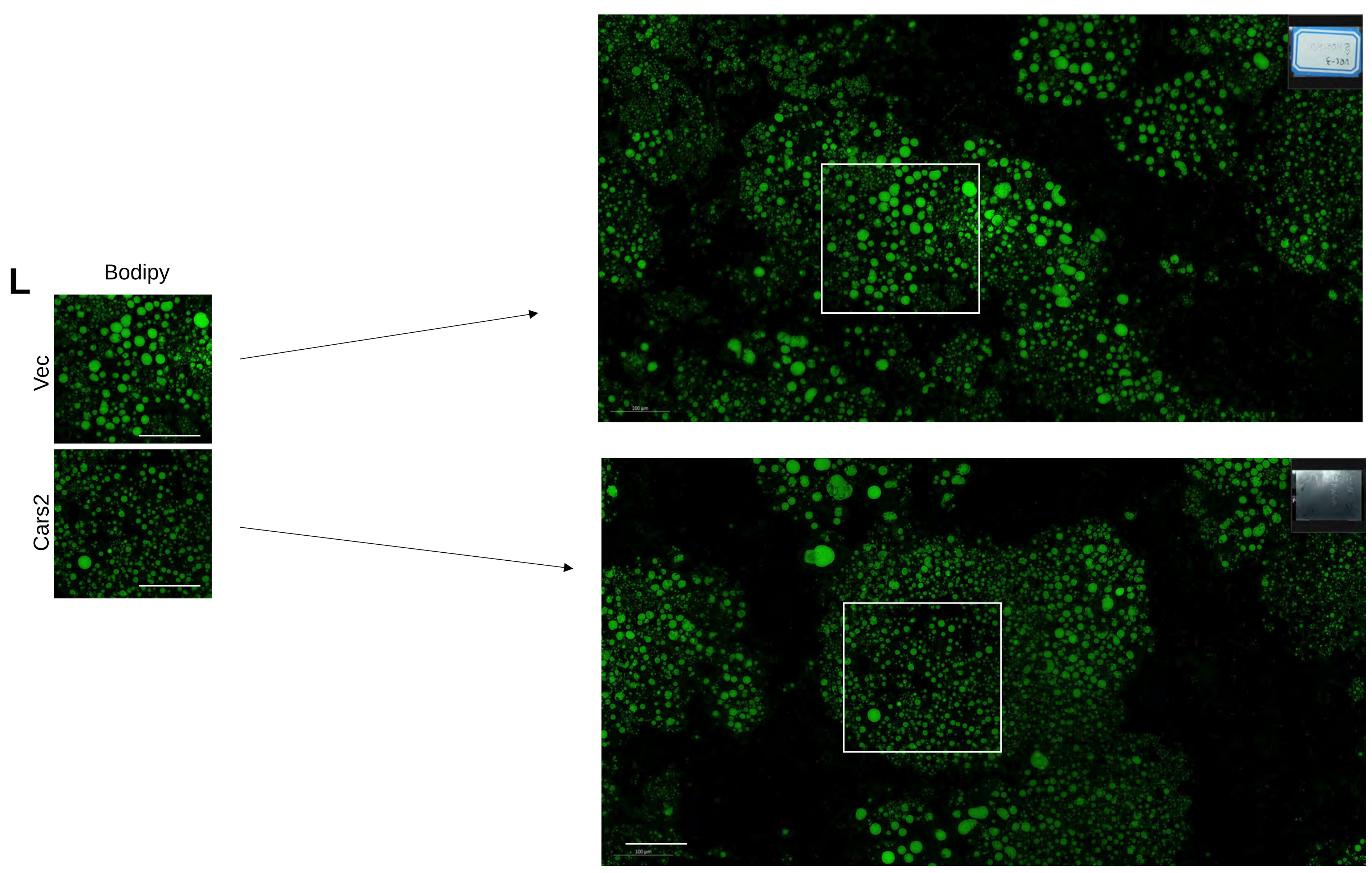

Figure S7K

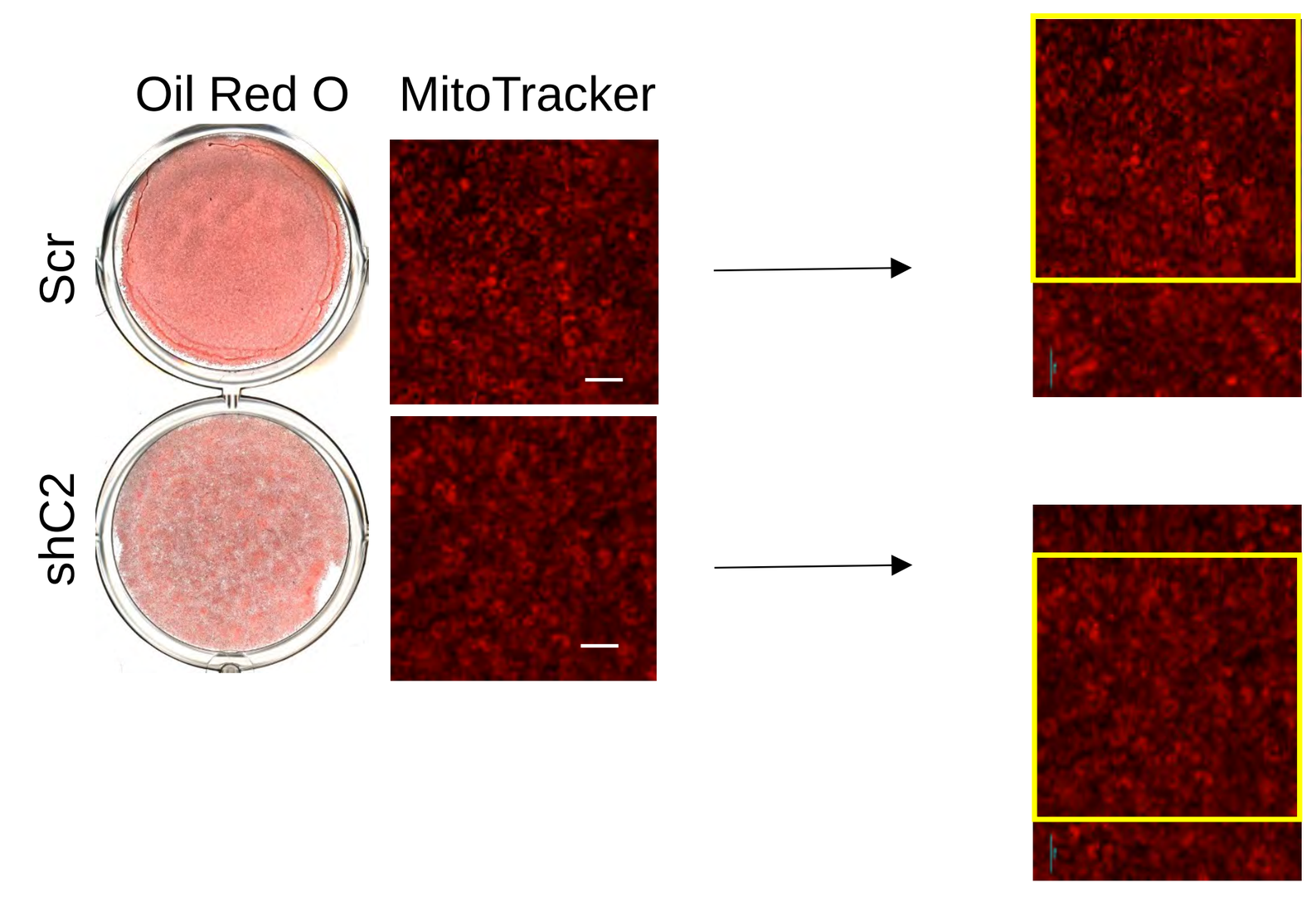

Figure S 8I

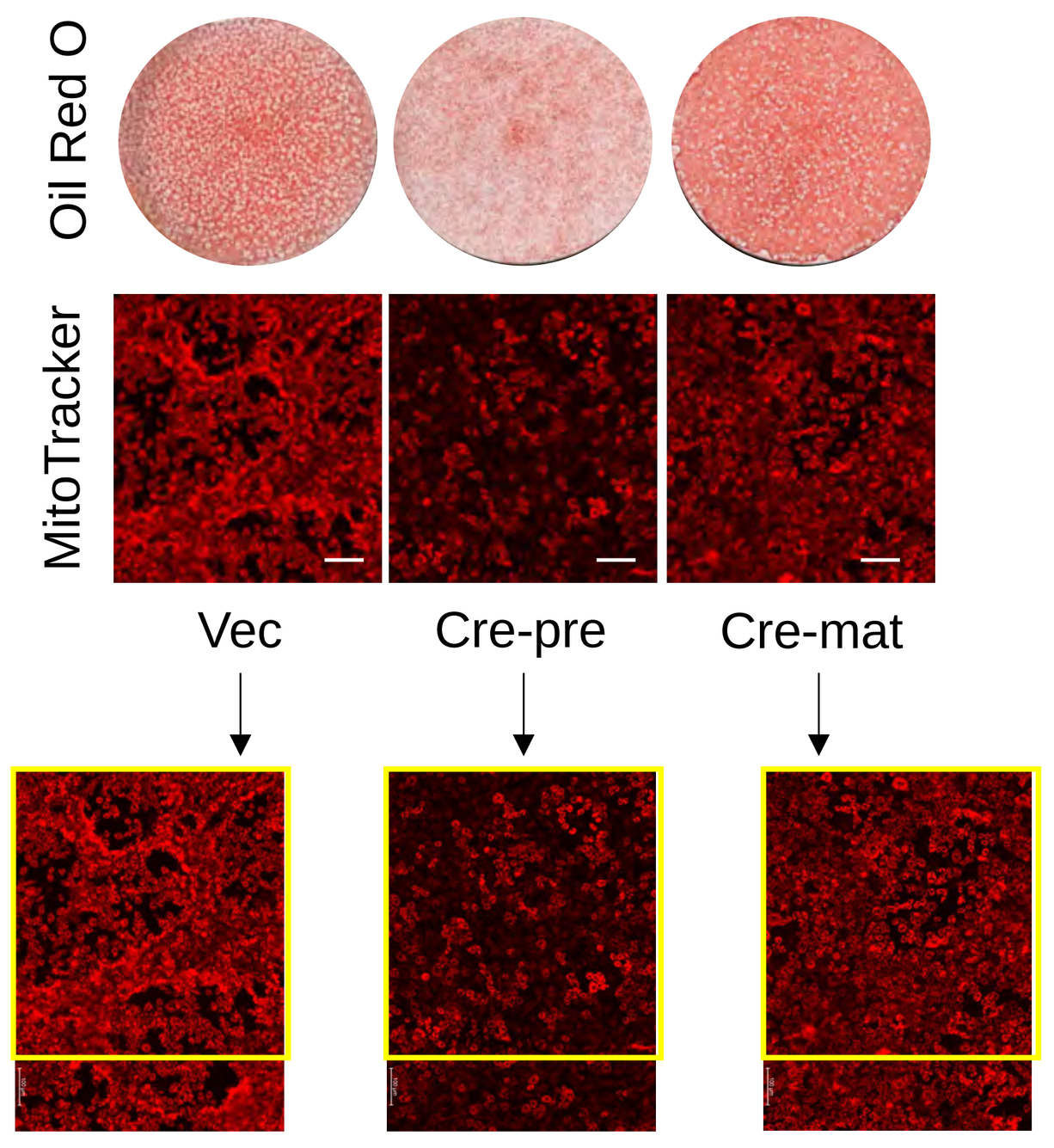

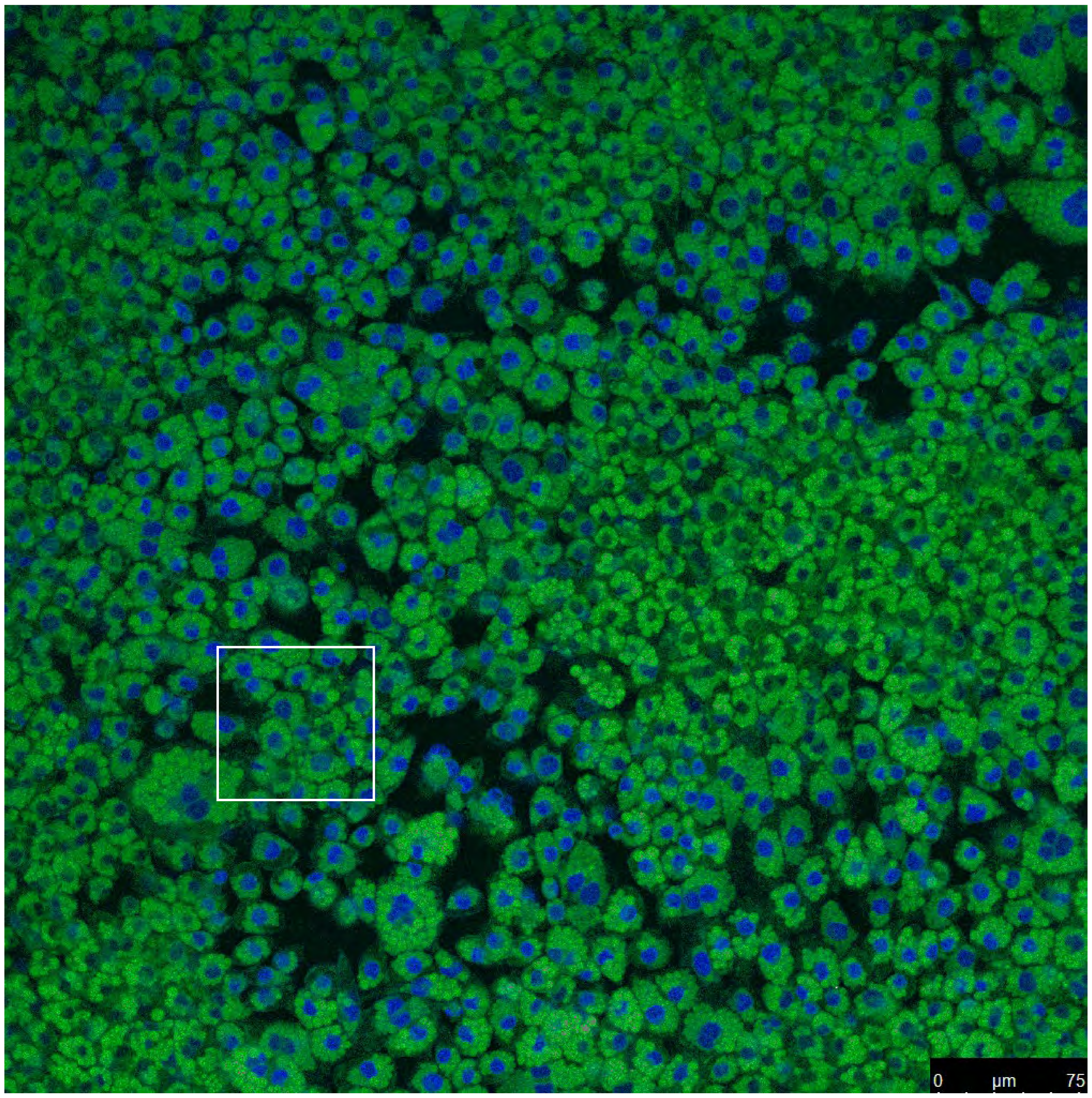

Figure S11 N left

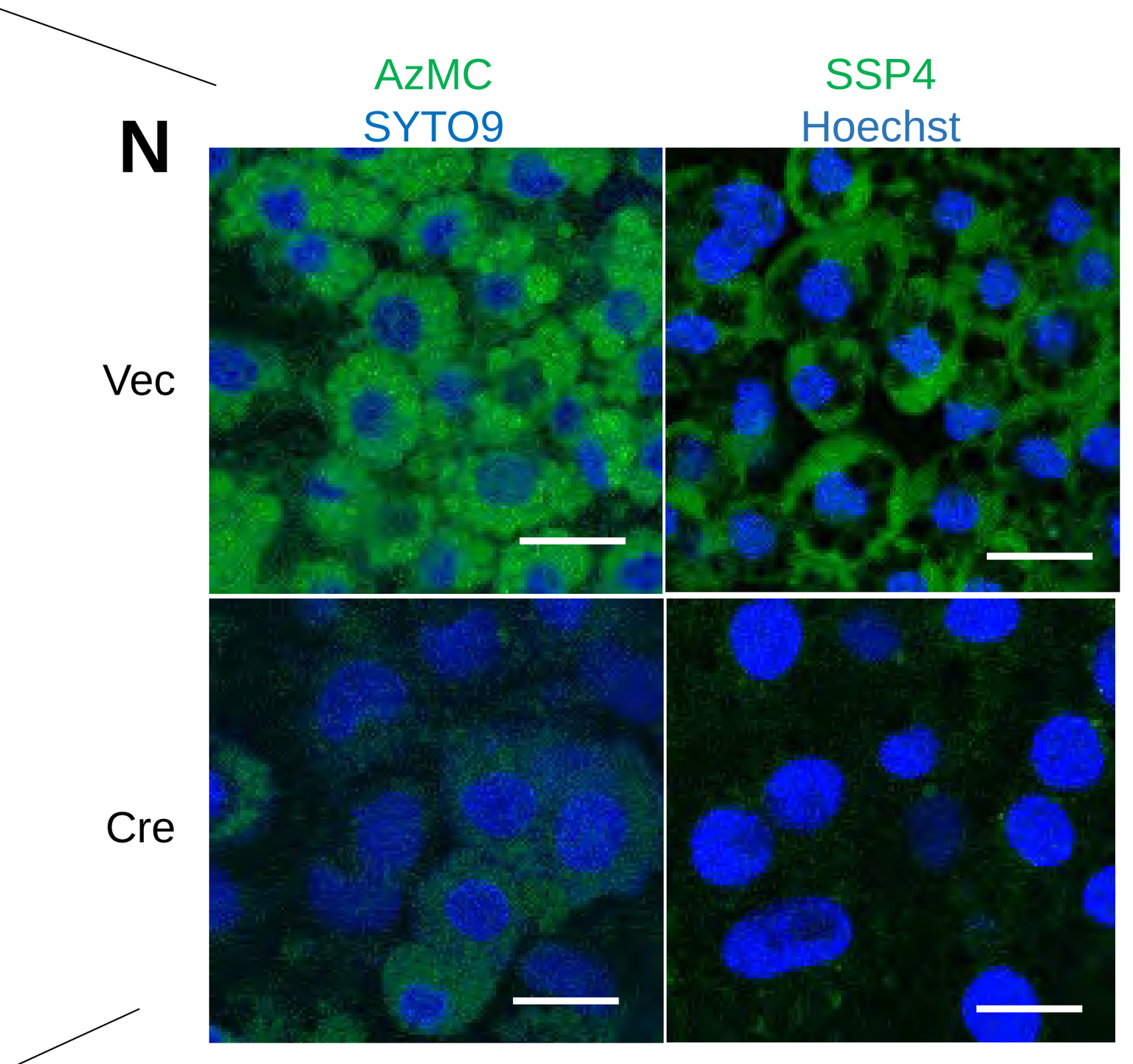

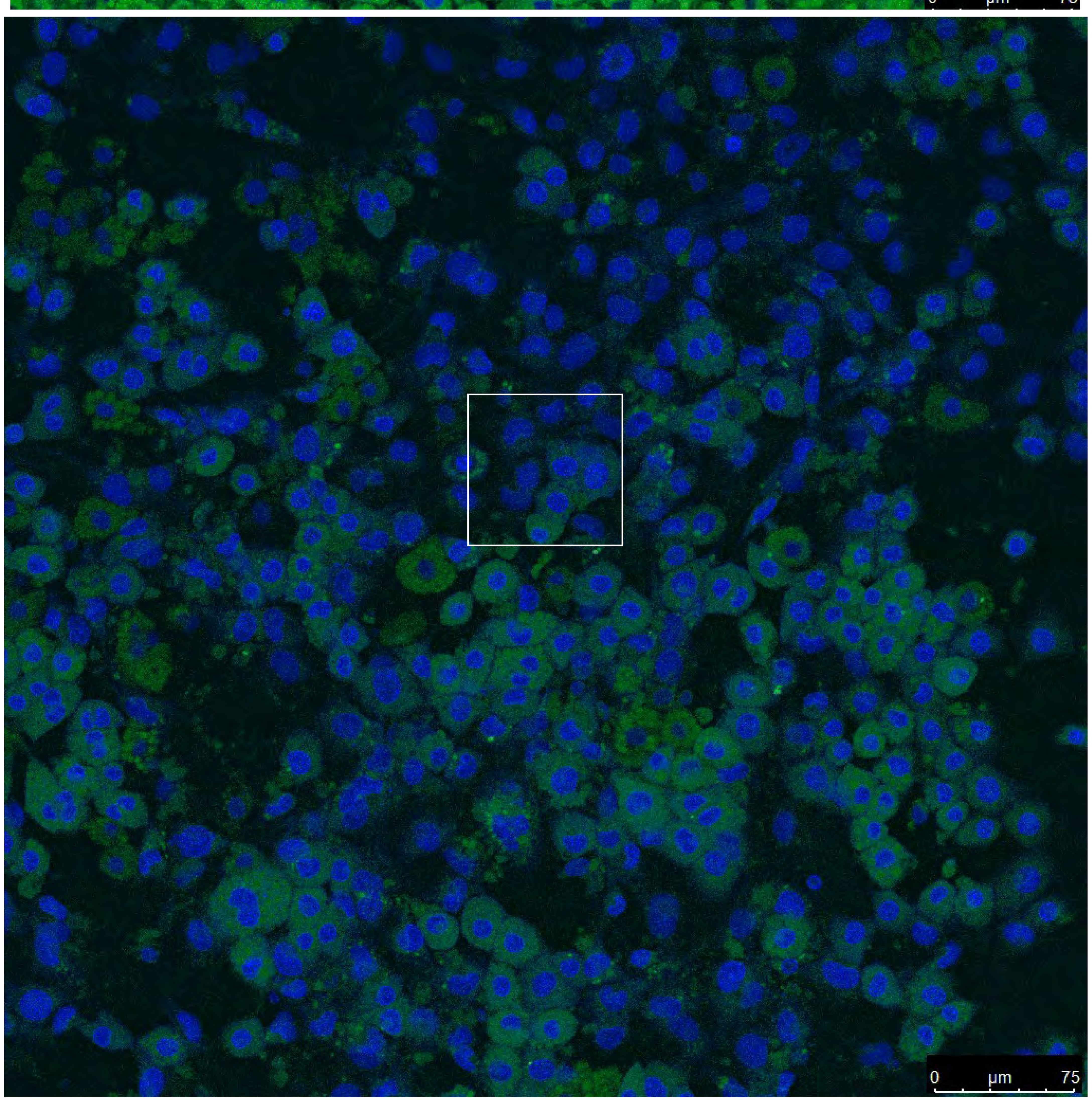

Figure S11 N right

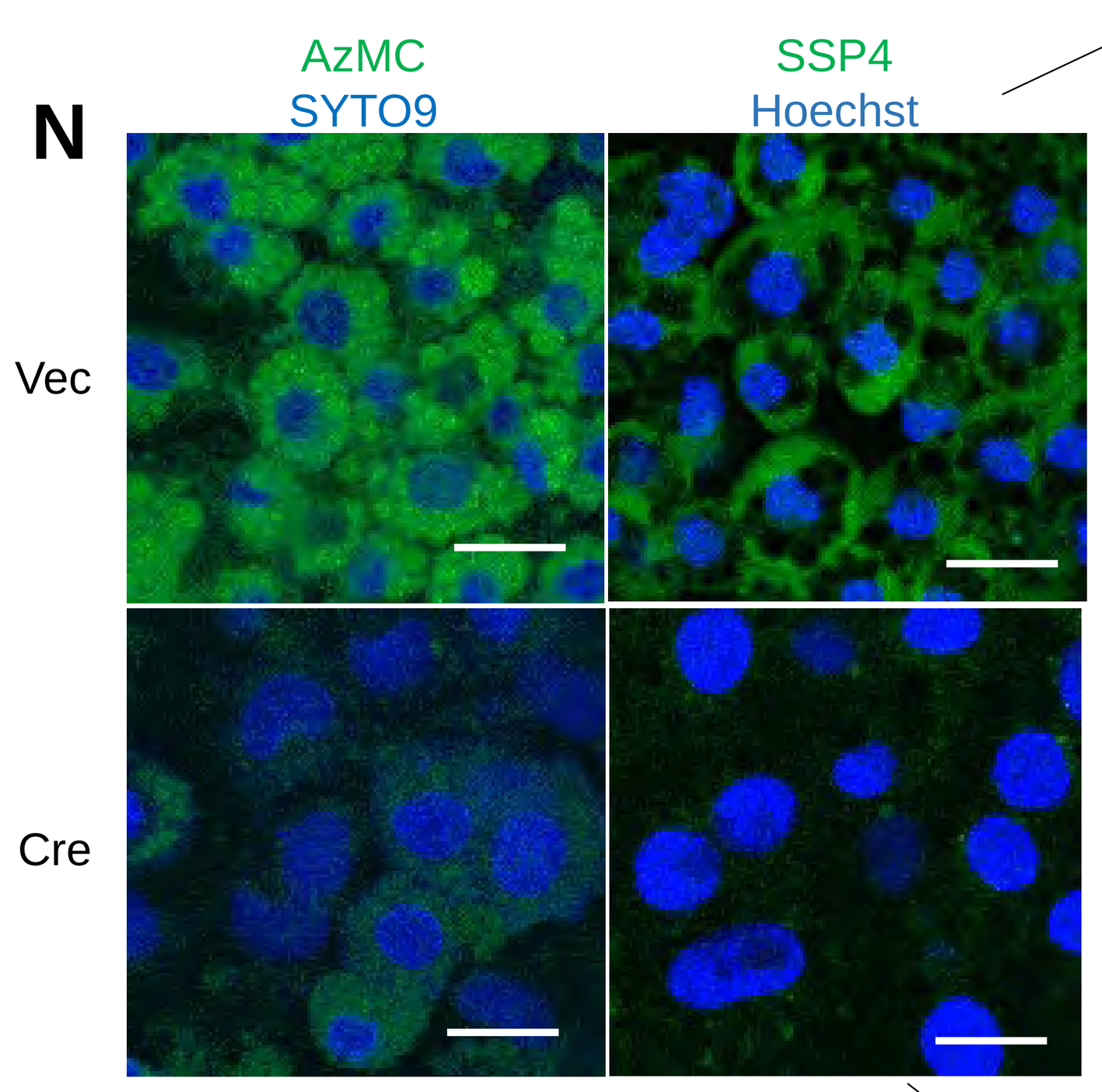

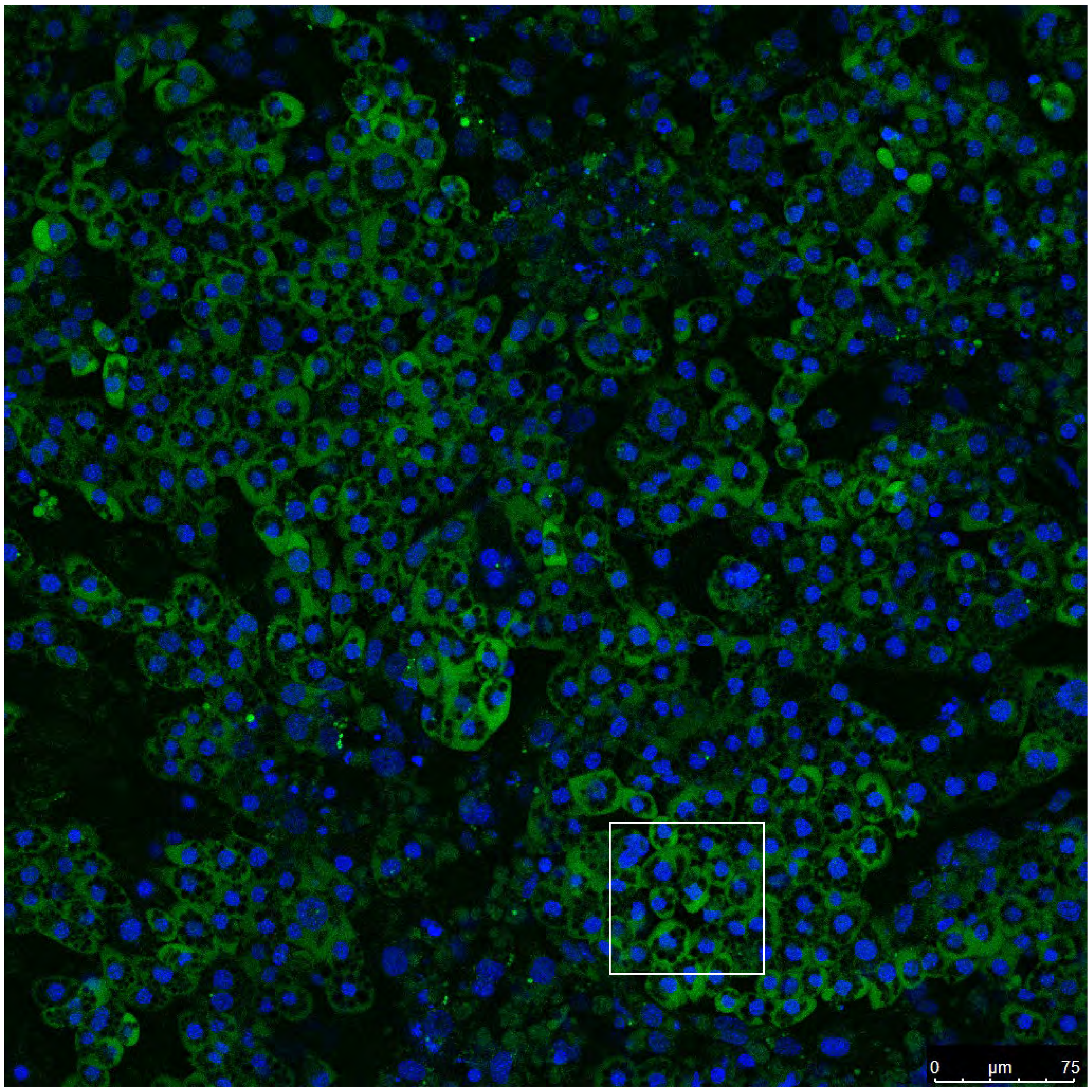

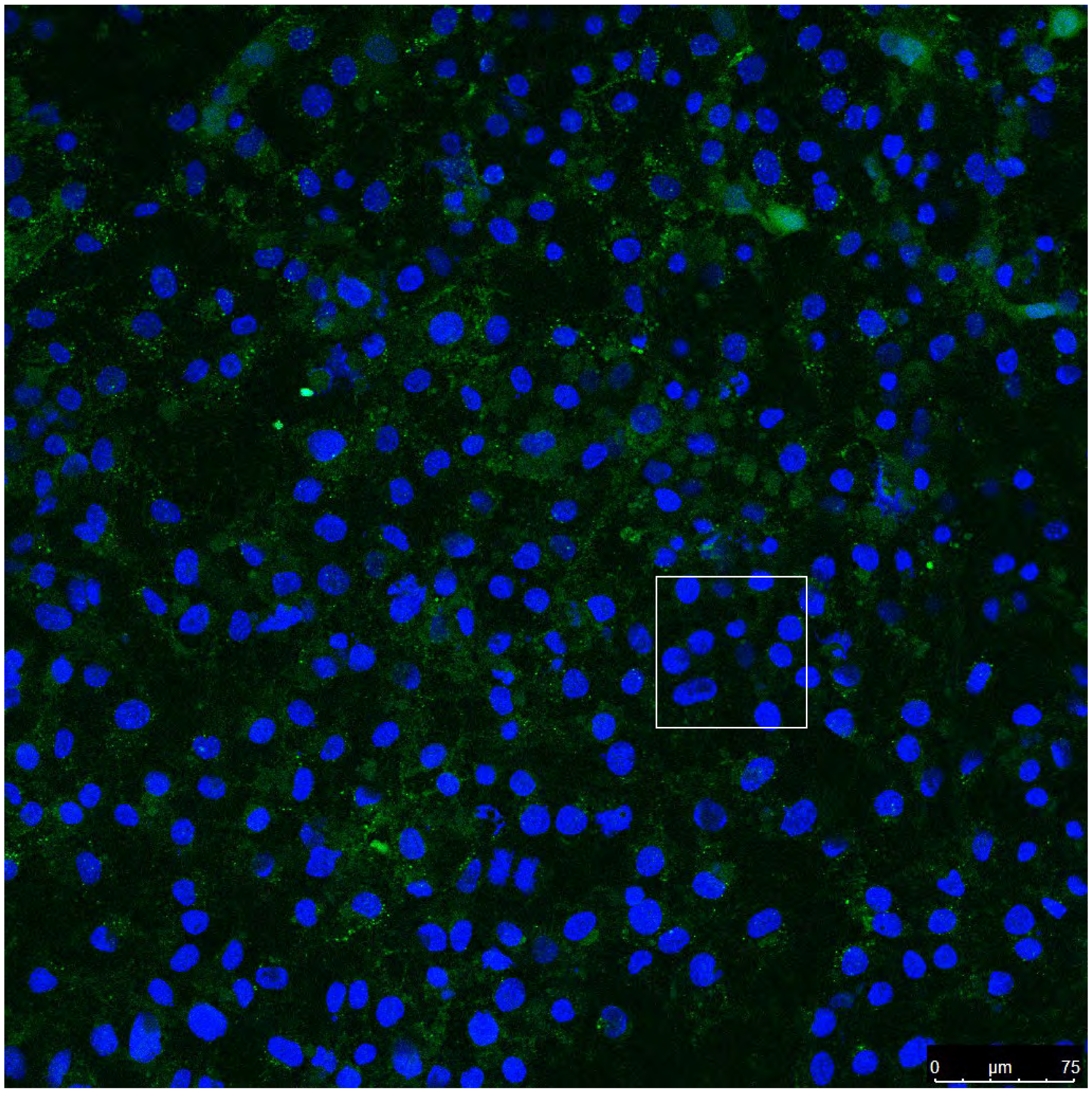

Supplement: Supplementary file 1 [file ADVS-13-e22690-s001.pdf]
