# Supplementary material for: Cars2‐Mediated Cysteine Catabolism Drives Brown Fat Development and Thermogenesis Through Persulfidating EBF2
Source: Adv Sci (Weinh). 2026 Mar 18;13(31):e22690. doi: 10.1002/advs.202522690 (PMC13588024; doi:10.1002/advs.202522690)
Supplement: Supplementary file 2 [file ADVS-13-e22690-s003.pdf]

Table S1: List of primer sequences.

**Primers for qPCR**		
**Mouse**		
Gene	Forward	Reverse
Ucp1	GGCATTCAGAGGCAAATCAGCT	CAATGAACACTGCCACACCTC
Cidea	GCAGCCTGCAGGAACTTATCAGC	GATCATGAAATGCGTGTTGTCC
Dio2	GATGCTCCCAATTCCAGTGT	TGAACCAAAGTTGACCACCA
Prdm16	CGGAAGAGCGTGAGTACAAATG	TCCGTGAACACCTTGACACAGT
Pgc1α	AGCCGTGACCACTGACAACGAG	GCTGCATGGTTCTGAGTGCTAAG
Cars2	GTGGACCGACTGTGTACGAC	CCACATTGCATCCAAACACTCT
Ebf2	GGAACCGGAACGAGACCCCT	TCCCTTGGGTTTCCCGCTGT
Cre	GAAAATGCTTCTGTCCGTTTGC	ATTGCTGTCACTTGGTCGTGGC
Cbs	TCAAAGAATGCCGGTCTCAAGT	TGTGTTGCCAGAAGTTGGCTC
Cse	CATTTAAGCAGGACTTCCCGG	TCGTAATGGTGGCAGCAAGAC
mtNd1	CCTATCACCCTTGCCATCAT	GAGGCTGTTGCTTGTGTGAC
mtCox1	CTACTATTCGGAGCCTGAGC	GCATGGGCAGTTACGATAAC
Col1a1	AAGAGGCGAGAGAGGTTTCC	AGAACCATCAGCACCTTTGG
Col1a2	GTAACTTCGTGCCTAGCAACA	CCTTTGTCAGAATACTGAGCAGC
Col3a1	CTGTAACATGGAAACTGGGGAAA	CCATAGCTGAACTGAAAACCACC
Col5a2	ACAGGTGAAGTGGGATTCTCA	CCATAGCACCCATTGGACCA
Col5a3	CGGGGTACTCCTGGTCCTAC	GCATCCCTACTTCCCCCTTG
Col6a1	CTGCTGCTACAAGCCTGCT	CCCCATAAGGTTTCAGCCTCA
Col6a2	AAGGCCCCATTGGATTCCC	CTCCCTTCCGACCATCCGAT
Col6a3	GCTGCGGAATCACTTTGTGC	CACCTTGACACCTTTCTGGGT

Srebp1c	TGACCCGGCTATTCCGTGA	CTGGGCTGAGCAATACAGTTC
Leptin	GAGACCCCTGTGTCGGTTC	CTGCGTGTGTGAAATGTCATTG
Igf1	GACCAGAGACCCTTTGCGGGG	TGAGGTGCCCTCCGAATGCTG
Enpep	TGCCGGAGCTAAGGAGGCCC	CCACTGGTGGCCGCAAGGTC
Arrpp0	GAAACTGCTGCCTCACATCCG	GCTGGCACAGTGACCTCACACG
**Human**		
Gene	Forward	Reverse
UCP1	CGGCTTTCTTCAAGGGGTTG	GCACAGTCCATAGTCTGCCTT
CARS2	AAGCCGCCTCCTGGTATAG	CTTCCTCATAAAGACTGGCGAG
18S	GTAACCCGTTGAACCCCATT	CCATCCAATCGGTAGTAGCG
**Primers for ChIP**		
Gene	Forward	Reverse
Ucp1	ACCACACCATTTGGAGCCTGAC	TGAGTTTGCAGGGAGGATGGGC
Cars2	CCAGTAGGGGTGCATTTGTG	ACAGGAGCAGCAATGAGTTC

Table S2: List of small hairpin RNA and gDNA sequences.

**Small hairpin RNA**		
**Mouse**		
Gene	Forward	Reverse
shCars2	GCTCTAGCCAATGACTTTGTT<br>CAAGAGACAAAGTCATTGGC<br>TAGAGCTTTTTT	AAAAAAGCTCTAGCCAATGAC<br>TTTGTCTCTTGAACAAAGTCA<br>TTGGCTAGAGC
**gDNA**		
**Mouse**		
Gene	Forward	Reverse
Ebf2	GCTGTGCGCTCAATCTCCAC	GTGGAGATTGAGCGCACAGC
**Human**		
Gene	Forward	Reverse
CARS2	AACTGGATATCCATTCAGGT	ACCTGAATGGATATCCAGTT

Table S3. RNAseq analysis identified the genes that are commonly down-regulated in the BAT of Cars2 AKO and Ebf2 KO mice										
NAME	Cars2_WT1	Cars2_WT2	Cars2_WT3	Cars2_AKO1	Cars2_AKO2	Cars2_AKO3	Ebf2_WT1	Ebf2_WT2	Ebf2_KO1	Ebf2_KO2
Ffar4	944	1225	896	193	216	129	2612	2575	63	51
Lrrc52	352	342	210	51	70	44	61	55	0	2
Pld6	68	70	117	21	19	9	35	43	6	3
Slc27a2	4493	4292	3889	1144	789	657	588	413	3	2
Gm32718	764	987	968	231	216	148	73	85	4	3
Tmx4	1101	1043	1400	360	220	217	260	276	98	131
Clstn3	7366	6252	6791	2623	1381	1180	4596	3330	830	193
St3gal5	2531	2731	2129	767	523	615	362	419	45	68
Slc35g1	166	204	174	68	48	30	43	41	23	28
Cables1	1599	1324	1071	421	353	297	307	301	115	132
Elovl3	3719	3773	4215	1655	654	857	399	572	28	10
Rilpl2	405	424	354	132	114	87	159	179	100	84
Ucp1	131849	123519	112781	51839	34807	24029	137990	122560	131	45
Hoxc5	346	409	301	116	113	89	90	99	36	31
Gm826	91	120	57	24	28	29	17	22	1	1
Cars2	6634	3749	5086	1773	1977	1002	2012	1353	121	112
Ankrd9	2629	2877	2480	939	872	713	2413	1898	103	51
Ctcflos	630	607	654	250	231	170	170	200	52	48
Elovl6	6746	7176	7414	2709	2681	1967	1047	829	101	90
Atxn1	1079	646	615	181	370	267	220	202	125	134
Lgr6	1204	2363	1920	818	461	644	790	1240	13	8
Ctcfl	149	201	192	72	74	51	71	81	26	15
Egln3	131	208	148	54	73	52	452	407	231	160
Gm40011	161	174	158	70	62	51	111	112	76	65
Gm10638	98	151	88	49	25	53	94	94	12	12
Adrbk2	740	1036	1123	396	417	287	278	339	29	26
Atg4d	891	919	897	441	278	331	508	553	275	208
Bbs1	65	60	68	31	21	24	45	42	19	21
Adtrp	606	1073	1086	396	435	266	962	692	100	18
Ppifos	423	405	412	197	140	157	81	84	27	5
Gm9899	2068	2410	2126	1056	758	856	413	276	24	17
Gk	10550	9625	10087	4857	3960	3457	3159	2567	50	38
Cyp2u1	252	217	247	121	72	100	85	110	13	5
Lncbate1	497	565	483	250	216	168	300	246	131	104
Slc12a2	1941	1565	1519	735	646	709	534	615	279	252
Syngr1	134	125	112	53	59	43	88	93	24	20
Pfkfb3	7128	7844	6965	3195	3852	2206	2079	1754	1014	866
Aspa	1007	963	791	410	438	317	470	444	155	93
Mapkapk2	5880	5516	4842	2842	2136	1952	3225	2440	875	712
Cnst	1071	1168	915	410	536	401	369	376	145	189
Gpat4	4845	4687	4115	2057	2269	1523	3503	2871	960	710
Tmem94	648	852	882	340	390	302	285	316	186	188
Lurap1	153	164	158	81	49	78	36	31	14	5
Otop1	1671	2112	1925	1131	694	680	1453	1118	8	4
Slc25a48	26	29	38	17	12	12	14	17	5	6
Flcn	2420	2450	2493	1236	973	1061	386	456	192	207
Gm32817	424	479	431	260	142	193	238	248	14	9
Zfp35	226	198	224	97	101	92	125	144	61	68
P4ha1	1662	1614	1634	885	630	690	336	363	189	192
Leng9	61	78	45	34	24	25	144	132	29	17
Jmjd4	144	142	130	71	62	56	64	60	25	28
Slc25a17	2026	1953	1844	954	860	840	1073	931	390	389
Tmem79	427	527	504	252	213	198	123	151	19	22
Gm34921	657	500	552	333	259	196	244	250	147	128
Pank1	4505	4271	4196	2291	1879	1835	1478	1109	48	35
Zfp672	692	794	659	409	317	271	309	323	177	115
4732440D04I	84	123	118	49	50	52	43	45	8	11
Pnp	1318	1411	1145	728	589	492	925	1098	362	295
Cxadr	707	601	737	326	302	331	170	163	6	6
Adra1a	1296	1358	1545	683	761	540	325	260	27	32
Tmem186	279	360	319	194	144	115	170	173	66	67
Gm33838	28	34	35	14	12	21	13	14	5	3
Etfdh	6616	7203	6506	3213	3480	2943	7462	5587	781	756
Exoc8	379	407	351	230	150	161	232	219	155	128
Nudt15	105	121	136	57	66	50	28	21	4	3
Uck1	1470	1995	2085	1022	969	658	1166	1304	658	566
5031439G07I	1096	1118	1202	741	395	495	819	606	207	184
Acot11	2108	3570	3600	2010	1131	1370	101	156	8	10
Iba57	255	254	222	141	101	114	249	224	98	66
Dhrs9	1139	1196	1287	670	544	554	78	49	1	2
Pde4d	3319	3022	3228	1131	2331	1214	914	697	230	217
Fbxl15	72	74	73	46	32	28	107	114	52	42
Slc4a4	3551	3575	3260	1596	1755	1766	1326	1167	109	121
Adrb1	258	212	216	141	97	101	112	106	22	13
Gm36393	207	211	213	85	100	128	60	47	1	0
Col27a1	542	478	825	311	330	273	682	525	111	64
Ptger1	2801	2882	2849	1900	1225	1119	1938	1883	414	349
Ric1	770	723	835	369	435	355	186	143	61	72
Rilp	321	418	378	216	159	182	303	391	110	116
Samd4b	1317	1132	1098	721	591	465	330	353	206	235
Plin5	1397	1683	1468	948	682	656	1830	1412	366	132
Tmem37	574	711	573	373	250	315	509	389	142	74
Gnao1	619	458	607	267	297	286	167	151	24	30
Egln1	1600	2526	1894	1101	981	973	1626	1985	662	553
Acss1	2112	3163	2801	1354	1521	1243	913	926	129	95
Arhgef12	3217	3080	2923	1608	1629	1470	1277	996	454	472
Wdr90	55	55	37	25	22	28	54	51	31	30

Acot8	316	275	246	170	138	124	195	168	106	83
Rundc3a	144	148	155	82	67	81	147	180	58	35
Acacb	6088	7616	7510	4335	3431	3182	1997	1523	466	228
Tecpr1	1613	2011	1629	1054	751	907	689	813	298	225
Timm9	510	512	442	308	244	204	333	331	195	163
Ppp1r3c	831	957	658	382	388	492	309	250	150	142
Cox8b	5233	6541	5686	3189	3214	2613	19409	13309	2865	788
Mtfp1	496	563	488	340	220	242	511	420	64	42
Tmed5	3910	3252	3672	1722	2036	1862	3777	3739	735	630
Cgrrf1	536	593	493	337	286	219	334	285	189	197
Zfp961	133	149	184	92	69	81	73	75	45	52
Slc36a2	5904	5831	5117	3168	3191	2422	5021	3870	1590	1389
Dnajc27	252	268	251	156	127	119	73	77	37	37
Letm1	2542	3132	2785	1799	1342	1295	1899	1586	565	464
Tgoln1	5599	5688	5382	3094	2890	2778	805	739	536	446
Smcr8	844	839	716	458	408	397	188	171	99	105
Gja1	3622	2836	2732	1533	1981	1341	2170	1818	226	255
Mlycd	831	1151	885	613	437	466	1210	1099	530	360
4931406C07F	4841	4969	5414	3139	2601	2388	2016	1776	825	717
Tbc1d9	172	192	198	102	104	97	138	148	33	31
Spsb3	494	552	576	331	248	297	514	444	155	107
Pkn1	4170	4527	4196	2998	2039	1937	2569	2773	466	438
Fastkd5	312	271	282	188	143	138	61	62	33	28
Fuom	583	709	623	414	358	270	1077	977	342	182
Akr1b3	1605	1494	1337	916	759	747	692	697	422	345
Cluh	8313	8913	8436	5376	4244	4396	4789	4548	2169	1251
Gde1	1746	1967	1693	1104	1005	845	1059	937	448	379
Pgap3	38	25	35	19	16	18	57	63	38	28
Tstd3	174	198	241	128	97	111	189	200	34	54
Arfgef2	1210	1275	1310	732	604	743	502	407	211	197
Pfkp	3087	3477	2953	1956	1593	1663	949	1234	266	291
Dnajc11	1310	1436	1258	891	652	661	879	799	299	158
Smpd1	1469	1837	1482	1006	816	814	912	819	489	395
Armc1	836	846	836	504	450	436	528	465	282	234
Ndrg3	2338	2391	2200	1418	1236	1178	342	381	197	181
Nhlrc1	57	37	44	30	16	30	51	51	15	14
Frrs1	245	237	219	161	104	124	90	83	47	38
Slc22a5	1018	1059	904	605	656	394	113	122	83	72
Bet1l	354	346	302	228	171	158	429	405	261	222
Slc25a51	4939	5724	5263	3388	3068	2402	3682	2672	735	656
Poldip2	1840	2013	1712	1163	1076	859	2233	1815	827	703
Amd1	359	309	338	204	198	159	69	74	40	45
Smpd5	247	289	235	149	141	142	328	291	87	57
Unc119b	635	658	701	477	263	381	174	185	107	118
Rnf152	371	325	397	200	193	222	346	238	28	51
Slc25a20	5011	5740	4972	3462	2902	2526	5462	5735	915	476
Abcd3	1850	2249	2242	1207	1289	1120	1370	1321	369	448
Cfap157	36	33	26	23	12	20	19	17	10	10
Ptdss2	546	609	511	368	269	313	552	604	240	213
Pgk1	7382	7091	7028	4472	4040	3768	3757	3321	872	778
Dlg1	988	957	1095	567	602	568	576	473	200	183
Car14	167	246	174	98	142	97	125	144	85	83
Rps6kl1	149	177	152	104	92	78	149	130	50	36
Ppp1r15b	2415	2369	2200	1638	1293	1102	971	1059	528	439
Pgam1	4457	5063	4430	3116	2429	2515	997	1014	180	90
Dusp6	1596	1329	1394	966	820	710	315	342	165	153
Zfp870	73	66	84	38	46	46	78	66	44	43
Taco1	340	356	344	195	184	225	111	85	36	23
Atg2b	587	598	500	387	292	303	155	156	85	106
Gnas	27235	33453	28532	18487	18006	15652	53590	48398	12382	12028
Ei24	2184	1963	1670	1116	1322	964	1551	1293	708	645
Acads	2865	3634	3124	2167	1831	1636	3988	4324	1433	1075
Mlst8	191	201	236	155	102	112	197	232	104	93
Tbrg4	934	1039	949	665	539	518	1504	1140	444	240
Gm36117	63	43	64	37	27	36	34	33	5	9
Smg5	848	762	907	603	422	471	632	767	373	268
Pomgnt1	1022	1142	941	674	606	568	451	425	303	246
Agpat3	4651	5220	4928	3444	2849	2527	5464	4831	1876	1349
Phospho2	369	351	412	265	189	221	138	132	63	71
Ppme1	753	742	628	533	384	350	204	226	117	95
Bricd5	117	118	139	78	66	79	128	161	59	59
Vamp2	695	795	745	444	460	434	823	736	541	485
Adat2	125	142	136	79	79	84	99	114	41	35
Usp19	1521	1552	1631	1072	795	957	754	810	470	428
Ndufa3	1984	2283	1912	1362	1242	1115	5407	4277	2233	1875
Oplah	1895	2472	2123	1389	1261	1258	1915	2278	451	242
Acad8	357	453	389	262	251	212	498	500	329	295
Them4	203	204	206	153	91	127	102	111	38	53
Gfm1	2143	2158	2144	1456	1270	1177	846	731	269	212
Cmtm4	965	1251	1048	689	719	574	522	455	244	182
Sgpl1	5095	4816	3340	2558	2652	2854	1439	1569	567	426
Atp6v0c	892	879	683	655	420	427	565	496	266	166
Mief1	429	438	356	293	233	225	259	234	161	119
Ogdh	22107	26823	23860	16003	14132	14591	7819	8289	1713	1431
Letmd1	5249	6493	5882	4248	3302	3348	4380	3737	375	234
Dtnbp1	1278	1503	1236	956	790	745	2040	1623	913	746
Tufm	2230	2560	2387	1566	1514	1400	1860	1527	614	419
Ap1g1	1141	1017	1158	718	710	642	563	476	322	311
Acat1	5582	7125	6605	3932	4602	3539	5241	6028	2048	2072

D17H6S53E	163	172	198	130	108	95	114	131	45	42
Tecpr2	242	244	214	165	125	148	82	78	29	38
Aph1a	767	786	634	489	448	435	581	717	318	214
Poc1a	52	50	69	41	38	28	87	81	22	23
Ldhb	6887	7550	6810	4713	4721	3926	6082	5220	609	351
Kcnk3	8625	11255	9032	7031	4922	6240	4488	4533	693	391
LOC1081691	21	19	23	13	14	13	16	15	8	8
Pfkl	4698	6126	4721	3876	2886	3036	4624	5365	1484	715
9130221H12I	1642	1551	1510	1199	834	936	641	663	435	305
Ciapin1	859	1084	998	710	561	586	1145	1124	297	197
Gtf2ird1	1035	1004	937	749	513	618	328	401	187	208
Wdtc1	1061	1023	716	604	547	620	319	333	172	186
Spata2	243	247	213	184	129	132	137	139	87	65
Mcee	596	785	700	430	462	426	1151	1123	424	378
Cdk17	427	417	425	283	260	267	295	274	118	117
Sdhd	3381	3864	3413	2403	2352	2060	9361	6891	2343	1861
D10Jhu81e	1826	2601	2028	1434	1420	1276	1949	1675	987	745
Gsk3a	1512	1448	1286	1138	769	814	1045	1264	649	489
Rmdn3	2141	2164	1969	1654	1089	1284	1105	918	285	309
Slmo2	2096	2397	2177	1611	1394	1301	2876	2855	470	318
Cox7a1	1846	2379	1880	1297	1432	1215	8201	8915	213	285
Dhrs4	697	937	827	577	563	453	1498	1289	637	413
Sdr39u1	692	776	740	531	419	485	956	758	301	243
Rhot2	650	848	724	575	414	458	674	709	231	174
Syp	95	130	102	87	65	62	149	106	25	34
Zfp317	165	145	148	83	103	114	129	113	71	72
Hadhb	16410	21018	18077	12056	12733	11588	13327	11541	1866	1321
Acadl	14199	16419	14823	9709	11330	8825	17139	16854	3175	3456
Hmgxb3	437	362	339	297	204	248	146	171	89	85
Ttc1	540	476	510	350	347	309	300	281	188	200
AW209491	237	215	205	158	120	156	97	90	31	37
Naa40	412	369	378	306	218	242	236	263	138	132
Aste1	191	193	208	137	124	130	134	116	33	26
Tmem185b	174	182	144	130	100	101	114	112	70	78
Ssfa2	1472	1547	1365	972	961	973	321	268	134	147
Khdrbs3	577	584	580	429	392	334	278	262	31	44
Zfp653	116	115	129	86	85	68	166	168	79	67
Ppm1m	728	994	906	617	637	499	682	700	316	179
Dhrs11	369	440	370	280	252	255	463	438	181	112
LOC1081689	578	620	461	453	358	296	561	518	302	247
Gm5785	167	168	130	98	120	92	143	110	45	25
Zfp213	103	94	82	72	58	57	61	67	24	28
Rab21	524	538	557	364	361	358	296	280	173	189
Bap1	833	809	747	590	521	488	567	491	313	272
