# Supplementary material for: Cars2‐Mediated Cysteine Catabolism Drives Brown Fat Development and Thermogenesis Through Persulfidating EBF2
Source: Adv Sci (Weinh). 2026 Mar 18;13(31):e22690. doi: 10.1002/advs.202522690 (PMC13588024; doi:10.1002/advs.202522690)
Supplement: Supplementary file 3 [file ADVS-13-e22690-s002.docx]

**Supplemental Information**

**Supplemental Method**

**Mass spectrometry analysis of persulfidated Ebf2**

293T cells were infected with retrovirus expressing scramble or *CARS2*-targeted guide RNA and selected with puromycin to acquire vector control (293T-V) and *CARS2* knockout (293T-KO) cells. Ebf2-Flag-overexpressing plasmids were transfected into 293T-V and 293T-KO cells. Forty-eight hours later, the cells were lysed with RIPA buffer supplemented with 1 mM N-iodoacetyl-L-tyrosine methyl ester (TME-IAM) (HEOWNS, N-0447807). After sonication and centrifugation to remove cell debris, the supernatants were transferred to new tubes and incubated at 1000 rpm and 37°C for another 1 h in thermo mixer to allow the TME-IAM label to -SH and -SSH of cysteine to form -S-AM-TME and -S-S-AM-TME. The proteins were then precipitated by adding 2.5 times the volume of -20°C precooled acetone for 15 min at -20°C, followed by centrifugation at 3100 rpm for 5 min to pellet the protein and washing twice with 70% acetone to remove the excess TME-IAM. The pellets were resuspended in HENS buffer (250 mM HEPES-NaOH pH 7.7, 1 mM EDTA, 0.1 mM neocuproine, 1% SDS), and 2 mg of total protein was used for EBF2-Flag immunoprecipitation by incubation with Flag-antibody-conjugated agarose (Sigma, A2220). The protein-binding agarose was subsequently resuspended in 1x loading buffer and boiled at 95°C for 5 minutes, after which the supernatants were subjected to LC‒MS/MS analysis. Briefly, the samples were separated by SDS-polyacrylamide gel electrophoresis and visualized with colloidal coomassie blue. The EBF2 bands from the gels were cut into 1 mm slices, and each slice was washed twice with 50 mM NH_4_HCO_3_ and 50% acetonitrile (ACN) and dehydrated with ACN. DTT (10 mM) and iodoacetamide (55 mM) were used for protein reduction and alkylation, respectively. After DTT reduction, the -S-S-AM-TME was reduced to -SH. Proteins were then washed with 50 mM NH_4_HCO_3_ and ACN and subjected to gel digestion with sequencing grade trypsin (Promega, V511C) overnight at 37°C, followed by extraction of tryptic peptides from the gel pieces with 0.1% trifluoroacetic acid and 60% ACN. The extracted peptides were dried by vacuum centrifugation and dissolved in 10 μl of 2% ACN and 0.1% trifluoroacetic acid. Afterwards, a Michrom peptide CapTrap (MW 0.5–50 kDa, 0.5 × 2 mm; Michrom BioResources, Inc.) was used to desalt and preconcentrate the samples. The eluent was dried under vacuum and reconstituted in 2% ACN and 0.1% formic acid. The MS spectrum was acquired via a hybrid linear ion trap (LTQ) Orbitrap mass spectrometer (ThermoFinnigan) assembled with ADVANCE Spray Source (Michrom Bioresources) with the following parameters. A high-resolution MS survey scan (R = 100,000 at m/z 400) was obtained for m/z 350–1800, with 106 ion accumulations of automatic gain control. Siloxane (m/z 445.120025) was used as an internal standard to calibrate the mass accuracy.

**Bodipy staining of lipid droplets**

Discard the culture medium of differentiated brown adipocytes, washed once with PBS and incubated with complete culture medium supplemented with 5μM BODIPY 493/503 (MedChemExpress, HY-W090090) at 37 °C for 30 min. Cells were then washed three times with PBS, fixed with 4% formalin, and the diameter of lipid droplets were analysed by using a custom image analysis pipeline implemented in Python. To ensure robust and conservative quantification, only well-defined, near-circular lipid droplets were included for further analysis. Objects were filtered based on the following criteria: (1) minimum area threshold (diameter< 5 µm) to exclude small noise particles; (2) circularity ≥ 0.75, calculated as 4π×area/perimeter^2^; (3) an upper limit on equivalent diameter (≤ 30 µm) to exclude fused or irregular structures.

**Oxygen bomb calorimetry of feces**

Calorimetry was conducted using a Parr 6400 Calorimeter. Fecal samples from mice were collected over a 24 h period. Samples were baked at 60°C for 24 h to remove water content. Fecal samples were ignited and the energy content was measured as heat of combustion (kilo calories/g).

**Supplemental Figures**

**Figure S1. Cysteine catabolism is regulated by Cars2 but not Cbs/Cse in brown adipocytes and is associated with thermogenic gene expression in brown adipocytes**

(A) Oil Red O staining was performed in mouse brown adipose cells (BAC) and 3T3-L1 cells after 6 and 8 days of differentiation, respectively. (B) After differentiation, the culture medium of BAC (n=3) was deprived of cystine for 24 h. Intracellular cysteine levels were measured using a commercial kit. (C) Oil Red O staining was performed in the cells described in B. (D) The viability of the cells described in B was determined using the CCK-8 assay. (E) The relative amounts of UCP1 in Figure 1F were determined by the density of the bands of UCP1, normalized to that of HSP90. (F) Plasma cysteine levels were measured using a commercial kit in mice (n=4) under feeding (Fed) or 16 h fasting (Fast) conditions. (G) A diagram depicting the cysteine metabolic pathway. (H) The culture medium of BAC (n=6) was deprived of cystine for 24 h after differentiation. The hydrogen sulfide and sulfane sulfur levels were measured by AzMC and SSP4 probes, respectively. (I) The mitochondrial content, hydrogen sulfide and sulfane sulfur levels of the mouse BAC after differentiation were stained with MitoTracker, AzMC and SSP4 respectively; the nucleus was stained with Hoechst 33342 (for SSP4) and SYTO9 (for AzMC); and fluorescence images were taken via confocal microscopy. Scale bar=5 µm. (J) Total GSH content of mouse BAC (n=3) before (Day 0) and after (Day 6) differentiation. (K-L) Mouse BAC (n=3) was treated with taurine (200 µM) during differentiation (day0-day6), and browning genes in the mature BAC treated with vehicle (Veh) or norepinephrine (NE, 1 µM, 6 h) were subsequently examined via qPCR (K) and the protein of UCP1 was determined via western blot (L, left panel), the relative amounts of UCP1 were determined by the density of the bands of UCP1, normalized to that of HSP90 (L, right panel). (M)The expression of *Cbs* and *Cse* in mouse fat and liver tissues (n=5) was determined by qPCR. (N-O) Mouse BAC was treated with propargylglycine (PAG, 500 µM) during differentiation (day0-day6). The hydrogen sulfide and sulfane sulfur levels were measured by AzMC and SSP4 probes (n=9) after differentiation (N), and the protein expression of UCP1 was analyzed by western blotting after treated with vehicle (Veh) or norepinephrine (NE, 1 µM, 6 h) (O, left panel), the relative amounts of UCP1 determined by the density of the bands of UCP1, normalized to that of HSP90 (O, right panel) . Data are shown as the mean ± SEM. **p < 0.01, and ***p < 0.001, by unpaired Student’s t test (B, D, E, F, H, J, K, L, N and O); by one-way ANOVA with dunnett's test (M); ns, nonsignificant.

**Figure S2. Cars2 is induced by β-adrenergic signaling in fat tissues and is required for maintaining body temperature, whereas *Cars2* deficiency does not affect mitochondrial oxidative respiration in iBAT**

(A) QPCR analysis of *Cars2* in the liver, iBAT, muscle and heart of mice kept at room temperature (n=3). (B) The relative amounts of CARS2 and UCP1 in Figure 2F were determined by the density of the corresponding bands, normalized to that of HSP90. (C) The relative amounts of CARS2 and UCP1 in Figure 2H were determined by the density of the corresponding bands, normalized to that of HSP90. (D) The relative amounts of persulfidated protein in Figure 2I (left panel) and Figure 2J (right panel) were determined by the density of the bands of persulfidated protein in biotin-HPDP(+) lane minus the bands of persulfidated protein in biotin-HPDP(-) lane, normalized to that of HSP90. Then, the fold changes were calculated by dividing the relative persulfidated protein in the day 6 and day10 by that in day 0. (E) The relative amounts of UCP1 and CARS2 in Figure 2L were determined by the density of the corresponding bands of protein, normalized to that of HSP90, Then, the fold changes were calculated by dividing the relative density of protein in the chronic cold (CC) and CL316243 treatment (CL) by that in room temperature (RT). (F) QPCR analysis of *Cars2* in the inguinal white adipose tissue (iWAT) and epididymal white adipose tissue (eWAT) of mice kept at room temperature (RT, n=4), subjected to chronic cold (CC, n=4) or treated with CL for 7 days (n=4). (G-H) The relative amounts of CARS2 in Figure 2O (G) and Figure 2Q (H) were determined by the density of the bands of CARS2, normalized to that of HSP90. (I) QPCR analysis of *Cars2* in iBAT, iWAT and eWAT from *Cars2* Adiponectin-Cre knockout (AKO, n=7, left) and Ucp1-Cre knockout (UKO, n=4, right) mice and controls (Flox, AKO, n=7, UKO, n=5) after acute cold exposure. (J) The rectal temperatures of *Cars2* AKO and UKO mice and controls in C were measured at the indicated time points during acute cold exposure. (K-L) H&E staining of eWAT, iWAT and liver of *Cars2* AKO (K) and UKO (L) mice and controls in Figure 3I after cold exposure. Scale bar=50 µm. (M-P) Mitochondrial DNA (M-N), cristae number and mitochondria number (O-P) in the iBAT from Cars2 AKO and UKO mice and controls. Data are shown as the mean ± SEM. *p < 0.05, **p < 0.01, and ***p < 0.001, by unpaired Student’s t test (left panel of D, G-I, M-P); by one-way ANOVA with dunnett's test (A-C, right panel of D, E, and F); by two-way ANOVA with multiple comparisons (J); ns, nonsignificant.

**Figure S3. *Cars2* deficiency reduces energy expenditure but does not influence food intake, activity and energy excretion**

(A-B) Energy expenditure of *Cars2* AKO (A, n=8) and UKO (B, n=8) mice compared with those of controls (Flox, n=7) were measured via metabolic cage experiments.(C-D) Food intake (C) and activity (D) of *Cars2*-AKO and Flox mice used in A. *Cars2*-AKO and Flox mice were maintained on a chow diet (n=7 per group). (E-F) Fecal energy density (E) and daily fecal output (F) were determined. (G-H) Food intake (G) and activity (H) of *Cars2*-UKO and Flox mice used in B. (I) Fat mass and lean mass of *Cars2* AKO (n=8) and control mice (n=7). Data are shown as the mean ± SEM. P value was measured by unpaired Student’s t test (C-I); by two-sided ANCOVA analysis (A and B); ns, nonsignificant.

**Figure S4. Knockout of *Cars2* promotes whitening gene expression and fibrogenesis in the iBAT, while ATP production is not affected**

(A-B) QPCR analysis of whitening genes in the iBAT from *Cars2* AKO (A, n=7) and UKO (B, n=4) mice and controls (Flox, AKO, n=7, UKO, n=5) after acute cold exposure for 4 h. (C-D) The expression of collagen-associated genes in the iBAT of *Cars2* AKO and UKO mice and controls used in Figure 3 C-D after cold exposure were determined by qPCR. (E-F) Sirius red staining of iBAT from *Cars2* AKO and UKO mice and control mice after cold exposure. Scale bar=50 µm. (G-H) The mRNA levels of browning genes in the iWAT (G) and eWAT (H) of *Cars2* AKO and UKO mice and the controls used in Figure 3C-D after cold exposure were determined by qPCR. (I-J) The relative amounts of protein from the iBAT indicated in western blotting of Figure 3Y (I) and 3Z (J) were determined by the density of the corresponding bands of protein, normalized to that of HSP90, Then, the fold changes were calculated by dividing the relative density of protein in the AKO and UKO by that in Flox. (K-L) ATP production in adipose tissues of *Cars2* AKO and UKO mice compared with that in controls. Data are shown as the mean ± SEM. *p < 0.05, **p < 0.01, and ***p < 0.001, by unpaired Student’s t test (A-D and G-L); ns, nonsignificant.

**Figure S5. Thermoneutral treatment diminishes the metabolic differences between *Cars2* Adiponectin-Cre knockout and control mice.** The *Cars2* Adiponectin-Cre knockout (AKO, n=6) mice and controls (Flox, n=6) were housed at ambient thermoneutral temperature (30℃) for 4 weeks, and then subjected to metabolic cage experiments at 30℃. (A-B) Body weights (A), morphology and H&E staining (B) of iBAT from *Cars2*-AKO and controls after thermoneutral treatment. Scale bar=100 µm. (C) The mRNA levels of browning genes in the iBAT of *Cars2*-AKO and the controls after thermoneutral treatment were determined by qPCR. (D-H) Oxygen consumption curves (VO_2_, D), VO_2_ and energy expenditure regression plots (E-F), food intake (G) and activity (H) of *Cars2*-AKO and controls housed at ambient thermoneutral temperature (30℃) were measured by metabolic cages at 30℃. (I-J) Plasma triglyceride (TAG, I) and nonesterified fatty acid (NEFA, J) of *Cars2* AKO and controls after thermoneutral treatment. Data are shown as the mean ± SEM. ***p < 0.001, by unpaired Student’s t test (A, C and G-J); by two-sided ANCOVA analysis (E and F); ns, nonsignificant.

**Figure S6. Overexpressing of Cars2 in BAC promotes H_2_S and RSSH production and thermogenic gene expression**

(A-B) Hydrogen sulfide and sulfane sulfur levels in vector control (Vec) and Cars2-overexpressing (Cars2) mouse BAC after differentiation were measured via AzMC (n=6) and SSP4 (n=6~9) probes. (C) The content of persulfidated protein in vector control (Vec) and Cars2-overexpressing mouse BAC after differentiation was determined by western blot after the biotin-HPDP switch assay. (D) The relative amounts of persulfidated protein in C were determined by the density of the bands of persulfidated protein in biotin-HPDP(+) lane minus the bands of persulfidated protein in biotin-HPDP(-) lane, normalized to that of HSP90. Then, the fold changes were calculated by dividing the relative persulfidated protein in the Cars2-overexpressing BAC by that Vec-expression BAC. (E-F) The mRNA and protein expression levels of browning genes in Vec (n=3) and Cars2-overexpressing (Cars2, C2, n=3) BAC during differentiation were analyzed by qPCR (E) and western blotting (F). (G) The relative amounts of protein in F were determined by the density of the corresponding bands of protein, normalized to that of HSP90, Then, the fold changes were calculated by dividing the relative density of protein in the Cars2-overexpressing BAC by that Vec-expression BAC. (H-I) The mRNA (H, n=3) and protein (I) expression levels of browning genes in vector- and Cars2-overexpressing mouse BAC treated with vehicle (Veh) or norepinephrine (NE, 1 µM, 6 h) after differentiation were analyzed by qPCR and western blotting. (J) The relative amounts of protein in I were determined by the density of the corresponding bands of protein, normalized to that of TUBULIN, Then, the fold changes were calculated by dividing the relative density of protein in the Cars2-overexpressing BAC by that Vec-expression BAC. (K) Lipid droplets and mitochondria in vector- and Cars2-overexpressing mouse BAC after differentiation were stained with Oil red O and MitoTracker dye. Scale bar=50 µm.(L-M) Lipid droplets in vector- and Cars2-overexpressing mouse BAC after differentiation were stained with Bodipy dye (L) and the area distribution of lipid droplets were calculated (M); Scale bar=100 µm.(N) Oxygen consumption rates of Vec- and Cars2-overexpressing BAC (n=6) treated with FCCP (10 µM) and oligomycin (Oligo, 10 µg/ml). Data are shown as the mean ± SEM. *p < 0.05, **p < 0.01, and ***p < 0.001, by unpaired Student’s t test (A, B, D, G, H, J, M and N); by two-way ANOVA with multiple comparisons (E); ns, nonsignificant.

**Figure S7. Knockdown of Cars2 in BAC attenuates H_2_S and RSSH production and thermogenic gene expression**

(A-B) Hydrogen sulfide and sulfane sulfur levels in mouse BAC (n=3) expressing small hairpin RNA targeting *Cars2* (shC2) or the scramble sequence (Scr) were measured by AzMC and SSP4 probes. (C) The content of persulfidated protein in scramble (Scr)- and short hairpin RNA (shRNA)-targeting *Cars2* (shC2)-expressing BAC after differentiation was determined by western blotting after the biotin-HPDP switch assay. (D) The relative amounts of persulfidated protein in C were determined by the density of the bands of persulfidated protein in biotin-HPDP(+) lane minus the bands of persulfidated protein in biotin-HPDP(-) lane, normalized to that of HSP90. Then, the fold changes were calculated by dividing the relative persulfidated protein in the sh-C2 BAC by that Scr BAC. (E-F) The mRNA and protein expression levels of browning genes in Scr- and shC2-expressing BAC (n=3) during differentiation were analyzed via qPCR (E) and western blotting (F).(G) The relative amounts of protein in F were determined by the density of the corresponding bands of protein, normalized to that of TUBULIN, Then, the fold changes were calculated by dividing the relative density of protein in the sh-C2 BAC by that Scr BAC. (H-I) The mRNA (H) and protein (I) expression levels of browning genes in Scr- and shC2-expressing mouse BAC (n=3) treated with Veh or NE (1 µM) after differentiation were analyzed by qPCR and western blotting. (J) The relative amounts of protein in I were determined by the density of the corresponding bands of protein, normalized to that of TUBULIN, Then, the fold changes were calculated by dividing the relative density of protein in the sh-C2 BAC by that Scr BAC. (K) Lipid droplets and mitochondrial content in Scr- and shC2-expressing mouse BAC after differentiation were stained with Oil red O and MitoTracker dyes. Scale bar=50 µm. (L) Oxygen consumption rates of Scr- and shC2-expressing BAC treated with FCCP (10 µM) and oligomycin (Oligo, 10 µg/ml, n=3~5). Data are shown as the mean ± SEM. **p < 0.01, and ***p < 0.001, by unpaired Student’s t test (A, B, D, G, H, J and L); by two-way ANOVA with multiple comparisons (E); ns, nonsignificant.

**Figure S8. Cars2 cell-****autonomously regulates BAC differentiation and thermogenic gene expression and uncoupled respiration in mature adipocytes**

(A-B) Hydrogen sulfide (A) and sulfane sulfur (B) levels were measured by AzMC and SSP4 in differentiated primary BAC isolated from the iBAT of newborn *Cars2*^flox/flox^ mice expressing Vec or Cre recombinase in mature adipocytes (n=6~7). (C) The content of persulfidated protein in BAC from *Cars2*^flox/flox^ mice expressing Vec and Cre recombinase in precursor cells (Cre-pre) and Cre recombinase in mature adipocytes (Cre-mat) was determined after differentiation by western blot after the biotin-HPDP switch assay.(D) The relative amounts of persulfidated protein were determined by the density of the bands of persulfidated protein, normalized to that of HSP90. Then, the fold changes were calculated by dividing the relative persulfidated protein in the Cre-pre and Cre-mat BAC by that of Vec-expressing BAC. (E-F) The protein and mRNA expression levels of browning genes in BAC from *Cars2*^flox/flox^ mice expressing Vec (n=3) and Cre in precursor cells (Cre-pre, n=3) and Cre in mature cells (Cre-mat, n=3) treated with vehicle (Veh) or norepinephrine (NE, 1 µM, 6 h) after differentiation were analyzed via qPCR (E) and western blotting (F). (G) The relative amounts of protein in F were determined by the density of the corresponding bands of protein, normalized to that of HSP90, Then, the fold changes were calculated by dividing the relative density of protein in the Cre-pre and Cre-mat BAC by that of Vec- expressing BAC. (H) Oxygen consumption rates of Vec-, Cre-pre- and Cre-mat-expressing *Cars2*-floxed BAC (n=3) treated with FCCP (10 µM) and oligomycin (Oligo, 10 µg/ml) after differentiation. (I) Lipid droplets and mitochondrial content in *Cars2*^flox/flox^ mouse BAC expressing Vec and Cre recombinase in precursor cells (Cre-pre) and Cre recombinase in mature adipocytes (Cre-mat) were stained with Oil red O and MitoTracker dye after differentiation. Scale bar=50 µm. Data are shown as the mean ± SEM. ***p < 0.001, by unpaired Student’s t test (A and B); by one-way ANOVA with dunnett's test (D, E, G and H); ns, nonsignificant.

**Figure S9. Cars2 mediates thermogenic gene expression through its CPERS activity**

(A-B) Cars2 wild-type (WT) and lysine-to-alanine (109,112,303,306) mutant (4KA)-expressing plasmids or vector (Vec) controls were transfected into wild-type (WT) or *CARS2* knockout (*CARS2*-KO) 293T cells, and the protein levels of MTCO1 and CARS2 were measured by western blotting (A) and the relative amounts of MTCO1 and CARS2 were determined by the density of the corresponding protein bands, normalized to that of HSP90, Then, the fold changes were calculated by dividing the relative density of protein in the *CARS2*-KO 293T cells expressing vector, wild type CARS2 and 4KA mutant by that of Vec-expressing control 293T cells (B). (C) The relative amounts of persulfidated protein in Figure 4B were determined by the density of the bands of persulfidated protein, normalized to that of HSP90. Then, the fold changes were calculated by dividing the relative persulfidated protein in the *Cars2*-KO BAC expressing vector, wild type CARS2 and 4KA mutant by that of Vec-expressing control BAC. (D) The relative amounts of protein in Figure 4D were determined by the density of the corresponding bands of protein, normalized to that of HSP90, Then, the fold changes were calculated by dividing the relative density of protein in the *Cars2*-KO BAC expressing vector, wild type CARS2 and 4KA mutant by that of Vec-expressing control BAC. (E) The exogenous expression of Cars2 in iBAT, iWAT, eWAT and liver from *Cars2* AKO mice injected with AAV viruses expressing Cars2-WT or vector controls into the iBAT (n=5 for each group) were determined via qPCR (left panel) and western blot (middle); The relative amounts of Cars2-Flag in middle panel was determined by the density of the FLAG bands of protein, normalized to that of HSP90. Then, the fold changes were calculated by dividing the relative density of FLAG in iBAT, iWAT, eWAT and liver from *Cars2*-AKO mice injected with AAV expressing wild type Cars2 by that of AKO mice injected with AAV expressing Vector (Vec) respectively (right panel). (F) The relative amounts of protein in Figure 4L were determined by the density of the corresponding bands of protein, normalized to that of HSP90. Then, the fold changes were calculated by dividing the relative density of protein of iBAT from *Cars2*-AKO mice injected with AAV expressing Vector, wild type CARS2 and 4KA mutant by that of Flox mice injected with AAV expressing Vector (G-H) Mouse BAC (n=3) was treated with pyridoxal 5-phosphate (PLP, 100 µM) during differentiation (day0-day6), and the expression of browning genes in mature BAC was analyzed by qPCR (G) and western blotting (H) after treated with vehicle (Veh) or norepinephrine (NE, 1 µM, 6 h). (I) The relative amounts of UCP1 protein in G were determined by the density of UCP1 bands, normalized to that of TUBULIN, Then, the fold changes were calculated by dividing the relative density of UCP1 in PLP treated BAC by that of Vehicle treated BAC. (J-K) Primary BAC isolated from the iBAT of newborn *Cars2*^flox/flox^ mice expressing vector (Vec, n=3) or Cre recombinase (n=3) in precursor cells were treated with PLP (100 µM) during differentiation (day0-day6), and the expression of browning genes was analyzed after treated with vehicle (Veh) or norepinephrine (NE, 1 µM, 6 h) by qPCR (J) and western blotting (K) after differentiation. (L) The relative amounts of UCP1 protein in K were determined by the density of UCP1 bands, normalized to that of HSP90, Then, the fold changes were calculated by dividing the relative density of UCP1 in PLP treated BAC by that of Vehicle treated BAC. Data are shown as the mean ± SEM. *p < 0.05, **p < 0.01, and ***p < 0.001, by unpaired Student’s t test (E,G, I, J and L); by one-way ANOVA with dunnett's test (B-D and F); ns, nonsignificant.

**Figure S10. Mapping EBF2 persulfidation site in 293T cells**

(A) Diagram depicting the mass spectra protocol used to identify the persulfidation site of EBF2 with a FLAG tag overexpressed in wild-type and *CARS2*-KO 293T cells (upper panel), and the abundance of peptides containing cysteine labeled with TME-IAM is presented with a heatmap (lower panel). (B) Plasmids expressing wild-type or mutant Ebf2 with a Flag tag were transfected into 293T cells, and persulfidated EBF2 was purified via the biotin-HPDP switch assay and determined via western blot analysis. (C) The relative amounts of persulfidated EBF2 in B were determined by the density of biotin immunobloting bands of immunoprecipitated EBF2, normalized to the density of EBF2 immunobloting bands of immunoprecipitated EBF2. Then, the fold changes were calculated by dividing the relative density of persulfidated EBF2 in EBF2-mutant expressing 293T cells by persulfidated EBF2 in wild type EBF2 expressing 293T cells. (D) The relative amounts of persulfidated EBF2 in Figure 5F were determined by the density of biotin immunobloting bands of immunoprecipitated EBF2, normalized to the density of EBF2 immunobloting bands of immunoprecipitated EBF2. Then, the fold changes were calculated by dividing the relative density of persulfidated EBF2 in EBF2-mutant expressing mouse BAC cells by persulfidated EBF2 in wild type EBF2 expressing BAC cells after differentiation. Data are shown as the mean ± SEM. **p < 0.01, and ***p < 0.001, by one-way ANOVA with dunnett's test (C and D); ns, nonsignificant.

**Figure S11. CARS2 controls EBF2 persulfidation and its interaction with PPARγ and BRG1**

(A) Ebf2 with a Flag tag was overexpressed in differentiated primary BAC isolated from the iBAT of newborn *Cars2*^flox/flox^ mice expressing vector (Vec) or Cre recombinase in precursor cells, which were pulled down with Flag-agarose after the biotin-HPDP switch assay was performed, and the persulfidation of EBF2 was analyzed by western blotting. (B) The relative amounts of persulfidated EBF2 in A were determined by the density of biotin immunobloting bands of immunoprecipitated EBF2, normalized to the density of EBF2 immunobloting bands of immunoprecipitated EBF2. Then, the fold changes were calculated by dividing the relative density of persulfidated EBF2 in Cre-expressing *Cars2*^flox/flox^ BAC by that of Vector-expressing *Cars2*^flox/flox^ BAC. (C) The relative amounts of protein in Figure 5N were determined by the density of the corresponding bands of protein, normalized to that of HSP90, Then, the fold changes were calculated by dividing the relative density of protein in the *Cars2*-KO BAC expressing vector, wild type and mutant EBF2 by that of Vec-expressing control BAC. (D)The Ebf2-Flag expression plasmid was transfected into wild-type and *CARS2*-KO 293T cells. Overexpressed EBF2 was pulled down with Flag-agarose after the biotin-HPDP switch assay, and the persulfidation of EBF2 was analyzed by western blotting. (E) The relative amounts of persulfidated EBF2 in D were determined by the density of biotin immunobloting bands of immunoprecipitated EBF2, normalized to the density of EBF2 immunobloting bands of immunoprecipitated EBF2. Then, the fold changes were calculated by dividing the relative density of persulfidated EBF2 in *CARS2-*KO 293T cells by that of vector-expressing 293T cells. (F) The relative amounts of PPARγ and BRG1 interacted with EBF2 in Figure 6A were determined by the density of myc immunobloting bands of immunoprecipitated EBF2, normalized to the density of EBF2 immunobloting bands of immunoprecipitated EBF2. Then, the fold changes were calculated by dividing the relative density of PPARγ and BRG1 interacted with EBF2 in *CARS2-*KO 293T cells by that of vector-expressing 293T cells. (G) Wild-type Ebf2 (Flag-Ebf2-WT) and vector was overexpressed in primary BAC isolated from the iBAT of newborn *Cars2*^flox/flox^ mice. Vector or Cre was expressed in precursor cells as indicated. After differentiation, the interaction of endogenous PPARγ or BRG1 with exogenous EBF2 in the presence or absence of *Cars2* in mature mouse BAC was determined via an immunoprecipitation assay. (H) The relative amounts of PPARγ and BRG1 interacted with EBF2 in G were determined by the density of PPARγ or BRG1 immunobloting bands of immunoprecipitated EBF2, normalized to the density of EBF2 immunobloting bands of immunoprecipitated EBF2. Then, the fold changes were calculated by dividing the relative density of PPARγ and BRG1 interacted with EBF2 in Cre-expressing cells by that of vector-expressing cells. (I-L) The interaction of PPARγ (I) or BRG1 (K) with wild-type EBF2 or mutant EBF2 (containing cysteine 160, 163 and 169 to alanine) was determined via an immunoprecipitation assay. The relative amounts of PPARγ (J) and BRG1 (L) interacted with EBF2 in I and K were determined by the density of myc immunobloting bands of immunoprecipitated EBF2, normalized to the density of EBF2 immunobloting bands of immunoprecipitated EBF2. Then, the fold changes were calculated by dividing the relative density of interacted PPARγ and BRG1 in EBF2 mutant expressing 293T cells by that of wild type EBF2 expressing 293T cells. (M) The relative amounts of protein in Figure 6G were determined by the density of the corresponding bands of protein, normalized to that of HSP90, Then, the fold changes were calculated by dividing the relative density of protein in *Cars2*-KO and control BAC treated with or without Rosi by that of control BAC treated with vehicle. (N) The hydrogen sulfide and sulfane sulfur levels of primary BAC isolated from the iBAT of newborn *Cars2*^flox/flox^ mice expressing Vec or Cre recombinase in precursor cells after differentiation were stained with AzMC and SSP4 respectively; the nucleus was stained with Hoechst 33342 (for SSP4) and SYTO9 (for AzMC); and fluorescence images were taken via confocal microscopy. Scale bar=20 µm. (O) The relative amounts of UCP1 protein in Figure 7C were determined by the density of UCP1 bands, normalized to that of HSP90, Then, the fold changes were calculated by dividing the relative density of UCP1 in GYY treated BAC by that of Vehicle treated BAC. (P) The relative amounts of UCP1 protein in Figure 7F were determined by the density of UCP1 bands, normalized to that of HSP90, Then, the fold changes were calculated by dividing the relative density of UCP1 in ZnCl_2_ treated BAC by that of Vehicle treated BAC. (Q) The relative amounts of persulfidated EBF2 in Figure 7G were determined by the density of biotin immunobloting bands of immunoprecipitated EBF2, normalized to the density of EBF2 immunobloting bands of immunoprecipitated EBF2. Then, the fold changes were calculated by dividing the relative density of persulfidated EBF2 in GYY treated 293T cells by that of vehicle treated 293T cells. (R) The relative amounts of PPARγ interacted with EBF2 in Figure7H were determined by the density of myc immunobloting bands of immunoprecipitated EBF2, normalized to the density of EBF2 immunobloting bands of immunoprecipitated EBF2. Then, the fold changes were calculated by dividing the relative density of interacted PPARγ in GYY and ZnCl_2_ treated 293T cells by that of vehicle treated 293T cells. Data are shown as the mean ± SEM. *p < 0.05, **p < 0.01, and ***p < 0.001, by unpaired Student’s t test (B, E, F, H, J, L, O-Q); by one-way ANOVA with dunnett's test (C and R); ns, nonsignificant.

**Figure S12. Glucose tolerance test and insulin tolerance test in GYY- and PLP-treated mice following high-fat diet feeding.** (A-B) The glucose tolerance test (GTT, A) and insulin tolerance test (ITT, B) were performed in Veh- and GYY-treated mice after HFD feeding for 12 weeks and 13 weeks, respectively. The relative glucose level expressed as a percentage of the baseline was calculated by dividing each mouse’s glucose level by the average glucose level of the vehicle (Veh)-treated group. (C) The relative amounts of UCP1 and CARS2 protein in Figure 7P were determined by the density of UCP1 and CARS2 bands, normalized to that of HSP90. Then, the fold changes were calculated by dividing the relative protein density of iBAT from GYY treated mice by that of vehicle treated mice. (D-E) GTT (D) and ITT (E) assays were performed in Veh- and PLP-treated mice after HFD feeding for 9 weeks and 10 weeks, respectively. The relative glucose level expressed as a percentage of the baseline was calculated by dividing each mouse’s glucose level by the average glucose level of the vehicle (Veh)-treated group. (F) The relative amounts of UCP1 and CARS2 protein in Figure 7X were determined by the density of UCP1 and CARS2 bands, normalized to that of HSP90, Then, the fold changes were calculated by dividing the relative protein density of iBAT from PLP treated mice by that of vehicle treated mice. Data are shown as the mean ± SEM. *p < 0.05, **p < 0.01, and ***p < 0.001, by unpaired Student’s t test (C and F); by two-way ANOVA with multiple comparisons (A-B and D-E)；ns, nonsignificant

**Figure S13. Treating GYY and PLP in mice mitigates obesity progression and promotes energy expenditure but does not influence food intake or activity**

(A-C) Wild-type mice were fed on high-fat diet (HFD) for 14 weeks and intraperitoneally injected with GYY4137 (GYY, 120 mg/kg, n=6), pyridoxal-5′-phosphate (PLP, 75 mg/kg, n=6) or saline (Veh, n=6) daily. Energy expenditure (A), food intake (B) and activity (C) were measured in metabolic cages. (D) Fat mass and lean mass of wild-type mice treated with GYY, PLP or Veh in A-C after HFD feeding. Data are shown as the mean ± SEM. *p < 0.05 and ***p < 0.001 by one-way ANOVA with dunnett's test (B, C, and D); by two-sided ANCOVA analysis (A); ns, nonsignificant.
